# 4-Sulfanyl-1,2,3-triazole as a Powerful Ligand in CuAAC to Synthesize 1,4-Substituted 1,2,3-Triazoles Under Solvent-Free and Low Catalyst Loading

**DOI:** 10.3390/molecules31101723

**Published:** 2026-05-19

**Authors:** Jie Shen, Jinwei Li, Shitang Xu, Ting Wang, Zhiling Zou, Hui Li, Lifen Peng, Zilong Tang, Xinhua Xu

**Affiliations:** 1 State Key Laboratory of Chemo/Biosensing and Chemometrics, College of Chemistry and Chemical Engineering, Hunan University, Changsha 410082, China; jieshen@hnu.edu.cn (J.S.); lijinwei@hnu.edu.cn (J.L.); xst978@hnu.edu.cn (S.X.); huil23@hnu.edu.cn (H.L.); 2 Key Laboratory of Theoretical Organic Chemistry and Functional Molecule of Ministry of Education, Key Laboratory of Molecular Design and Green Chemistry of Hunan Provincial Universities, Hunan Provincial Key Laboratory of Controllable Preparation and Functional Application of Fine Polymers, School of Chemistry and Chemical Engineering, Hunan University of Science and Technology, Xiangtan 411201, China; 23010601032@mail.hnust.edu.cn (T.W.); 23010601035@mail.hnust.edu.cn (Z.Z.)

**Keywords:** 4-sulfanyl-1,2,3-triazole, solvent-free, *N*-sulfonyl-1,2,3-triazole, ligand

## Abstract

4-Sulfanyl-1,2,3-triazole (**L1**) accelerated the solvent-free CuAAC efficiently with low catalyst loading (0.1 mol% for common azides and 1 mol% for sulfonyl azides). **L1** exhibited higher catalytic activity compared to 1,4-substituted 1,2,3-triazole without sulfanyl group (**5a**) and sulfide, demonstrating that coordination of both sulfanyl and 1,2,3-triazole moieties with copper was critical to enhance the activity of **L1**. The Cu(OAc)_2_/**L1** catalytic system displayed high selectivity in synthesis of alkynyl- or azido-involved 1,2,3-triazoles. The di-copper system Cu(OAc)_2_/CuBr/**L1** promoted the reaction of electron-deficient and less reactive sulfonyl azides well, generating *N*-sulfonyl-1,2,3-triazoles in good yields, and **L1** showed better performance than 1,3-di-o-tolylthiourea (**L′**). Other features of this protocol included recyclable ligand, 1:1 substance ratio, high yields, wide substance scope, and easily scaled up and facile purification of most products.

## 1. Introduction

**Scheme 1:**
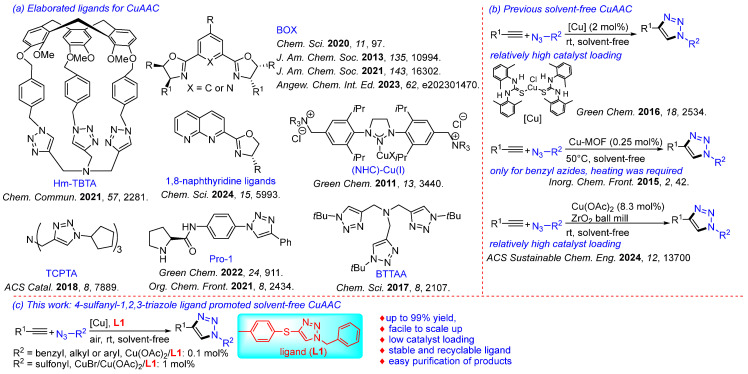
Elaborated ligands and solvent-free CuAAC [14,15,16,17,18,19,20,21,22,23,24,27,28,29].

1,4-Substituted 1,2,3-triazoles are potent moieties in bioactive compounds like agrochemicals and pharmaceuticals because they serve as facile-to-install pharmacophores and display various biological activities [1,2]. Cu-catalyzed azide–alkyne cycloaddition (CuAAC) [3,4], a valuable protocol to construct 1,4-substituted 1,2,3-triazoles, has been widely applied in synthetic chemistry [1,5], glycoscience [6], chemosensors [7], material science [8], protein science [9], biochemistry [10], drugs [11] and agrochemicals [12]. Traditional CuAAC is usually carried out in organic solvents with copper salt or complex as a catalyst and an excess amount of amine as a base [13]. To promote CuAAC efficiently in the absence of excess amine, several ligands have been developed in recent years (Scheme 1a); for example, tris-(benzyltriazolylmethyl)amine (Hm-TBTA) promoted CuAAC efficiently with 1 mol% catalyst loading [14]. Bisoxazoline (BOX) derivatives (catalyst loading, [Cu]: 10 mol%, ligand: 12%) [15,16,17,18], 1,8-naphthyridines (catalyst loading, [Cu]: 10 mol%, ligand: 13%) [19] and tris((1-cyclopentyl-1H-1,2,3-triazol-4-yl)methyl)amine (TCPTA) (catalyst loading, 5 mol%) [20] were fabricated as well, but most of these reactions proceeded in volatile organic solvents with relatively high catalyst loading. Although *N*-heterocyclic carbene (NHC)-Cu(I) (catalyst loading, 5 mol%) [21] and triazolyl-prolinamide (Pro-1) (catalyst loading, [Cu]: 5 mol%, ligand: 10%) [22,23] could promote CuAAC in H_2_O, the catalyst loading also remained to improve. Tris(triazolylmethyl)amine (BTTAA) enabled CuAAC to be performed inside live cells at very low concentrations, but Na ascorbate was required [24]. Moreover, most of the above ligands were somewhat complex and difficult to synthesize. Thus, developing other simple, readily manufactured and powerful ligands was still required for the importance of CuAAC. On the other hand, establishing solvent-free CuAAC with very low catalyst loading at rt not only reduced cost and energy consumption, but also was necessary for conducting biorthogonal CuAAC in living organisms due to the toxicity of copper catalyst [25,26]. Recently, solvent-free CuAAC protocols were developed using 1,3-bis(2,6-dimethylphenyl)thiourea-based copper [27], Cu-MOF [28] and ZrO_2_ ball mill/Cu(OAc)_2_ [29] catalytic systems (Scheme 1b). But these reactions suffered some drawbacks like relatively high catalyst loading and heating. Establishing solvent-free CuAAC with wide substance scope under rt and low catalyst loading was still challenging. 4-Sulfanyl-1,2,3-triazoles were firstly and systematically synthesized by Peng and Orita from 1-phosphinyl-2-sulfanylethynes which were obtained from ethynyl(diphenyl) phosphine oxide by bromination and coupling with thiols [30], but the one-pot synthesis of 4-sulfanyl-1,2,3-triazoles from commercially available (bromoethynyl)triisopropylsilane and their application as ligands were not disclosed. Herein, 4-sulfanyl-1,2,3-triazole (**L1**), which was accessed facilely by a one-pot desilylation/CuAAC protocol, was developed as a powerful ligand for CuAAC (Scheme 1c). To our delight, **L1** enabled the solvent-free CuAAC to be carried out smoothly with a 1:1 substance ratio under rt and low catalyst loading, producing various 1,4-substituted 1,2,3-triazoles including bioactive compounds, azido- or alkynyl-functionalized 1,2,3-triazoles and *N*-sulfonyl-1,2,3-triazoles in high yields.

## 2. Results and Discussion

**Scheme 2:**
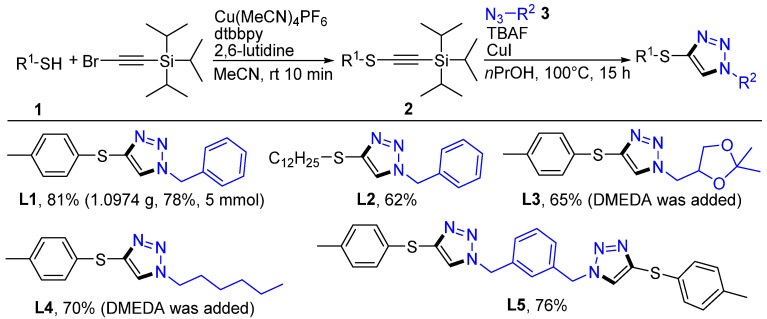
Synthesis of ligands.

Initially, we synthesized 4-sulfanyl-1,2,3-triazoles using a one-pot method (Scheme 2). The Cu(MeCN)_4_PF_6_-catalyzed cross-coupling between thiols (**1**) and (bromoethynyl)triisopropylsilane afforded sulfanylethynes (**2**) [31], and the following one-pot desilylation/CuAAC between **2** and benzyl azide (**3a**) formed the desired **L1** and **L2** in 81% and 62% yields, respectively. When 1,3-bis(azidomethyl)benzene (0.4 equiv.) was applied, bis(4-sulfanyl-1,2,3-triazoles) (**L5**) was obtained in 76% yield. Adding *N*,*N*′-dimethyl-1,2-ethanediamine (DMEDA) realized the formation of **L3** and **L4** in reasonable yields. This reaction was facile for a gram-scale synthesis, with 0.6021 g (5 mmol) of **1a** producing 1.0974 g (3.9 mmol) of **L1** in 78% yield.

**Scheme 3:**
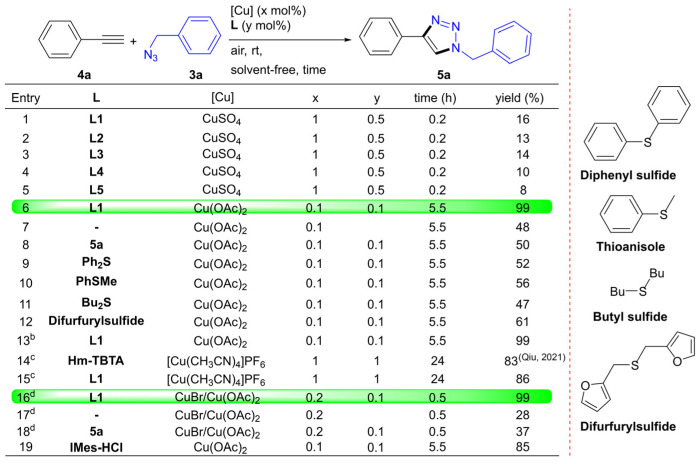
Optimization of the reaction conditions of CuAAC between **4a** and **3a** ^a^. ^a^ **4a** (2.0 mmol), **3a** (2.0 mmol), isolated yields. ^b^ **4a** (10.0 mmol), **3a** (10.0 mmol). ^c^ **4a** (0.2 mmol), **3a** (0.2 mmol), glutathione (0.04 mmol), CH_2_Cl_2_ (2 mL), CH_3_OH (0.2 mL). ^d^ CuBr (0.1 mol%), Cu(OAc)_2_ (0.1 mol%) [14].

The solvent-free CuAAC of ethynes (**4**) and azides (**3**) using **L1**–**L5** as ligands was then performed (Scheme 3). When CuSO_4_/**L1** was used, the desired **5a** was formed in 16% yield from phenylacetylene (**4a**) and (azidomethyl)benzene (**3a**) (entry 1). Among **L1**–**L5**, **L1** gave the highest yield (entry 1–5). Notably, **L1** displayed better performance than **5a**, Ph_2_S, PhSMe, Bu_2_S and difurfurylsulfide (entry 6 vs. 8–12, 16 vs. 18), demonstrating that the coordination of both sulfanyl and 1,2,3-triazole moieties with copper was critical to enhance the catalytic performance of **L1**. The di-copper system (Cu(OAc)_2_/CuBr/**L1**) enabled the reaction to complete in 0.5 h and showed higher catalytic activity than the mono-copper catalytic system (Cu(OAc)_2_/**L1**) (entry 16 vs. 6). Without ligand, **5a** was formed in 48% yield in the mono-copper system within 5.5 h and 28% yield in the di-copper system within 0.5 h (entries 7 and 17). In the reported CuAAC in CH_2_Cl_2_/CH_3_OH [12], **L1** also exhibited higher activity than Hm-TBTA (entry 15 vs. 14). After examining the reaction carefully (seen in Supplementary Materials), the optimized conditions were obtained as follows: a mixture of **4a** (2.0 mmol), **3a** (2.0 mmol), Cu(OAc)_2_ (0.002 mmol) and **L1** (0.002 mmol), at rt under air for 5.5 h, giving **5a** in 99% yield (0.4659 g) (entry 6). In a gram-scale synthesis, 1.0213 g (10.0 mmol) of **4a** formed 2.3294 g (9.9 mmol) of **5a** in 99% yield (entry 13).

**Scheme 4:**
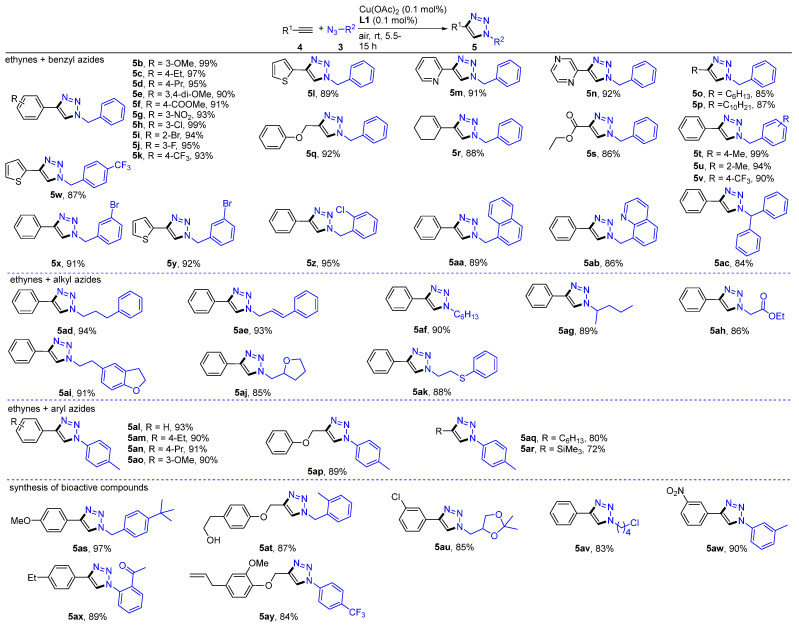
The solvent-free CuAAC of **4** and **3** ^a^. ^a^ **4** (2.0 mmol), **3** (2.0 mmol), isolated yields.

The substance scope was then extended (Scheme 4). The CuAAC of benzyl azides was firstly investigated. Phenyl ethynes with electron-donating (OMe, Et, Pr) and electron-withdrawing (MeOC(O), NO_2_, Cl, Br, F, CF_3_) groups gave **5b**–**5k** in 90–99% yields. 2-Ethynyl-substituted thiophene, pyridine and pyrazine gave **5l**–**5n** in 89–92% yields. Alkyl ethynes formed **5o**–**5q** in excellent yields. Alkenyl and ester groups were tolerated, forming **5r** and **5s** in 88% and 86% yields, respectively. When benzyl azides with Me, CF_3_, Br or Cl groups were used, **5t**–**5z** were obtained in 87–99% yields. Naphthyl and quinolyl azides gave **5aa** and **5ab** in 89% and 86% yields, respectively. Diphenylmethyl azide survived to produce **5ac** in 84% yield. Moreover, the less reactive alkyl and phenyl azides [32] were applicable as well. Primary and secondary alkyl azides provided **5ad**–**5ah** in 86–94% yields. 2,3-Dihydrobenzofuranyl-, tetra-hydrofuranyl- and phenylthiol-involved alkyl azides afforded **5ai**–**5ak** in excellent yields. The reaction of 1-azido-4-methylbenzene and ethynes formed **5al**–**5aq** in 80–93% yields. Trimethylsilylacetylene was also tolerated to form **5ar** in 72% yield. Bioactive compounds were also obtained: **5as** (97% yield) with heat shock protein 90-inhibitory activities [33], **5at** (87%) with phytotoxic activity [34], **5au** (85%) with fungicide activity [11], **5av** (83%) with anti-plasmodial activity [35], **5aw** (90%) inhibiting the Nrf2-Keap1 protein–protein interaction [36], **5ax** (89%) with antidepressant-like effect [37], and **5ay** (84%) reducing the mycelial growth of colletotrichum gloeosporioides [38]. All products were purified by washing, which avoided the tedious column chromatography.

**Scheme 5:**
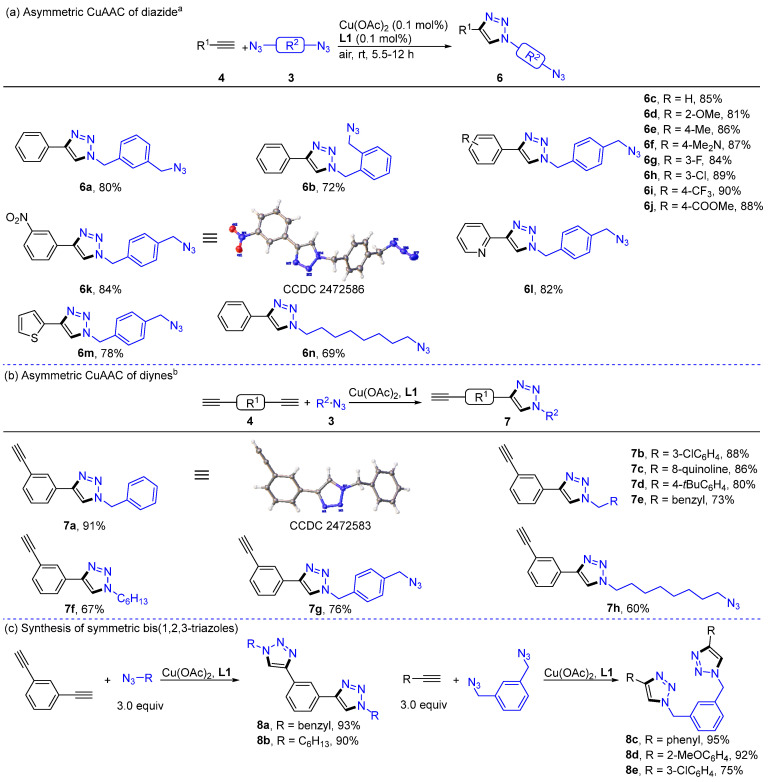
Asymmetric CuAAC and synthesis of bis(1,2,3-triazoles). ^a^ **4** (2.0 mmol), **3** (2.0 mmol), isolated yields. ^b^ **4** (2.0 mmol), **3** (2.0 mmol), isolated yields.

Generally, alkynyl- or azido-involved 1,2,3-triazoles, versatile intermediates in organic synthesis, were difficult to synthesize via asymmetric CuAAC in organic solvents for the further conversions of dissolved products [15,16,17,18,19,20,39,40,41]. In this regard, our solvent-free protocol exhibited high selectivity due to the tiny reactions of precipitated products. As disclosed in Scheme 5, the reaction of ethynes and diazides formed 1,2,3-triazoles (**6**) bearing an unreacted azido in good yields. Meta-, ortho- and para-substituted benzyl diazides were applicable, giving **6a**–**6c** in 72–85% yields. Ethynes with electron-donating (OMe, Me, Me_2_N) and electron-withdrawing (F, Cl, CF_3_, MeO(O)C, NO_2_) groups afforded **6d**–**6k** in 81–90% yields. 2-Ethynylpyridine and 2-ethynylthiophene were tolerated, forming **6l** and **6m** in good yields. Alkyl diazide was suitable as well (**6n**, 69% yield). Subjection of diyne to this reaction produced 1,2,3-triazole (**7**) with an unaltered alkynyl in high yields. Benzyl and alkyl azides afforded **7a**–**7f** in 67–91% yields. The reaction of diazides and 1,3-diethynylbenzene proceeded well to afford **7g** and **7h** in moderate yields. In most cases, the azido in **6** and alkynyl in **7** were unconverted for the deposition of products. Treatment of 1,3-diethynylbenzene with benzyl azide and 1-azidohexane afforded bis(1,2,3-triazoles) (**8a**, **8b**) in 93% and 90% yields, respectively. When 1,3-bis(azidomethyl)benzene was employed, **8c** was formed in 95% yield. Similarly, **8d** and **8e** were also provided in high yields.

**Scheme 6:**
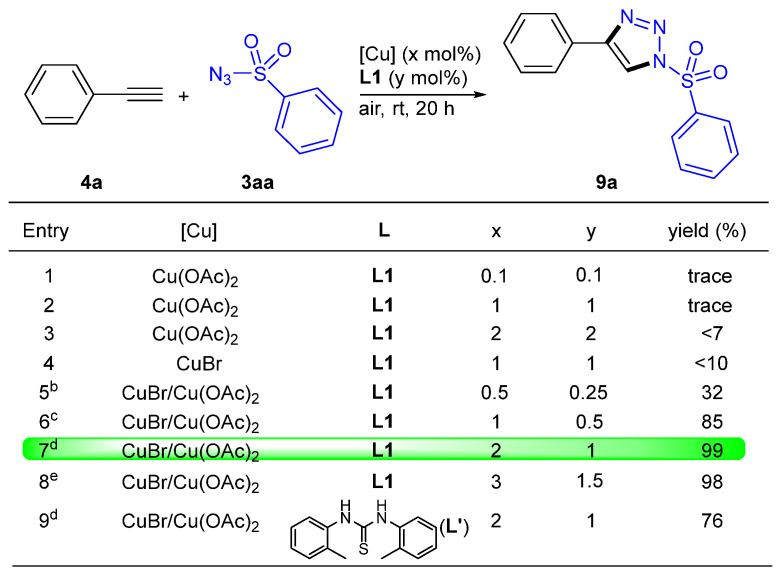
Optimization conditions of CuAAC between **4a** and **3aa** ^a^. ^a^ **4a** (1.0 mmol), **3aa** (1.0 mmol), isolated yields. ^b^ CuBr (0.25 mol%), Cu(OAc)_2_ (0.25 mol%). ^c^ CuBr (0.5 mol%), Cu(OAc)_2_ (0.5 mol%). ^d^ CuBr (1 mol%), Cu(OAc)_2_ (1 mol%). ^e^ CuBr (1.5 mol%), Cu(OAc)_2_ (1.5 mol%).

*N*-Sulfonyl-1,2,3-triazoles were not only key synthons in denitrogenative transformation [42] but also displayed antitumor activity [43]. Sulfonyl azides displayed lower reactivities in CuAAC than common azides due to electron deficiency. In the reaction between **4a** and benzenesulfonyl azide (**3aa**), *N*-sulfonyl-1,2,3-triazole (**9a**) was formed in less than 7% yield in the presence of Cu(OAc)_2_/**L1** even when the catalyst loading was elevated to 2 mol% (Scheme 6, entry 1–3). CuBr/**L1** also provided **9a** in less than 10% yield (entry 4). Combination of Cu(OAc)_2_, CuBr and **L1** with catalyst loading of 0.25 mol% enhanced the yield of **9a** to 32% (entry 5). Increasing the catalyst loading to 1.0 mol% gave the best yield (entry 7 vs. 6 and 8). However, ligand 1,3-di-o-tolylthiourea (**L′**) afforded **9a** in 76% yield (entry 9). The best conditions were provided as follows: a mixture of **4a** (1.0 mmol), **3aa** (1.0 mmol), Cu(OAc)_2_ (0.01 mmol), CuBr (0.01 mmol) and **L1** (0.01 mmol), at rt under air for 20 h, producing **9a** in 99% yield (0.2825 g) (entry 7).

**Scheme 7:**
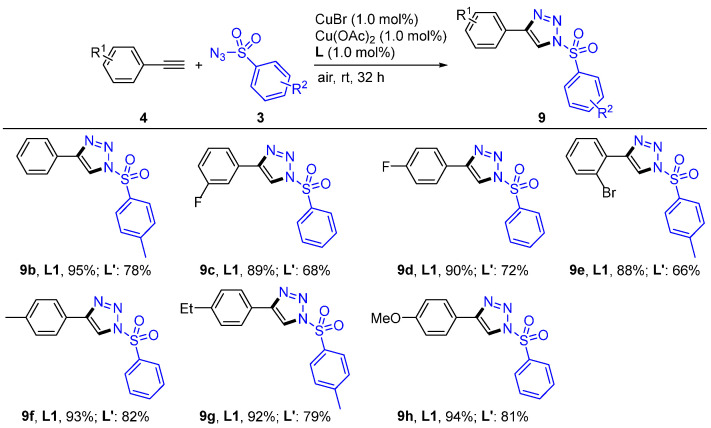
The solvent-free CuAAC of ethynes with sulfonyl azides.

With **L1** as a ligand, phenyl ethynes with either electron-withdrawing (F, Br) or electron-donating (Me, Et, OMe) group were applicable under the above best reaction conditions, producing the corresponding **9c**–**9h** in 88–94% yields, while **9c**–**9h** were generated in 66–82% yields when 1,3-di-o-tolylthiourea (**L′**) was employed. These above results demonstrated that **L1** displayed better performance than **L′** in the solvent-free CuAAC of sulfonyl azides (Scheme 7).

**Scheme 8:**
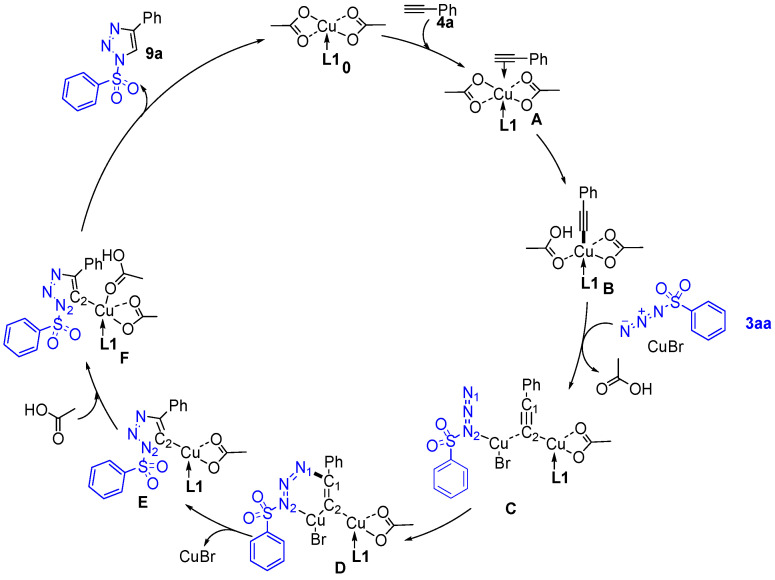
The proposed mechanism for solvent-free CuAAC of ethyne **4a** with sulfonyl azide **3aa**.

A plausible mechanism for the above di-copper system was proposed. Firstly, activation of Cu(OAc)_2_ by ligand (**L1**) generated **L1**-Cu(OAc)_2_ (**0**). Coordination of **0** with **4a** formed the intermediate **A**, which then underwent H atom abstraction from ethyne by OAc and produced **B**. Di-copper intermediate **C** was formed by incorporation of CuBr and **3aa** with **B** with elimination of HOAc. Intramolecular cyclization of **C** gave the six-membered metallacycle **D** by C1–N1 bond formation. Formation of C2–N2 in **D** gave the five-membered cycle **E** with the liberation of CuBr. Coordination of HOAc with **E** yielded **F**, which underwent H transformation to afford **9a** and regenerate the catalyst (**0**) (Scheme 8). The coordination of both sulfanyl and 1,2,3-triazole moieties in **L1** with copper was critical to enhance the activity of **L1** possibly due to the formation of more-stable six-membered metallacycle **D**, which then lowered the activation energy of this CuAAC reaction.

**Figure 1:**
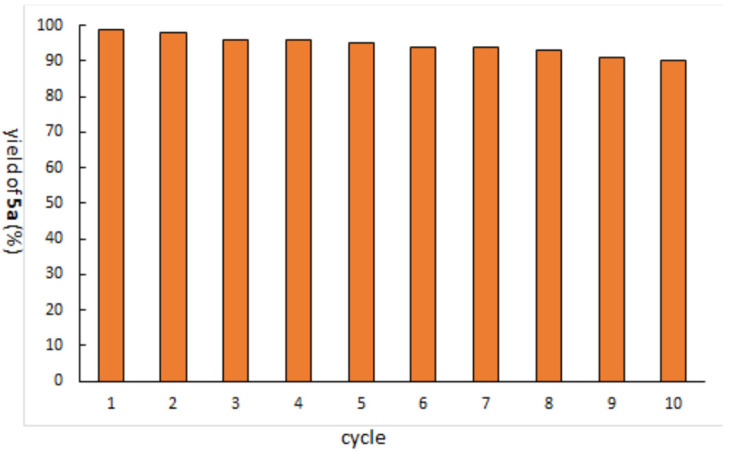
The recyclability of **L1**.

The ligand **L1** could be recycled using the reaction of **4a** and **3a** to synthesize **5a** as a model (10 mmol scale). After workup with NH_4_Cl aq. and extracting with ethyl acetate, the recycled ligand **L1** was pure enough and could be reused without further purification. Subjecting the recycled **L1** to the second cycle afforded **5a** in 98% yield. **L1** could be reused for up to ten successive cycles. In this cycle, we found that it took 8 h to achieve 95% yield up to five cycles. After five catalytic cycles, 9 h was required to achieve excellent yields (Figure 1).

## 3. Materials and Methods

All reagents were commercially available and used without further purification. The dehydrated solvents were purchased and redistilled before use. Glassware was dried in an oven and heated under reduced pressure before use. Column chromatography was undertaken on silica gel (300–400 mesh) using a proper eluent system. Analytical thin-layer chromatography (TLC) was performed on Haiyang TLC silica gel GF254 (Qingdao, China, 0.25 mm) plates. Proton, carbon and fluorine nuclear magnetic resonance spectra (^1^H, ^13^C and ^19^F NMR) were recorded on a Bruker-400 (Billerica, MA, USA, 400 MHz for ^1^H NMR, 101 MHz for ^13^C NMR and 376 MHz for ^19^F NMR spectroscopy) spectrometer with solvent resonance as the internal standard (^1^H NMR, CDCl_3_ at 7.26 ppm, DMSO-*d*_6_ at 2.50 ppm; ^13^C NMR, CDCl_3_ at 77.16 ppm, DMSO-*d*_6_ at 39.52 ppm). Chemical shifts are reported in ppm (δ) relative to internal tetramethylsilane (TMS). Data are reported as follows: chemical shift, multiplicity (s = singlet, d = doublet, t = triplet, q = quartet, m = multiplet); coupling constants (*J*) in hertz. Melting points were measured using a melting point meter RY-1G. Crystal data were acquired at 296 K on a Rigaku Oxford Diffraction Supernova Dual Source (Akishima, Japan), Cu at zero, equipped with an Atlas S2 CCD using Cu Kα radiation. HRMS data were acquired using the Waters G2-Xs qtof mass spectrometer (Milford, MA, USA) under Electron Spray Ionization conditions. Ethynes **4y** [34] and **4z** [38] were prepared according to the reported methods; other ethynes **4a**–**4x** were purchased from Energy, SigmaAldrich (St. Louis, MO, USA) and Leyan (Shanghai, China).

Procedure for Synthesis of **L1**–**L5**. Cu(MeCN)_4_PF_6_ (0.1 mmol) was dissolved in degassed acetonitrile (9 mL) completely under nitrogen. A Schlenk tube of 100 mL equipped with a magnetic stir bar was charged with (bromoethynyl)triisopropylsilane (1.0 or 2.0 mmol), thiol **1** (1.1 or 2.2 mmol), dtbbpy (0.2 mmol), 2,6-lutidine (2.0 mmol) and degassed acetonitrile (20 mL) under nitrogen. Then the above acetonitrile solution of Cu(MeCN)_4_PF_6_ was added to the Schlenk tube. The mixture was stirred at rt for 15–30 min, and then was concentrated under vacuum to provide a crude reaction mixture. The residue was extracted with EtOAc and saturated NH_4_Cl aqueous solution. The organic layer was washed with brine and dried over Na_2_SO_4_. The solvents were evaporated after filtration. The crude residue was subjected to short-column chromatography on silica gel (hexanes) to extract alkynyl sulfides **2** [24], which were used for the next step without further purification. The crude product **2** was dissolved in THF (10 mL) and TBAF (1.0 M in THF, 1.0 mmol) was added at 0 °C. The mixture was stirred at rt for 10 h. Then CuI (0.1 mmol), azide **3** (1.0 mmol) and *n*PrOH (20 mL) were added, and the mixture was stirred at 100–110 °C for 15 h. The product was extracted with EtOAc after the mixture was quenched with saturated NH_4_Cl aqueous solution. The combined organic layer was washed with brine and dried over Na_2_SO_4_. The solvents were evaporated after filtration. The crude residue was subjected to column chromatography on silica gel (petroleum ether/EtOAc, 4:1) to extract **L1**–**L5** in pure forms.

General Procedure for the Synthesis of **5**, **6**, **7** and **8**. A mixture of Cu(OAc)_2_, **L1** (0.0006 g, 0.002 mmol), **4** (2.0 mmol) and **3** (2.0 mmol) was stirred under air at rt for 5.5–15 h. Then the reaction mixture was washed with water twice followed by cold petroleum ether (or petroleum ether/ethyl acetate (10/1)) three times or the reaction mixture was subjected to column chromatography on silica gel to produce the desired **5a**–**5ay** (purified by washing with water and cold petroleum ether), **6a**–**6n** (purified by column chromatography), **7a**–**7h** (purified by column chromatography), and **8a**–**8e** (purified by washing with water and petroleum ether/ethyl acetate (10/1)) in pure forms.

General Procedure for the Synthesis of **9**. A mixture of CuBr, Cu(OAc)_2_, **L1**, **4** and **3** was stirred under air at rt for 20 h or 32 h. Then the reaction mixture was washed with water twice followed by petroleum ether three times to produce the desired **9** in pure form.

## 4. Conclusions

In summary, 4-sulfanyl-1,2,3-triazole (**L1**) with excellent catalytic performance in CuAAC was obtained facilely in gram scale using a one-pot method. Using Cu(OAc)_2_/**L1** as a catalytic system, the solvent-free CuAAC between common azides and ethynes with a substance ratio of 1:1 proceeded smoothly at rt under air and low catalyst loading conditions, which not only avoided volatile organic solvents and reduced cost and energy consumption, but also exhibited high atomic economy. The di-copper system Cu(OAc)_2_/CuBr/**L1** accelerated the reaction of sulfonyl azides well, generating *N*-sulfonyl-1,2,3-triazoles in high yields.

## Data Availability

The original contributions presented in this study are included in the article and Supplementary Materials. Further inquiries can be directed to the corresponding authors.

## Supplementary Materials

The following supporting information can be downloaded at https://www.mdpi.com/article/10.3390/molecules31101723/s1, Figure S1: ORTEP drawing of **6k**; Table S1: Optimization the reaction conditions of CuAAC between **4a** and **3a**; Figure S2: ORTEP drawing of **7a**. Refs. [44,45,46,47,48,49,50,51,52,53,54,55,56,57,58,59,60,61,62,63,64,65,66,67,68,69,70,71,72,73,74,75,76,77,78,79,80,81,82,83,84,85,86,87,88] are cited in the Supplementary Materials.
