# Supplementary material for: 4-Sulfanyl-1,2,3-triazole as a Powerful Ligand in CuAAC to Synthesize 1,4-Substituted 1,2,3-Triazoles Under Solvent-Free and Low Catalyst Loading
Source: Molecules. 2026 May 19;31(10):1723. doi: 10.3390/molecules31101723 (PMC13209198; doi:10.3390/molecules31101723)

Supporting Information

4-Sulfanyl-1,2,3-triazole as a powerful ligand in CuAAC to synthesize 1,4-substituted 1,2,3-triazoles under solvent-free and low catalyst loading

Jie Shen[1], Jinwei Li[1], Shitang Xu[1], Ting Wang[2], Zhiling Zou[2], Hui Li[1], Lifen Peng[2,*], Zilong Tang[2,*], Xinhua Xu[1,*]

[a.] *State Key Laboratory of Chemo/Biosensing and Chemometrics, College of Chemistry and Chemical Engineering, Hunan University, Changsha 410082, P. R. China.*

[b.] *Key Laboratory of Theoretical Organic Chemistry and Functional Molecule of Ministry of Education, Key Laboratory of Molecular Design and Green Chemistry of Hunan Provincial Universities, Hunan Provincial Key Laboratory of Controllable Preparation and Functional Application of Fine Polymers, School of Chemistry and Chemical Engineering, Hunan University of Science and Technology, Xiangtan, Hunan 411201, China.*

Table of Contents

1. General Information 1

2. General Procedure for Synthesis of Starting Material Azide 1

3. General Procedure D for Preparation of 4-Sulfanyl-1,2,3-triazoles (**L1**–**L5**)............ 6

4. Optimization the reaction conditions of CuAAC between **4a** and **3a** 8

5. General Procedure E for Synthesis of 1,2,3-Triazoles (**5a**–**5ay**, **6a**–**6n**, **7a**–**7h** and **8a**–**8e**) 9

6. General Procedure F for Synthesis of *N*-Sulfonyl-1,2,3-triazoles (**9a**–**9h**) 29

7. X-Ray Crystallographic Data 31

8. References 34

9. NMR Copies of All Compounds. 38

1. General Information

All reagents were commercially available and used without further purification. The dehydrated solvents were purchased and redistilled before use. Glassware was dried in an oven and heated under reduced pressure before use. Column chromatography was undertaken on silica gel (300-400 mesh) using a proper eluent system. Analytical thin layer chromatography (TLC) was performed on Haiyang TLC silica gel GF254 (0.25 mm) plates. Proton, carbon and fluorine nuclear magnetic resonance spectrum (^{1}H, ^{13}C and ^{19}F NMR) were recorded on a Bruker-400 (400 MHz for ^{1}H NMR, 101 MHz for ^{13}C NMR and 376 MHz for ^{19}F NMR spectroscopy) spectrometer with solvent resonance as the internal standard (^{1}H NMR, $CDCl_3$ at 7.26 ppm, DMSO-d_6 at 2.50 ppm; ^{13}C NMR, $CDCl_3$ at 77.16 ppm, DMSO-d_6 at 39.52 ppm). Chemical shifts are reported in ppm (δ) relative to internal tetramethylsilane (TMS). Data are reported as follows: Chemical shift, multiplicity (s = singlet, d = doublet, t = triplet, q = quartet, m = multiplet), Coupling constants (*J*) are reported in hertz. Melting points were measured using a melting point meter RY-1G. Crystal Data were acquired at 296 K on a Rigaku Oxford Diffraction Supernova Dual Source, Cu at Zero equipped with an Atlas S2 CCD using Cu Kα radiation. HRMS data were acquired using the waters G2-Xs qtof mass spectrometer under Electron Spray Ionization conditions. Ethynes **4y**[1] and **4z**[2] were prepared according to the reported methods, other ethynes **4a**–**4x** were purchased from Energy, SigmaAldrich and Leyan.

2. General Procedure for Synthesis of Starting Material Azides

2.1 General Procedure A for the Synthesis of Azides 3a–3s, 3u–3z and 3ac

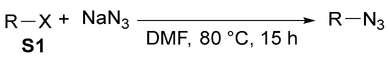

Under a nitrogen atmosphere, a Schlenk tube of 100 mL equipped with a magnetic stir bar was charged with **S1** (2.0 mmol), NaN_3 (3.0 or 6.0 mmol) and DMF (20 mL) under a nitrogen atmosphere. The reaction mixture was stirred at 80°C for 15 h. The product was extracted with Et_2O after the mixture was quenched with 10 mL of H_2O. The organic layer was washed with brine and dried over Na_2SO_4. The solvents were evaporated after filtration through a thin cake of silica gel. The crude residue was used for the following reaction without further purification.[3]

(azidomethyl)benzene (3a):[3] the crude residue was prepared according to the procedure A from (bromomethyl)benzene (0.3421 g, 2.0 mmol) and NaN_3 (0.1950 g, 3.0 mmol) as a colorless liquid in 95% crude yield (0.2530 g).

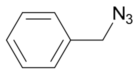

4-(azidomethyl)-2,2-dimethyl-1,3-dioxolane (3b):[4] The crude residue was prepared according to the procedure A from 4-(bromomethyl)-2,2-dimethyl-1,3-dioxolane (0.3901 g, 2.0 mmol) and NaN_3 (0.1950 g, 3.0 mmol) as a colorless liquid in 82% crude yield (0.2578 g).

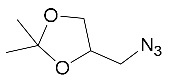

azidohexane (3c):[3] The crude residue was prepared according to the procedure A from 1-bromohexane (0.3301 g, 2.0 mmol) and NaN_3 (0.1950 g, 3.0 mmol) as a colorless liquid in 80% crude yield (0.2035 g).

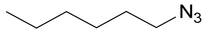

(azidomethyl)benzene (3d):[5] The crude residue was prepared according to the procedure A from 1,3-bis(bromomethyl)benzene (0.5279 g, 2.0 mmol) and NaN_3 (0.3900 g, 6.0 mmol) as a colorless liquid in 98% crude yield (0.3688 g).

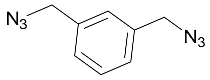

1-(azidomethyl)-4-methylbenzene (3e):[3] The crude residue was prepared according to the procedure A from 1-(bromomethyl)-4-methylbenzene (0.3701 g, 2.0 mmol) and NaN_3 (0.1950 g, 3.0 mmol) as a colorless liquid in 96% crude yield (0.2826 g).

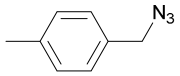

1-(azidomethyl)-2-methylbenzene (3f):[3] The crude residue was prepared according to the procedure A from 1-(bromomethyl)-2-methylbenzene (0.3701 g, 2.0 mmol) and NaN_3 (0.1950 g, 3.0 mmol) as a colorless liquid in 90% crude yield (0.2649 g).

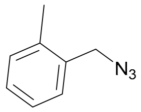

1-(azidomethyl)-4-(trifluoromethyl)benzene (3g):[3] The crude residue was prepared according to the procedure A from 1-(bromomethyl)-4-(trifluoromethyl)benzene (0.4781 mg, 2.0 mmol) and NaN_3 (0.1950 g, 3.0 mmol) as a colorless liquid in 97% crude yield (0.3902 g).

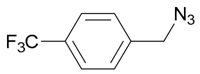

1-(azidomethyl)-3-bromobenzene (3h):[6] The crude residue was prepared according to the procedure A from 1-bromo-3-(bromomethyl)benzene (0.4999 g, 2.0 mmol) and NaN_3 (0.1950 g, 3.0 mmol) as a colorless liquid in 89% crude yield (0.3774 g).

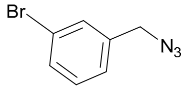

1-(azidomethyl)-2-chlorobenzene (3i):[7] The crude residue was prepared according to the procedure A from 1-(bromomethyl)-2-chlorobenzene (0.4110 g, 2.0 mmol) and NaN_3 (0.1950 g, 3.0 mmol) as a colorless liquid in 86% crude yield (0.2883 g).

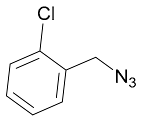

(azidomethyl)naphthalene (3j):[3] The crude residue was prepared according to the procedure A from 1-(bromomethyl)naphthalene (0.4422 g, 2.0 mmol) and NaN_3 (0.1950 g, 3.0 mmol) as a colorless liquid in 97% crude yield (0.3554 g).

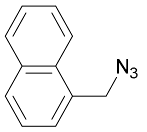

8-(azidomethyl)quinoline (3k):[8] The crude residue was prepared according to the procedure A from 8-(bromomethyl)quinoline (0.4442 g, 2.0 mmol) and NaN_3 (0.1950 g, 3.0 mmol) as a colorless liquid in 91% crude yield (0.3352 g).

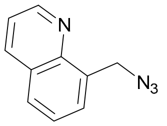

(azidomethylene)dibenzene (3l):[3] The crude residue was prepared according to the procedure A from (bromomethylene)dibenzene (0.4943 g, 2.0 mmol) and NaN_3 (0.1950 g, 3.0 mmol) as a colorless liquid in 94% crude yield (0.3934 g).

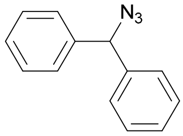

(3-azidopropyl)benzene (3m):[3] The crude residue was prepared according to the procedure A from (3-bromopropyl)benzene (0.3982 g, 2.0 mmol) and NaN_3 (0.1950 g, 3.0 mmol) as a colorless liquid in 90% crude yield (0.2902 g).

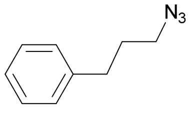

(3-azidoprop-1-en-1-yl)benzene (3n):[3] The crude residue was prepared according to the procedure A from (3-bromoprop-1-en-1-yl)benzene (0.3941 g, 2.0 mmol) and NaN_3 (0.1950 g, 3.0 mmol) as a colorless liquid in 87% crude yield (0.2770 g).

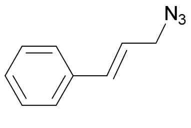

2-azidopentane (3o):[3] The crude residue was prepared according to the procedure A from 2-bromopentane (0.3021 g, 2.0 mmol) and NaN_3 (0.1950 g, 3.0 mmol) as a colorless liquid in 80% crude yield (0.1810 g).

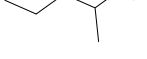

ethyl 2-azidoacetate (3p):[9] The crude residue was prepared according to the procedure A from ethyl 2-bromoacetate (0.3340 g, 2.0 mmol) and NaN_3 (0.1950 g, 3.0 mmol) as a colorless liquid in 85% crude yield (0.2195 g).

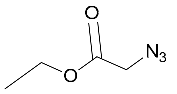

5-(2-azidoethyl)-2,3-dihydrobenzofuran (3q):[3] The crude residue was prepared according to the procedure A from 5-(2-bromoethyl)-2,3-dihydrobenzofuran (0.4542 g, 2.0 mmol) and NaN_3 (0.1950 g, 3.0 mmol) as a colorless liquid in 96% crude yield (0.3633 g).

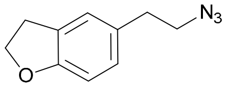

2-(azidomethyl)tetrahydrofuran (3r):[3] The crude residue was prepared according to the procedure A from tetrahydrofurfuryl bromide (0.3301 g, 2.0 mmol) and NaN_3 (0.1950 g, 3.0 mmol) as a colorless liquid in 80% crude yield (0.2034 g).

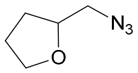

(2-azidoethyl)(phenyl)sulfane (3s):[3] The crude residue was prepared according to the procedure A from (2-bromoethyl)(phenyl)sulfane (0.4342 g, 2.0 mmol) and NaN_3 (0.1950 g, 3.0 mmol) as a colorless liquid in 91% crude yield (0.3262 g).

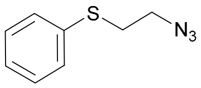

1,2-bis(azidomethyl)benzene (3u):[10] The crude residue was prepared according to the procedure A from 1,2-bis(bromomethyl)benzene (0.5280 g, 2.0 mmol) and NaN_3 (0.3900 g, 6.0 mmol) as a colorless liquid in 93% crude yield (0.3500 g).

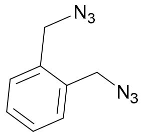

1,4-bis(azidomethyl)benzene (3v):[3] The crude residue was prepared according to the procedure A from 1,4-bis(bromomethyl)benzene (0.5280 g, 2.0 mmol) and NaN_3 (0.3900 g, 6.0 mmol) as a colorless liquid in 96% crude yield (0.3613 g).

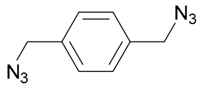

1,8-diazidooctane (3w):[5] The crude residue was prepared according to the procedure A from 1,8-dibromooctane (0.5440 g, 2.0 mmol) and NaN_3 (0.3900 g, 6.0 mmol) as a colorless liquid in 84% crude yield (0.3297 g).

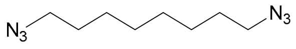

1-(azidomethyl)-3-chlorobenzene (3x):[11] The crude residue was prepared according to the procedure A from 1-(bromomethyl)-3-chlorobenzene (0.4110 g, 2.0 mmol) and NaN_3 (0.1950 g, 3.0 mmol) as a colorless liquid in 92% crude yield (0.3084 g).

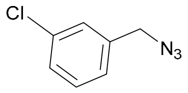

1-(azidomethyl)-4-(tert-butyl)benzene (3y):[12] The crude residue was prepared according to the procedure A from 1-(bromomethyl)-4-(*tert*-butyl)benzene (0.4543 g, 2.0 mmol) and NaN_3 (0.1950 g, 3.0 mmol) as a colorless liquid in 95% crude yield (0.3596 g).

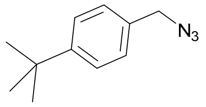

(2-azidoethyl)benzene (3z):[3] The crude residue was prepared according to the procedure A from (2-bromoethyl)benzene (0.3701 g, 2.0 mmol) and NaN_3 (0.1950 g, 3.0 mmol) as a colorless liquid in 90% crude yield (0.2649 g).

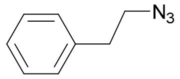

1-azido-4-chlorobutane (3ac):[13] The crude residue was prepared according to the procedure A from 1-bromo-4-chlorobutane (0.3429 g, 2.0 mmol) and NaN_3 (0.1950 g, 3.0 mmol) as a colorless liquid in 73% crude yield (0.1950 g).

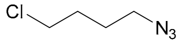

2.2 General Procedure B for the Synthesis of Azides 3t, 3ad–3af

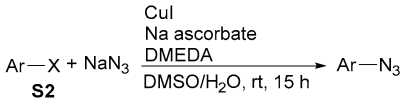

Under a nitrogen atmosphere, a Schlenk tube of 50 mL equipped with a magnetic stir bar was charged with **S2** (2.0 mmol), NaN_3 (0.2600 g, 4.0 mmol), CuI (0.0381 g, 0.2 mmol), Na ascorbate (0.0198 g, 0.1 mmol), DMEDA (0.0264 g, 0.3 mmol), DMSO (5 mL) and H_2O (5 mL) under a nitrogen atmosphere. The reaction mixture was stirred at rt for 15 h. The product was extracted with Et_2O after the mixture was quenched with 10 mL of H_2O. The organic layer was washed with brine and dried over $MgSO_4$. The solvents were evaporated after filtration through a thin cake of silica gel. The crude residue was used for the following reaction without further purification.[3]

1-azido-4-methylbenzene (3t):[3] The crude residue was prepared according to the procedure B from 1-bromo-4-methylbenzene (0.3421 g, 2.0 mmol) as a colorless liquid in 93% crude yield (0.2476 g).

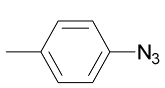

1-azido-3-methylbenzene (3ad):[3] The crude residue was prepared according to the procedure B from 1-bromo-3-methylbenzene (0.3421 g, 2.0 mmol) as a colorless liquid in 90% crude yield (0.2397 g).

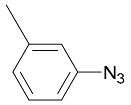

1-(2-azidophenyl)ethan-1-one (3ae):[14] The crude residue was prepared according to the procedure B from 2'-bromoacetophenone (0.3981 g, 2.0 mmol) as a colorless liquid in 96% crude yield (0.3094 g).

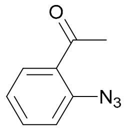

1-azido-4-(trifluoromethyl)benzene (3af):[3] The crude residue was prepared according to the procedure B from 1-bromo-4-(trifluoromethyl)benzene (0.4500 g, 2.0 mmol) as a colorless liquid in 90% crude yield (0.3368 g).

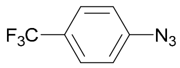

2.3 General Procedure C for the Synthesis of Azides 3aa and 3ab

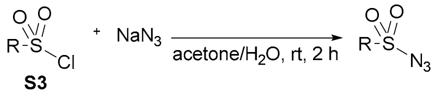

Under a nitrogen atmosphere, a Schlenk tube of 50 mL equipped with a magnetic stir bar was charged with **S3** (5.0 mmol) and acetone (5 mL). Add the water (5 mL) solution of NaN_3 (0.4876 g, 7.5 mmol) to the above Schlenk tube. The reaction mixture was stirred at rt for 2 h. The product was extracted with Et_2O after the mixture was quenched with 10 mL of H_2O. The organic layer was washed with brine and dried over $MgSO_4$. The solvents were evaporated after filtration through a thin cake of silica gel. The crude residue was used for the following reaction without further purification. [15]

benzenesulfonyl azide (3aa):[15] The crude residue was prepared according to the procedure C from benzenesulfonyl chloride (0.8830 g, 5.0 mmol) as a colorless liquid in 89% crude yield (0.8152 g).

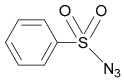

4-methylbenzenesulfonyl azide (3ab):[15] The crude residue was prepared according to the procedure C from 4-methylbenzenesulfonyl chloride (0.9532 g, 5.0 mmol) as a colorless liquid in 96% crude yield (0.9466 g).

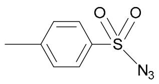

3. General Procedure D for Preparation of 4-Sulfanyl-1,2,3-triazoles (L1-L5)

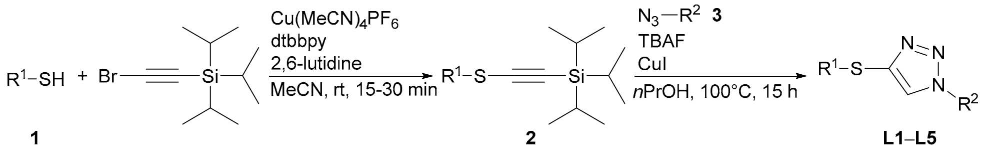

Dissolve $Cu(MeCN)_4PF_6$ (0.0373 g, 0.1 mmol) in degassed acetonitrile (9 mL) completely under nitrogen. A Schlenk tube of 100 mL equipped with a magnetic stir bar was charged with

(bromoethynyl)triisopropylsilane (0.2613 or 0.5226 g, 1.0 or 2.0 mmol), thiol **1** (1.1 or 2.2 mmol), dtbbpy (0.0713 g, 0.2 mmol, 20 mol%), 2,6-lutidine (0.23 mL, 0.2143 g, 2.0 mmol) and degassed acetonitrile (20 mL) under nitrogen. Then the above acetonitrile solution of $Cu(MeCN)_4PF_6$ was added to the Schlenk tube. The mixture was stirred at rt for 15–30 mins, and then was concentrated under vacuum to provide a crude reaction mixture. The residue was extracted with EtOAc (30 mL) and saturated NH_4Cl aqueous (20 mL) solution. The organic layer was washed with brine and dried over Na_2SO_4. The solvents were evaporated after filtration. The crude residue was subjected to a short column chromatography on silica-gel (hexanes) to extract alkynyl sulfides **2**[16], which was used for next step without further purification. Dissolve the crude product **2** in THF (10 mL) and added TBAF (1.0 M in THF, 1.0 mL, 1.0 mmol) at 0°C. The mixture was stirred at rt for 10 h, then added CuI (0.0190 g, 0.1 mmol), azide **3** (1.0 mmol) and *n*PrOH (20 mL). And the mixture was stirred at 100–110°C for 15 h. The product was extracted with EtOAc after the mixture was quenched with 20 mL of saturated NH_4Cl aqueous solution. The combined organic layer was washed with brine and dried over Na_2SO_4. The solvents were evaporated after filtration. The crude residue was subjected to column chromatography on silica-gel (petroleum ether/EtOAc, 4:1) to extract **L1−L5** in pure forms.

1-benzyl-4-(*p*-tolylthio)-1*H*-1,2,3-triazole (L1):[3] prepared from (bromoethynyl)triisopropylsilane (0.2613 g, 1.0 mmol), *p*-toluenethiol (0.1366 g, 1.1 mmol) and benzyl azide **3a** (0.1332 g, 1.0 mmol) as a white solid in 81% yield (0.2279 g).

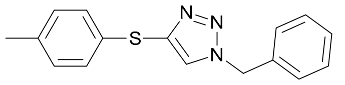

^{1}H NMR (400 MHz, $CDCl_3$) δ 7.49 (s, 1H), 7.36 (br, 3H), 7.25−7.21 (m, 4H), 7.05 (d, *J* = 8.0 Hz, 2H), 5.51 (s, 2H), 2.28 (s, 3H).

1-benzyl-4-(decylthio)-1*H*-1,2,3-triazole (L2):[3] Prepared from (bromoethynyl)triisopropylsilane (0.2613 g, 1.0 mmol), 1-decanethiol (0.2226 g, 1.1 mmol) and benzyl azide **3a** (0.1332 g, 1.0 mmol) as a white solid in 62% yield (0.2229 g).

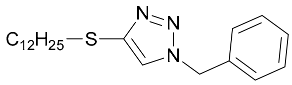

^{1}H NMR (400 MHz, $CDCl_3$) δ 7.39−7.38 (m, 4H), 7.28−7.26 (m, 2H), 5.51 (s, 2H), 2.91 (t, *J* = 7.4 Hz, 2H), 1.60 (q, *J* = 7.5 Hz, 2H), 1.38−1.35 (m, 2H), 1.24 (br, 16H), 0.89 (t, *J* = 6.7 Hz, 3H).

1-((2,2-dimethyl-1,3-dioxolan-4-yl)methyl)-4-(*p*-tolylthio)-1*H*-1,2,3-triazole (L3): prepared from (bromoethynyl)triisopropylsilane (0.2613 g, 1.0 mmol), *p*-toluenethiol (0.1366 g, 1.1 mmol), **3b** (0.1572 g, 1.0 mmol) and DMEDA (0.02 mL, 0.0176 g, 0.2 mmol) as a white solid in 65% yield (0.1985 g).

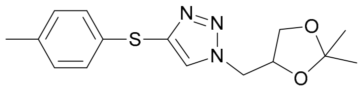

Melting point (M.P.): 66°C−68°C; ^{1}H NMR (400 MHz, $CDCl_3$) δ 7.76 (s, 1H), 7.24 (d, *J* = 8.4 Hz,

2H), 7.07 (d, J = 7.1 Hz, 2H), 4.55 (d, J = 11.8 Hz, 1H), 4.48–4.40 (m, 2H), 4.13–4.09 (m, 1H), 3.72 (dd, J = 8.2, 5.3 Hz, 1H), 2.29 (s, 3H), 1.32 (d, J = 4.0 Hz, 6H). ^{13}C NMR (101 MHz, $CDCl_3$) δ 138.88, 136.75, 131.41, 129.65, 129.42, 128.37, 109.97, 73.58, 65.99, 52.21, 26.38, 24.93, 20.78. HRMS-ESI (m/z) [M+H$^+$] Calcd for $C_{15}H_{20}O_2N_3S$ 306.1276; Found, 306.1277.

1-hexyl-4-(*p*-tolylthio)-1*H*-1,2,3-triazole (L4):[3] prepared from (bromoethynyl)triisopropylsilane (0.2613 g, 1.0 mmol), *p*-toluenethiol (0.1366 g, 1.1 mmol), **3c** (0.1272 g, 1.0 mmol) and DMEDA (0.02 mL, 0.0176 g, 0.2 mmol) as a white solid in 70% yield (0.1928 g).

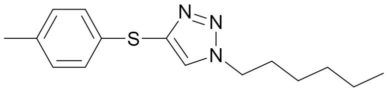

Melting point (M.P.): 98 °C−100 °C; 1H NMR (400 MHz, $CDCl_3$) δ 7.58 (s, 1H), 7.23 (d, J = 8.2 Hz, 2H), 7.07 (d, J = 8.1 Hz, 2H), 4.34 (t, J = 7.3 Hz, 2H), 2.30 (s, 3H), 1.93–1.86 (m, 2H), 1.69 (t, J = 4.3 Hz, 2H), 1.33–1.29 (m, 4H), 0.88 (t, J = 6.0 Hz, 3H).

1,3-bis((4-(*p*-tolylthio)-1*H*-1,2,3-triazol-1-yl)methyl)benzene (L5): prepared from (bromoethynyl)triisopropylsilane (0.5226 g, 2.0 mmol), *p*-toluenethiol (0.2732 g, 2.2 mmol) and **3d** (0.1882 g, 1.0 mmol) and purified by recrystallization as a white solid in 76% yield (0.3683 g).

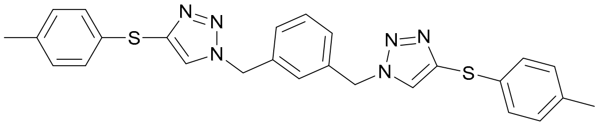

Melting point (M.P.): 111°C−113°C; ^1H NMR (400 MHz, $CDCl_3$) δ 7.50 (s, 2H), 7.38 (t, J = 7.7 Hz, 1H), 7.23 (d, J = 7.3 Hz, 6H), 7.17 (s, 1H), 7.07 (d, J = 8.0 Hz, 4H), 5.49 (s, 4H), 2.29 (s, 6H). ^{13}C NMR (101 MHz, $CDCl_3$) δ 140.33, 137.24, 135.41, 131.21, 130.11, 129.99, 129.90, 128.42, 127.50, 126.65, 53.94, 21.00. HRMS-ESI (m/z) [M+H$^+$] Calcd for $C_{26}H_{25}N_6S_2$ 485.1582; Found, 485.1585.

4. Optimization the reaction conditions of CuAAC between 4a and 3a

The solvent-free CuAAC of ethynes (**4**) and azides (**3**) using **L1**–**L5** as ligands was then performed (Table S1). When $CuSO_4$/**L1** was used, the desired **5a** was formed in 16% yield from phenylacetylene (**4a**) and (azidomethyl)benzene (**3a**) (entry 1). Among **L1**–**L5**, **L1** gave the highest yield (entry 1–5). Compared with $CuSO_4$, CuI, CuBr, CuCl, CuI_2, $CuBr_2$ and $CuCl_2$, $Cu(OAc)_2$ exhibited better performance (entry 1, 6–12). Combining CuBr and $Cu(OAc)_2$ catalysts enhanced the yield of **5a** to 98% yield (entry 13). The catalyst loading could be reduced to 0.1 mol% (CuBr/$Cu(OAc)_2$/**L1**) with prolonging the time to 0.5 h, affording **5a** in 99% yield (entry 15). While the yield of **5a** was only 28% without **L1** even when the di-copper catalytic system $Cu(OAc)_2$/CuBr was used (entry 16). When 1,4-substituted 1,2,3-triazole without sulfanyl group (**5a**) was applied, the yield of **5a** was 37% (entry 17). In the mono-copper system $Cu(OAc)_2$/**L1** (0.1 mol%), **5a** was provided in 78% yield after stirring for 2.5 h (entry 18). Prolonging the reaction time to 5.5 h enhanced the yield of **5a** to 99% (entry 19), while **5a** was formed in 48% yield without any ligand (entry 20). Notably, **L1** displayed better performance

compared to **5a**, Ph_2S, PhSMe, Bu_2S and difurfurylsulfide (entry 19 *vs* 21–25), demonstrating that the coordination of both sulfanyl and 1,2,3-triazole moieties to copper was critical to enhance the catalytic performance of **L1**. In the reported CuAAC proceeding in CH_2Cl_2/CH_3OH[14], **L1** also exhibited higher catalytic activity than **Hm-TBTA** (entry 28 *vs* 27). The optimized conditions were obtained as following: a mixture of **4a** (2.0 mmol), **3a** (2.0 mmol), $Cu(OAc)_2$ (0.002 mmol) and **L1** (0.002 mmol), at rt under air for 5.5 h, producing **5a** in 99% yield (0.4659 g) (entry 17). In a gram-scale synthesis, 1.0213 g (10.0 mmol) of **4a** produced 2.3294 g (9.9 mmol) of **5a** in 99% yield (entry 26).

Table S1. Optimization the reaction conditions of CuAAC between **4a** and **3a**.[a]

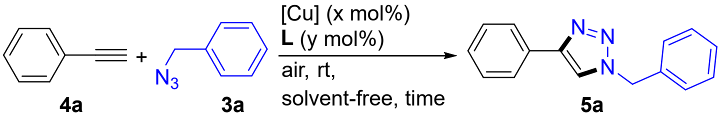

4a **3a** **5a**

Entry	L	[Cu]	x	y	time (h)	yield (%)
1	**L1**	$CuSO_4$	1	0.5	0.2	16
2	**L2**	$CuSO_4$	1	0.5	0.2	13
3	**L3**	$CuSO_4$	1	0.5	0.2	14
4	**L4**	$CuSO_4$	1	0.5	0.2	10
5	**L5**	$CuSO_4$	1	0.5	0.2	8
6	**L1**	CuI	1	0.5	0.2	17
7	**L1**	CuBr	1	0.5	0.2	26
8	**L1**	CuCl	1	0.5	0.2	22
9	**L1**	CuI_2	1	0.5	0.2	20
10	**L1**	$CuBr_2$	1	0.5	0.2	<5
11	**L1**	$CuCl_2$	1	0.5	0.2	12
12	**L1**	$Cu(OAc)_2$	1	0.5	0.2	50
13[b]	**L1**	$CuBr/Cu(OAc)_2$	1	0.5	0.2	98
14[c]	**L1**	$CuBr/Cu(OAc)_2$	0.5	0.25	0.2	85
15[d]	**L1**	$CuBr/Cu(OAc)_2$	0.2	0.1	0.5	99
16[d]	-	$CuBr/Cu(OAc)_2$	0.2		0.5	28
17[d]	**5a**	$CuBr/Cu(OAc)_2$	0.2	0.1	0.5	37
18	**L1**	$Cu(OAc)_2$	0.1	0.1	2.5	78
19	**L1**	$Cu(OAc)_2$	0.1	0.1	5.5	99
20	-	$Cu(OAc)_2$	0.1		5.5	48
21	**5a**	$Cu(OAc)_2$	0.1	0.1	5.5	50
22	**Ph_2S**	$Cu(OAc)_2$	0.1	0.1	5.5	52
23	**PhSMe**	$Cu(OAc)_2$	0.1	0.1	5.5	56
24	**Bu_2S**	$Cu(OAc)_2$	0.1	0.1	5.5	47
25	**Difurfurylsulfide**	$Cu(OAc)_2$	0.1	0.1	5.5	61
26[e]	**L1**	$Cu(OAc)_2$	0.1	0.1	5.5	99
27[f]	**Hm-TBTA**	$[Cu(CH_3CN)_4]PF_6$	1	1	24 h	83[14]
28[f]	**L1**	$[Cu(CH_3CN)_4]PF_6$	1	1	24 h	86

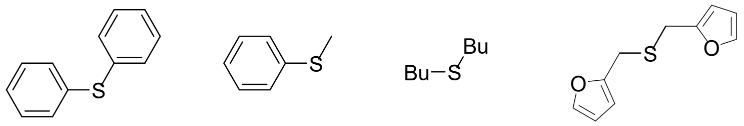

Diphenyl sulfide **Thioanisole** **Butyl sulfide** **Difurfurylsulfide**

[a] **4a** (2.0 mmol), **3a** (2.0 mmol), isolated yields. [b] CuBr (0.5 mol%), $Cu(OAc)_2$ (0.5 mol%). [c] CuBr (0.25 mol%), $Cu(OAc)_2$ (0.25 mol%). [d] CuBr (0.1 mol%), $Cu(OAc)_2$ (0.1 mol%). [e] **4a** (10.0 mmol), **3a** (10.0 mmol). [f] **4a** (0.2 mmol), **3a** (0.2 mmol), glutathione (0.04 mmol), CH_2Cl_2 (2 mL), CH_3OH (0.2 mL).

5. General Procedure E for Synthesis of 1,2,3-Triazoles (5a–5ay, 6a–6n, 7a–7h and 8a–8e)

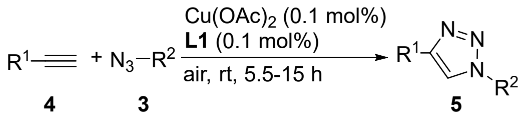

A mixture of $Cu(OAc)_2$ (0.0004 g, 0.002 mmol), **L1** (0.0006 g, 0.002 mmol), ethyne **4** (2.0 mmol) and azide **3** (2.0 mmol) was stirred under air at rt for 5.5–15 h. Then washed the reaction mixture with water twice followed by cold petroleum ether (or petroleum ether/ethyl acetate (10/1)) for three times or subjection the reaction mixture to column chromatography on silica gel to produce the desired **5a**–**5ay** (purified by washing with water and cold petroleum ether), **6a–6n** (purified by column chromatography), **7a–7h** (purified by column chromatography), and **8a–8e** (purified by washing with water and petroleum ether/ethyl acetate (10/1)) in pure forms.

1-benzyl-4-phenyl-1*H*-1,2,3-triazole (5a):[17] prepared from **4a** (0.2042 g, 2.0 mmol) and **3a** (0.2663 g, 2.0 mmol) by stirring for 5.5 h as a white solid in 99% yield (0.4659 g).

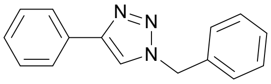

^{1}H NMR (400 MHz, $CDCl_3$) δ 7.80 (d, J = 7.0 Hz, 2H), 7.66 (s, 1H), 7.42–7.36 (m, 5H), 7.33–7.29 (m, 3H), 5.58 (s, 2H).

Gram-scale synthesis of 5a: A mixture of $Cu(OAc)_2$ (0.0018 g, 0.01 mmol), **L1** (0.0028 g, 0.01 mmol), **4a** (1.0213 g, 10.0 mmol) and **3a** (1.3315 g, 10.0 mmol) was stirred under air at rt for 5.5 h. Then washed the reaction mixture with water twice followed by cold hexane for three times to produce the desired **5a** as a white solid in 99% yield (2.3294 g).

1-benzyl-4-(3-methoxyphenyl)-1*H*-1,2,3-triazole (5b)[18]: prepared from **4b** (0.2643 g, 2.0 mmol) and **3a** (0.2663 g, 2.0 mmol) by stirring for 5.5 h as a white solid in 99% yield (0.5253 g).

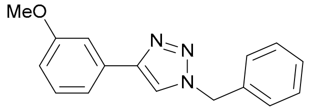

^{1}H NMR (400 MHz, $CDCl_3$) δ 7.65 (s, 1H), 7.43–7.36 (m, 4H), 7.31–7.27 (m, 4H), 6.89–6.84 (m, 1H), 5.57 (s, 2H), 3.85 (s, 3H).

1-benzyl-4-(4-ethylphenyl)-1*H*-1,2,3-triazole (5c)[19]: prepared from **4c** (0.2604 g, 2.0 mmol) and **3a** (0.2663 g, 2.0 mmol) by stirring for 5.5 h as a white solid in 97% yield (0.5109 g).

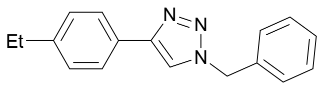

^{1}H NMR (400 MHz, $CDCl_3$) δ 7.71 (d, J = 7.9 Hz, 2H), 7.63 (s, 1H), 7.41–7.35 (m, 3H), 7.30 (d, J = 8.0 Hz, 2H), 7.23 (d, J = 8.0 Hz, 2H), 5.56 (s, 2H), 2.66 (q, J = 8.0 Hz, 2H), 1.24 (t, J = 7.8 Hz, 3H).

1-benzyl-4-(4-ethylphenyl)-1*H*-1,2,3-triazole (5d)[19]: prepared from **4d** (0.2884 g, 2.0 mmol) and **3a** (0.2663 g, 2.0 mmol) by stirring for 5.5 h as a white solid in 95% yield (0.5270 g).

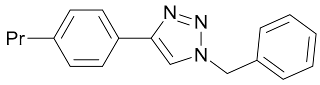

^{1}H NMR (400 MHz, $CDCl_3$) δ 7.71 (d, J = 8.2 Hz, 2H), 7.63 (s, 1H), 7.40–7.35 (m, 3H), 7.30–7.28 (m, 2H), 7.21 (d, J = 8.3 Hz, 2H), 5.55 (s, 2H), 2.59 (t, J = 8.0 Hz, 2H), 1.69–1.60 (m, 2H), 0.94 (t, J = 7.3 Hz, 3H).

1-benzyl-4-(4-ethylphenyl)-1*H*-1,2,3-triazole (5e)[20]: prepared from **4e** (0.3244 g, 2.0 mmol) and **3a** (0.2663 g, 2.0 mmol) by stirring for 8 h as a white solid in 90% yield (0.5316 g).

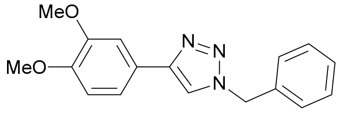

^{1}H NMR (400 MHz, $CDCl_3$) δ 7.61 (s, 1H), 7.46 (d, J = 2.0 Hz, 1H), 7.39–7.36 (m, 3H), 7.32–7.30 (m, 2H), 7.24 (dd, J = 8.3, 2.0 Hz, 1H), 6.87 (d, J = 8.3 Hz, 1H), 5.56 (s, 2H), 3.94 (s, 3H), 3.89 (s, 3H).

methyl 4-(1-benzyl-1*H*-1,2,3-triazol-4-yl)benzoate (5f)[21]: prepared from **4f** (0.3203 g, 2.0 mmol) and **3a** (0.2663 g, 2.0 mmol) by stirring for 6.5 h as a white solid in 91% yield (0.5338 g).

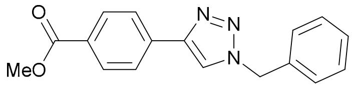

^{1}H NMR (400 MHz, $CDCl_3$) δ 8.07 (d, J = 8.4 Hz, 2H), 7.87 (d, J = 8.4 Hz, 2H), 7.75 (s, 1H), 7.43–7.38 (m, 3H), 7.33–7.32 (m, 2H), 5.59 (s, 2H), 3.92 (s, 3H).

1-benzyl-4-(3-nitrophenyl)-1*H*-1,2,3-triazole (5g)[22]: prepared from **4g** (0.2943 g, 2.0 mmol) and **3a** (0.2663 g, 2.0 mmol) by stirring for 8 h as a white solid in 93% yield (0.5213 g).

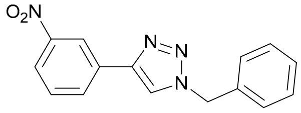

^{1}H NMR (400 MHz, $CDCl_3$) δ 8.56 (t, J = 2.0 Hz, 1H), 8.22 (dt, J = 7.7, 1.4 Hz, 1H), 8.17–8.14 (m, 1H), 7.81 (s, 1H), 7.59 (t, J = 8.0 Hz, 1H), 7.44–7.33 (m, 5H), 5.61 (s, 2H).

1-benzyl-4-(3-chlorophenyl)-1*H*-1,2,3-triazole (5h)[18]: prepared from **4h** (0.2732 g, 2.0 mmol) and **3a** (0.2663 g, 2.0 mmol) by stirring for 6 h as a white solid in 99% yield (0.5341 g).

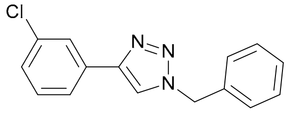

^{1}H NMR (400 MHz, $CDCl_3$) δ 7.78 (s, 1H), 7.69 (d, J = 7.5 Hz, 1H), 7.66 (s, 1H), 7.40–7.37 (m, 3H), 7.35–7.27 (m, 4H), 5.58 (s, 2H).

1-benzyl-4-(2-bromophenyl)-1*H*-1,2,3-triazole (5i)[23]: prepared from **4i** (0.3621 g, 2.0 mmol) and **3a** (0.2663 g, 2.0 mmol) by stirring for 6 h as a white solid in 94% yield (0.5907 g).

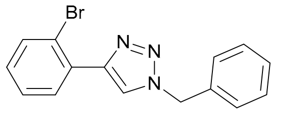

^{1}H NMR (400 MHz, $CDCl_3$) δ 8.18 (s, 1H), 8.12 (d, J = 7.8 Hz, 1H), 7.62 (d, J = 8.1 Hz, 1H), 7.49–7.32 (m, 6H), 7.18 (t, J = 7.7 Hz, 1H), 5.60 (s, 2H).

1-benzyl-4-(3-fluorophenyl)-1*H*-1,2,3-triazole (5j)[20]: prepared from **4j** (0.2403 g, 2.0 mmol) and **3a** (0.2663 g, 2.0 mmol) by stirring for 6 h as a white solid in 95% yield (0.4812 g).

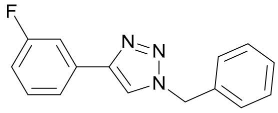

^{1}H NMR (400 MHz, $CDCl_3$) δ 7.71 (s, 1H), 7.55–7.50 (m, 2H), 7.37–7.27 (m, 6H), 7.00–6.95 (m, 1H), 5.54 (s, 2H).

1-benzyl-4-(4-(trifluoromethyl)phenyl)-1*H*-1,2,3-triazole (5k)[23]: prepared from **4k** (0.3403 g, 2.0 mmol) and **3a** (0.2663 g, 2.0 mmol) by stirring for 6 h as a white solid in 93% yield (0.5641 g).

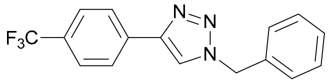

^{1}H NMR (400 MHz, $CDCl_3$) δ 7.91 (d, J = 7.8 Hz, 2H), 7.73 (s, 1H), 7.65 (d, J = 8.2 Hz, 2H), 7.44–7.39 (m, 3H), 7.34–7.32 (m, 2H), 5.60 (s, 2H).

1-benzyl-4-(thiophen-2-yl)-1*H*-1,2,3-triazole (5l)[24]: prepared from **4l** (0.2163 g, 2.0 mmol) and **3a** (0.2663 g, 2.0 mmol) by stirring for 8 h as a white solid in 89% yield (0.4295 g).

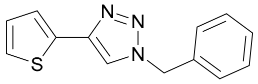

^{1}H NMR (400 MHz, $CDCl_3$) δ 7.58 (s, 1H), 7.41–7.36 (m, 3H), 7.33 (dd, J = 3.6, 1.2 Hz, 1H), 7.31–7.29 (dd, J = 7.4, 2.2 Hz, 2H), 7.27 (dd, J = 5.1, 1.2 Hz, 1H), 7.04 (dd, J = 5.1, 3.6 Hz, 1H), 5.54 (s, 2H).

2-(1-benzyl-1*H*-1,2,3-triazol-4-yl)pyridine (5m)[24]: prepared from **4m** (0.2062 g, 2.0 mmol) and **3a** (0.2663 g, 2.0 mmol) by stirring for 5.5 h as a white solid in 91% yield (0.4300 g).

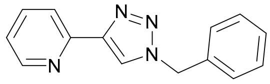

^{1}H NMR (400 MHz, $CDCl_3$) δ 8.54 (d, J = 3.9 Hz, 1H), 8.18 (d, J = 7.9 Hz, 1H), 8.06 (s, 1H), 7.76 (t, J = 6.0 Hz, 1H), 7.40–7.32 (m, 5H), 7.21 (dd, J = 7.6, 4.8 Hz, 1H), 5.58 (s, 2H).

2-(1-benzyl-1*H*-1,2,3-triazol-4-yl)pyrazine (5n)[25]: prepared from **4n** (0.2082 g, 2.0 mmol) and **3a** (0.2663 g, 2.0 mmol) by stirring for 5.5 h as a white solid in 92% yield (0.5028 g).

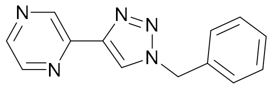

^{1}H NMR (400 MHz, $CDCl_3$) δ 9.42 (s, 1H), 8.49 (t, J = 2.8 Hz, 2H), 8.08 (s, 1H), 7.42–7.38 (m, 3H), 7.35–7.33 (m, 2H), 5.61 (s, 2H).

1-benzyl-4-hexyl-1*H*-1,2,3-triazole (5o)[18]: prepared from **4o** (0.2204 g, 2.0 mmol) and **3a** (0.2663 g, 2.0 mmol) by stirring for 8 h as a white solid in 85% yield (0.4137 g).

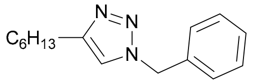

^{1}H NMR (400 MHz, $CDCl_3$) δ 7.40–7.34 (m, 3H), 7.26–7.24 (m, 2H), 7.18 (s, 1H), 5.49 (s, 2H), 2.68 (t, J = 4 Hz, 2H), 1.65–1.59 (m, 2H), 1.31–1.24 (m, 6H), 0.86 (t, J = 6 Hz, 3H).

1-benzyl-4-decyl-1*H*-1,2,3-triazole (5p)[26]: prepared from **4p** (0.3326 g, 2.0 mmol) and **3a** (0.2663 g, 2.0 mmol) by stirring for 8 h as a white solid in 87% yield (0.5211 g).

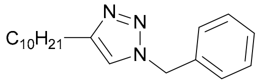

^{1}H NMR (400 MHz, $CDCl_3$) δ 7.33–7.28 (m, 3H), 7.21–7.18 (m, 3H), 5.43 (s, 2H), 2.63 (t, J = 7.7 Hz, 2H), 1.63–1.56 (m, 2H), 1.25–1.21 (m, 14H), 0.83 (t, J = 6.8 Hz, 3H).

1-benzyl-4-(phenoxymethyl)-1*H*-1,2,3-triazole (5q)[20]: prepared from **4q** (0.2643 g, 2.0 mmol) and **3a** (0.2663 g, 2.0 mmol) by stirring for 8 h as a white solid in 92% yield (0.4882 g).

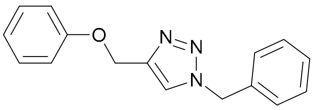

^{1}H NMR (400 MHz, $CDCl_3$) δ 7.54 (s, 1H), 7.40–7.34 (t, J = 2.3 Hz, 3H), 7.30–7.25 (m, 4H), 6.98–6.95 (m, 3H), 5.51 (s, 2H), 5.18 (s, 2H).

1-benzyl-4-(cyclohex-1-en-1-yl)-1*H*-1,2,3-triazole (5r)[18]: prepared from **4r** (0.2123 g, 2.0 mmol) and **3a** (0.2663 g, 2.0 mmol) by stirring for 8 h as a white solid in 88% yield (0.4212 g).

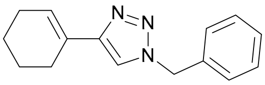

^{1}H NMR (400 MHz, $CDCl_3$) δ 7.38–7.33 (m, 3H), 7.30 (s, 1H), 7.26–7.24 (m, 2H), 6.48 (s, 1H), 5.49 (s, 2H), 2.36–2.33 (m, 2H), 2.18–2.16 (m, 2H), 1.76–1.71 (m, 2H), 1.67–1.61 (m, 2H).

ethyl 1-benzyl-1*H*-1,2,3-triazole-4-carboxylate (5s)[27]: prepared from **4s** (0.1962 g, 2.0 mmol) and **3a** (0.2663 g, 2.0 mmol) by stirring for 8 h as a white solid in 86% yield (0.3978 g).

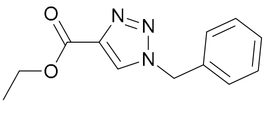

^{1}H NMR (400 MHz, $CDCl_3$) δ 7.98 (s, 1H), 7.40–7.39 (m, 3H), 7.31–7.28 (m, 2H), 5.58 (s, 2H), 4.40 (q, J = 7.1 Hz, 2H), 1.38 (t, J = 7.1 Hz, 3H).

1-(4-methylbenzyl)-4-phenyl-1*H*-1,2,3-triazole (5t)[18]: prepared from **4a** (0.2042 g, 2.0 mmol) and **3e** (0.2944 g, 2.0 mmol) by stirring for 5.5 h as a white solid in 99% yield (0.4936 g).

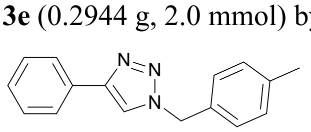

^{1}H NMR (400 MHz, $CDCl_3$) δ 7.79 (d, J = 7.2 Hz, 2H), 7.64 (s, 1H), 7.39 (t, J = 7.6 Hz, 2H), 7.30 (t, J = 7.4 Hz, 1H), 7.22–7.17 (m, 4H), 5.51 (s, 2H), 2.35 (s, 3H).

1-(2-methylbenzyl)-4-phenyl-1H-1,2,3-triazole (5u)[28]: prepared from **4a** (0.2042 g, 2.0 mmol) and **3f** (0.2944 g, 2.0 mmol) by stirring for 7 h as a white solid in 94% yield (0.4687 g).

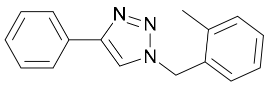

^{1}H NMR (400 MHz, $CDCl_3$) δ 7.79 (d, J = 7.4 Hz, 2H), 7.56 (s, 1H), 7.38 (t, J = 7.6 Hz, 2H), 7.30 (t, J = 7.1 Hz, 2H), 7.25–7.22 (m, 2H), 7.18 (d, J = 7.2 Hz, 1H), 5.55 (s, 2H), 2.30 (s, 3H).

4-phenyl-1-(4-(trifluoromethyl)benzyl)-1*H*-1,2,3-triazole (5v)[29]: prepared from **4a** (0.2042 g, 2.0 mmol) and **3g** (0.4023 g, 2.0 mmol) by stirring for 7 h as a white solid in 90% yield (0.5459 g).

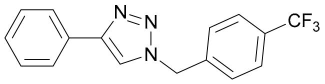

^{1}H NMR (400 MHz, $CDCl_3$) δ 7.80 (d, J = 7.5 Hz, 2H), 7.72 (s, 1H), 7.63 (d, J = 8.1 Hz, 2H), 7.40 (t, J = 7.8 Hz, 4H), 7.33 (t, J = 7.6 Hz, 1H), 5.63 (s, 2H).

4-(thiophen-2-yl)-1-(4-(trifluoromethyl)benzyl)-1*H*-1,2,3-triazole (5w)[30]: prepared from **4l** (0.2163 g, 2.0 mmol) and **3g** (0.4023 g, 2.0 mmol) by stirring for 7 h as a white solid in 87% yield (0.5382 g).

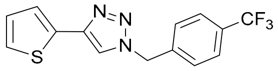

^{1}H NMR (400 MHz, $CDCl_3$) δ 7.65 (d, J = 8.1 Hz, 2H), 7.62 (s, 1H), 7.41 (d, J = 8.1 Hz, 2H), 7.36 (dd, J = 3.6, 1.2 Hz, 1H), 7.30 (dd, J = 5.0, 1.2 Hz, 1H), 7.07 (dd, J = 5.1, 3.6 Hz, 1H), 5.63 (s, 2H).

1-(3-bromobenzyl)-4-phenyl-1*H*-1,2,3-triazole (5x)[31]: prepared from **4a** (0.2042 g, 2.0 mmol) and **3h** (0.4241 g, 2.0 mmol) by stirring for 7 h as a white solid in 91% yield (0.5718 g).

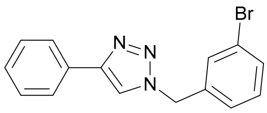

^{1}H NMR (400 MHz, $CDCl_3$) δ 7.82–7.80 (m, 2H), 7.70 (s, 1H), 7.49 (dt, J = 7.4, 1.8 Hz, 1H), 7.46 (s, 1H), 7.41 (t, J = 7.5 Hz, 2H), 7.33 (t, J = 7.4 Hz, 1H), 7.26–7.21 (m, 2H), 5.54 (s, 2H).

1-(3-bromobenzyl)-4-(thiophen-2-yl)-1*H*-1,2,3-triazole (5y): prepared from **4l** (0.2163 g, 2.0 mmol) and **3h** (0.4241 g, 2.0 mmol) by stirring for 7 h as a white solid in 92% yield (0.5892 g).

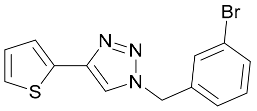

Melting point (M.P.): 116°C−118°C; ^{1}H NMR (400 MHz, $CDCl_3$) δ 7.61 (s, 1H), 7.50 (d, J = 7.5 Hz, 1H), 7.46 (s, 1H), 7.36 (t, J = 3.4 Hz, 1H), 7.30–7.28 (m, 1H), 7.25–7.21 (m, 2H), 7.08–7.05 (m, 1H), 5.53 (s, 2H). ^{13}C NMR (101 MHz, $CDCl_3$) δ 143.51, 136.65, 132.62, 132.01, 130.98, 130.73, 127.61,

126.55, 125.19, 124.31, 123.13, 118.98, 53.46. HRMS-ESI (m/z) [M+H$^+$] Calcd for $C_{13}H_{11}BrN_3S$ 319.9857; Found, 319.9859.

1-(2-chlorobenzyl)-4-phenyl-1*H*-1,2,3-triazole (5z)[32]: prepared from **4a** (0.2042 g, 2.0 mmol) and **3i** (0.3353 g, 2.0 mmol) by stirring for 7 h as a white solid in 95% yield (0.5125 g).

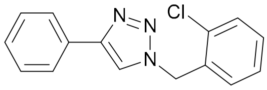

^{1}H NMR (400 MHz, $CDCl_3$) δ 7.82 (d, J = 7.1 Hz, 2H), 7.77 (s, 1H), 7.46–7.39 (m, 3H), 7.34–7.29 (m, 2H), 7.26 (t, J = 4.4 Hz, 1H), 7.22 (d, J = 9.5 Hz, 1H), 5.71 (s, 2H).

1-(naphthalen-1-ylmethyl)-4-phenyl-1H-1,2,3-triazole (5aa)[32]: prepared from **4a** (0.2042 g, 2.0 mmol) and **3j** (0.3664 g, 2.0 mmol) by stirring for 7 h as a white solid in 89% yield (0.5079 g).

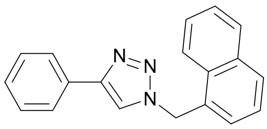

^{1}H NMR (400 MHz, $CDCl_3$) δ 8.02–7.99 (m, 1H), 7.93–7.88 (m, 2H), 7.73 (d, J = 7.0 Hz, 2H), 7.55–7.46 (m, 5H), 7.34 (t, J = 7.5 Hz, 2H), 7.28–7.25 (m, 1H), 6.01 (s, 2H).

8-((4-phenyl-1*H*-1,2,3-triazol-1-yl)methyl)quinoline (5ab): prepared from **4a** (0.2042 g, 2.0 mmol) and **3k** (0.3684 g, 2.0 mmol) by stirring for 7 h as a white solid in 86% yield (0.4925 g).

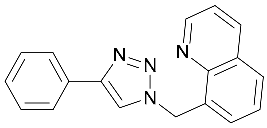

Melting point (M.P.): 120°C−122°C; ^{1}H NMR (400 MHz, $CDCl_3$) δ 9.00 (dd, J = 4.3, 1.8 Hz, 1H), 8.20 (d, J = 8.4 Hz, 1H), 7.99 (s, 1H), 7.83 (d, J = 8.2 Hz, 1H), 7.79–7.77 (m, 2H), 7.67 (d, J = 8.6 Hz, 1H), 7.53–7.47 (m, 2H), 7.37 (t, J = 7.7 Hz, 2H), 7.28 (d, J = 7.3 Hz, 1H), 6.28 (s, 2H). ^{13}C NMR (101 MHz, $CDCl_3$) δ 150.24, 147.74, 145.86, 136.49, 133.38, 130.81, 129.88, 128.93, 128.75, 128.43, 127.97, 126.52, 125.69, 121.69, 120.71, 49.85. HRMS-ESI (m/z) [M+H$^+$] Calcd for $C_{18}H_{15}N_4$ 287.1297; Found, 287.1299.

1-benzhydryl-4-phenyl-1*H*-1,2,3-triazole (5ac)[26]: prepared from **4a** (0.2042 g, 2.0 mmol) and **3l** (0.4185 g, 2.0 mmol) by stirring for 7 h as a white solid in 84% yield (0.5231 g).

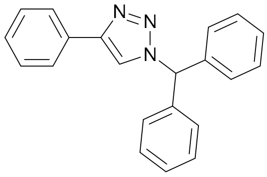

^{1}H NMR (400 MHz, $CDCl_3$) δ 7.81 (d, J = 7.8 Hz, 2H), 7.62 (s, 1H), 7.42–7.37 (m, 8H), 7.33–7.29 (m, 1H), 7.19–7.16 (m, 5H).

4-phenyl-1-(3-phenylpropyl)-1H-1,2,3-triazole (5ad)[33]: prepared from **4a** (0.2042 g, 2.0 mmol) and **3m** (0.3224 g, 2.0 mmol) by stirring for 15 h as a white solid in 94% yield (0.4951 g).

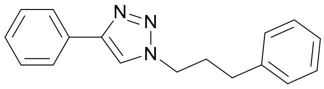

^{1}H NMR (400 MHz, CDCl$_3$) δ 7.84 (d, J = 8.0 Hz, 2H), 7.73 (s, 1H), 7.43 (d, J = 8.0 Hz, 2H), 7.35–7.29 (m, 3H), 7.23 (d, J = 7.9 Hz, 1H), 7.19 (d, J = 7.6 Hz, 2H), 4.38 (t, J = 8.0 Hz, 2H), 2.68 (t, J = 7.9 Hz, 2H), 2.32–2.25 (m, 2H).

1-cinnamyl-4-phenyl-1H-1,2,3-triazole (5ae)[28]: prepared from **4a** (0.2042 g, 2.0 mmol) and **3n** (0.3184 g, 2.0 mmol) by stirring for 15 h as a white solid in 93% yield (0.4861 g).

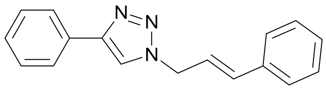

^{1}H NMR (400 MHz, CDCl$_3$) δ 7.84–7.81 (m, 3H), 7.43–7.40 (m, 4H), 7.36–7.29 (m, 4H), 6.71 (d, J = 15.8 Hz, 1H), 6.42–6.35 (m, 1H), 5.18 (d, J = 6.6 Hz, 2H).

1-hexyl-4-phenyl-1*H*-1,2,3-triazole (5af)[33]: prepared from **4a** (0.2042 g, 2.0 mmol) and **3c** (0.2544 g, 2.0 mmol) by stirring for 15 h as a white solid in 90% yield (0.4128 g).

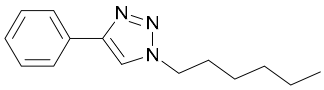

^{1}H NMR (400 MHz, CDCl$_3$) δ 7.82 (d, J = 8.1 Hz, 2H), 7.74 (s, 1H), 7.41 (t, J = 7.6 Hz, 2H), 7.31 (t, J = 7.3 Hz, 1H), 4.36 (t, J = 7.2 Hz, 2H), 1.95–1.88 (m, 2H), 1.31 (s, 6H), 0.87 (t, J = 7.2 Hz, 3H).

1-(pentan-2-yl)-4-phenyl-1H-1,2,3-triazole (5ag): prepared from **4a** (0.2042 g, 2.0 mmol) and **3o** (0.2263 g, 2.0 mmol) by stirring for 15 h as a white solid in 89% yield (0.3832 g).

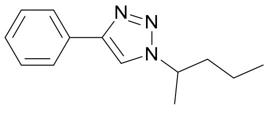

Melting point (M.P.): 58°C−61°C; ^{1}H NMR (400 MHz, CDCl$_3$) δ 7.84 (d, J = 7.3 Hz, 2H), 7.73 (s, 1H), 7.42 (t, J = 7.6 Hz, 2H), 7.32 (t, J = 7.4 Hz, 1H), 4.78–4.69 (m, 1H), 2.00–1.90 (m, 1H), 1.87–1.78 (m, 1H), 1.60 (d, J = 6.8 Hz, 3H), 1.37–1.29 (m, 1H), 1.26–1.20 (m, 1H), 0.93 (t, J = 7.3 Hz, 3H). ^{13}C NMR (101 MHz, CDCl$_3$) δ 147.5, 130.9, 128.8, 128.0, 125.6, 117.2, 57.2, 39.3, 21.4, 19.2, 13.6. HRMS-ESI (m/z) [M+H$^+$] Calcd for $C_{13}H_{18}N_3$ 216.1501; Found, 216.1502.

ethyl 2-(4-phenyl-1H-1,2,3-triazol-1-yl)acetate (5ah)[17]: prepared from **4a** (0.2042 g, 2.0 mmol) and **3p** (0.2582 g, 2.0 mmol) by stirring for 15 h as a white solid in 86% yield (0.3978 g).

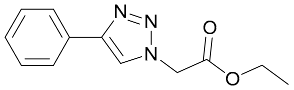

^{1}H NMR (400 MHz, CDCl$_3$) δ 7.92 (s, 1H), 7.86–7.84 (m, 2H), 7.43 (t, J = 7.4 Hz, 2H), 7.34 (t, J = 7.4 Hz, 1H), 5.20 (s, 2H), 4.28 (q, J = 7.1 Hz, 2H), 1.31 (t, J = 7.1 Hz, 3H).

1-(2-(2,3-dihydrobenzofuran-5-yl)ethyl)-4-phenyl-1H-1,2,3-triazole (5ai): prepared from **4a** (0.2042 g, 2.0 mmol) and **3q** (0.3784 g, 2.0 mmol) by stirring for 15 h as a white solid in 91% yield (0.5303 g).

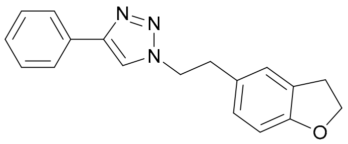

Melting point (M.P.): 106°C−108°C; ^{1}H NMR (400 MHz, $CDCl_3$) δ 7.77 (d, J = 7.8 Hz, 2H), 7.49 (s, 1H), 7.41 (t, J = 8.0 Hz, 2H), 7.32 (t, J = 7.7 Hz, 1H), 6.93 (s, 1H), 6.87 (d, J = 7.6 Hz, 1H), 6.71 (d, J = 7.8 Hz, 1H), 4.60–4.52 (m, 4H), 3.19–3.12 (m, 4H). ^{13}C NMR (101 MHz, $CDCl_3$) δ 159.2, 147.4, 130.7, 128.9, 128.8, 128.2, 128.0, 127.6, 125.7, 125.3, 119.9, 109.4, 71.2, 52.2, 36.2, 29.6. HRMS-ESI (m/z) [M+H$^+$] Calcd for $C_{18}H_{18}N_3O$ 292.1450; Found, 292.1453.

4-phenyl-1-((tetrahydrofuran-2-yl)methyl)-1H-1,2,3-triazole (5aj)[34]: prepared from **4a** (0.2042 g, 2.0 mmol) and **3r** (0.2119 g, 2.0 mmol) by stirring for 15 h as a white solid in 85% yield (0.3898 g).

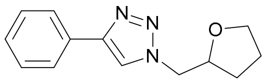

^{1}H NMR (400 MHz, $CDCl_3$) δ 7.93 (s, 1H), 7.83 (d, J = 6.9 Hz, 2H), 7.40 (t, J = 7.6 Hz, 2H), 7.30 (t, J = 7.4 Hz, 1H), 4.56 (dd, J = 14.1, 3.4 Hz, 1H), 4.39 (dd, J = 14.1, 6.1 Hz, 1H), 4.28–4.22 (m, 1H), 3.85–3.73 (m, 2H), 2.08–2.02 (m, 1H), 1.91–1.81 (m, 1H), 1.78–1.68 (m, 1H), 1.66–1.57 (m, 1H).

4-phenyl-1-(2-(phenylthio)ethyl)-1H-1,2,3-triazole (5ak): prepared from **4a** (0.2042 g, 2.0 mmol) and **3s** (0.3585 g, 2.0 mmol) by stirring for 15 h as a white solid in 88% yield (0.4952 g).

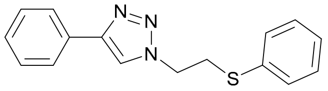

Melting point (M.P.): 86°C−88°C; ^{1}H NMR (400 MHz, $CDCl_3$) δ 7.80 (d, J = 7.8 Hz, 2H), 7.76 (s, 1H), 7.44–7.39 (m, 4H), 7.35–7.30 (m, 3H), 7.26–7.23 (m, 1H), 4.58 (t, J = 6.8 Hz, 2H), 3.44 (t, J = 6.8 Hz, 2H). ^{13}C NMR (101 MHz, $CDCl_3$) δ 147.7, 133.8, 130.5, 130.4, 129.3, 128.8, 128.2, 127.3, 125.7, 120.2, 49.5, 34.2. HRMS-ESI (m/z) [M+H$^+$] Calcd for $C_{16}H_{16}N_3S$ 282.1065; Found, 282.1067.

4-phenyl-1-(p-tolyl)-1H-1,2,3-triazole (5al)[22]: prepared from **4a** (0.2042 g, 2.0 mmol) and **3t** (0.2663 g, 2.0 mmol) by stirring for 15 h as a white solid in 93% yield (0.4376 g).

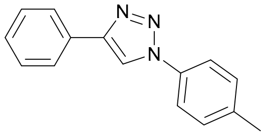

^{1}H NMR (400 MHz, $CDCl_3$) δ 8.16 (s, 1H), 7.91 (d, J = 7.0 Hz, 2H), 7.66 (d, J = 8.5 Hz, 2H), 7.45 (t, J = 7.5 Hz, 2H), 7.37 (d, J = 7.4 Hz, 1H), 7.33 (d, J = 8.4 Hz, 2H), 2.43 (s, 3H).

4-(4-ethylphenyl)-1-(p-tolyl)-1H-1,2,3-triazole (5am): prepared from **4c** (0.2604 g, 2.0 mmol) and **3t** (0.2663 g, 2.0 mmol) by stirring for 15 h as a white solid in 90% yield (0.4740 g).

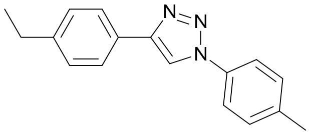

Melting point (M.P.): 154°C−157°C; ^{1}H NMR (400 MHz, $CDCl_3$) δ 8.12 (s, 1H), 7.82 (d, J = 7.8 Hz, 2H), 7.66 (d, J = 7.7 Hz, 2H), 7.33 (d, J = 7.6 Hz, 2H), 7.29 (d, J = 8.0 Hz, 2H), 2.73–2.43 (m, 2H), 2.43 (s, 3H), 1.28 (t, J = 6.0 Hz, 3H). ^{13}C NMR (101 MHz, $CDCl_3$) δ 148.4, 144.6, 138.8, 134.8, 130.2, 128.4, 127.7, 125.8, 120.4, 117.3, 28.7, 21.1, 15.5. HRMS-ESI (m/z) [M+H$^+$] Calcd for $C_{17}H_{18}N_3$ 264.1501; Found, 264.1503.

4-(4-propylphenyl)-1-(p-tolyl)-1H-1,2,3-triazole (5an): prepared from **4d** (0.2884 g, 2.0 mmol) and **3t** (0.2663 g, 2.0 mmol) by stirring for 15 h as a white solid in 91% yield (0.5048 g).

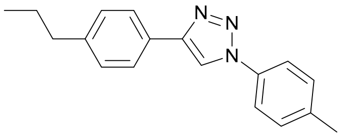

Melting point (M.P.): 145°C−147°C; ^{1}H NMR (400 MHz, $CDCl_3$) δ 8.12 (s, 1H), 7.81 (d, J = 7.9 Hz, 2H), 7.66 (d, J = 8.1 Hz, 2H), 7.33 (d, J = 8.1 Hz, 2H), 7.27 (d, J = 7.8 Hz, 2H), 2.63 (t, J = 7.6 Hz, 2H), 2.44 (s, 3H), 1.68 (m, 2H), 0.97 (t, J = 7.3 Hz, 3H). ^{13}C NMR (101 MHz, $CDCl_3$) δ 143.1, 138.8, 134.8, 130.2, 129.0, 127.8, 125.7, 120.4, 117.3, 37.8, 24.5, 21.1, 13.8. HRMS-ESI (m/z) [M+H$^+$] Calcd for $C_{18}H_{20}N_3$ 278.1657; Found, 278.1659.

4-(3-methoxyphenyl)-1-(p-tolyl)-1H-1,2,3-triazole (5ao): prepared from **4b** (0.2643 mg, 2.0 mmol) and **3t** (0.2663 g, 2.0 mmol) by stirring for 15 h as a white solid in 90% yield (0.4776 g).

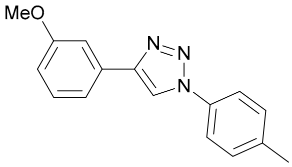

Melting point (M.P.): 169°C−171°C; ^{1}H NMR (400 MHz, $CDCl_3$) δ 8.14 (s, 1H), 7.66 (d, J = 8.5 Hz, 2H), 7.52 (dd, J = 2.7, 1.4 Hz, 1H), 7.43 (dt, J = 7.6, 1.3 Hz, 1H), 7.38–7.32 (m, 3H), 6.93–6.90 (m, 1H), 3.89 (s, 3H), 2.43 (s, 3H). ^{13}C NMR (101 MHz, $CDCl_3$) δ 160.0, 148.1, 138.9, 134.7, 131.6, 130.2, 129.9, 120.4, 118.2, 117.8, 114.4, 110.8, 55.4, 21.1. HRMS-ESI (m/z) [M+H$^+$] Calcd for $C_{16}H_{16}N_3O$ 266.1293; Found, 266.1294.

4-(phenoxymethyl)-1-(p-tolyl)-1H-1,2,3-triazole (5ap)[35]: prepared from **4q** (0.2643 g, 2.0 mmol) and **3t** (0.2663 g, 2.0 mmol) by stirring for 15 h as a white solid in 89% yield (0.4723 g).

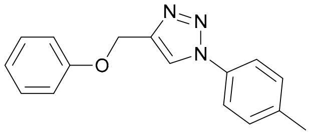

^{1}H NMR (400 MHz, $CDCl_3$) δ 8.01 (s, 1H), 7.61 (d, J = 7.9 Hz, 2H), 7.33–7.29 (m, 4H), 7.04–6.99 (m, 3H), 5.30 (s, 2H), 2.42 (s, 3H).

4-hexyl-1-(p-tolyl)-1H-1,2,3-triazole (5aq)[36]: prepared from **4o** (0.2204 g, 2.0 mmol) and **3t** (0.2663 g, 2.0 mmol) by stirring for 15 h as a white solid in 80% yield (0.3894 g).

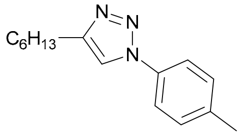

^{1}H NMR (400 MHz, $CDCl_3$) δ 7.68 (s, 1H), 7.59 (d, J = 8.3 Hz, 2H), 7.29 (d, J = 8.2 Hz, 2H), 2.78 (t, J = 7.7 Hz, 2H), 2.41 (s, 3H), 1.85–1.69 (m, 2H), 1.42–1.31 (m, 6H), 0.89 (t, J = 7.9 Hz, 3H).

1-(p-tolyl)-4-(trimethylsilyl)-1H-1,2,3-triazole (5ar)[22]: prepared from trimethylsilylacetylene (0.1964 g, 2.0 mmol) and **3t** (0.2663 g, 2.0 mmol) by stirring for 15 h as a white solid in 72% yield (0.3332 g).

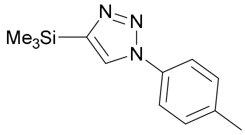

^{1}H NMR (400 MHz, $CDCl_3$) δ 7.90 (s, 1H), 7.60 (d, J = 8.1 Hz, 2H), 7.29 (d, J = 7.9 Hz, 2H), 2.41 (s, 3H), 0.37 (s, 9H).

1-(4-(tert-butyl)benzyl)-4-(4-methoxyphenyl)-1H-1,2,3-triazole (5as)[30]: prepared from **4x** (0.2643 g, 2.0 mmol) and **3y** (0.3785 g, 2.0 mmol) by stirring for 15 h as a white solid in 97% yield (0.6236 g).

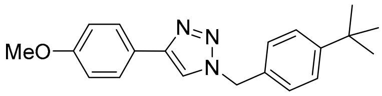

^{1}H NMR (400 MHz, $CDCl_3$) δ 7.71 (d, J = 8.8 Hz, 2H), 7.58 (s, 1H), 7.39 (d, J = 8.4 Hz, 2H), 7.24 (d, J = 8.2 Hz, 2H), 6.92 (d, J = 8.8 Hz, 2H), 5.50 (s, 2H), 3.81 (s, 3H), 1.31 (s, 9H).

2-(4-((1-(2-methylbenzyl)-1H-1,2,3-triazol-4-yl)methoxy)phenyl)ethan-1-ol (5at)[1]: prepared from **4y** (0.3524 g, 2.0 mmol) and **3f** (0.2944 g, 2.0 mmol) by stirring for 15 h as a white solid in 87% yield (0.5627 g).

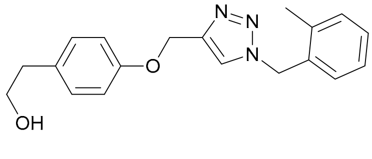

^{1}H NMR (400 MHz, $CDCl_3$) δ 7.41 (s, 1H), 7.31–7.28 (m, 1H), 7.24–7.20 (m, 2H), 7.17–7.12 (m, 3H), 6.91 (d, J = 8.6 Hz, 2H), 5.54 (s, 2H), 5.15 (s, 2H), 3.82 (t, J = 6.6 Hz, 2H), 2.80 (t, J = 6.5 Hz, 2H), 2.28 (s, 3H).

4-(3-chlorophenyl)-1-((2,2-dimethyl-1,3-dioxolan-4-yl)methyl)-1H-1,2,3-triazole (5au)[41]: prepared from **4h** (0.2732 g, 2.0 mmol) and **3b** (0.3143 g, 2.0 mmol) by stirring for 15 h as a white solid in 85% yield (0.4994 g).

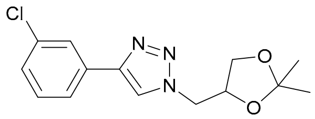

^{1}H NMR (400 MHz, $CDCl_3$) δ 7.93 (s, 1H), 7.83 (s, 1H), 7.73 (d, J = 7.5 Hz, 1H), 7.36 (t, J = 7.8 Hz, 1H), 7.31 (d, J = 8.3 Hz, 1H), 4.64–4.60 (m, 1H), 4.54–4.45 (m, 2H), 4.16 (dd, J = 8.8, 6.2 Hz, 1H), 3.78 (dd, J = 8.8, 5.8 Hz, 1H), 1.39 (d, J = 17.8 Hz, 6H).

1-(4-chlorobutyl)-4-phenyl-1H-1,2,3-triazole (5av)[42]: prepared from **4a** (0.2042 g, 2.0 mmol) and **3ac** (0.2672 g, 2.0 mmol) by stirring for 15 h as a white solid in 83% yield (0.3913 g).

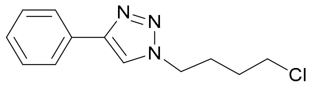

^{1}H NMR (400 MHz, $CDCl_3$) δ 7.83 (d, J = 7.0 Hz, 2H), 7.77 (s, 1H), 7.43 (t, J = 7.7 Hz, 2H), 7.34 (t, J = 7.4 Hz, 1H), 4.46 (t, J = 7.0 Hz, 2H), 3.59 (t, J = 6.3 Hz, 2H), 2.18–2.11 (m, 2H), 1.88–1.81 (m, 2H).

4-(3-nitrophenyl)-1-(m-tolyl)-1H-1,2,3-triazole (5aw)[43]: prepared from **4g** (0.2943 g, 2.0 mmol) and **3ad** (0.2663 g, 2.0 mmol) by stirring for 15 h as a white solid in 90% yield (0.5045 g).

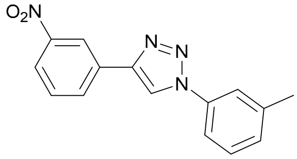

^{1}H NMR (400 MHz, $CDCl_3$) δ 8.69 (t, J = 2.0 Hz, 1H), 8.34–8.32 (m, 2H), 8.23–8.20 (m, 1H), 7.67–7.63 (m, 2H), 7.59 (dd, J = 7.9, 2.2 Hz, 1H), 7.45 (t, J = 7.8 Hz, 1H), 7.30 (d, J = 7.6 Hz, 1H), 2.48 (s, 3H).

1-(2-(4-(4-ethylphenyl)-1H-1,2,3-triazol-1-yl)phenyl)ethan-1-one (5ax)[44]: prepared from **4c** (0.2604 g, 2.0 mmol) and **3ae** (0.3223 g, 2.0 mmol) by stirring for 15 h as a white solid in 89% yield (0.5186 g).

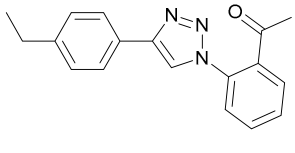

^{1}H NMR (400 MHz, $CDCl_3$) δ 8.06 (s, 1H), 7.83 (d, J = 8.2 Hz, 2H), 7.72 (dd, J = 7.5, 1.7 Hz, 1H), 7.66 (td, J = 7.6, 1.8 Hz, 1H), 7.60 (td, J = 7.6, 1.4 Hz, 1H), 7.54–7.52 (m, 1H), 7.30 (d, J = 8.2 Hz, 2H), 2.70 (q, J = 7.6 Hz, 2H), 2.20 (s, 3H), 1.28 (t, J = 7.6 Hz, 3H).

4-(4-((4-allyl-2-methoxyphenoxy)methyl)phenyl)-1-(4-(trifluoromethyl)phenyl)-1H-1,2,3-triazole (5ay)[2]: prepared from **4z** (0.5567 g, 2.0 mmol) and **3af** (0.3742 g, 2.0 mmol) by stirring for 15 h as a white solid in 84% yield (0.6542 mg).

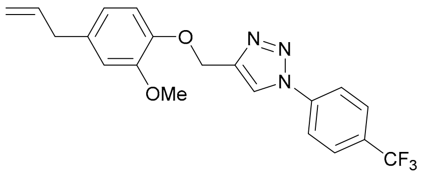

^{1}H NMR (400 MHz, $CDCl_3$) δ 8.15 (s, 1H), 7.89 (d, J = 9.1 Hz, 2H), 7.80 (d, J = 8.6 Hz, 2H), 6.99 (d, J = 8.1 Hz, 1H), 6.75–6.72 (m, 2H), 6.00–5.90 (m, 1H), 5.36 (s, 2H), 5.11–5.06 (m, 2H), 3.88 (s, 3H), 3.34 (d, J = 6.7 Hz, 2H).

1-(3-(azidomethyl)benzyl)-4-phenyl-1H-1,2,3-triazole (6a): prepared from **4a** (0.2042 g, 2.0 mmol) and **3d** (0.3764 g, 2.0 mmol) by stirring for 5.5 h as a white solid in 80% yield (0.4645 g).

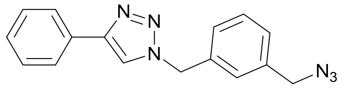

Melting point (M.P.): 55°C−57°C; ^{1}H NMR (400 MHz, $CDCl_3$) δ 7.80 (d, J = 7.0 Hz, 2H), 7.69 (s, 1H), 7.41–7.38 (m, 3H), 7.33–7.29 (m, 2H), 7.26 (t, J = 6.5 Hz, 2H), 5.57 (s, 2H), 4.34 (s, 2H). ^{13}C NMR (101 MHz, $CDCl_3$) δ 148.30, 136.51, 135.40, 130.37, 129.68, 128.79, 128.47, 128.20, 127.84, 127.58, 125.67, 119.50, 54.29, 53.91. HRMS-ESI (m/z) [M+H$^+$] Calcd for $C_{16}H_{15}N_6$ 291.1358; Found, 291.1359.

1-(2-(azidomethyl)benzyl)-4-phenyl-1H-1,2,3-triazole (6b): prepared from **4a** (0.2042 g, 2.0 mmol) and **3u** (0.3764 g, 2.0 mmol) by stirring for 5.5 h as a white solid in 72% yield (0.4180 g).

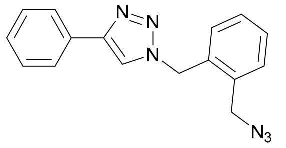

Melting point (M.P.): 115°C−117°C; ^{1}H NMR (400 MHz, $CDCl_3$) δ 7.80 (d, J = 7.1 Hz, 2H), 7.66 (s, 1H), 7.43–7.37 (m, 5H), 7.34–7.27 (m, 2H), 5.67 (s, 2H), 4.46 (s, 2H). ^{13}C NMR (101 MHz, $CDCl_3$) δ 148.22, 133.86, 133.27, 130.46, 130.35, 129.98, 129.47, 129.41, 128.80, 128.23, 125.68, 119.56, 52.32, 51.30. HRMS-ESI (m/z) [M+H$^+$] Calcd for $C_{16}H_{15}N_6$ 291.1358; Found, 291.1361.

1-(4-(azidomethyl)benzyl)-4-phenyl-1H-1,2,3-triazole (6c)[37]: prepared from **4a** (0.2042 g, 2.0 mmol) and **3v** (0.3764 g, 2.0 mmol) by stirring for 5.5 h as a white solid in 85% yield (0.4935 g).

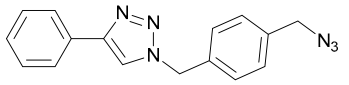

^{1}H NMR (400 MHz, $CDCl_3$) δ 7.80 (d, J = 7.1 Hz, 2H), 7.67 (s, 1H), 7.40 (t, J = 7.5 Hz, 2H), 7.36–7.30 (m, 5H), 5.59 (s, 2H), 4.36 (s, 2H).

1-(4-(azidomethyl)benzyl)-4-(2-methoxyphenyl)-1H-1,2,3-triazole (6d): prepared from **4t** (0.2643 g, 2.0 mmol) and **3v** (0.3764 g, 2.0 mmol) by stirring for 6 h as a white solid in 81% yield (0.5189 g).

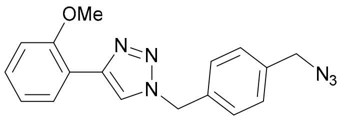

Melting point (M.P.): 61°C−63°C; ^{1}H NMR (400 MHz, $CDCl_3$) δ 8.37–8.34 (m, 1H), 7.98 (s, 1H), 7.34–7.28 (m, 5H), 7.08 (t, J = 7.9 Hz, 1H), 6.95 (d, J = 7.8 Hz, 1H), 5.60 (s, 2H), 4.34 (s, 2H), 3.89 (s, 3H). ^{13}C NMR (101 MHz, $CDCl_3$) δ 155.58, 143.67, 135.80, 135.28, 128.96, 128.79, 128.20, 127.63, 123.03, 121.02, 119.24, 110.72, 55.31, 54.28, 53.53. HRMS-ESI (m/z) [M+H$^+$] Calcd for $C_{17}H_{17}N_6O$ 321.1464; Found, 321.1466.

1-(4-(azidomethyl)benzyl)-4-(p-tolyl)-1H-1,2,3-triazole (6e): prepared from **4u** (0.2323 g, 2.0 mmol) and **3v** (0.3764 g, 2.0 mmol) by stirring for 5.5 h as a white solid in 86% yield (0.5235 g).

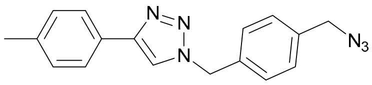

Melting point (M.P.): 122°C−124°C; ^{1}H NMR (400 MHz, $CDCl_3$) δ 8.37–8.34 (m, 1H), 7.98 (s, 1H), 7.34–7.28 (m, 5H), 7.08 (t, J = 7.9 Hz, 1H), 6.95 (d, J = 7.8 Hz, 1H), 5.60 (s, 2H), 4.34 (s, 2H), 3.89 (s, 3H). ^{13}C NMR (101 MHz, $CDCl_3$) δ 148.37, 138.05, 136.09, 134.84, 129.46, 128.84, 128.44, 127.58, 125.58, 119.13, 54.23, 53.73, 21.23. HRMS-ESI (m/z) [M+H$^+$] Calcd for $C_{17}H_{17}N_6$ 305.1515; Found, 305.1516.

4-(1-(4-(azidomethyl)benzyl)-1H-1,2,3-triazol-4-yl)-N,N-dimethylaniline (6f): prepared from **4v** (0.2904 g, 2.0 mmol) and **3v** (0.3764 g, 2.0 mmol) by stirring for 6 h as a white solid in 87% yield (0.5798 g).

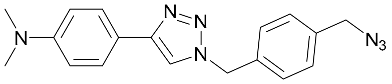

Melting point (M.P.): 117°C−122°C; ^{1}H NMR (400 MHz, $CDCl_3$) δ 7.67 (d, J = 8.9 Hz, 2H), 7.54 (s, 1H), 7.32 (q, J = 2.7 Hz, 4H), 6.75 (d, J = 8.8 Hz, 2H), 5.56 (s, 2H), 4.35 (s, 2H), 2.98 (s, 6H). ^{13}C NMR (101 MHz, $CDCl_3$) δ 150.36, 148.75, 135.90, 135.04, 128.77, 128.34, 126.59, 118.57, 118.00, 112.36, 54.22, 53.61, 40.39. HRMS-ESI (m/z) [M+H$^+$] Calcd for $C_{18}H_{20}N_7$ 334.1780; Found, 334.1781.

1-(4-(azidomethyl)benzyl)-4-(3-fluorophenyl)-1H-1,2,3-triazole (6g): prepared from **4j** (0.2403 g, 2.0 mmol) and **3v** (0.3764 g, 2.0 mmol) by stirring for 5.5 h as a white solid in 84% yield (0.5179 g).

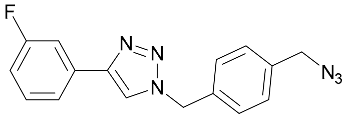

Melting point (M.P.): 113°C−116°C; ^{1}H NMR (400 MHz, $CDCl_3$) δ 7.68 (s, 1H), 7.57–7.51 (m, 2H),

7.38–7.32 (m, 5H), 7.03–6.98 (m, 1H), 5.59 (s, 2H), 4.36 (s, 2H). ^{13}C NMR (101 MHz, $CDCl_3$) δ 148.22, 133.86, 133.27, 130.46, 130.35, 129.98, 129.47, 129.41, 128.80, 128.23, 125.68, 119.56, 52.32, 51.30. ^{19}F NMR (376 MHz, $CDCl_3$) δ -112.64. HRMS-ESI (m/z) [M+H$^+$] Calcd for $C_{16}H_{14}FN_6$ 309.1264; Found, 309.1266.

1-(4-(azidomethyl)benzyl)-4-(3-chlorophenyl)-1H-1,2,3-triazole (6h): prepared from **4h** (0.2732 g, 2.0 mmol) and **3v** (0.3764 g, 2.0 mmol) by stirring for 5.5 h as a white solid in 89% yield (0.5781 g).

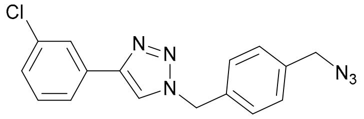

Melting point (M.P.): 112°C−114°C; ^{1}H NMR (400 MHz, $CDCl_3$) δ 7.79 (t, J = 1.8 Hz, 1H), 7.70–7.68 (m, 2H), 7.37–7.31 (m, 5H), 7.30–7.27 (m, 1H), 5.59 (s, 2H), 4.36 (s, 2H). ^{13}C NMR (101 MHz, $CDCl_3$) δ 146.98, 136.25, 134.73, 134.52, 132.16, 130.08, 128.89, 128.49, 128.15, 125.71, 123.72, 119.85, 54.19, 53.85. HRMS-ESI (m/z) [M+H$^+$] Calcd for $C_{16}H_{14}ClN_6$ 325.0968; Found, 325.0969.

1-(4-(azidomethyl)benzyl)-4-(4-(trifluoromethyl)phenyl)-1H-1,2,3-triazole (6i): prepared from **4k** (0.3403 g, 2.0 mmol) and **3v** (0.3764 g, 2.0 mmol) by stirring for 5.5 h as a white solid in 90% yield (0.6449 g).

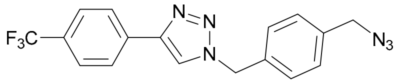

Melting point (M.P.): 91°C−93°C; ^{1}H NMR (400 MHz, $CDCl_3$) δ 7.92 (d, J = 8.2 Hz, 2H), 7.75 (s, 1H), 7.66 (d, J = 8.2 Hz, 2H), 7.35 (q, J = 1.8 Hz, 4H), 5.61 (s, 2H), 4.37 (s, 2H). ^{13}C NMR (101 MHz, $CDCl_3$) δ 146.92, 136.35, 134.46, 133.84, 130.03 (q, J = 32.3 Hz), 128.95, 128.54, 125.79 (q, J = 4.0 Hz), 124.0 (q, J = 273.7 Hz), 120.23, 54.21, 53.92. ^{19}F NMR (376 MHz, $CDCl_3$) δ -62.60. HRMS-ESI (m/z) [M+H$^+$] Calcd for $C_{17}H_{14}F_3N_6$ 359.1232; Found, 359.1235.

methyl 4-(1-(4-(azidomethyl)benzyl)-1H-1,2,3-triazol-4-yl)benzoate (6j): prepared from **4f** (0.3203 g, 2.0 mmol) and **3v** (0.3764 g, 2.0 mmol) by stirring for 6 h as a white solid in 88% yield (0.6131 g).

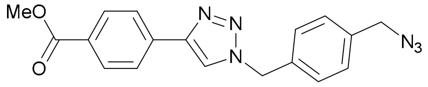

Melting point (M.P.): 149°C−152°C; ^{1}H NMR (400 MHz, $CDCl_3$) δ 8.07 (d, J = 8.4 Hz, 2H), 7.88 (d, J = 6.8 Hz, 2H), 7.77 (s, 1H), 7.37–7.32 (m, 4H), 5.60 (s, 2H), 4.36 (s, 2H), 3.92 (s, 3H). ^{13}C NMR (101 MHz, $CDCl_3$) δ 166.71, 147.23, 136.28, 134.69, 134.49, 130.15, 129.58, 128.91, 128.51, 125.43, 120.36, 54.19, 53.88, 52.11. HRMS-ESI (m/z) [M+H$^+$] Calcd for $C_{18}H_{17}N_6O_2$ 349.1413; Found, 349.1414.

1-(4-(azidomethyl)benzyl)-4-(3-nitrophenyl)-1H-1,2,3-triazole (6k): prepared from **4g** (0.2943 g, 2.0 mmol) and **3v** (0.3764 g, 2.0 mmol) by stirring for 7 h as a white solid in 84% yield (0.5633 g).

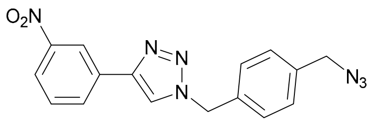

Melting point (M.P.): 111°C−112°C; ^{1}H NMR (400 MHz, $CDCl_3$) δ 8.56 (t, J = 1.9 Hz, 1H), 8.23 (d, J = 7.8 Hz, 1H), 8.18–8.15 (m, 1H), 7.82 (s, 1H), 7.60 (t, J = 8.0 Hz, 1H), 7.39–7.35 (m, 4H), 5.62 (s, 2H), 4.38 (s, 2H). ^{13}C NMR (101 MHz, $CDCl_3$) δ 148.59, 146.12, 136.46, 134.29, 132.21, 131.44, 129.89, 128.98, 128.61, 122.77, 120.43, 120.36, 54.20, 54.03. HRMS-ESI (m/z) [M+H$^+$] Calcd for $C_{16}H_{14}N_7O_2$ 336.1209; Found, 336.1211.

2-(1-(4-(azidomethyl)benzyl)-1H-1,2,3-triazol-4-yl)pyridine (6l): prepared from **4m** (0.2062 g, 2.0 mmol) and **3v** (0.3764 g, 2.0 mmol) by stirring for 5.5 h as a white solid in 82% yield (0.4778 g).

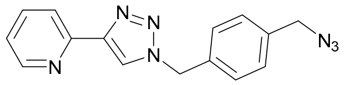

Melting point (M.P.): 58°C−60°C; ^{1}H NMR (400 MHz, $CDCl_3$) δ 8.54 (d, J = 4.5 Hz, 1H), 8.18 (d, J = 6.9 Hz, 1H), 8.07 (s, 1H), 7.77 (td, J = 7.8, 1.8 Hz, 1H), 7.37–7.32 (m, 4H), 7.23–7.20 (m, 1H), 5.60 (s, 2H), 4.35 (s, 2H). ^{13}C NMR (101 MHz, $CDCl_3$) δ 150.07, 149.30, 148.76, 136.90, 136.19, 134.43, 128.84, 128.68, 122.88, 121.91, 120.20, 54.18, 53.90. HRMS-ESI (m/z) [M+H$^+$] Calcd for $C_{15}H_{14}N_7$ 292.1311; Found, 292.1313.

1-(4-(azidomethyl)benzyl)-4-(thiophen-2-yl)-1H-1,2,3-triazole (6m): prepared from **4l** (0.2163 g, 2.0 mmol) and **3v** (0.3764 g, 2.0 mmol) by stirring for 6 h as a white solid in 78% yield (0.4623 g).

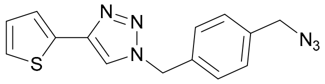

Melting point (M.P.): 106°C−108°C; ^{1}H NMR (400 MHz, $CDCl_3$) δ 7.59 (s, 1H), 7.36–7.31 (m, 5H), 7.28 (dd, J = 5.1, 1.2 Hz, 1H), 7.05 (dd, J = 5.1, 3.6 Hz, 1H), 5.57 (s, 2H), 4.36 (s, 2H). ^{13}C NMR (101 MHz, $CDCl_3$) δ 143.38, 136.23, 134.58, 132.73, 128.89, 128.48, 127.57, 125.09, 124.21, 118.95, 54.23, 53.82. HRMS-ESI (m/z) [M+H$^+$] Calcd for $C_{14}H_{13}N_6S$ 297.0922; Found, 297.0923.

1-(8-azidooctyl)-4-phenyl-1H-1,2,3-triazole (6n): prepared from **4a** (0.2042 g, 2.0 mmol) and **3w** (0.3925 g, 2.0 mmol) by stirring for 12 h as a white solid in 69% yield (0.4118 g).

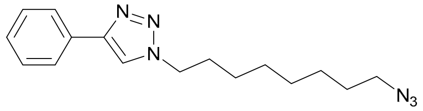

Melting point (M.P.): 64°C−66°C; ^{1}H NMR (400 MHz, $CDCl_3$) δ 7.83 (d, J = 7.1 Hz, 2H), 7.74 (s, 1H), 7.43 (t, J = 7.7 Hz, 2H), 7.33 (t, J = 7.4 Hz, 1H), 4.40 (t, J = 7.2 Hz, 2H), 3.25 (t, J = 6.9 Hz, 2H), 1.99–1.92 (m, 2H), 1.62–1.55 (m, 2H), 1.42–1.29 (m, 8H). ^{13}C NMR (101 MHz, $CDCl_3$) δ 147.73, 130.66, 128.81, 128.08, 125.66, 119.35, 51.36, 50.35, 30.27, 28.86, 28.83, 28.73, 26.54, 26.35. HRMS-ESI (m/z) [M+H$^+$] Calcd for $C_{16}H_{23}N_6$ 299.1984; Found, 299.1985.

1-benzyl-4-(3-ethynylphenyl)-1H-1,2,3-triazole (7a): prepared from **4w** (0.2523 g, 2.0 mmol) and **3a** (0.2663 g, 2.0 mmol) by stirring for 6 h as a white solid in 91% yield (0.4719 g).

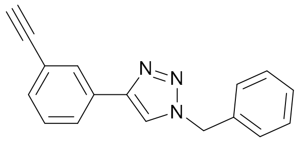

Melting point (M.P.): 109°C−111°C; ^{1}H NMR (400 MHz, $CDCl_3$) δ 7.88 (s, 1H), 7.83 (d, J = 7.7 Hz, 1H), 7.67 (s, 1H), 7.43 (d, J = 7.7 Hz, 1H), 7.40–7.34 (q, J = 7.4 Hz, 4H), 7.32–7.30 (m, 2H), 5.57 (s, 2H), 3.09 (s, 1H). ^{13}C NMR (101 MHz, $CDCl_3$) δ 147.23, 134.45, 131.66, 130.74, 129.24, 129.16, 128.85, 128.83, 128.08, 126.01, 122.60, 119.71, 83.19, 77.52, 54.26. HRMS-ESI (m/z) [M+H^{+}] Calcd for $C_{17}H_{14}N_3$ 260.1188; Found, 260.1190.

1-(3-chlorobenzyl)-4-(3-ethynylphenyl)-1H-1,2,3-triazole (7b): prepared from **4w** (0.2523 g, 2.0 mmol) and **3x** (0.3353 g, 2.0 mmol) by stirring for 6 h as a white solid in 88% yield (0.5170 g).

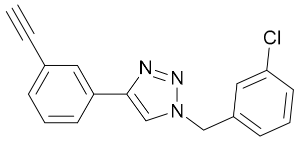

Melting point (M.P.): 118°C−120°C; ^{1}H NMR (400 MHz, $CDCl_3$) δ 7.90 (s, 1H), 7.83 (d, J = 7.7 Hz, 1H), 7.71 (s, 1H), 7.44 (d, J = 7.7 Hz, 1H), 7.39–7.30 (m, 4H), 7.18 (d, J = 6.9 Hz, 1H), 5.54 (s, 2H), 3.09 (s, 1H). ^{13}C NMR (101 MHz, $CDCl_3$) δ 147.41, 136.41, 135.02, 131.76, 130.56, 130.45, 129.26, 129.02, 128.88, 128.06, 126.06, 126.02, 122.65, 119.77, 83.14, 77.60, 53.50. HRMS-ESI (m/z) [M+H^{+}] Calcd for $C_{17}H_{13}ClN_3$ 294.0798; Found, 294.0799.

8-((4-(3-ethynylphenyl)-1H-1,2,3-triazol-1-yl)methyl)quinoline (7c): prepared from **4w** (0.2523 g, 2.0 mmol) and **3k** (0.3684 g, 2.0 mmol) by stirring for 6 h as a white solid in 86% yield (0.5338 g).

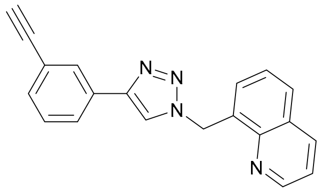

Melting point (M.P.): 98°C−100°C; ^{1}H NMR (400 MHz, $CDCl_3$) δ 9.02–9.00 (m, 1H), 8.20 (d, J = 9.4 Hz, 1H), 8.00 (s, 1H), 7.86–7.80 (m, 3H), 7.69 (d, J = 6.9 Hz, 1H), 7.53–7.47 (m, 2H), 7.40 (d, J = 7.7 Hz, 1H), 7.33 (t, J = 7.7 Hz, 1H), 6.27 (s, 2H), 3.07 (s, 1H). ^{13}C NMR (101 MHz, $CDCl_3$) δ 150.23, 146.68, 145.79, 136.41, 133.12, 131.42, 131.03, 129.94, 129.17, 128.96, 128.73, 128.36, 126.42, 126.00, 122.45, 121.64, 120.86, 83.29, 77.39, 49.85. HRMS-ESI (m/z) [M+H^{+}] Calcd for $C_{20}H_{15}N_4$ 311.1297; Found, 311.1298.

1-(4-(tert-butyl)benzyl)-4-(3-ethynylphenyl)-1H-1,2,3-triazole (7d): prepared from **4w** (0.2523 g, 2.0 mmol) and **3y** (0.3785 g, 2.0 mmol) by stirring for 6 h as a white solid in 80% yield (0.5046 g).

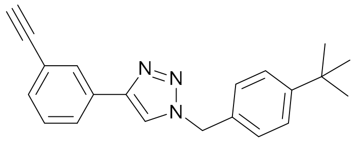

Melting point (M.P.): 127°C−129°C; ^{1}H NMR (400 MHz, $CDCl_3$) δ 7.88 (s, 1H), 7.84 (d, J = 7.7 Hz, 1H), 7.66 (s, 1H), 7.44–7.40 (m, 3H), 7.36 (t, J = 7.7 Hz, 1H), 7.25 (d, J = 7.4 Hz, 2H), 5.54 (s, 2H), 3.08 (s, 1H), 1.32 (s, 9H). ^{13}C NMR (101 MHz, $CDCl_3$) δ 151.97, 147.13, 131.60, 131.41, 130.83, 129.23, 128.82, 127.91, 126.08, 126.02, 122.57, 119.69, 83.22, 77.49, 53.96, 34.61, 31.20. HRMS-ESI (m/z) [M+H$^+$] Calcd for $C_{21}H_{22}N_3$ 316.1814; Found, 316.1815.

4-(3-ethynylphenyl)-1-phenethyl-1H-1,2,3-triazole (7e): prepared from **4w** (0.2523 g, 2.0 mmol) and **3z** (0.2944 g, 2.0 mmol) by stirring for 12 h as a white solid in 73% yield (0.3990 g).

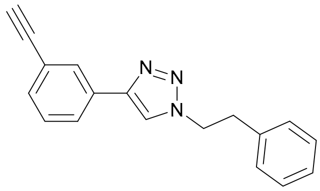

Melting point (M.P.): 104°C−106°C; ^{1}H NMR (400 MHz, $CDCl_3$) δ 7.86 (s, 1H), 7.79 (d, J = 7.8 Hz, 1H), 7.47 (s, 1H), 7.44 (d, J = 7.7 Hz, 1H), 7.37 (t, J = 7.8 Hz, 1H), 7.33–7.24 (m, 3H), 7.14–7.12 (m, 2H), 4.64 (t, J = 7.2 Hz, 2H), 3.25 (t, J = 7.2 Hz, 2H), 3.10 (s, 1H). ^{13}C NMR (101 MHz, $CDCl_3$) δ146.48, 136.90, 131.58, 130.83, 129.20, 128.84, 128.82, 128.66, 127.13, 126.00, 122.59, 120.14, 83.21, 77.51, 51.75, 36.70. HRMS-ESI (m/z) [M+H$^+$] Calcd for $C_{18}H_{16}N_3$ 274.1344; Found, 274.1347.

4-(3-ethynylphenyl)-1-hexyl-1H-1,2,3-triazole (7f): prepared from **4w** (0.2523 g, 2.0 mmol) and **3c** (0.2544 g, 2.0 mmol) by stirring for 12 h as a white solid in 67% yield (0.3395 g).

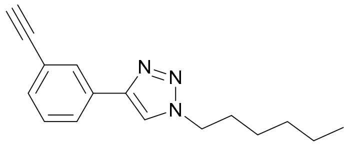

Melting point (M.P.): 61°C−63°C; ^{1}H NMR (400 MHz, $CDCl_3$) δ 7.93 (s, 1H), 7.87 (d, J = 7.7 Hz, 1H), 7.76 (s, 1H), 7.45 (d, J = 7.7 Hz, 1H), 7.39 (t, J = 7.7 Hz, 1H), 4.40 (t, J = 7.2 Hz, 2H), 3.11 (s, 1H), 1.98–1.91 (m, 2H), 1.37–1.30 (m, 6H), 0.89 (t, J = 7.6 Hz, 3H). ^{13}C NMR (101 MHz, $CDCl_3$) δ 146.73, 131.57, 130.94, 129.23, 128.86, 126.01, 122.61, 119.61, 83.25, 77.48, 50.45, 31.11, 30.24, 26.11, 22.37, 13.90. HRMS-ESI (m/z) [M+H$^+$] Calcd for $C_{16}H_{20}N_3$ 254.1657; Found, 254.1658.

1-(4-(azidomethyl)benzyl)-4-(3-ethynylphenyl)-1H-1,2,3-triazole (7g): prepared from **4w** (0.2523 g, 2.0 mmol) and **3v** (0.3764 g, 2.0 mmol) by stirring for 5.5 h as a white solid in 76% yield (0.4778 g).

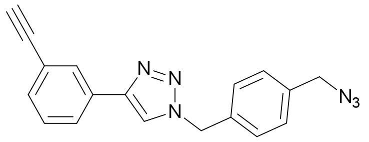

Melting point (M.P.): 114°C−116°C; ^{1}H NMR (400 MHz, $CDCl_3$) δ 7.89 (s, 1H), 7.84 (d, J = 7.7 Hz, 1H), 7.68 (s, 1H), 7.44 (d, J = 7.7 Hz, 1H), 7.39–7.31 (m, 5H), 5.59 (s, 2H), 4.36 (s, 2H), 3.09 (s, 1H). ^{13}C NMR (101 MHz, $CDCl_3$) δ 147.36, 136.24, 134.59, 131.73, 130.66, 129.27, 128.91, 128.51, 126.03, 122.64, 119.71, 83.17, 77.56, 54.21, 53.85. HRMS-ESI (m/z) [M+H$^+$] Calcd for $C_{18}H_{15}N_6$ 315.1358; Found, 315.1359.

1-(8-azidooctyl)-4-(3-ethynylphenyl)-1H-1,2,3-triazole (7h): prepared from **4w** (0.2523 g, 2.0 mmol) and **3w** (0.3925 g, 2.0 mmol) by stirring for 12 h as a white solid in 60% yield (0.3868 g).

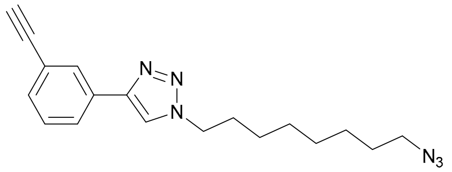

Melting point (M.P.): 40°C−42°C; ^{1}H NMR (400 MHz, $CDCl_3$) δ 7.93 (s, 1H), 7.86 (d, J = 7.8 Hz, 1H), 7.76 (s, 1H), 7.45 (d, J = 7.8 Hz, 1H), 7.39 (t, J = 7.7 Hz, 1H), 4.39 (t, J = 7.2 Hz, 2H), 3.25 (t, J = 6.9 Hz, 2H), 3.11 (s, 1H), 1.98–1.91 (m, 2H), 1.62–1.55 (m, 2H), 1.37–1.33 (m, 8H). ^{13}C NMR (101 MHz, $CDCl_3$) δ 146.73, 131.57, 130.88, 129.20, 128.86, 125.98, 122.60, 119.62, 83.21, 77.51, 51.31, 50.37, 30.20, 28.82, 28.78, 28.69, 26.50, 26.29. HRMS-ESI (m/z) [M+H$^+$] Calcd for $C_{18}H_{23}N_6$ 323.1984; Found, 323.1986.

1,3-bis(1-benzyl-1H-1,2,3-triazol-4-yl)benzene (8a)[29]: prepared from **4w** (0.1262 g, 1.0 mmol) and **3a** (0.3994 g, 3.0 mmol) by stirring for 8 h as a white solid in 93% yield (0.3650 g).

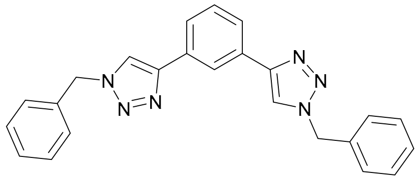

Melting point (M.P.): 133°C−135°C; ^{1}H NMR (400 MHz, $CDCl_3$) δ 8.18 (s, 1H), 7.78 (dd, J = 7.8, 1.8 Hz, 2H), 7.74 (s, 2H), 7.46–7.37 (m, 7H), 7.32 (dd, J = 7.4, 2.1 Hz, 4H), 5.58 (s, 4H).

1,3-bis(1-hexyl-1H-1,2,3-triazol-4-yl)benzene (8b)[38]: prepared from **4w** (0.1262 g, 1.0 mmol) and **3c** (0.3816 g, 3.0 mmol) by stirring for 15 h as a white solid in 90% yield (0.3425 g).

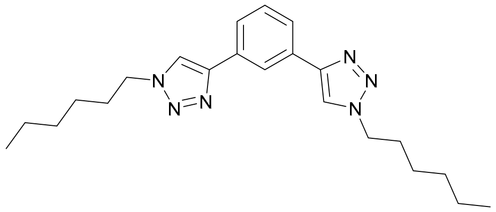

Melting point (M.P.): 92°C−95°C; ^{1}H NMR (400 MHz, $CDCl_3$) δ 8.30 (s, 1H), 7.86 (s, 2H), 7.84 (d, J = 1.7 Hz, 1H), 7.82 (d, J = 1.7 Hz, 1H), 7.48 (t, J = 7.7 Hz, 1H), 4.41 (t, J = 7.2 Hz, 4H), 1.98–1.91 (m, 4H), 1.37–1.30 (m, 12H), 0.89 (t, J = 6.2 Hz, 6H).

1,3-bis((4-phenyl-1H-1,2,3-triazol-1-yl)methyl)benzene (8c)[17]: prepared from **4a** (0.3064 g, 3.0 mmol) and **3d** (0.1882 g, 1.0 mmol) by stirring for 8 h as a white solid in 95% yield (0.3728 g).

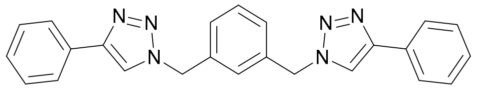

^{1}H NMR (400 MHz, $CDCl_3$) δ 7.79 (d, J = 7.0 Hz, 4H), 7.70 (s, 2H), 7.39 (t, J = 7.4 Hz, 5H), 7.30 (dd, J = 19.2, 7.3 Hz, 5H), 5.56 (s, 4H).

1,3-bis((4-(2-methoxyphenyl)-1H-1,2,3-triazol-1-yl)methyl)benzene (8d): prepared from **4t** (0.3965 g, 3.0 mmol) and **3d** (0.1882 g, 1.0 mmol) by stirring for 12 h as a white solid in 92% yield (0.4163 g).

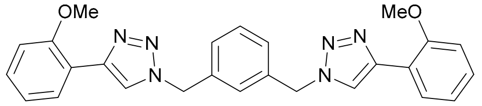

Melting point (M.P.): 176°C−178°C; ^{1}H NMR (400 MHz, $CDCl_3$) δ 8.35 (dd, J = 7.8, 1.8 Hz, 2H), 7.99 (s, 2H), 7.36 (t, J = 7.5 Hz, 1H), 7.33–7.28 (m, 2H), 7.24 (s, 3H), 7.07 (t, J = 7.6 Hz, 2H), 6.93 (d, J = 9.4 Hz, 2H), 5.56 (s, 4H), 3.84 (s, 6H). ^{13}C NMR (101 MHz, $CDCl_3$) δ 155.54, 143.63, 136.10, 129.72, 128.95, 127.78, 127.49, 126.86, 123.10, 120.92, 119.10, 110.69, 55.23, 53.50. HRMS-ESI (m/z) [M+H$^+$] Calcd for $C_{26}H_{25}N_6O_2$ 453.2039; Found, 453.2041.

1,3-bis((4-(3-chlorophenyl)-1H-1,2,3-triazol-1-yl)methyl)benzene (8e): prepared from **4h** (0.4097 g, 3.0 mmol) and **3d** (0.1882 g, 1.0 mmol) by stirring for 15 h as a white solid in 75% yield (0.3460 g).

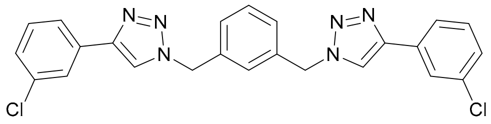

Melting point (M.P.): 184°C−186°C; ^{1}H NMR (400 MHz, $CDCl_3$) δ 7.79 (s, 2H), 7.71 (s, 2H), 7.68 (d, J = 7.4 Hz, 2H), 7.43 (t, J = 6.2 Hz, 1H), 7.35–7.28 (m, 7H), 5.58 (s, 4H). ^{13}C NMR (101 MHz, DMSO-d_6) δ 145.34, 136.48, 133.69, 132.71, 130.83, 129.42, 127.84, 127.67, 127.46, 124.76, 123.66, 122.36, 52.87. HRMS-ESI (m/z) [M+H$^+$] Calcd for $C_{24}H_{19}Cl_2N_6$ 461.1048; Found, 461.1049.

6. General Procedure F for Synthesis of *N*-Sulfonyl-1,2,3-triazoles (9a–9h)

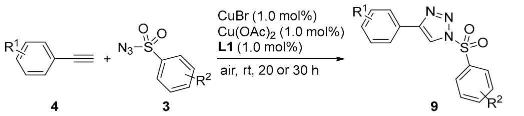

A mixture of CuBr (0.0014 g, 0.01 mmol), $Cu(OAc)_2$ (0.0020 g, 0.01 mmol), ligand **L1** (0.0028 g, 0.01 mmol), ethyne **4** (1.0 mmol) and azide **3** (1.0 mmol) was stirred under air at rt for 20 h or 32 h. Then washed the reaction mixture with water twice followed by petroleum ether for three times. After drying, the desired **9a**–**9h** were obtained in pure forms.

4-Phenyl-1-(phenylsulfonyl)-1*H*-1,2,3-triazole (9a)[39]: prepared from **4a** (0.1021 g, 1.0 mmol) and **3aa** (0.1832 g, 1.0 mmol) as a white solid in 99% yield (0.2825 g).

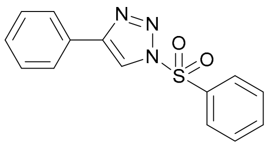

^{1}H NMR (400 MHz, $CDCl_3$) δ 8.34 (s, 1H), 8.16 (d, J = 7.2 Hz, 2H), 7.83 (d, J = 6.9 Hz, 2H), 7.74 (t, J = 7.5 Hz, 1H), 7.62 (d, J = 15.6 Hz, 2H), 7.44 (t, J = 7.3 Hz, 2H), 7.38 (t, J = 7.3 Hz, 1H).

4-Phenyl-1-tosyl-1*H*-1,2,3-triazole (9b)[40]: prepared from **4a** (0.1021 g, 1.0 mmol) and **3ab** (0.1972 g, 1.0 mmol) as a white solid in 95% yield (0.2844 g).

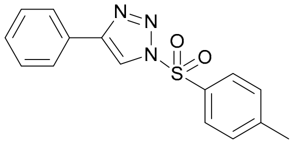

^{1}H NMR (400 MHz, $CDCl_3$) δ 8.32 (s, 1H), 8.02 (d, J = 8.5 Hz, 2H), 7.83–7.81 (m, 2H), 7.45–7.34 (m, 5H), 2.44 (s, 3H).

4-(3-Fluorophenyl)-1-(phenylsulfonyl)-1*H*-1,2,3-triazole (9c)[40]: prepared from **4j** (0.1201 g, 1.0 mmol) and **3aa** (0.1832 g, 1.0 mmol) by stirring for 30 min as a white solid in 89% yield (0.2699 g).

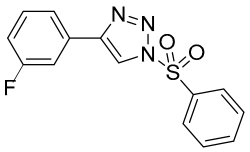

^{1}H NMR (400 MHz, $CDCl_3$) δ 8.36 (s, 1H), 8.16 (d, J = 7.2 Hz, 2H), 7.74 (t, J = 7.5 Hz, 1H), 7.64–7.55 (m, 4H), 7.43–7.37 (m, 1H), 7.09–7.04 (m, 1H). ^{13}C NMR (101 MHz, $CDCl_3$) δ 163.10 (d, J = 248.5 Hz), 146.30, 136.01, 135.80, 130.91, 130.68 (d, J = 8.1 Hz), 129.89, 128.69, 121.71, 119.43, 116.06 (d, J = 21.0 Hz), 113.10 (d, J = 23.2 Hz). ^{19}F NMR (376 MHz, $CDCl_3$) δ -111.94.

4-(4-Fluorophenyl)-1-(phenylsulfonyl)-1*H*-1,2,3-triazole (9d): prepared from **4aa** (0.1201 g, 1.0 mmol) and **3aa** (0.1832 g, 1.0 mmol) as a white solid in 90% yield (0.2730 g).

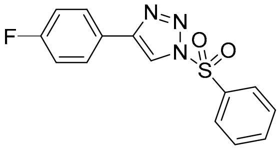

^{1}H NMR (400 MHz, $CDCl_3$) δ 8.31 (s, 1H), 8.15 (d, J = 7.3 Hz, 2H), 7.81 (dd, J = 8.8, 5.2 Hz, 2H), 7.74 (t, J = 7.5 Hz, 1H), 7.61 (t, J = 7.9 Hz, 2H), 7.12 (t, J = 8.7 Hz, 2H). ^{13}C NMR (101 MHz, $CDCl_3$) δ 163.18 (d, J = 249.5 Hz), 146.55, 136.08, 135.73, 129.86, 128.65, 127.94 (d, J = 9.1 Hz), 124.99, 118.73, 116.10 (d, J = 21.2 Hz). ^{19}F NMR (376 MHz, $CDCl_3$) δ -111.67. HRMS-ESI (m/z) [$M+H^+$] Calcd for $C_{14}H_{11}FN_3O_2S$ 304.0556; Found, 304.0557.

4-(2-bromophenyl)-1-tosyl-1*H*-1,2,3-triazole (9e)[40]: prepared from **4i** (0.1810 g, 1.0 mmol) and **3ab** (0.1972 g, 1.0 mmol) as a white solid in 88% yield (0.3328 g).

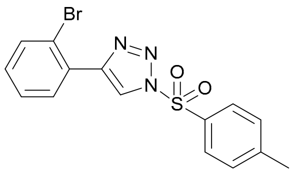

^{1}H NMR (400 MHz, $CDCl_3$) δ 8.80 (s, 1H), 8.09–8.04 (m, 3H), 7.66 (d, J = 8.0 Hz, 1H), 7.42–7.39 (m, 3H), 7.22 (d, J = 7.7 Hz, 1H), 2.46 (s, 3H).

1-(Phenylsulfonyl)-4-(p-tolyl)-1*H*-1,2,3-triazole (9f): prepared from **4u** (0.1162 g, 1.0 mmol) and **3aa** (0.1832 g, 1.0 mmol) as a white solid in 93% yield (0.2784 g).

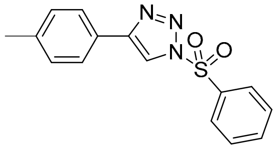

^{1}H NMR (400 MHz, $CDCl_3$) δ 8.29 (s, 1H), 8.15 (d, J = 7.5 Hz, 2H), 7.71 (d, J = 8.1 Hz, 3H), 7.60 (t, J = 7.9 Hz, 2H), 7.23 (d, J = 8.0 Hz, 2H), 2.37 (s, 3H). ^{13}C NMR (101 MHz, $CDCl_3$) δ 143.43, 141.64, 130.80, 130.26, 129.72, 129.51, 128.61, 128.50, 126.64, 125.99, 21.42. HRMS-ESI (m/z) [M+H$^+$] Calcd for $C_{15}H_{14}N_3O_2S$ 300.0807; Found, 300.0809.

4-(4-Ethylphenyl)-1-tosyl-1*H*-1,2,3-triazole (9g)[40]: prepared from **4c** (0.1302 g, 2.0 mmol) and **3ab** (0.1972 g, 1.0 mmol) by stirring for 30 min as a white solid in 92% yield (0.3012 g).

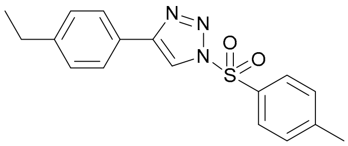

^{1}H NMR (400 MHz, $CDCl_3$) δ 8.28 (s, 1H), 8.01 (d, J = 8.5 Hz, 2H), 7.73 (d, J = 8.3 Hz, 2H), 7.37 (d, J = 8.2 Hz, 2H), 7.25 (d, J = 8.3 Hz, 2H), 2.66 (q, J = 7.6 Hz, 2H), 2.43 (s, 3H), 1.24 (t, J = 7.6 Hz, 3H).

4-(4-Methoxyphenyl)-1-(phenylsulfonyl)-1*H*-1,2,3-triazole (9h): prepared from **4x** (0.1322 g, 1.0 mmol) and **3aa** (0.1832 g, 1.0 mmol) as a white solid in 94% yield (0.2964 g).

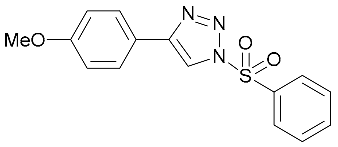

^{1}H NMR (400 MHz, $CDCl_3$) δ 8.25 (s, 1H), 8.14 (d, J = 7.4 Hz, 2H), 7.73 (dd, J = 19.4, 8.2 Hz, 3H), 7.59 (t, J = 7.9 Hz, 2H), 6.95 (d, J = 8.8 Hz, 2H), 3.83 (s, 3H). ^{13}C NMR (101 MHz, $CDCl_3$) δ 160.24, 147.33, 136.15, 135.56, 129.76, 128.49, 127.40, 121.25, 118.03, 114.35, 55.28. HRMS-ESI (m/z) [M+H$^+$] Calcd for $C_{15}H_{14}N_3O_3S$ 316.0756; Found, 316.0758.

7. X-Ray Crystallographic Data

7.1 X-Ray Crystallographic Data for 6k

X-Ray crystallographic data for compound **6k** was recorded at 296 K on a Rigaku Oxford Diffraction Supernova Dual Source, Cu at Zero equipped with an AtlasS2 CCD using Cu Kα radiation. Data reduction was carried out with the diffractometer software. The structures were solved by direct methods using Olex2 software[45] and the non-hydrogen atoms were located from the trial structure and then refined anisotropically with SHELXL-2014[46] using a full-matrix least squares procedure based on F2. The weighted R factor, wR and goodness-of-fit S values were obtained based on F2.The hydrogen atom positions were fixed geometrically at the calculated distances and allowed to ride on their parent atoms. Crystallographic data for the structure reported in this paper have been deposited at the Cambridge Crystallographic Data Center and allocated with the deposition numbers: 2472586.

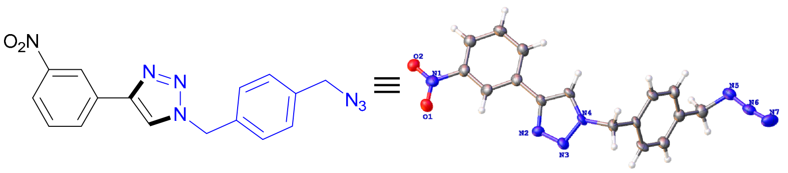

6k CCDC 2472586

Figure S1. ORTEP drawing of **6k**

Crystal data and structure refinement for 6k.

Identification code	**6k**
Empirical formula	$C_{16}H_{13}N_7O_2$
Formula weight	335.33
Temperature/K	100.01(10)
Crystal system	triclinic
Space group	P1
a/Å	5.6360(3)
b/Å	10.2411(5)
c/Å	14.5137(12)
α/°	107.404(6)
β/°	97.794(6)
γ/°	99.047(5)
Volume/Å^3	774.45(9)
Z	2
ρ_{calc}g/cm^3	1.438
μ/mm^{-1}	0.843
F(000)	348.0
Crystal size/mm^3	0.16 × 0.1 × 0.08
Radiation	Cu Kα (λ = 1.54184)
2Θ range for data collection/°	9.254 to 133.198
Index ranges	-6 ≤ h ≤ 6, -12 ≤ k ≤ 12, -17 ≤ l ≤ 17
Reflections collected	4448

Independent reflections	4448 [R_{int} = ?, R_{sigma} = 0.0147]
Data/restraints/parameters	4448/3/452
Goodness-of-fit on F^2	1.102
Final R indexes [I>=2σ (I)]	R_1 = 0.0835, wR_2 = 0.2405
Final R indexes [all data]	R_1 = 0.0904, wR_2 = 0.2517
Largest diff. peak/hole / e Å^{-3}	0.35/-0.41
Flack parameter	0.0(5)

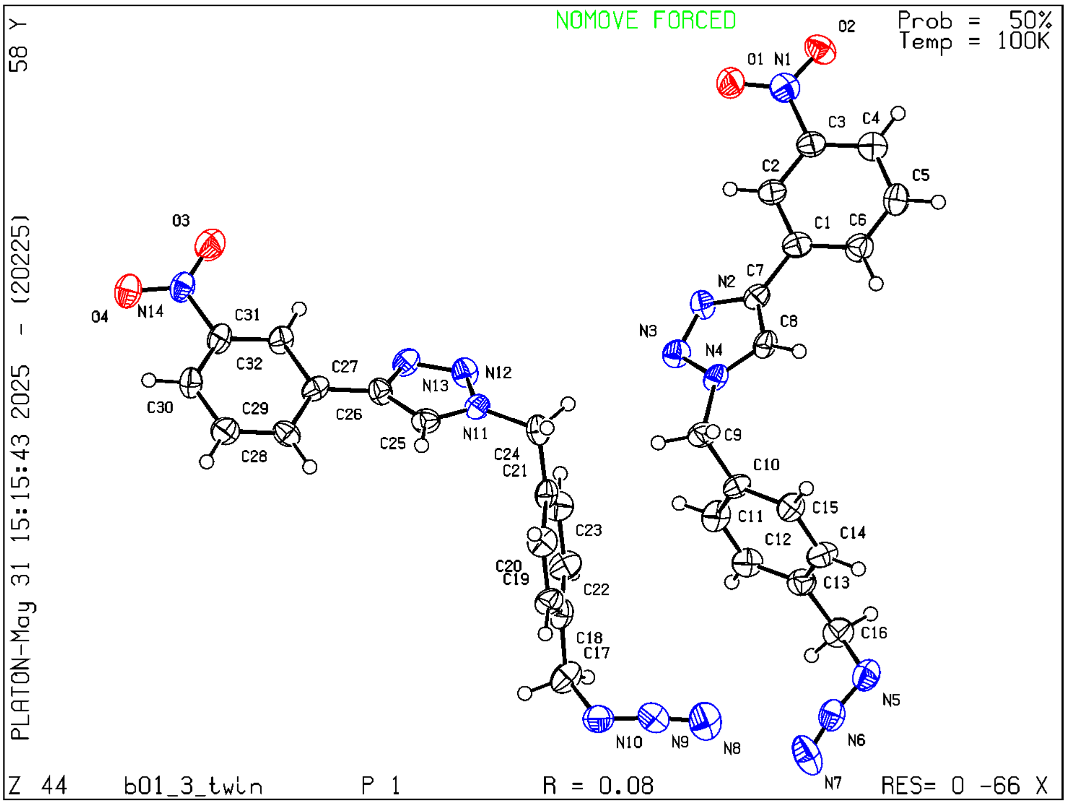

The ellipsoid contour % probability level in the caption for the image of the structure is 50%.

General procedure for preparation of single crystal of compound 6k

6k (20 mg) was dissolved in 3.0 mL of EtOAc in 10 mL of test tube, and then 6 mL of *n*-hexane was added into the test tube. Subsequently, the tube was sealed with sealing film and placed in rt to give **6k** as single crystals in 5 days.

7.2 X-Ray Crystallographic Data for 7a

X-Ray crystallographic data for compound **7a** was recorded at 296 K on a Rigaku Oxford Diffraction Supernova Dual Source, Cu at Zero equipped with an AtlasS2 CCD using Cu Kα radiation. Data reduction was carried out with the diffractometer software. The structures were solved by direct methods using Olex2 software[45] and the non-hydrogen atoms were located from

the trial structure and then refined anisotropically with SHELXL-2014[46] using a full-matrix least squares procedure based on F2. The weighted R factor, wR and goodness-of-fit S values were obtained based on F2. The hydrogen atom positions were fixed geometrically at the calculated distances and allowed to ride on their parent atoms. Crystallographic data for the structure reported in this paper have been deposited at the Cambridge Crystallographic Data Center and allocated with the deposition numbers: 2472583.

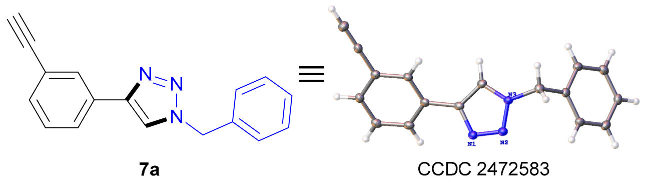

Figure S2. ORTEP drawing of **7a**

Crystal data and structure refinement for 7a.

Identification code	**7a**
Empirical formula	$C_{17}H_{13}N_3$
Formula weight	259.30
Temperature/K	100.00(10)
Crystal system	monoclinic
Space group	$P2_1/c$
a/Å	28.9361(9)
b/Å	5.7791(2)
c/Å	7.7851(2)
α/°	90
β/°	93.208(3)
γ/°	90
Volume/Å^3	1299.82(7)
Z	4
ρ_{calc}g/cm^3	1.325
μ/mm^{-1}	0.633
F(000)	544.0
Crystal size/mm^3	0.15 × 0.13 × 0.12
Radiation	Cu Kα (λ = 1.54184)
2Θ range for data collection/°	9.184 to 149.682
Index ranges	$-35 \leq h \leq 35, -4 \leq k \leq 6, -9 \leq l \leq 9$
Reflections collected	5521
Independent reflections	2505 [R_{int} = 0.0306, R_{sigma} = 0.0206]
Data/restraints/parameters	2505/0/181
Goodness-of-fit on F^2	1.134
Final R indexes [I>=2σ (I)]	R_1 = 0.0636, wR_2 = 0.1719
Final R indexes [all data]	R_1 = 0.0649, wR_2 = 0.1729
Largest diff. peak/hole / e Å^{-3}	0.29/-0.34

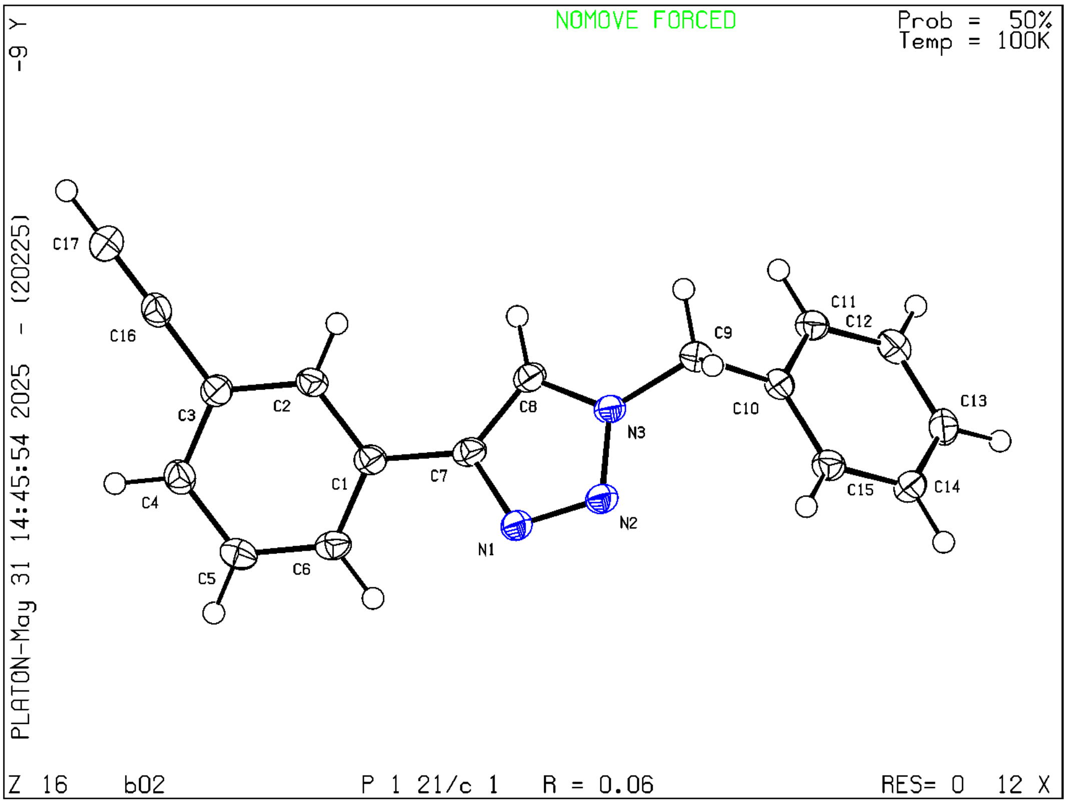

The ellipsoid contour % probability level in the caption for the image of the structure is 50%.

General procedure for preparation of single crystal of compound 7a

7a (20 mg) was dissolved in 3.0 mL of EtOAc in 10 mL of test tube, and then 6 mL of *n*-hexane was added into the test tube. Subsequently, the tube was sealed with sealing film and placed in rt to give **7a** as single crystals in 5 days.

8. References

[1] C. A. Franco, T. I. Silva, M. G. Dias, B. W. Ferreira, B. L. Sousa, G. M. Bousada, R. W. Barreto, B. G. Vaz, G. S. Lima, M. H. Santos, J. A. S. Grossi and E. V. V. Varejão, *J. Agric. Food Chem.*, **2022**, *70*, 2806.

[2] Â. M. A. lima, L. C. Moreira, P. R. Gazolla, M. B. Oliveira, R. R. Teixeira, V. T. Queiroz, M. R. Rocha, W. B. Moraes, N. A. Santos, W. Romaõ, V. L. Jr., P. A. B. Morais, O. V. Oliveira, W. C. J. Juńior, L. C. A. Barbosa, C. J. Nascimento, J. Junker and A. V. Costa, *J. Agric. Food Chem.*, **2024**, *72*, 12459.

[3] L.-F. Peng, Y.-T. Zhao, Y. Okuda, L.-Y. Le, Z.-L. Tang, S.-F. Yin, R.-H. Qiu and A. Orita, *J. Org. Chem.*, **2023**, *88*, 3089.

[4] B. Peng, A.-G. Thorsell, T. Karlberg, H. Schüler and S. Q. Yao. *Angew. Chem. Int. Ed.* **2017**, *56*, 248.

[5] E. J. O′Neil, K. M. DiVittorio and B. D. Smith, *Org. Lett.* **2007**, *9*, 199.

[6] E. Tasca, G. L. Sorella, L. Sperni, G. Strukul and A. Scarso, *Green Chem.*, **2015**, *17*, 1414.

[7] L. Luciani, E. Goff, D. Lanari, S. Santoroa and L. Vaccaro. *Green Chem.*, **2018**, *20*, 183.

[8] R. K. Pathak, J. Dessingou, V. K. Hinge, A. G. Thawari, S. K. Basu and C. P. Rao, *Anal. Chem.*, **2013**, *85*, 3707.

[9] K. Staffan, C. Calum, E. Hans, F. Kenny, G. Paul and M. Mubina, *Org. Process Res. Dev.*, **2017**, *21*, 1668.

[10] Y.-H. Ju, D. Kumar and R. S. Varma, *J. Org. Chem.*, **2006**, *71*, 6697.

[11] D. Rodríguez-Hernández, L. C.A. Barbosa, A. J. Demuner, A. Nain-Perez, S. R. Ferreira, R. T. Fujiwara, R. M. Almeida, L. Heller and R. Csuk, *Eur. J. Med. Chem.*, **2017**, *140*, 624.

[12] V. Camberlein, N. Kraupner, N. B. Karroum, E.e Lipka, R. Deprez-Poulain, B. Deprez and D. Bosc, *Tetrahedron Lett.*, **2021**, *73*, 153131.

[13] Z.-X. Fang, Y.-M. Gong, B.-B. Liu, J. Zhang, X.-Y. Han and Z.-H. Liu, *Org. Lett.*, **2022**, *24*, 8920.

[14] H.-M. Cheng, J.-T. Wan, M.-I. Lin, Y.-X. Liu, X.-Y. Lu, J.-S. Liu, Y. Xu, J.-X. Chen, Z.-C. Tu, Y.-S. E. Cheng and K. Ding. *J. Med. Chem.*, **2012**, *55*, 2144.

[15] S.-H. H. Orcid, H. K. Orcid, H. Ryu, I. E. S. Orcid and D. Lee, *J. Am. Chem. Soc.*, **2022**, *144*, 1778.

[16] É. Godin, Je. Santandrea, A. Caron and S. K. Collins, *Org. Lett.*, **2020**, *22*, 5905.

[17] H. S. G. Beckmann and V. Wittmann, *Org. Lett.*, **2007**, *9*, 1.

[18] K. Yamaguchi, T. Oishi, T. Katayama and N. Mizuno, *Chem. Eur. J.*, **2009**, *15*, 10464.

[19] S.-H. Sun, R.-X. Bai and Y.-L. Gu, *Chem. Eur. J.*, **2014**, *20*, 549.

[20] C. Zhang, B. Huang, Y. Chen and D.-M. Cui, *New J. Chem.*, **2013**, *37*, 2606.

[21] G.-F. Liu, Z.-W. Li, Z.-J. Huang, Z.-Y. Zhou, Y.-X. Li, A.-L. Huang, Z.-Y. Cai, G.-F. Ouyang, B.-H. Ye, Y.-B. Zhang, *J. Am. Chem. Soc.*, **2025**, *147*, 1840.

[22] G. A. Chesnokov, M. A. Topchiy, P. B. Dzhevakov, P. S. Gribanov, A. A. Tukov, V. N. Khrustalev, A. F. Asachenko and M. S. Nechaev, *Dalton Trans.*, **2017**, *46*, 4331.

[23] F. Friscourt and G.-J. Boons, *Org. Lett.*, **2010**, *12*, 4936.

[24] W. Abdelgayed, A. Arafa and A.-A. A. Nayl, *Appl. Organometal. Chem.*, **2019**, *33*, e5156.

[25] P. A. Scattergood, A. M. Ranieri, L. Charalambou, A. Comia, D. A. W. Ross, D. A. W. Ross, C. R. Rice, S. J. O. Hardman, J.-L. Heully, I. M. Dixon, M. Massi, F. Alary and P. I. P. Elliott, *Inorg. Chem.*, **2020**, *59*, 1785.

[26] J. Doiron, A. H. Soultan, R, Richard, M. M. Touré, N. Picot, R. Richard, M. Čuperlović-Culf, G. A. Robichaud and M. Touaibia, *Eur. J. Med. Chem.*, 2011, 46, 4010.

[27] A. Makarem, R. Berg, F. Rominger and B. F. Straub, *Angew. Chem. Int. Ed.*, **2015**, *54*, 7431.

[28] M. Liu and O. Reiser, *Org. Lett.*, **2011**, *13*, 1102.

[29] P. Gogoi, H. Deka, B. Das, K. Deori and D. Sarma, *ACS Sustainable Chem. Eng.*, **2025**, *13*, 936.

[30] M.-Y. Xu, C. Zhao, B.-Y. Zhu, L.-Y. Wang, H.-H. Zhou, D.-G. Yan, Q. Gu and J. Xu, *J. Med. Chem.*, **2021**, *64*, 2010.

[31] J. McNulty and K. Keskar, *Eur. J. Org. Chem.*, **2012**, 5462.

[32] S. B. Ötvös, G. Hatoss, A. Georgiádes, S. Kovács, I. M. Mándity, Z. Novák and F. Fülöp, *RSC Adv.*, **2014**, *4*, 46666.

[33] J.-A. Shin, Y.-G. Lim and K.-H. Lee, *J. Org. Chem.*, **2012**, *77*, 4117.

[34] B. E. Velasco, A. Fuentes, C. Gonzalez, D. Corona, I. Garcia-Orozco and E. Cuevas-Yanez, *Synth. Commun.*, **2011**, *41*, 2966.

[35] L. Bahsis, H. B. E. Ayouchia, H. Anane, A. Pascual-Álvarez, G. D. Munno and S.-E. Stiriba, *Appl. Organometal. Chem.*, **2019**, *33*, e4669.

[36] C.-Z. Tao, X. Cui, J. Li, A.-X. Liu, L. Liu and Q.-X. Guo. *Tetrahedron Lett.*, **2007**, *48*, 3525.

[37] B. Liu, C.-Y. Chen, Y.-J. Zhang, X.-L. Liu and W.-Z. Chen, *Organometallics*, **2013**, *32*, 5451.

[38] S. Ø. Scott, E. L. Gavey, S. J. Lind, K. C. Gordona and J. D. Crowley, *Dalton Trans.*, **2011**, *40*, 12117.

[39] S. Azeez, P. Chaudhary, P. Sureshbabu, S. Sabiahb and J. Kandasamy, *Org. Biomol. Chem.*, **2018**, *16*, 8280.

[40] J.-N. Li, J. Feng, T. Chen, Z.-F. Xu and C.-Y. Li, *Org. Biomol. Chem.*, **2023**, *21*, 5935.

[41] A. M. A. Lima, R. R. Teixeira, W. B. Moraes, M. R. Rocha, A. F. C. Moraes, S. C. Gomes, P. R. Gazolla, S. F. Silva, V. T. Queiroz, V. R. Fonseca, W. Romão, P. A. B. Morais, V. Lacerda, L. M. Abreu, F. M. Oliveira, O. V. Oliveira and A. V. Costa, *J. Agric. Food Chem.*, **2023**, *71*, 6818.

[42] (a) S. M. A. Rahman, J. S. Bhatti, S. Thareja and V. Monga, *Eur. J. Med. Chem.*, **2023**, *259*, 115699; (b) I. Seck, I. Ciss, A. Diédhiou, M. Baldé, S. Ka, L. A. Ba, S. F. Ndoye, B. Figadère, B. Seon-Meniel, G. Gomez, S. Cojean, S. Pomel, P. M. Loiseau, Y. Fall and M. Seck, *Med. Chem. Res.*, **2023**, *32*, 158.

[43] H. Bertrand, M. Schaap, L. Baird, N. D. Georgakopoulos, A. Fowkes, C. Thiollier, H. Kachi, A. T. Dinkova-Kostova and G. Wells, *J. Med. Chem.*, **2015**, *58*, 7186.

[44] M. H. Presa, M. J. Rocha, C. S. Pires, K. N. B. Ledebuhr, G. P. Costa, D. Alves, C. F. Bortolatto, C. A. Brüning, *ACS Chem. Neurosci.*, **2023**, 14, 2333.

[45] O. V. Dolomanov, L. J. Bourhis, R. J. Gildea, J. A. K. Howard, H. Puschmann, *J. Appl. Cryst.* **2009**, *42*, 339.

[46] D. Kratzert, I. Krossing, J. J. Holstein, *J. Appl. Cryst.* **2015**, *48*, 933.

[47] F. Neese, *WIREs Comput. Mol. Sci.* **2022**; *12*: e1606.

[48] P. J. Stephens, F. J. Devlin, C. F. Chabalowski, M. J. Frisch, *J. phys. Chem*. **1994**, *98*, 11623.

[49] S. Grimme, S. Ehrlich, L. Goerigk, *J. Comput. Chem.* **2011**, *32*, 1456.

[50] F. Weigend, R. Ahlrichs, *Phys. Chem. Chem. Phys.* **2005**, *7*, 3297.

[51] F. Weigend, *Phys. Chem. Chem. Phys.* **2006**, *8*, 1057.

[52] K. Fukui, *Acc. Chem. Res*. **1981**, *14*, 363.

[53] K. Fukui, *J. Phys.Chem.* **1970**, *74*, 4161.

9. NMR Copies of All Compounds

^{1}H NMR (400 MHz, $CDCl_3$) spectrum for L1

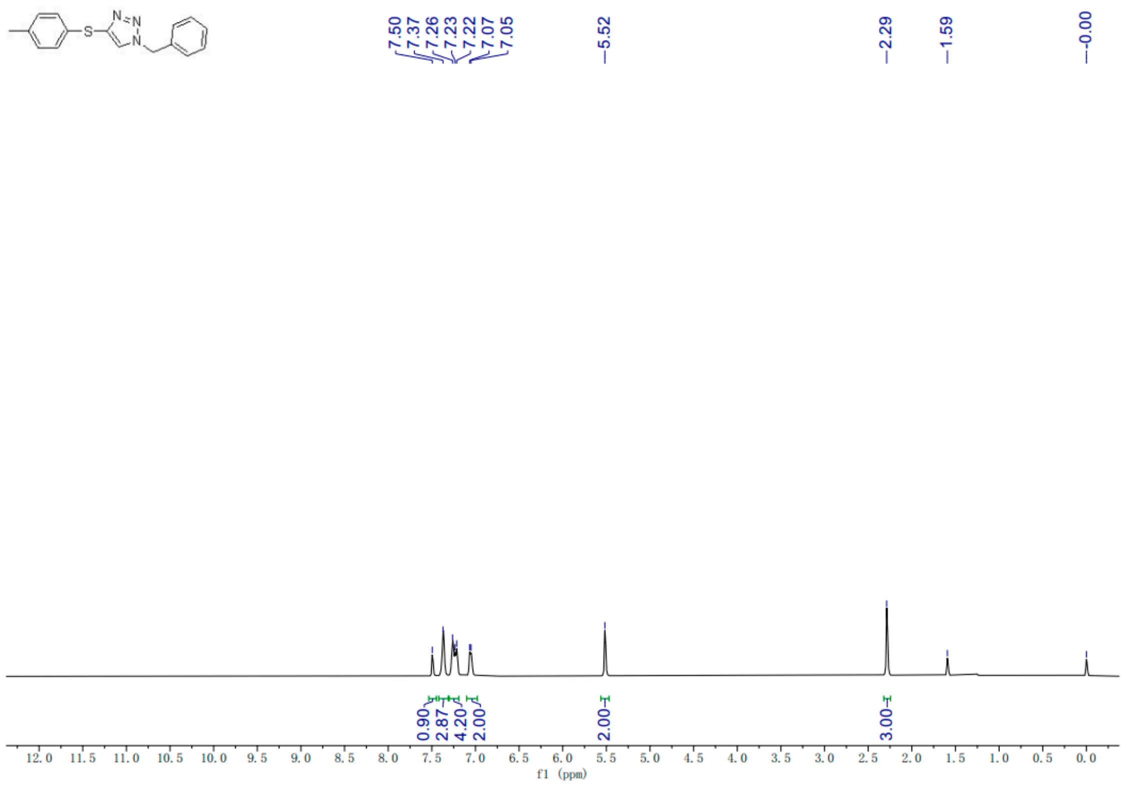

^{1}H NMR (400 MHz, $CDCl_3$) spectrum for L2

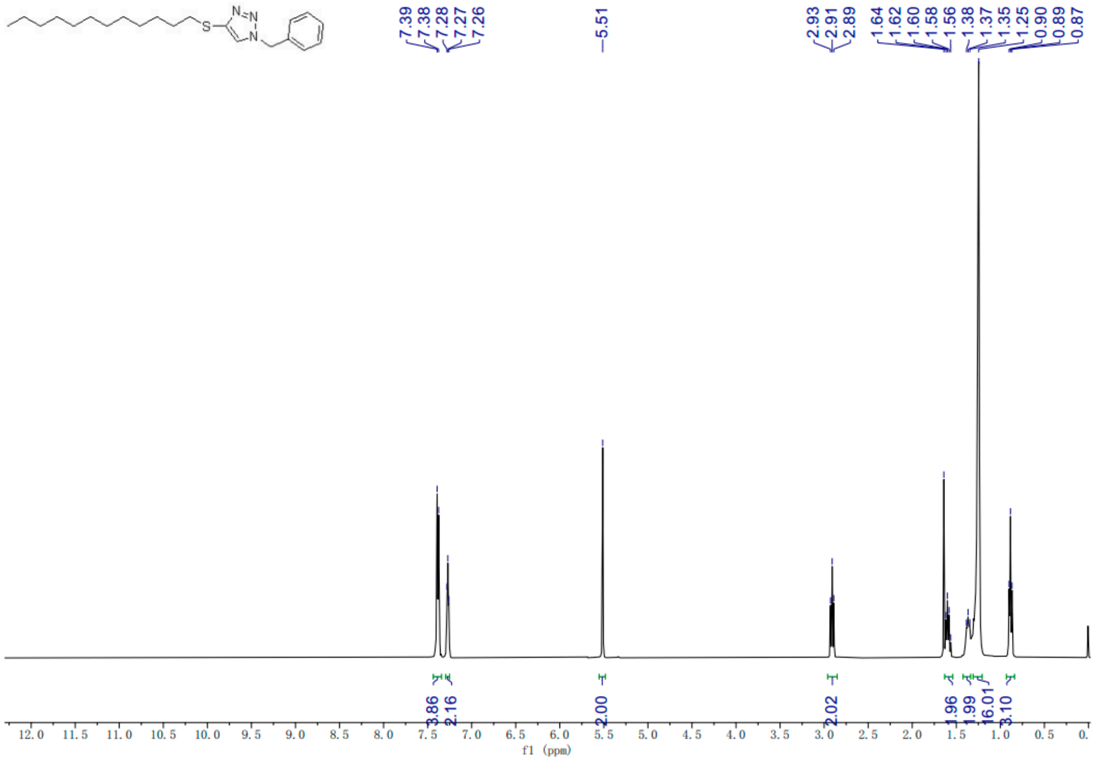

^{1}H NMR (400 MHz, $CDCl_3$) spectrum for L3

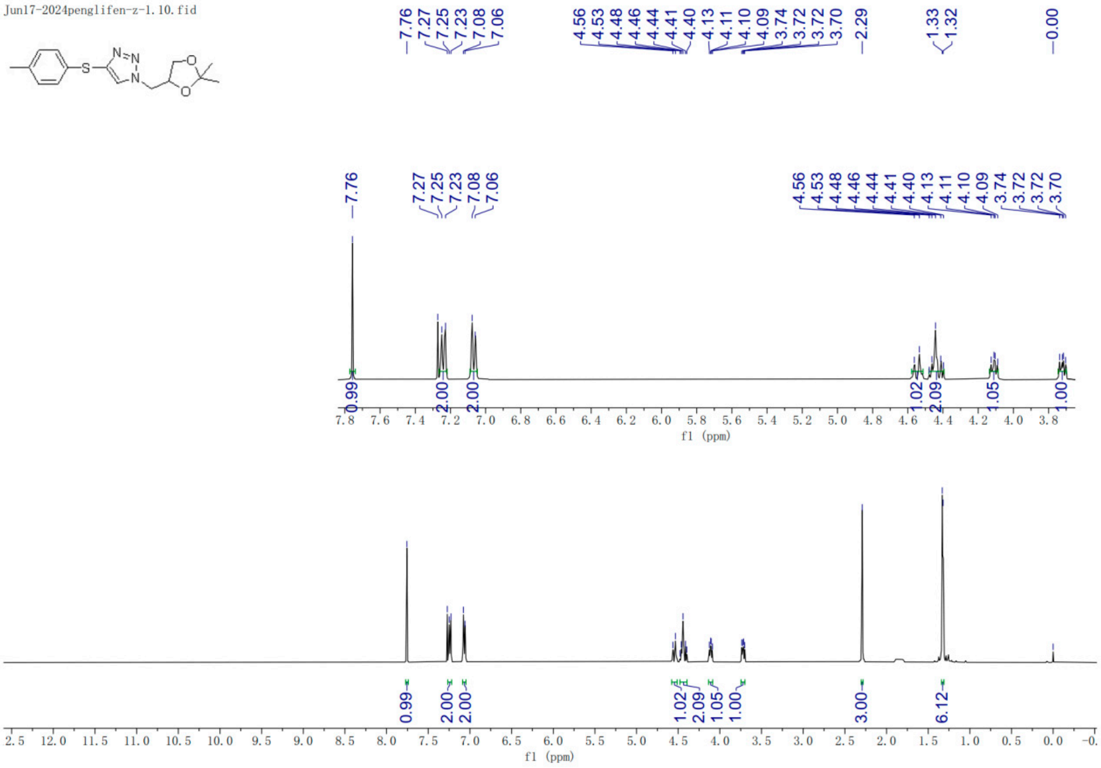

^{13}C NMR (101 MHz, CDCl$_3$) spectrum for L3

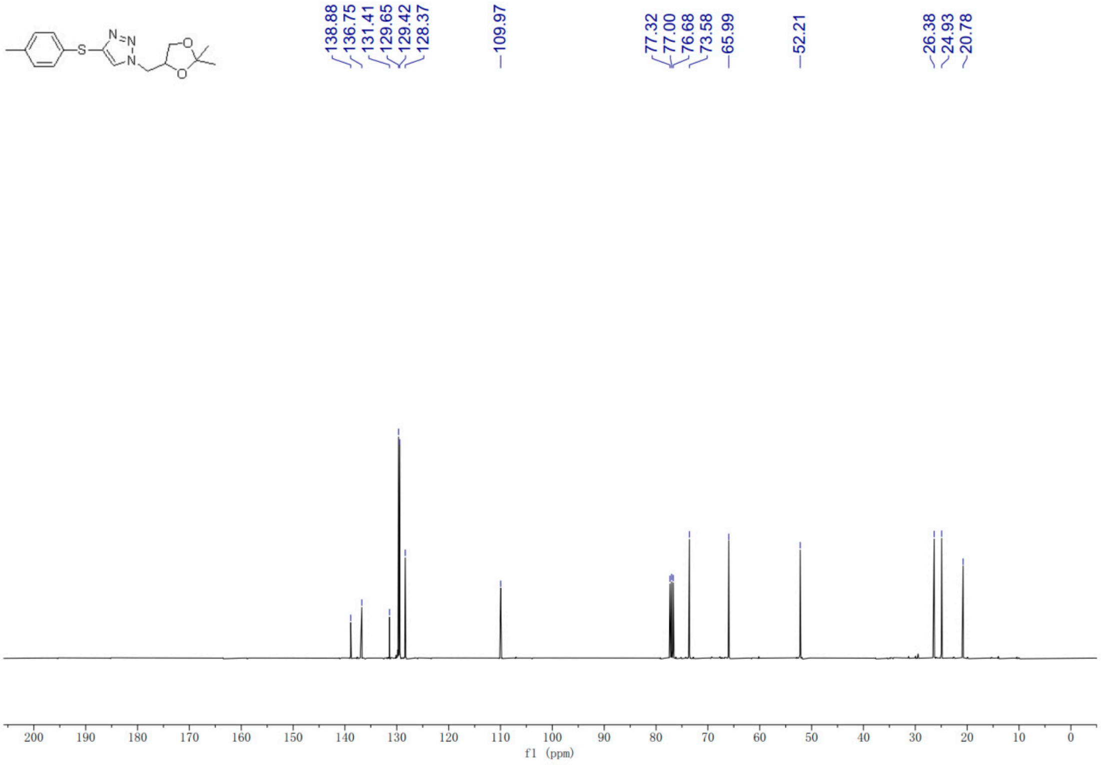

^{1}H NMR (400 MHz, CDCl$_3$) spectrum for L4

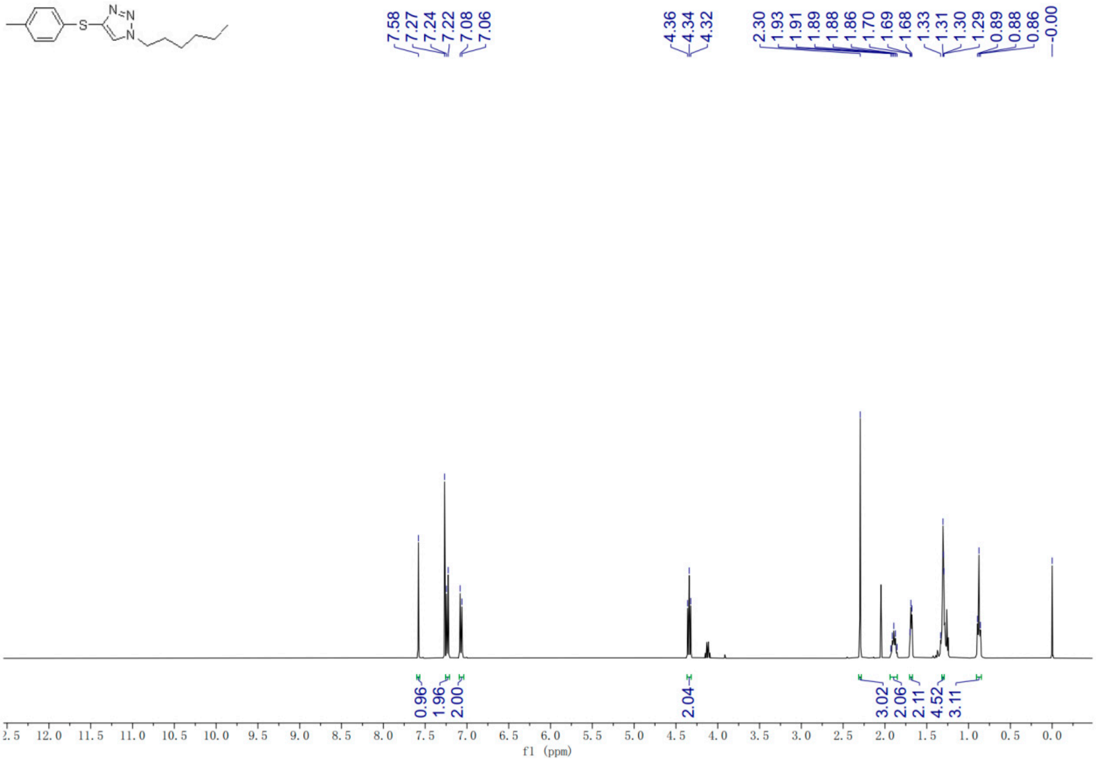

¹H NMR (400 MHz, CDCl$_3$) spectrum for L5

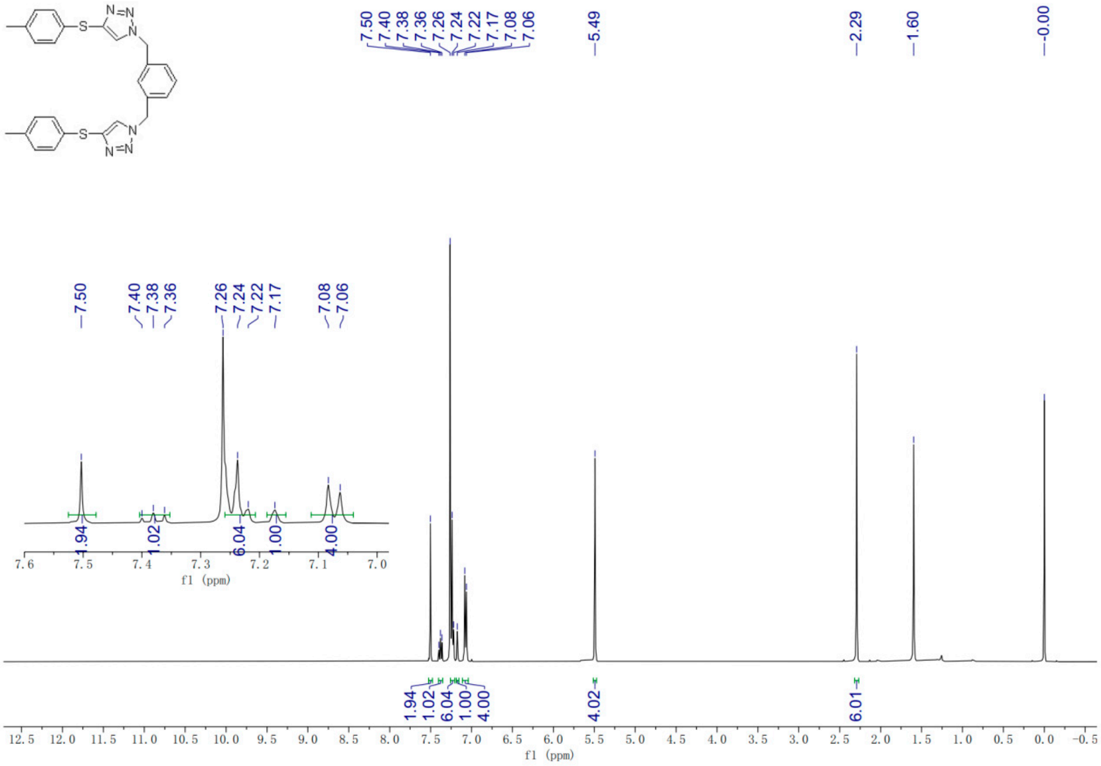

¹³C NMR (101 MHz, CDCl$_3$) spectrum for L5

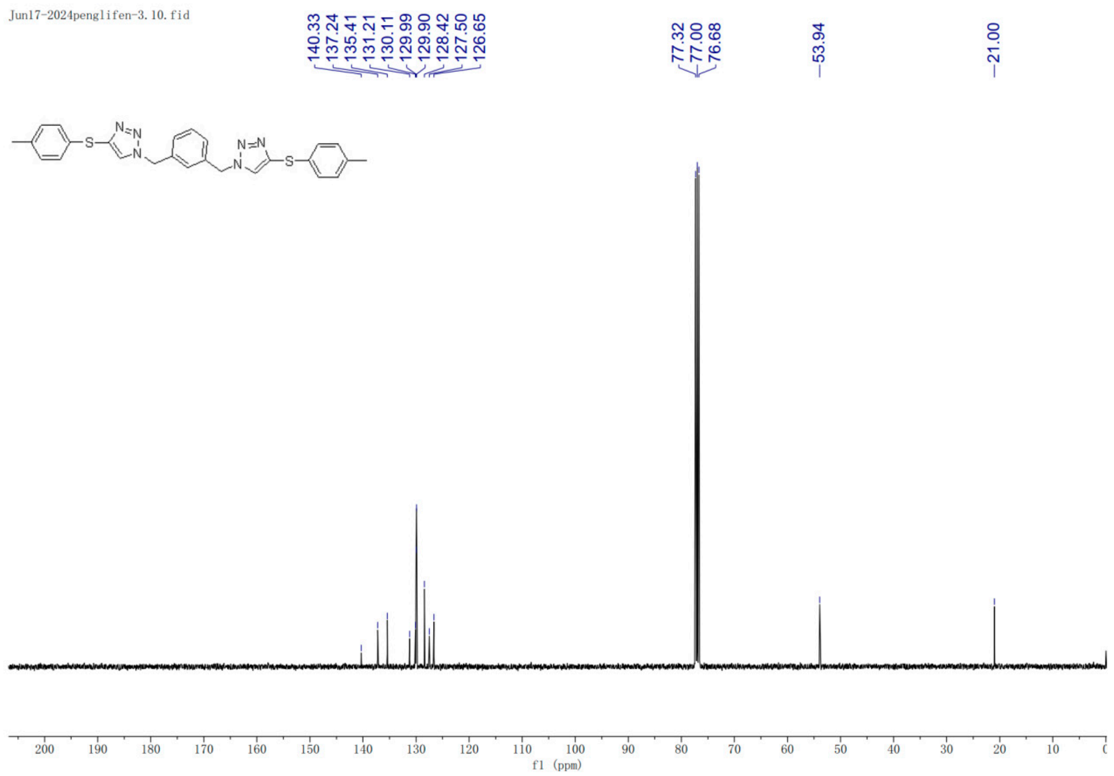

^{1}H NMR (400 MHz, CDCl$_3$) spectrum for 5a

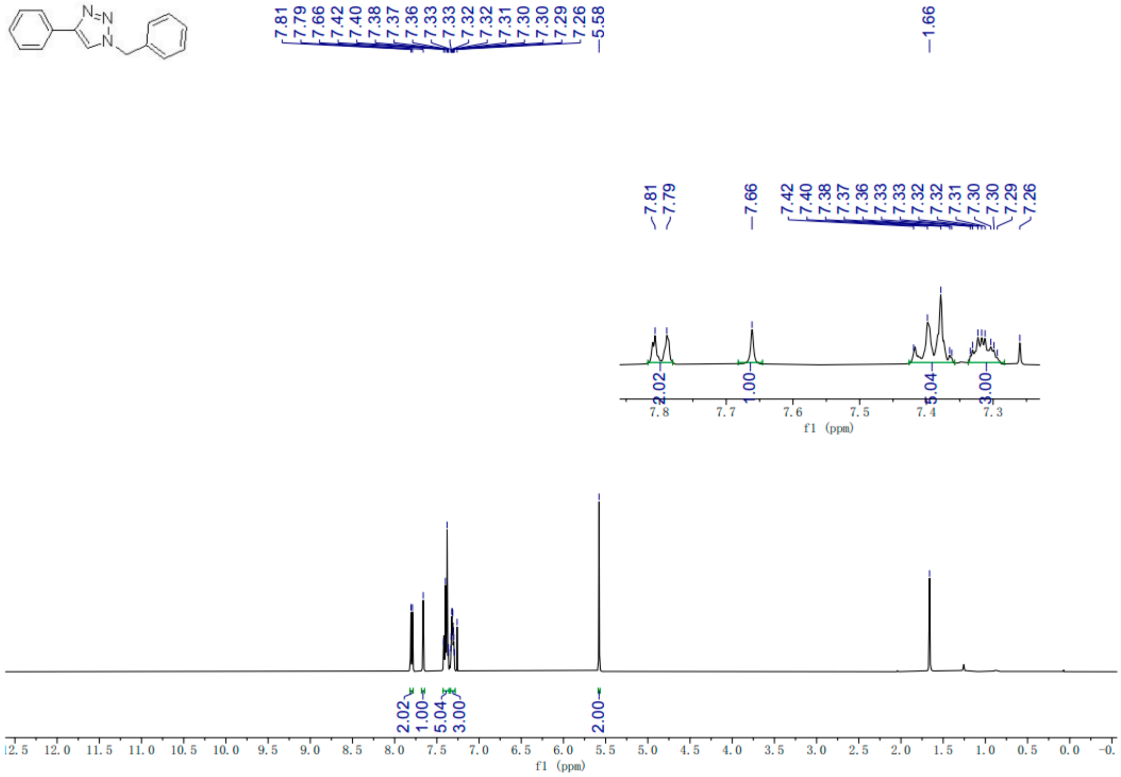

^{1}H NMR (400 MHz, CDCl$_3$) spectrum for 5b

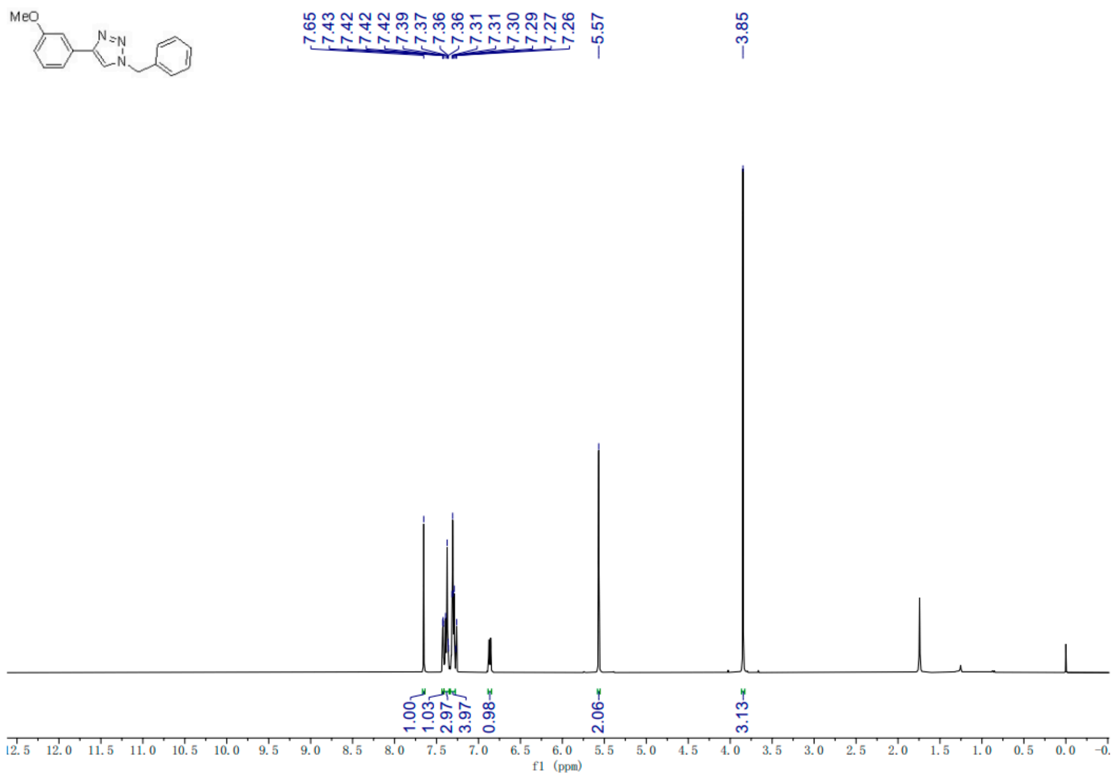

^{1}H NMR (400 MHz, $CDCl_3$) spectrum for 5c

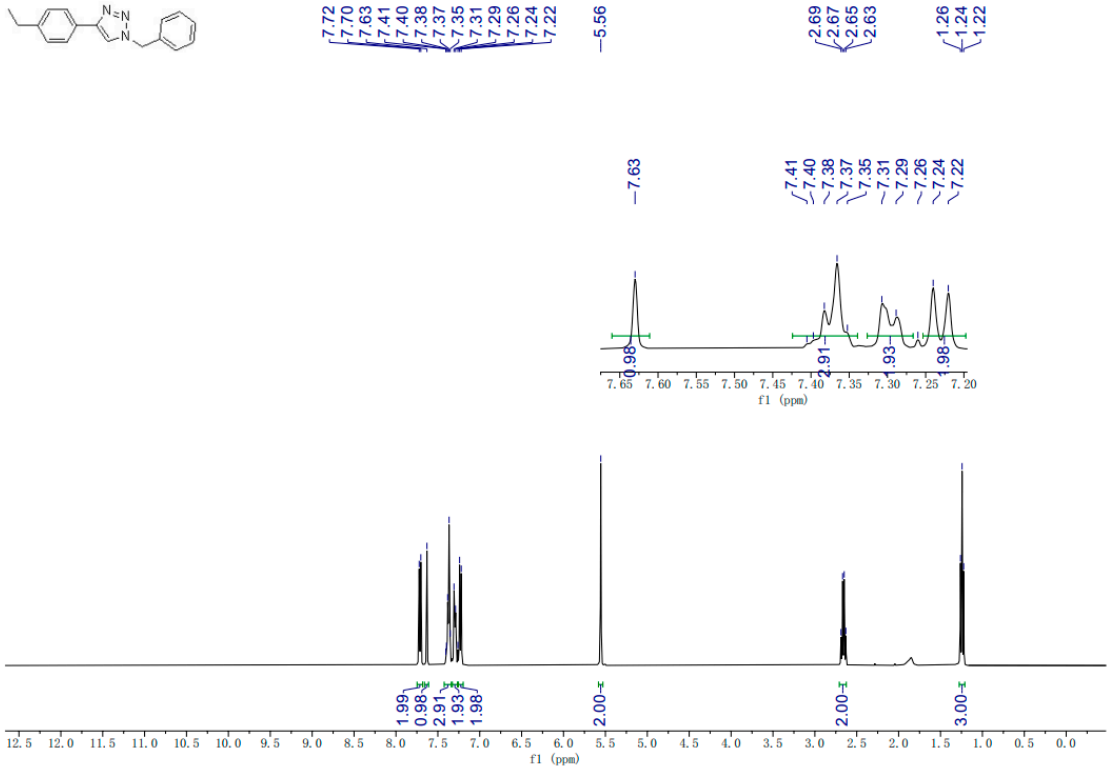

^{1}H NMR (400 MHz, $CDCl_3$) spectrum for 5d

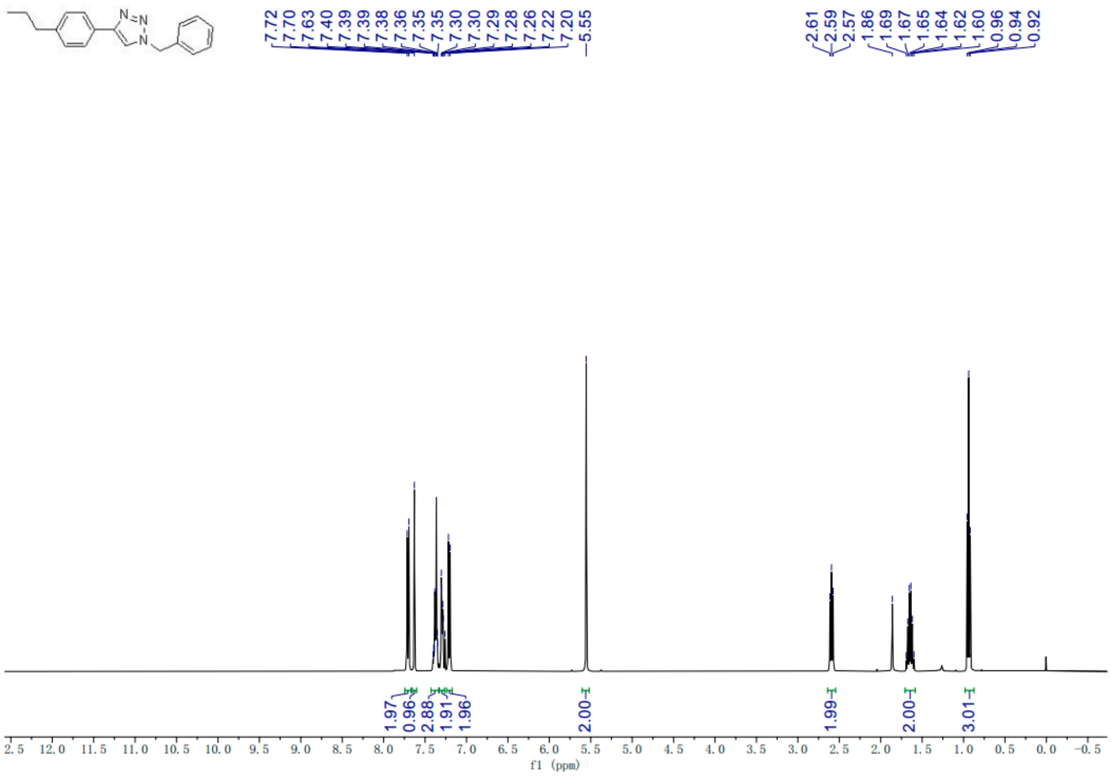

^{1}H NMR (400 MHz, $CDCl_3$) spectrum for 5e

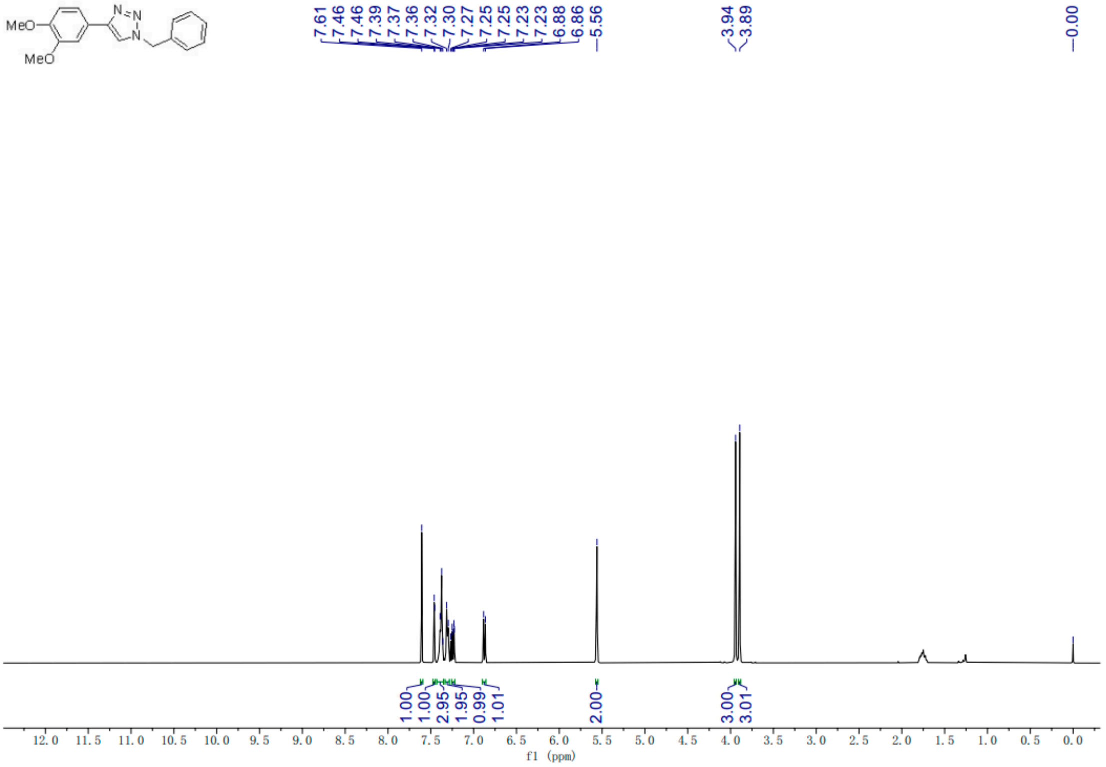

^{1}H NMR (400 MHz, $CDCl_3$) spectrum for 5f

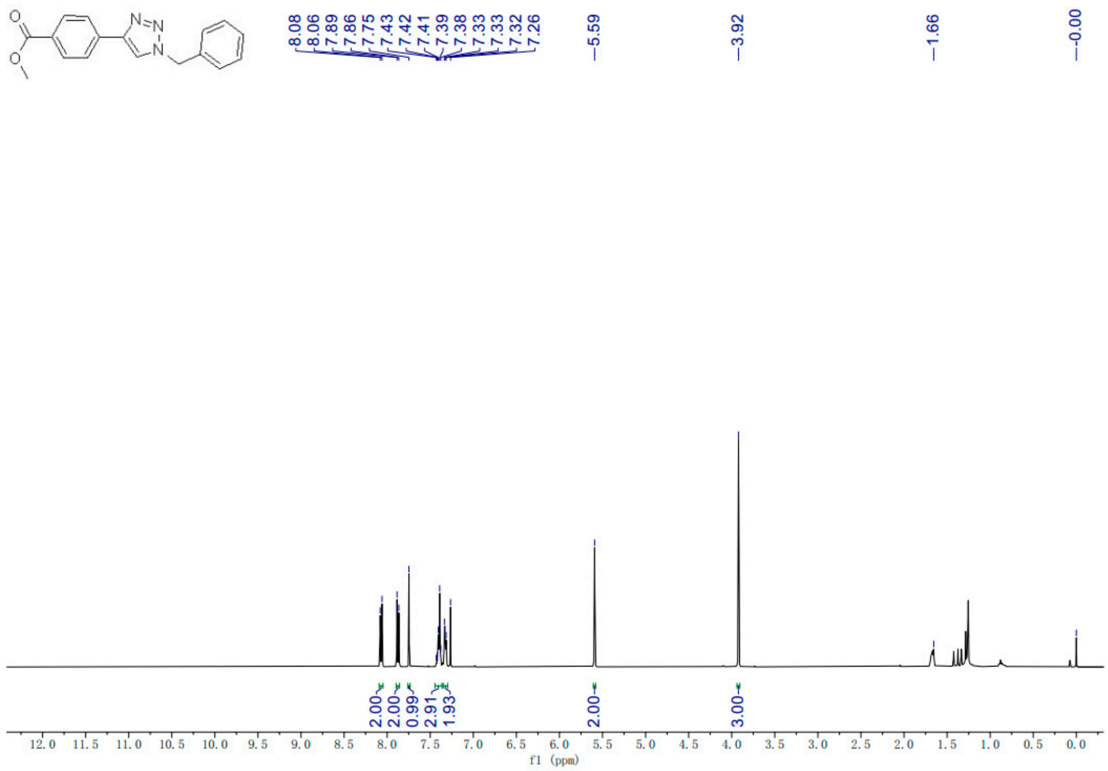

^{1}H NMR (400 MHz, $CDCl_3$) spectrum for 5g

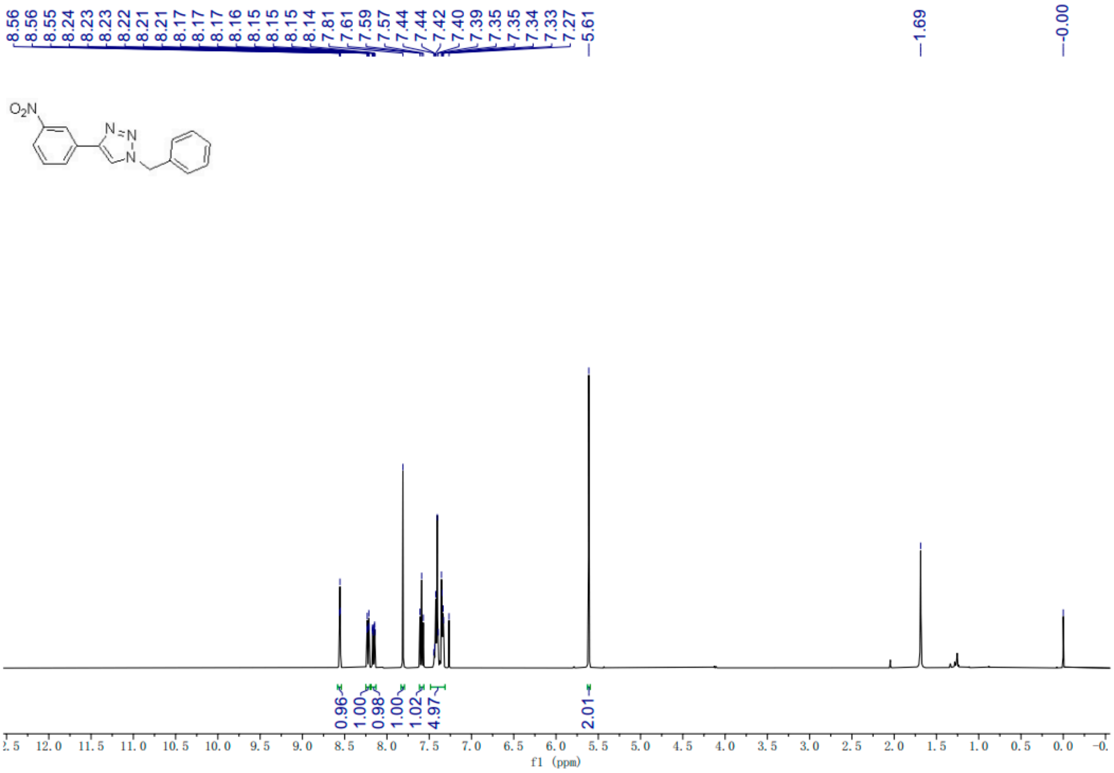

^{1}H NMR (400 MHz, $CDCl_3$) spectrum for 5h

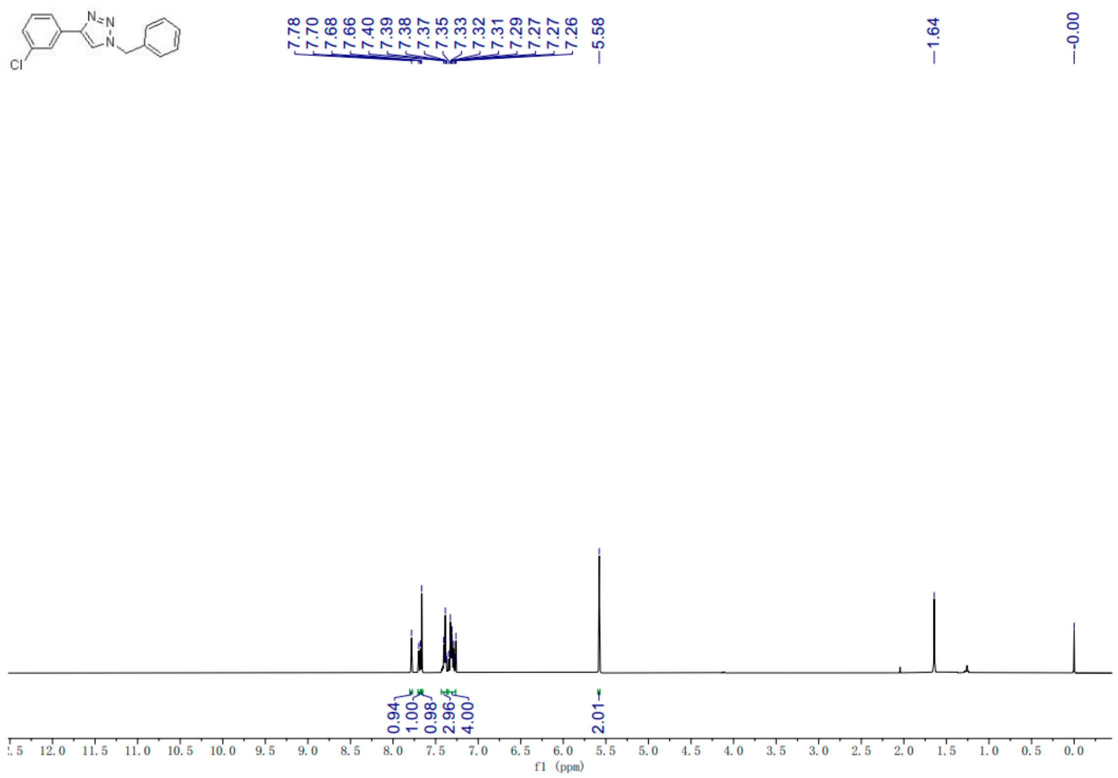

^{1}H NMR (400 MHz, CDCl$_3$) spectrum for 5i

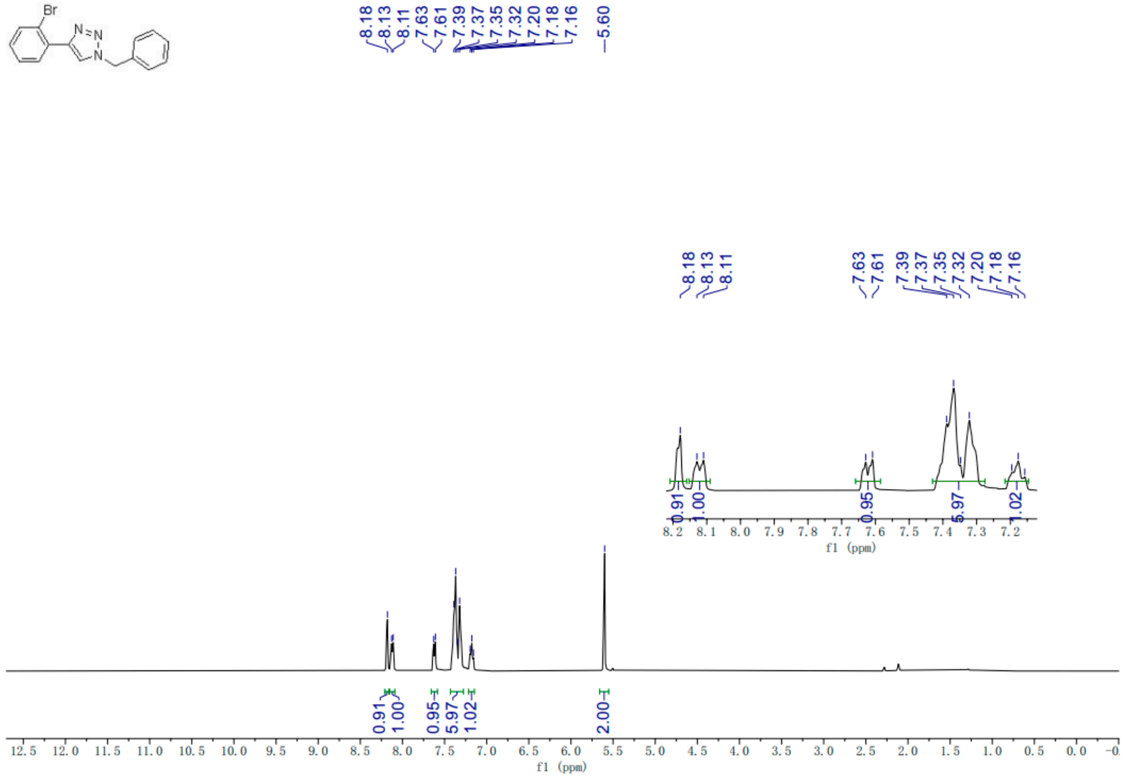

^{1}H NMR (400 MHz, CDCl$_3$) spectrum for 5j

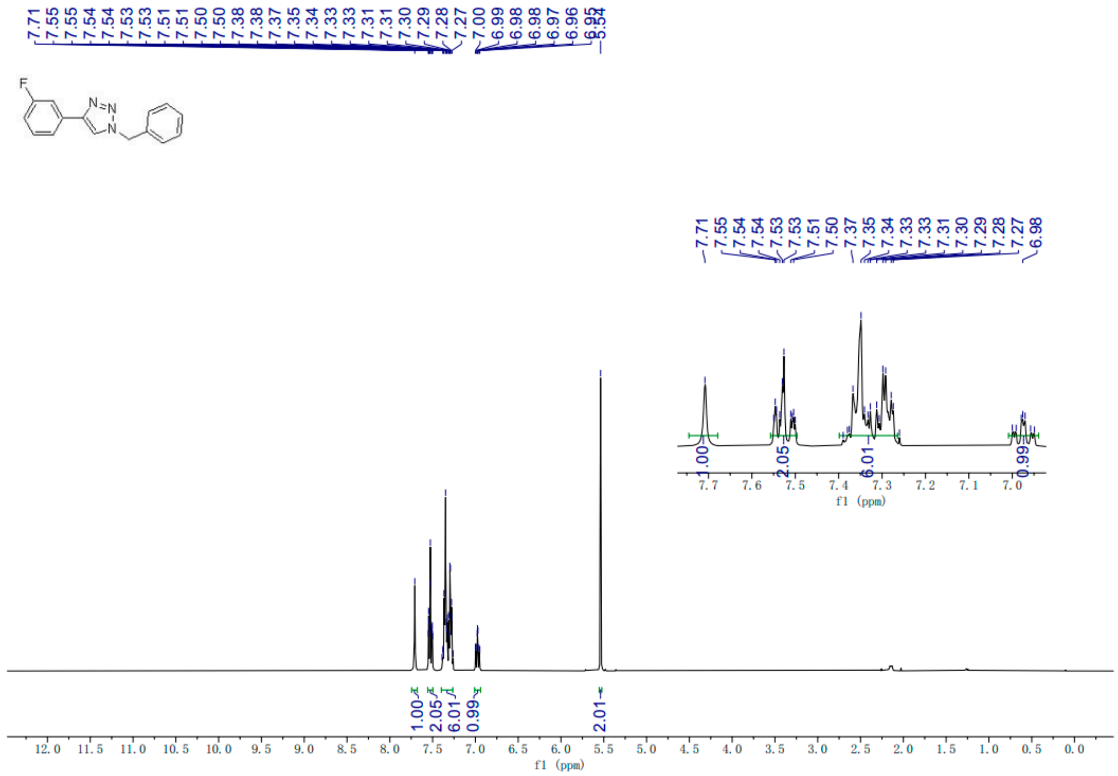

^{1}H NMR (400 MHz, $CDCl_3$) spectrum for 5k

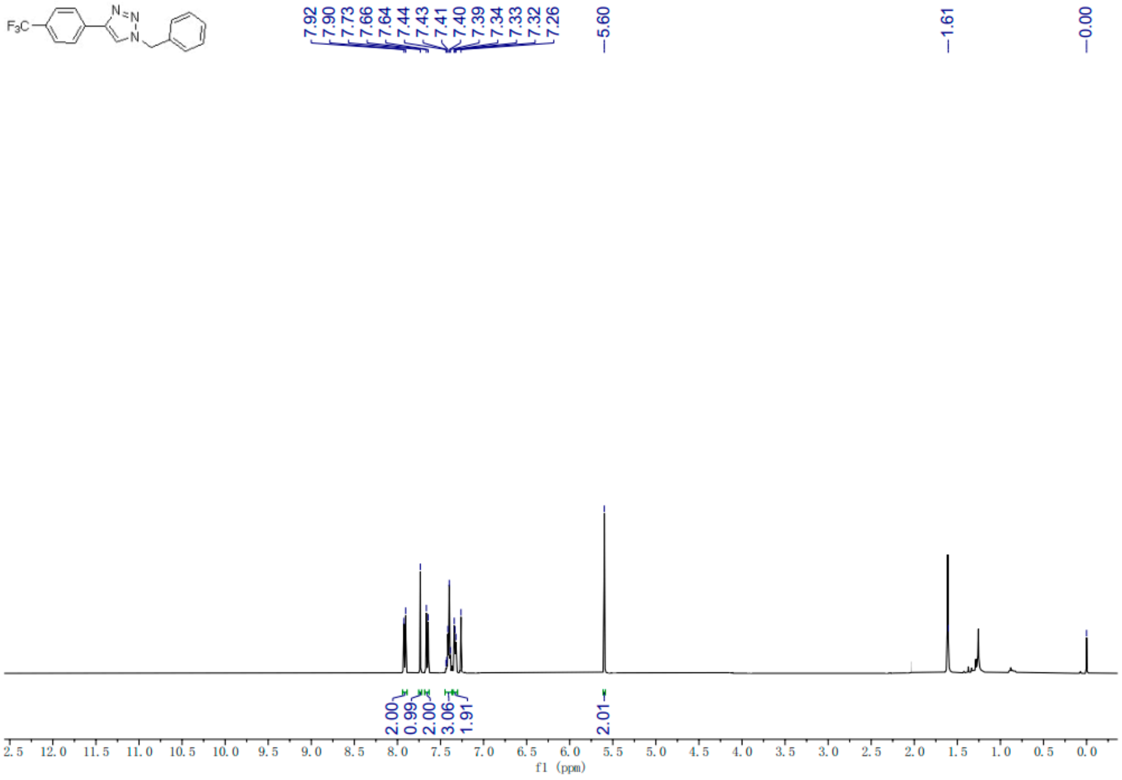

^{1}H NMR (400 MHz, $CDCl_3$) spectrum for 5l

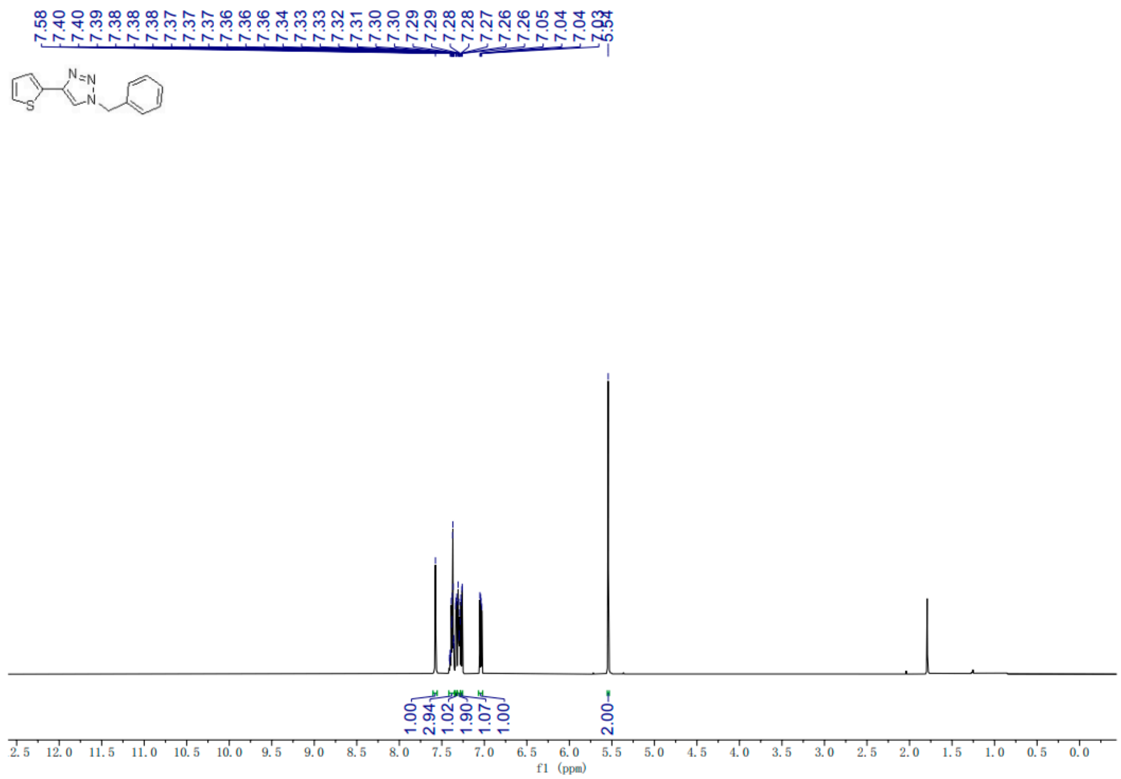

^{1}H NMR (400 MHz, $CDCl_3$) spectrum for 5m

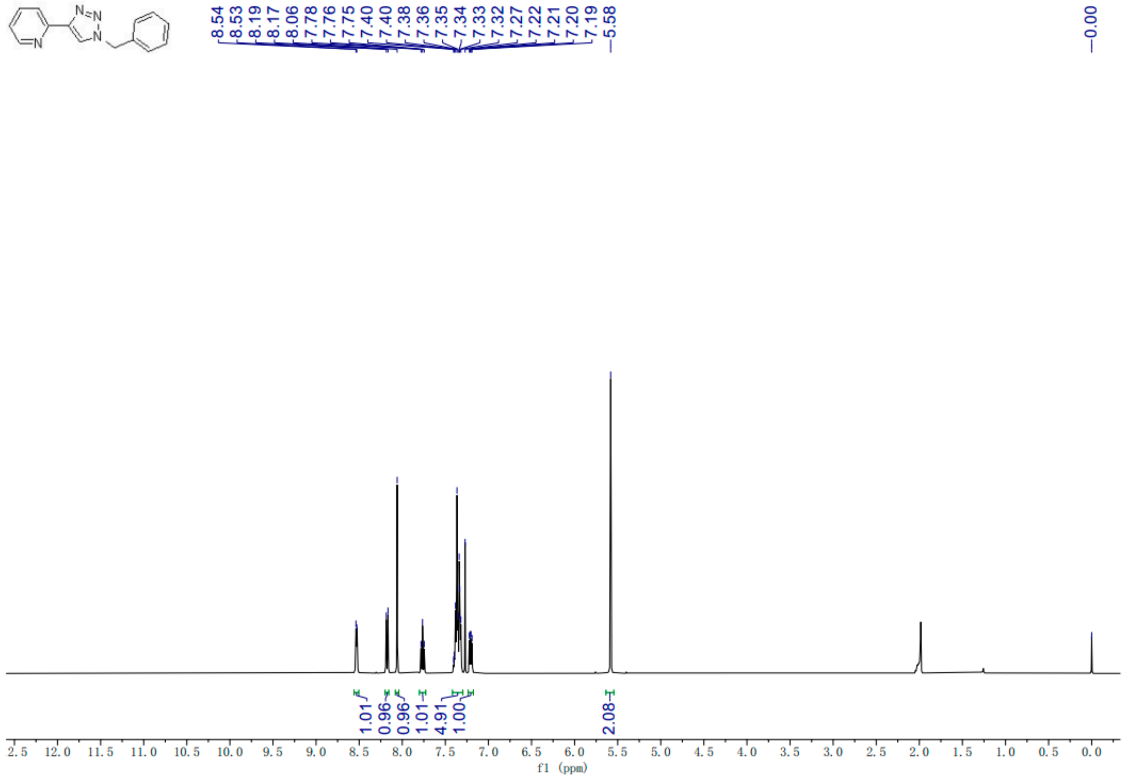

^{1}H NMR (400 MHz, $CDCl_3$) spectrum for 5n

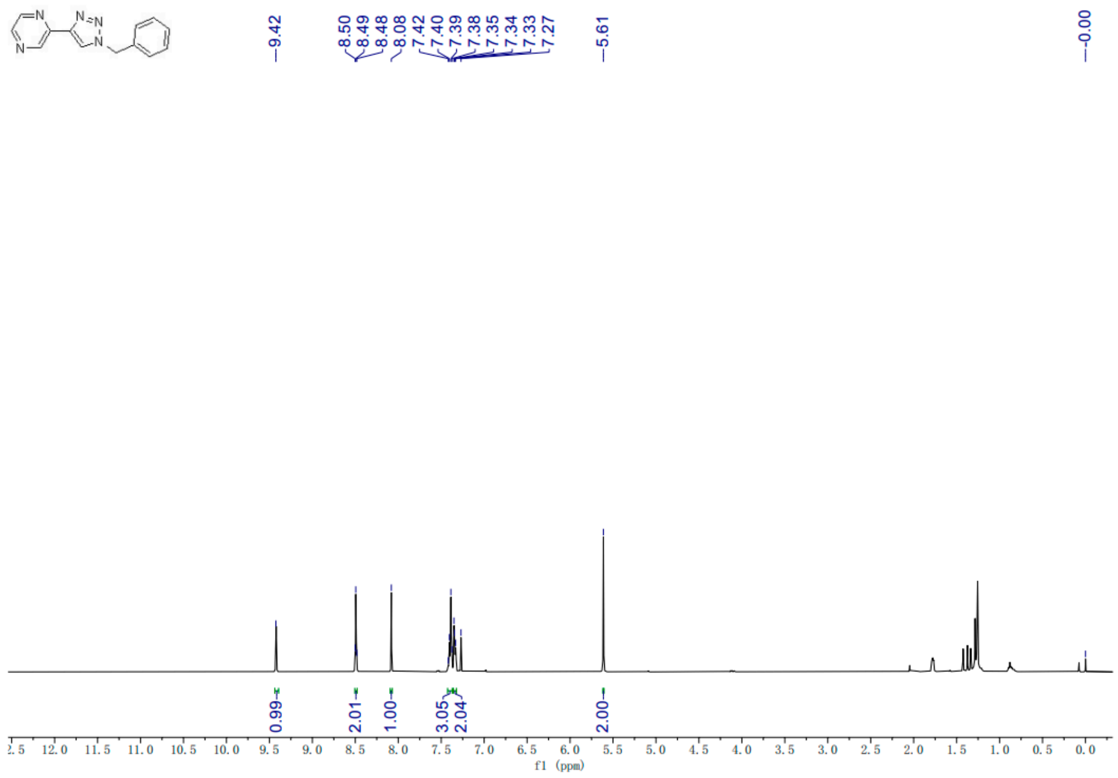

^{1}H NMR (400 MHz, CDCl$_3$) spectrum for 5o

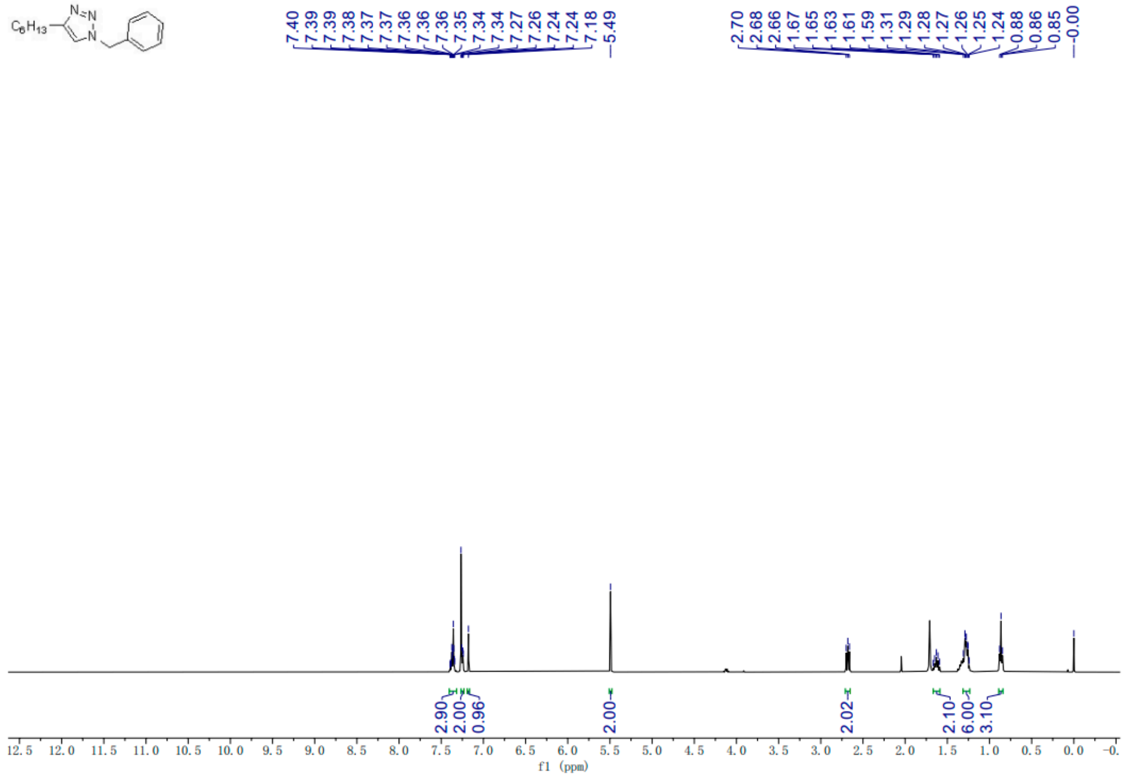

^{1}H NMR (400 MHz, CDCl$_3$) spectrum for 5p

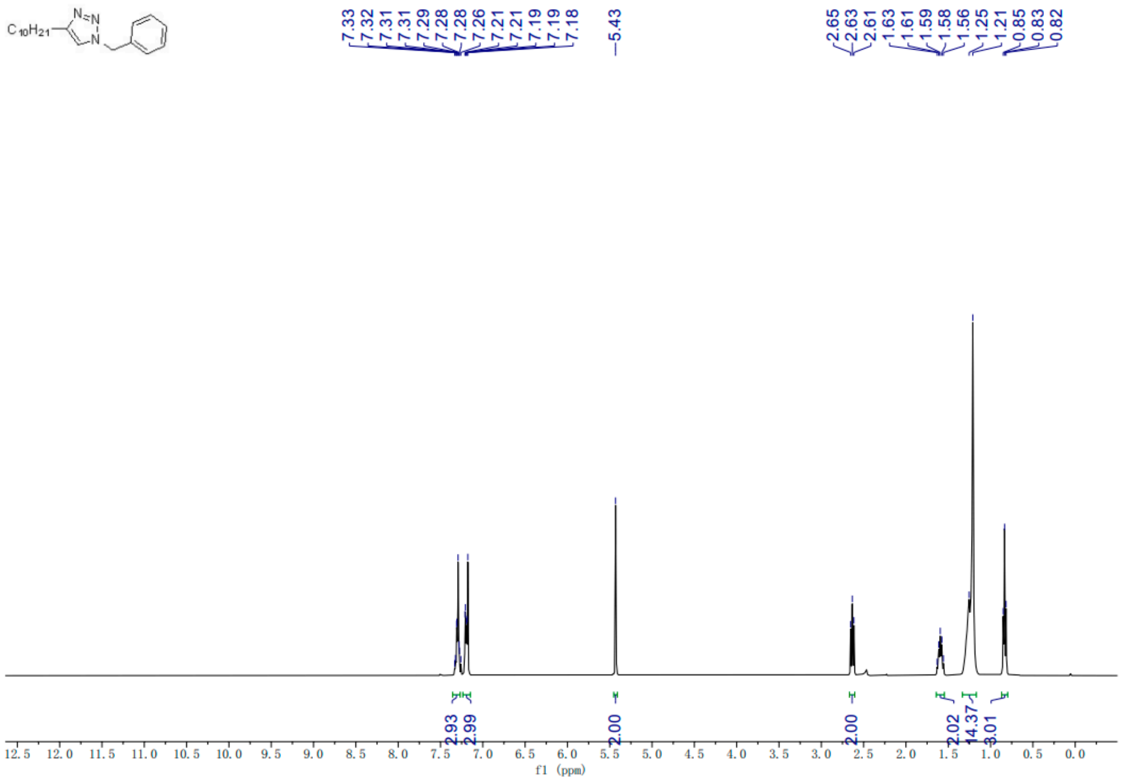

^{1}H NMR (400 MHz, CDCl$_3$) spectrum for 5q

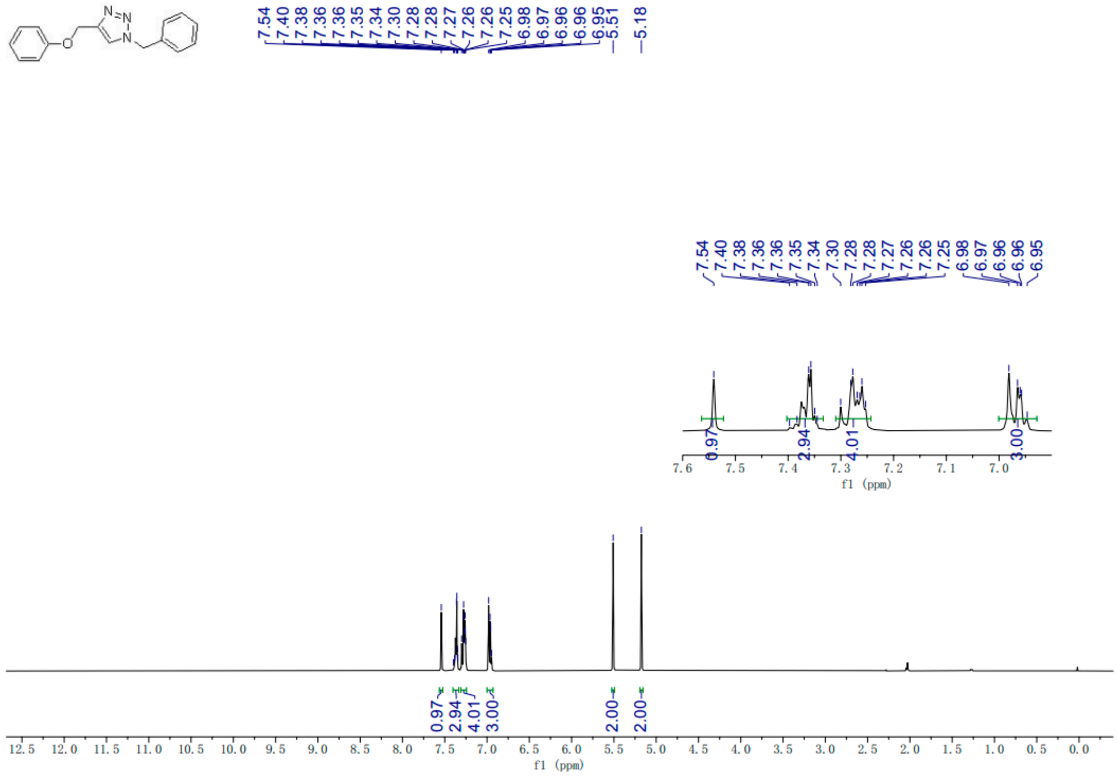

^{1}H NMR (400 MHz, CDCl$_3$) spectrum for 5r

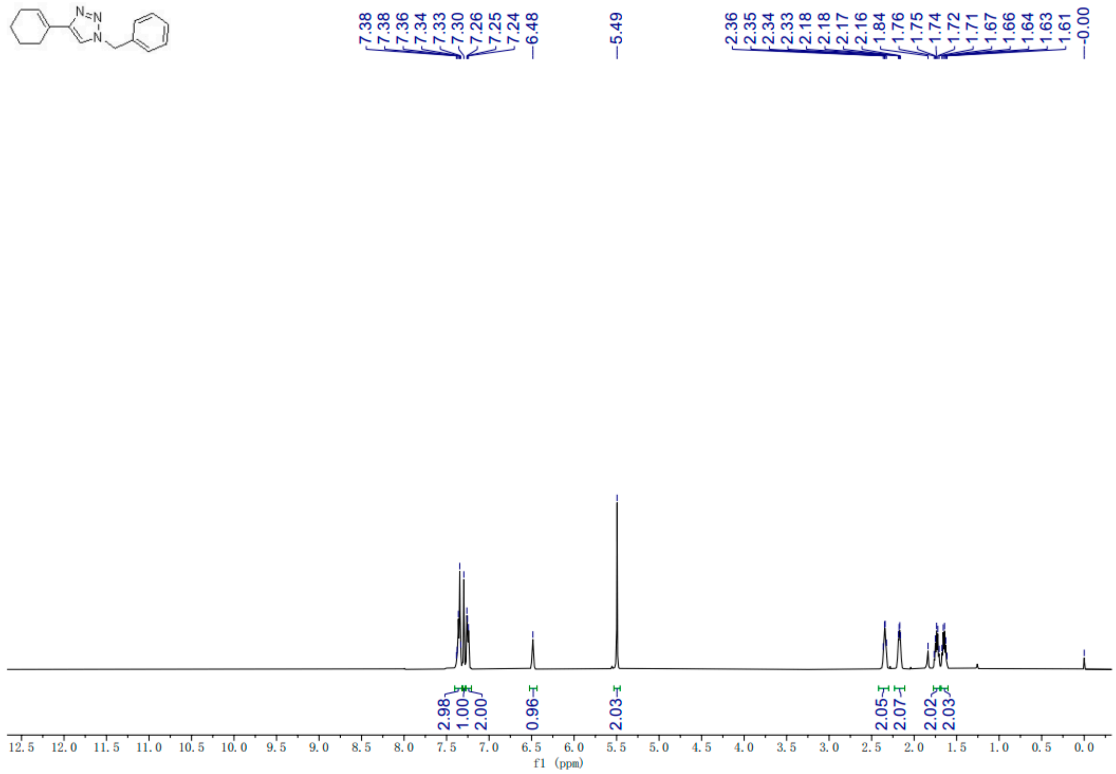

^{1}H NMR (400 MHz, CDCl$_3$) spectrum for 5s

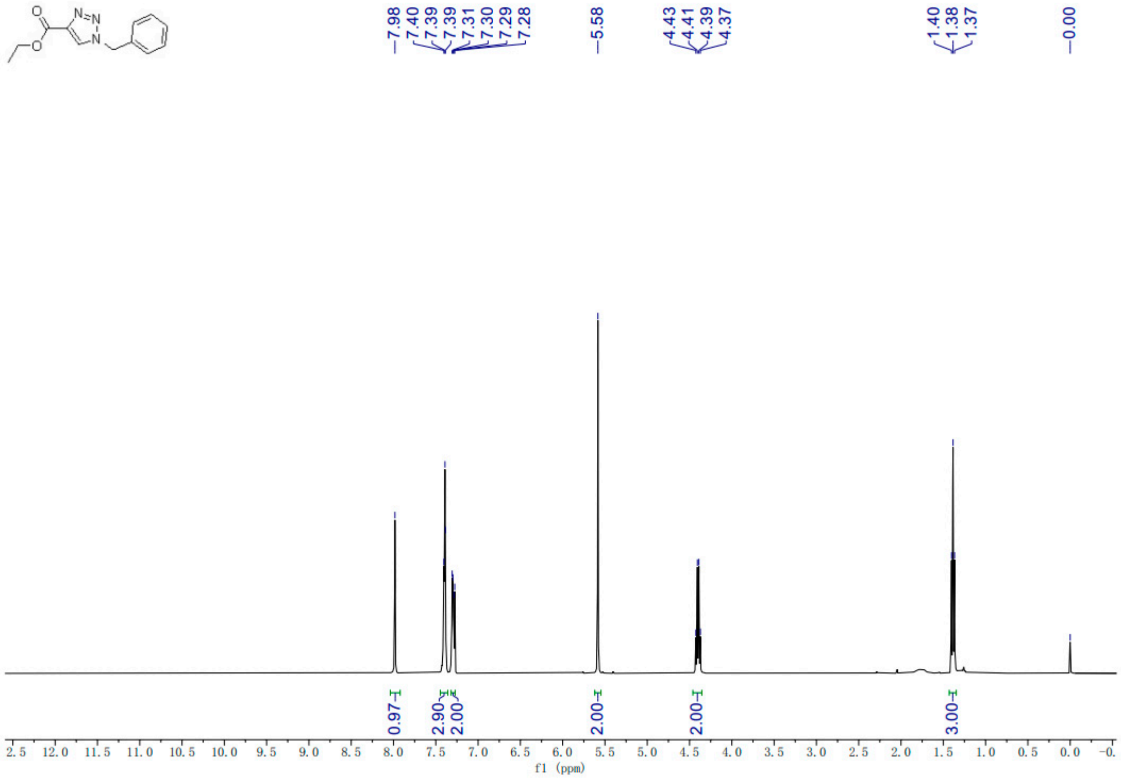

^{1}H NMR (400 MHz, CDCl$_3$) spectrum for 5t

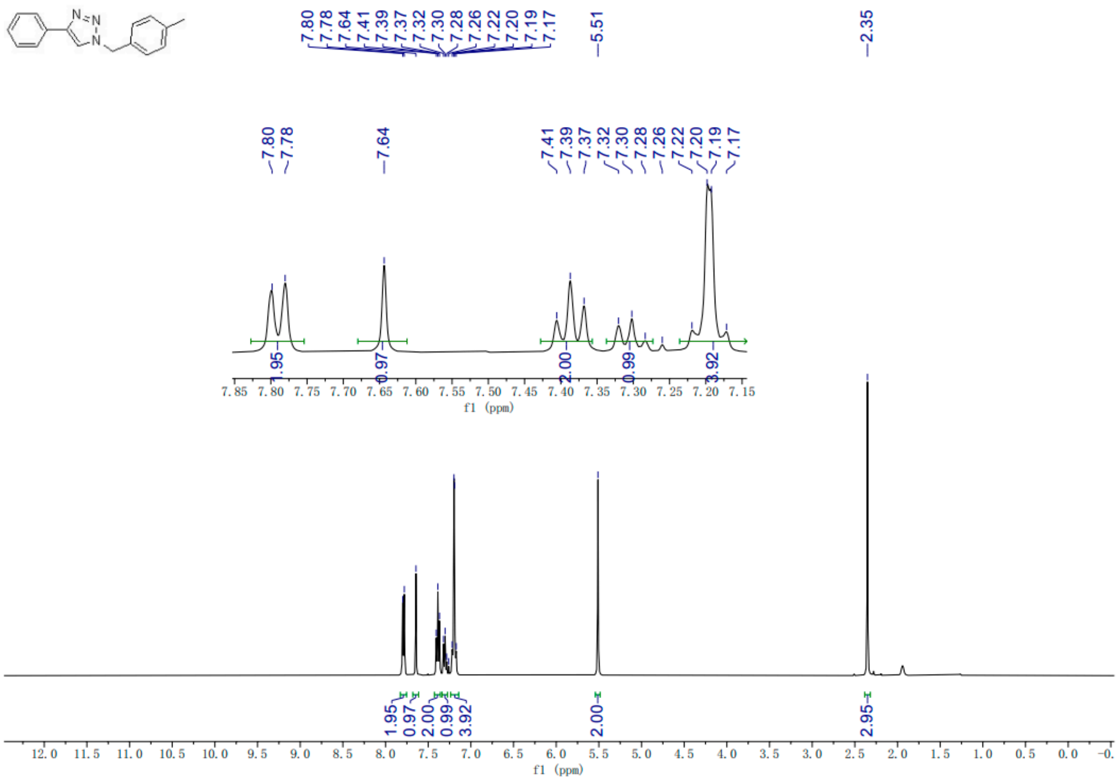

^{1}H NMR (400 MHz, $CDCl_3$) spectrum for 5u

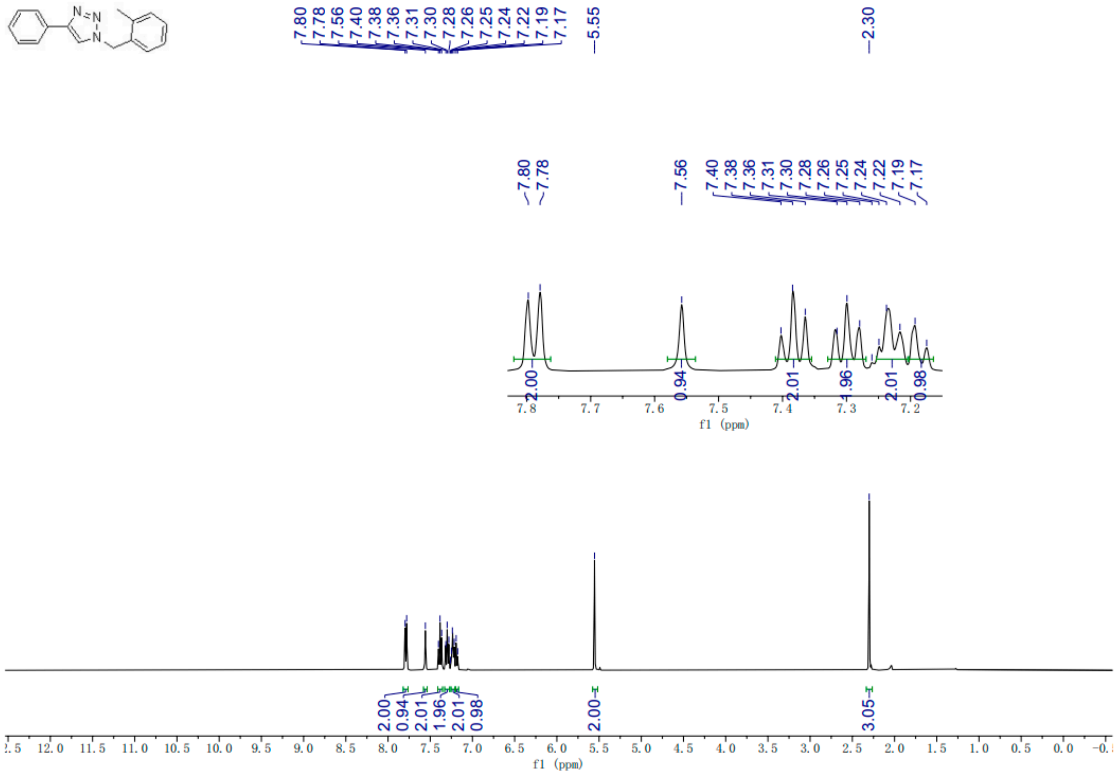

^{1}H NMR (400 MHz, $CDCl_3$) spectrum for 5v

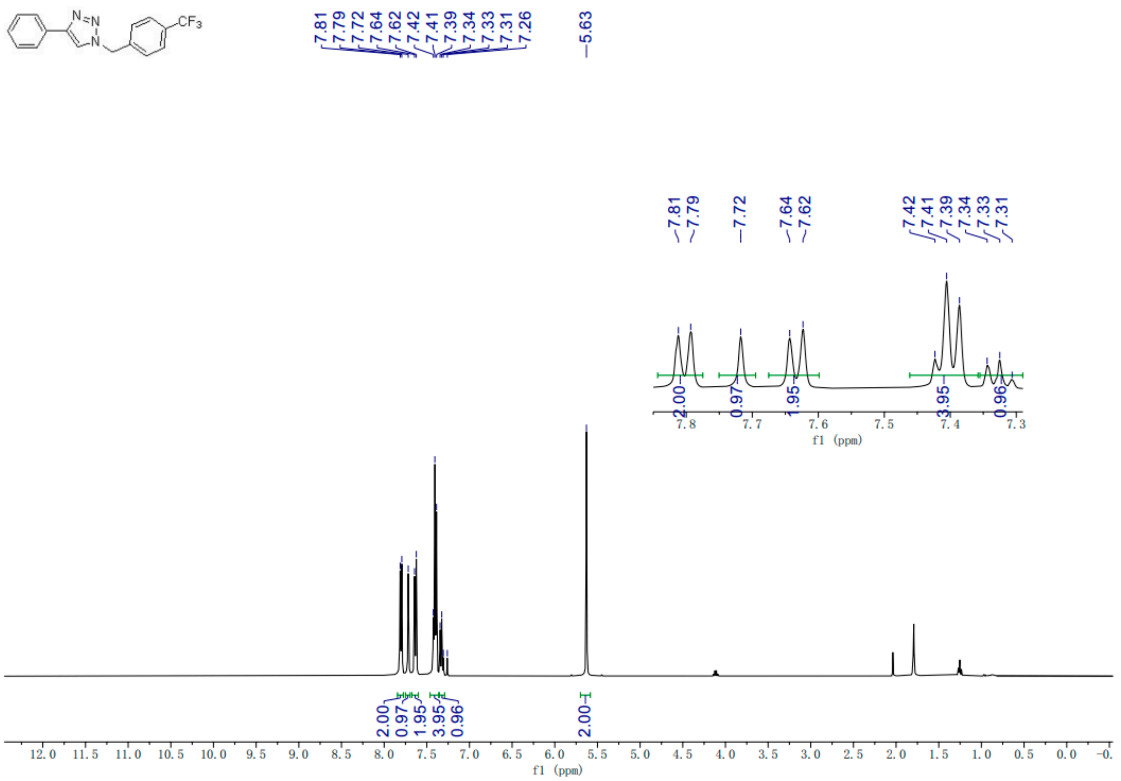

^{1}H NMR (400 MHz, CDCl$_3$) spectrum for 5w

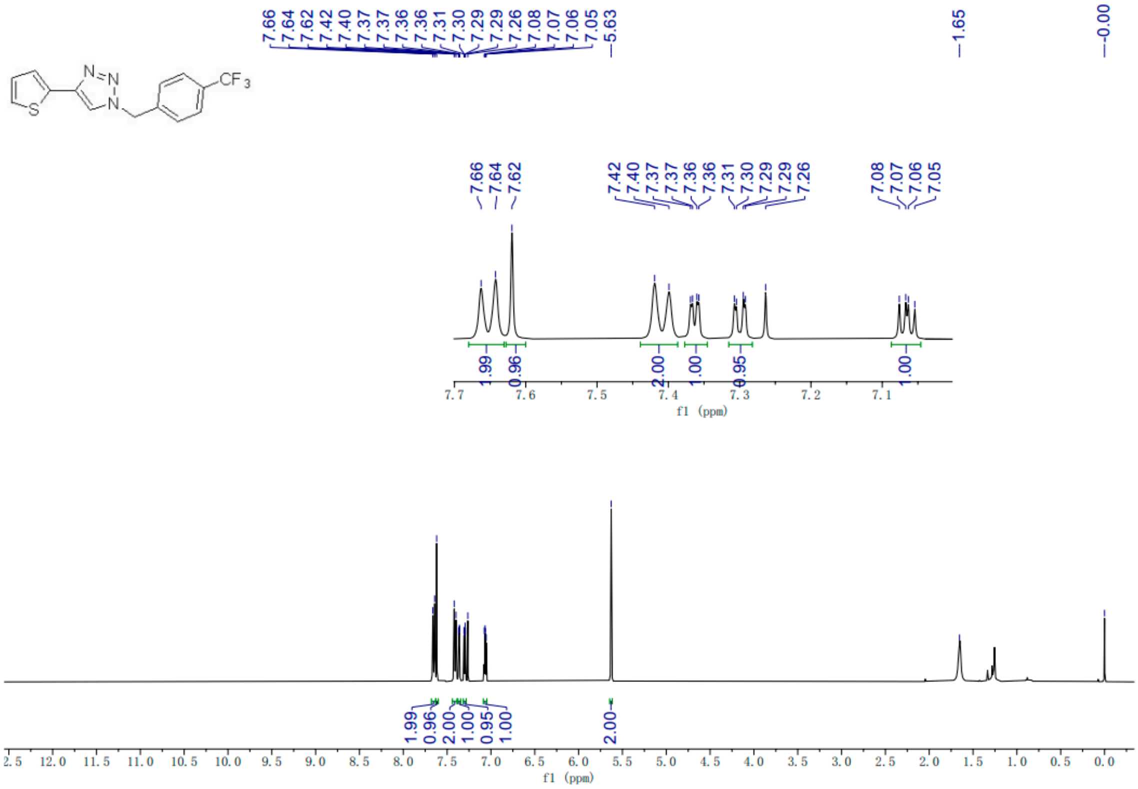

^{1}H NMR (400 MHz, CDCl$_3$) spectrum for 5x

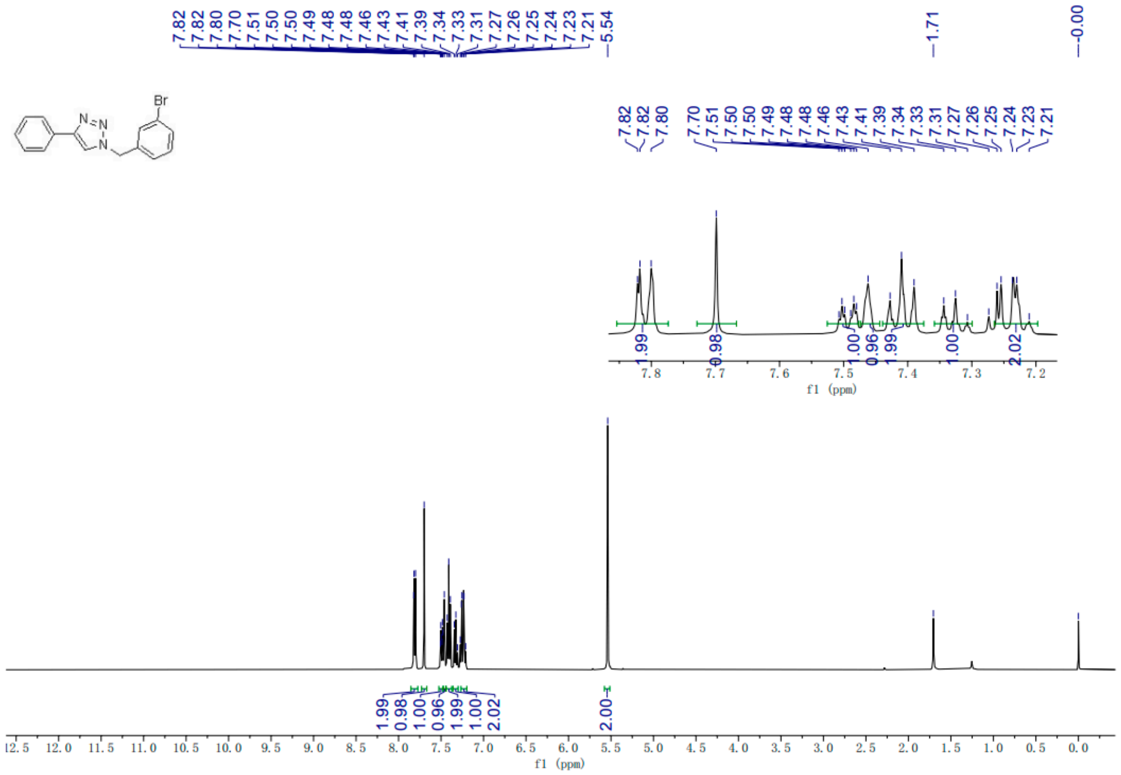

^{1}H NMR (400 MHz, $CDCl_3$) spectrum for 5y

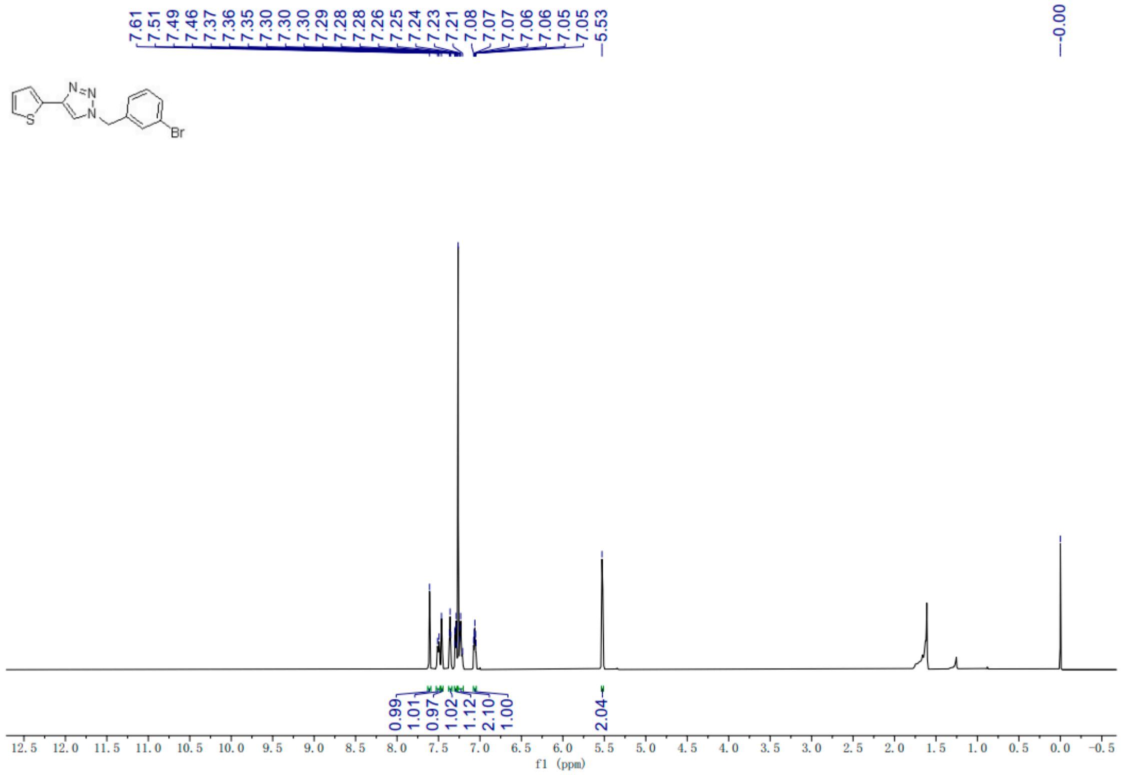

^{13}C NMR (101 MHz, $CDCl_3$) spectrum for 5y

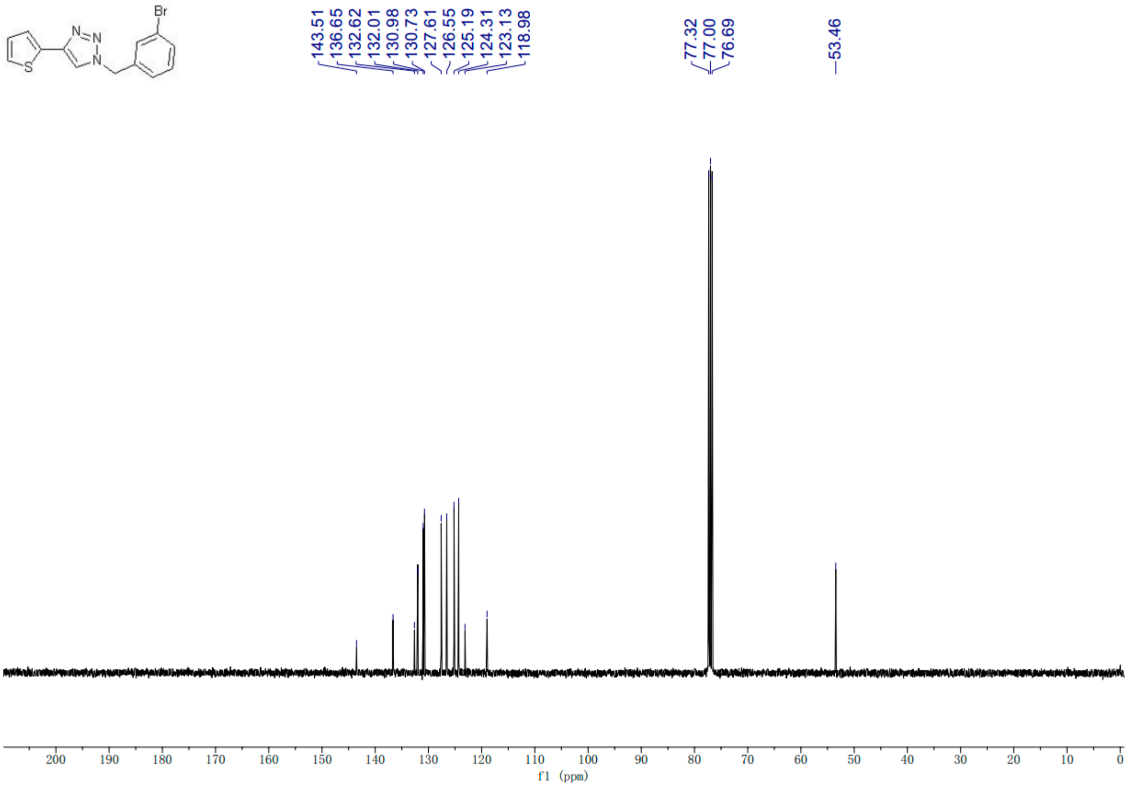

^{1}H NMR (400 MHz, CDCl$_3$) spectrum for 5z

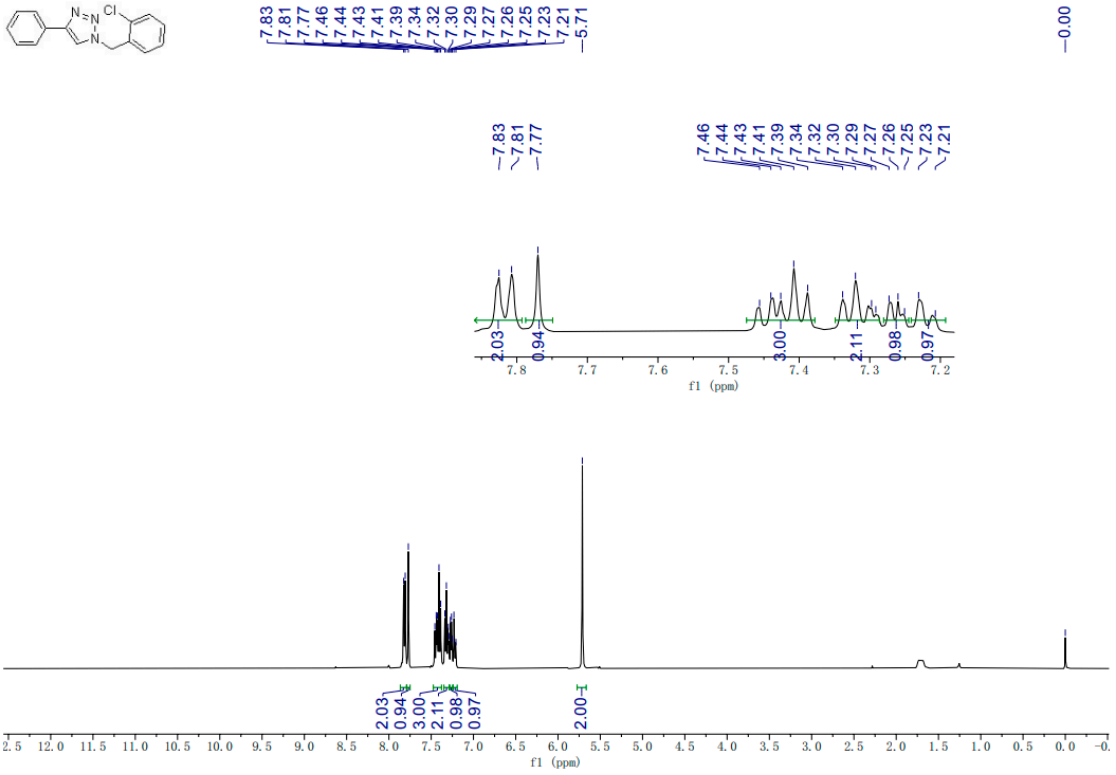

^{1}H NMR (400 MHz, CDCl$_3$) spectrum for 5aa

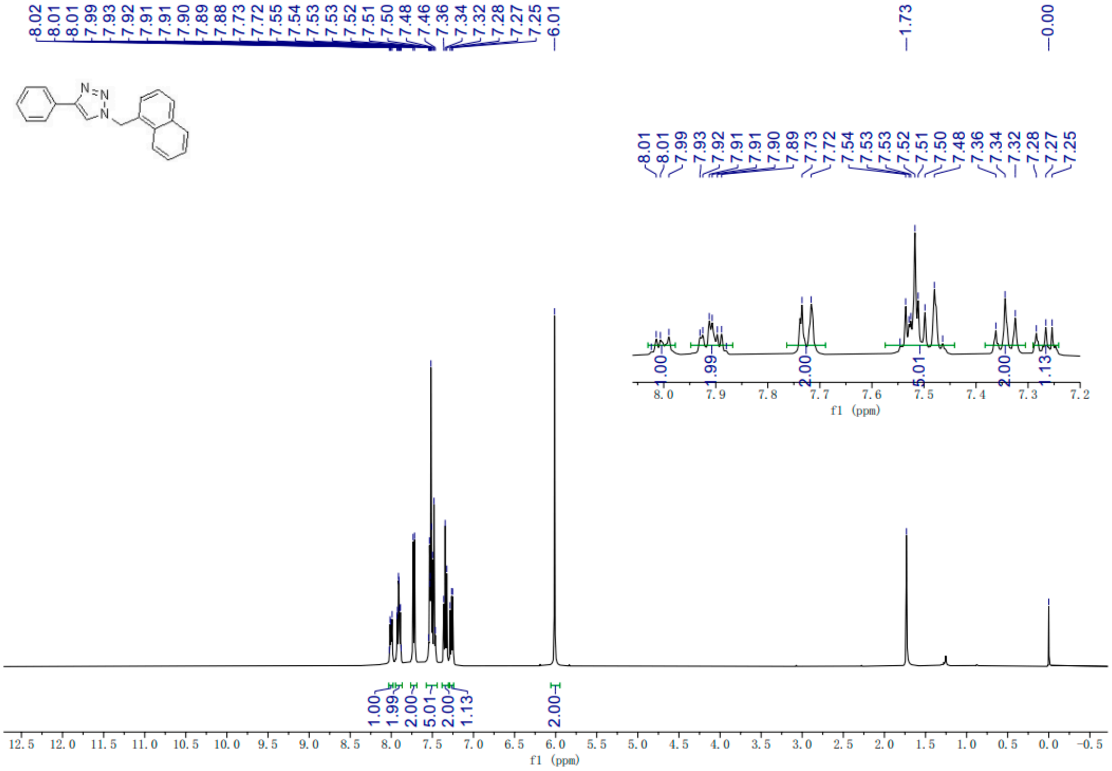

^{1}H NMR (400 MHz, CDCl$_3$) spectrum for 5ab

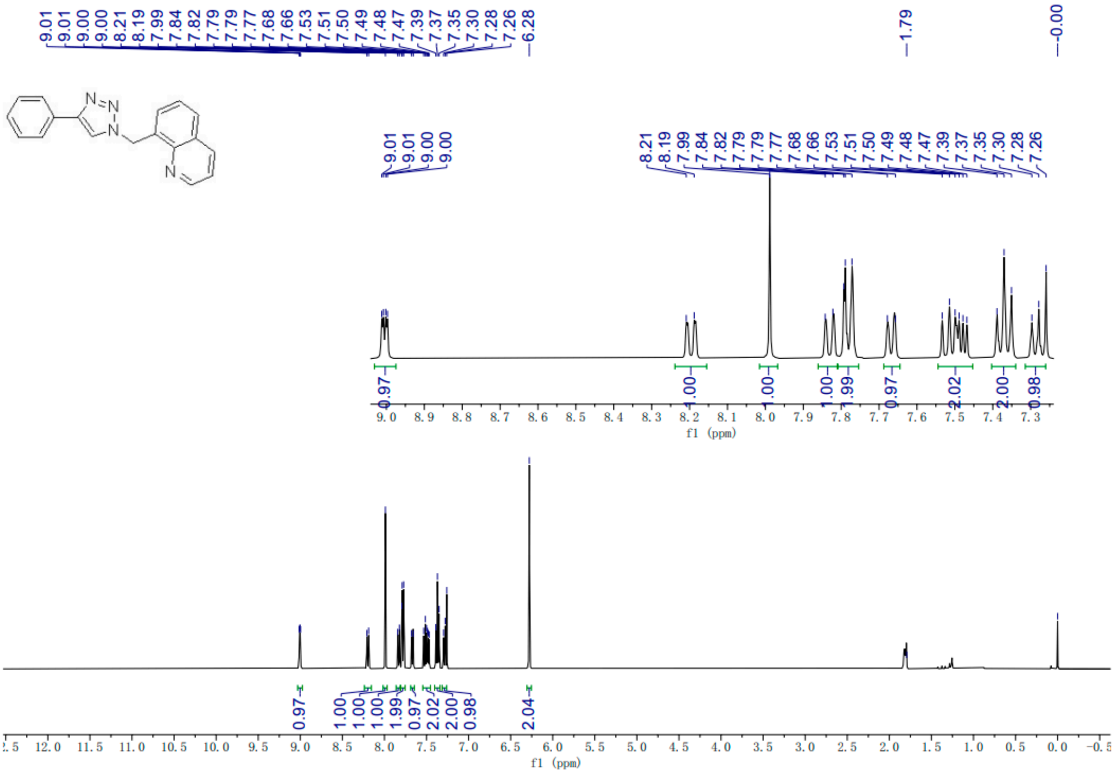

^{13}C NMR (101 MHz, CDCl$_3$) spectrum for 5ab

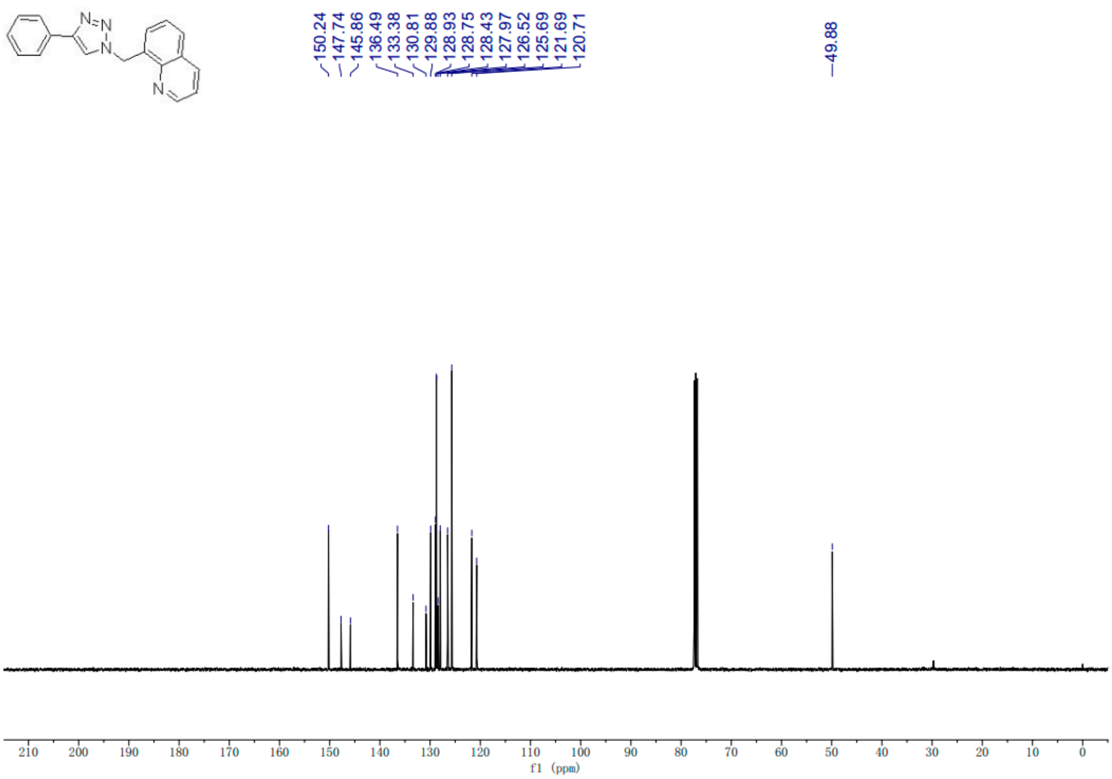

^{1}H NMR (400 MHz, CDCl$_3$) spectrum for 5ac

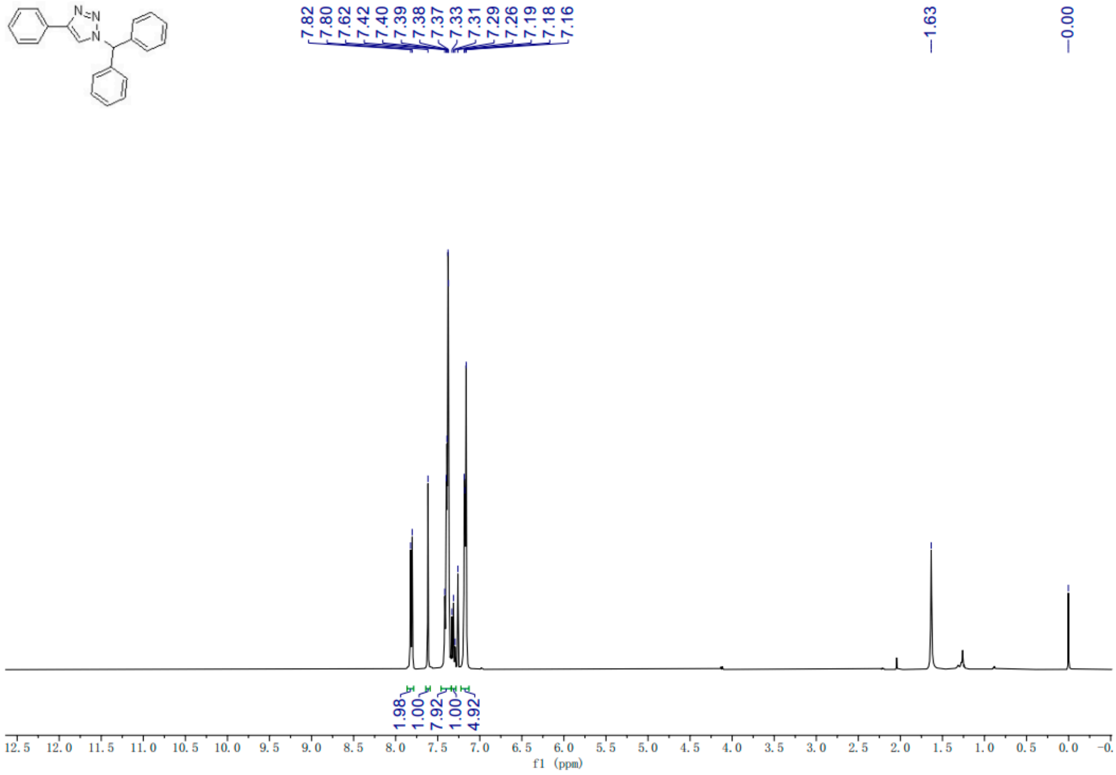

^{1}H NMR (400 MHz, CDCl$_3$) spectrum for 5ad

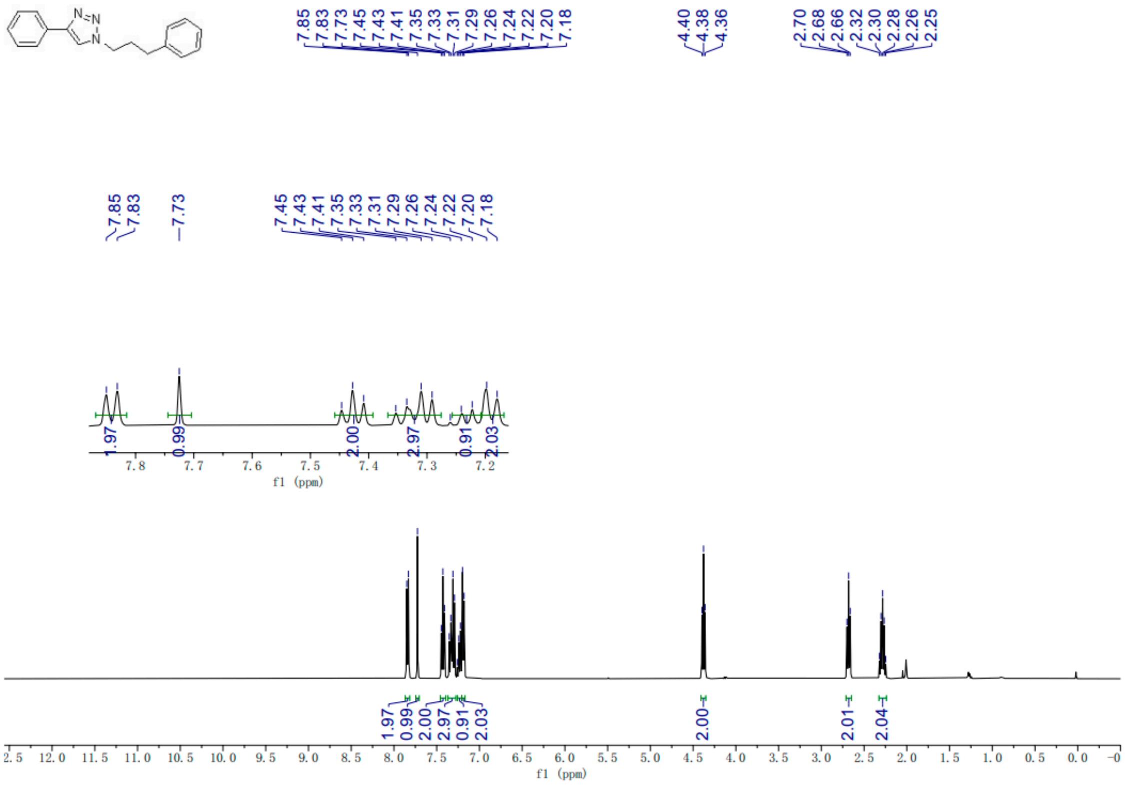

^{1}H NMR (400 MHz, $CDCl_3$) spectrum for 5ae

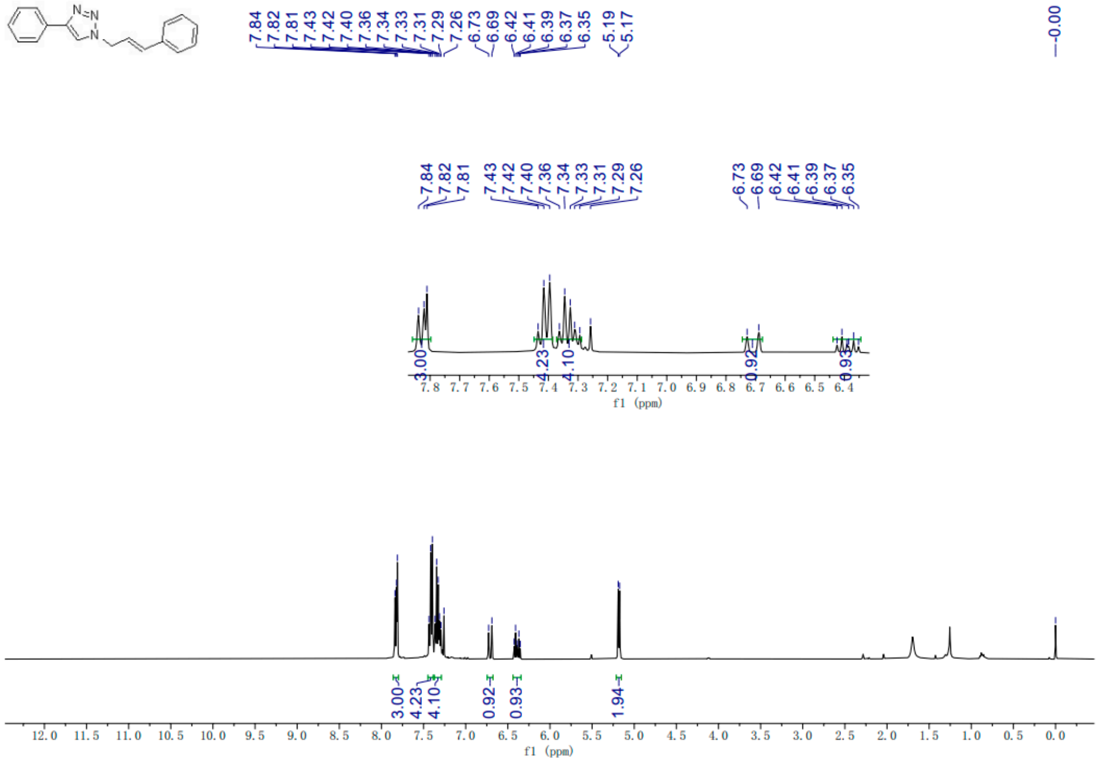

^{1}H NMR (400 MHz, $CDCl_3$) spectrum for 5af

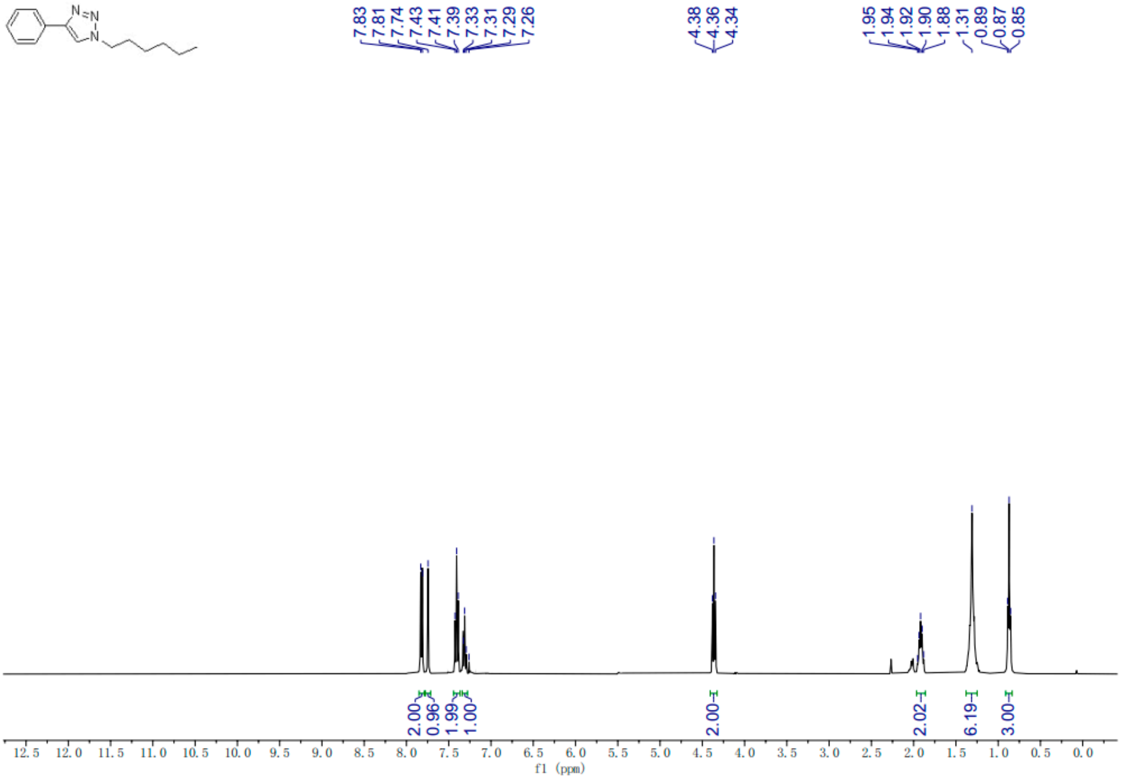

^{1}H NMR (400 MHz, $CDCl_3$) spectrum for 5ag

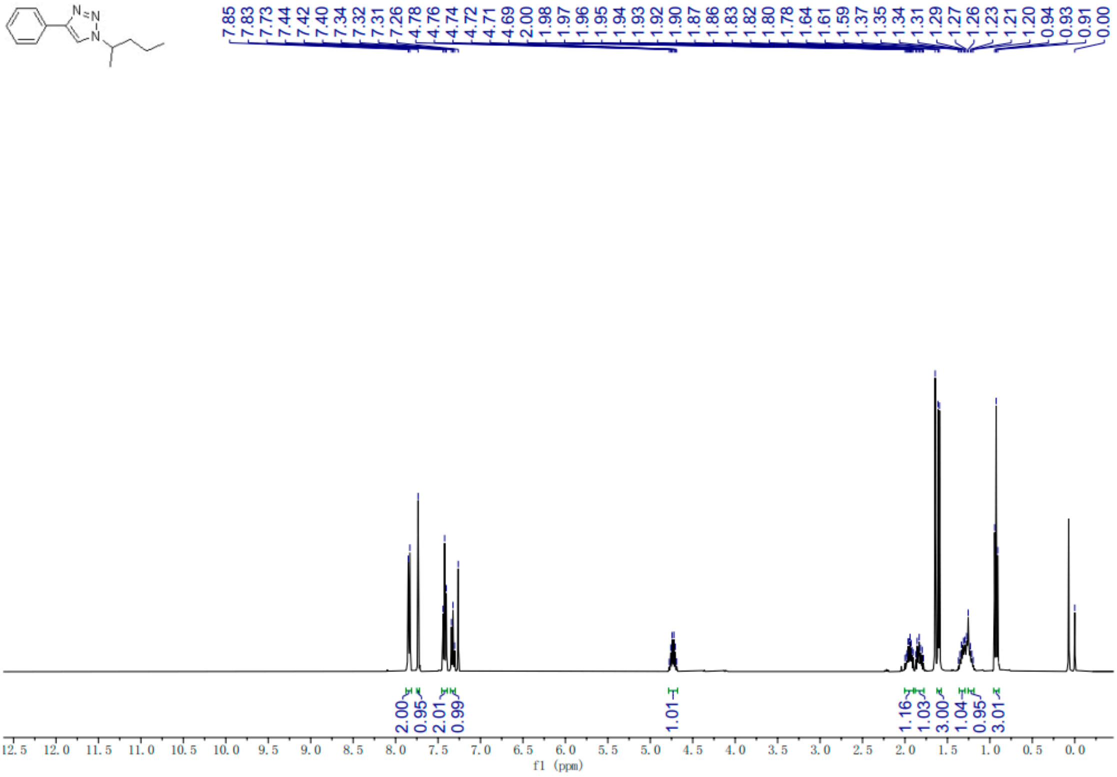

^{13}C NMR (101 MHz, $CDCl_3$) spectrum for 5ag

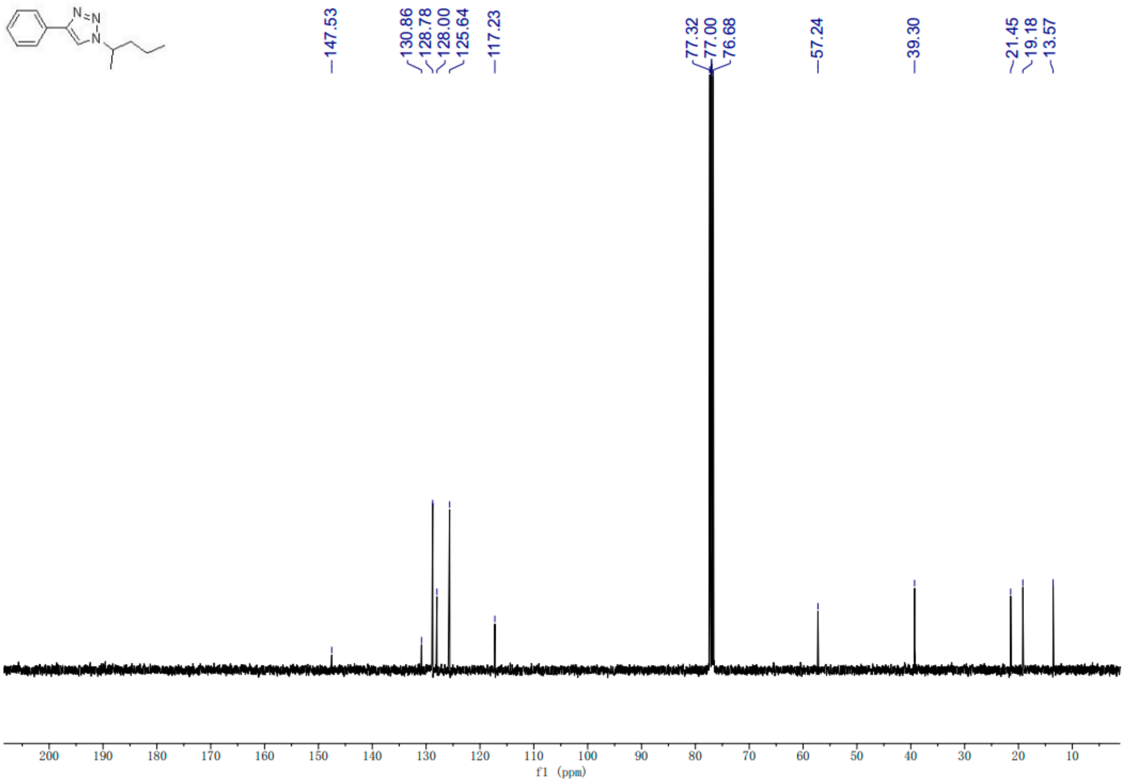

^{1}H NMR (400 MHz, CDCl$_3$) spectrum for 5ah

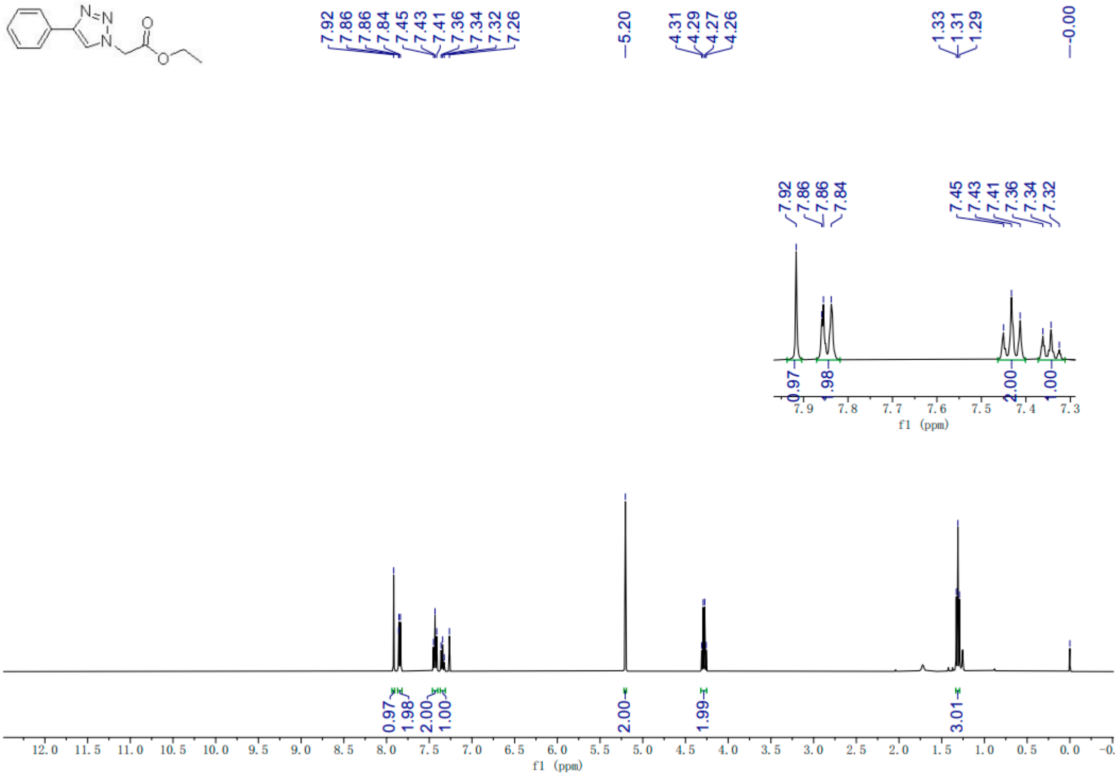

^{1}H NMR (400 MHz, CDCl$_3$) spectrum for 5ai

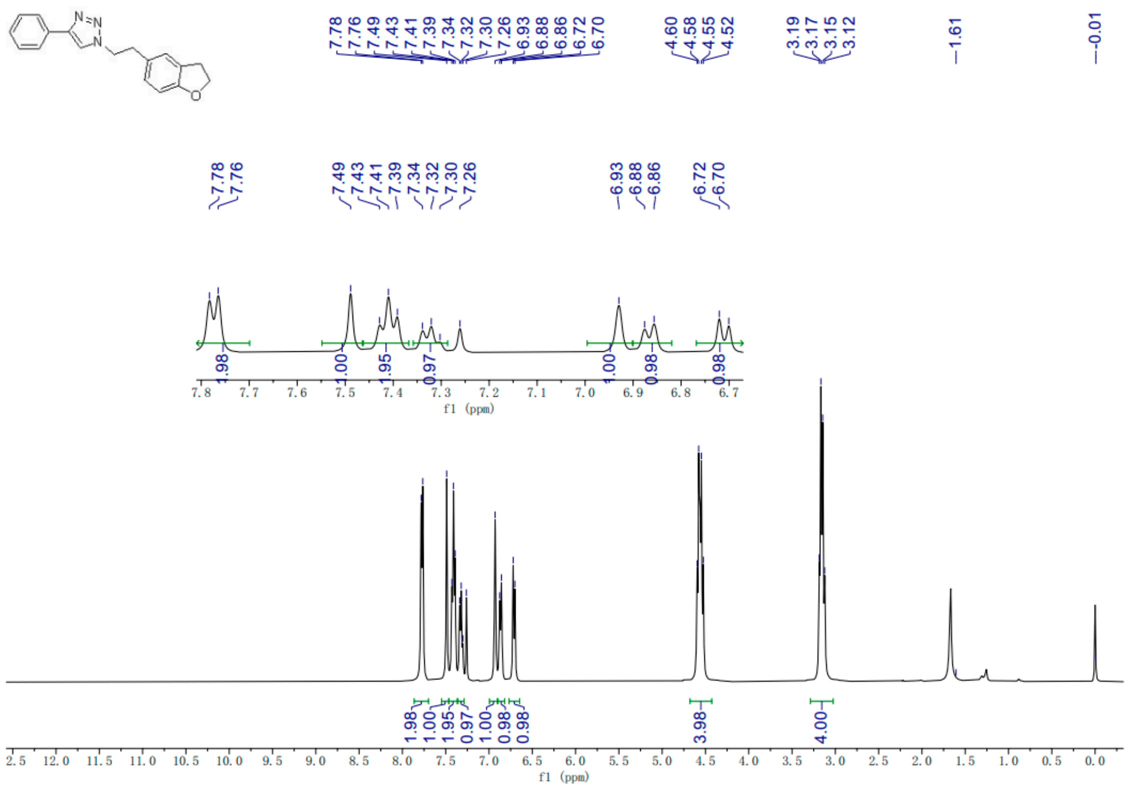

^{13}C NMR (101 MHz, CDCl$_3$) spectrum for 5ai

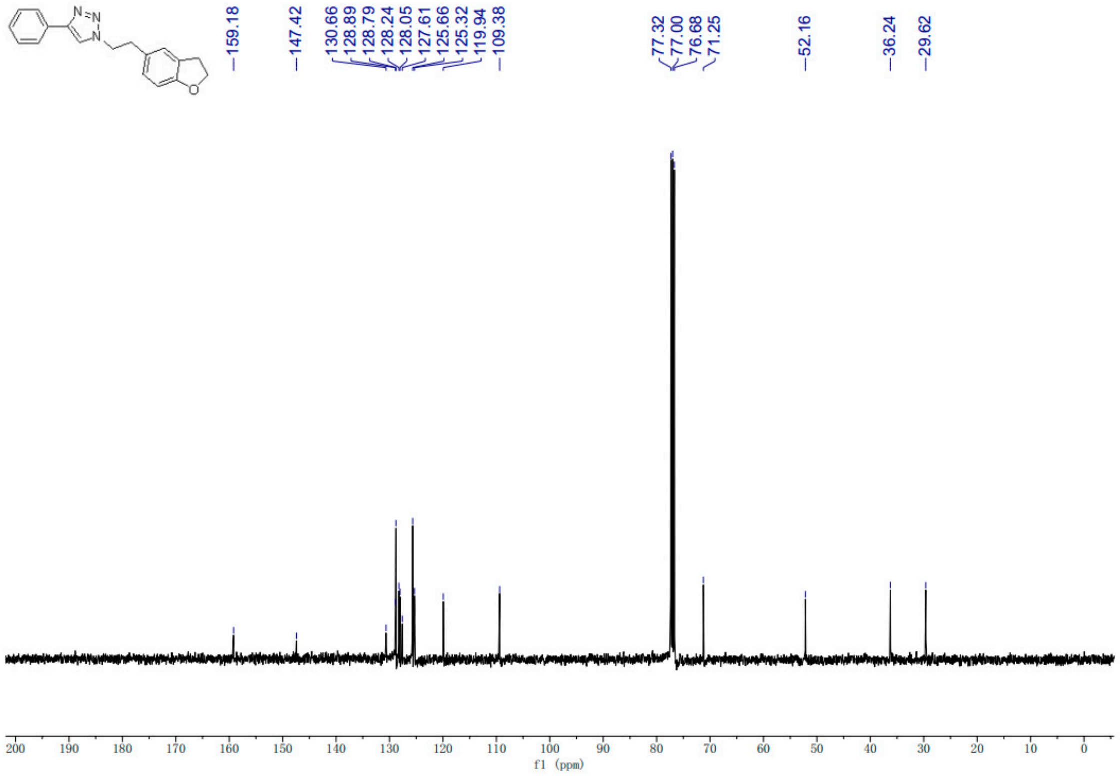

^{1}H NMR (400 MHz, CDCl$_3$) spectrum for 5aj

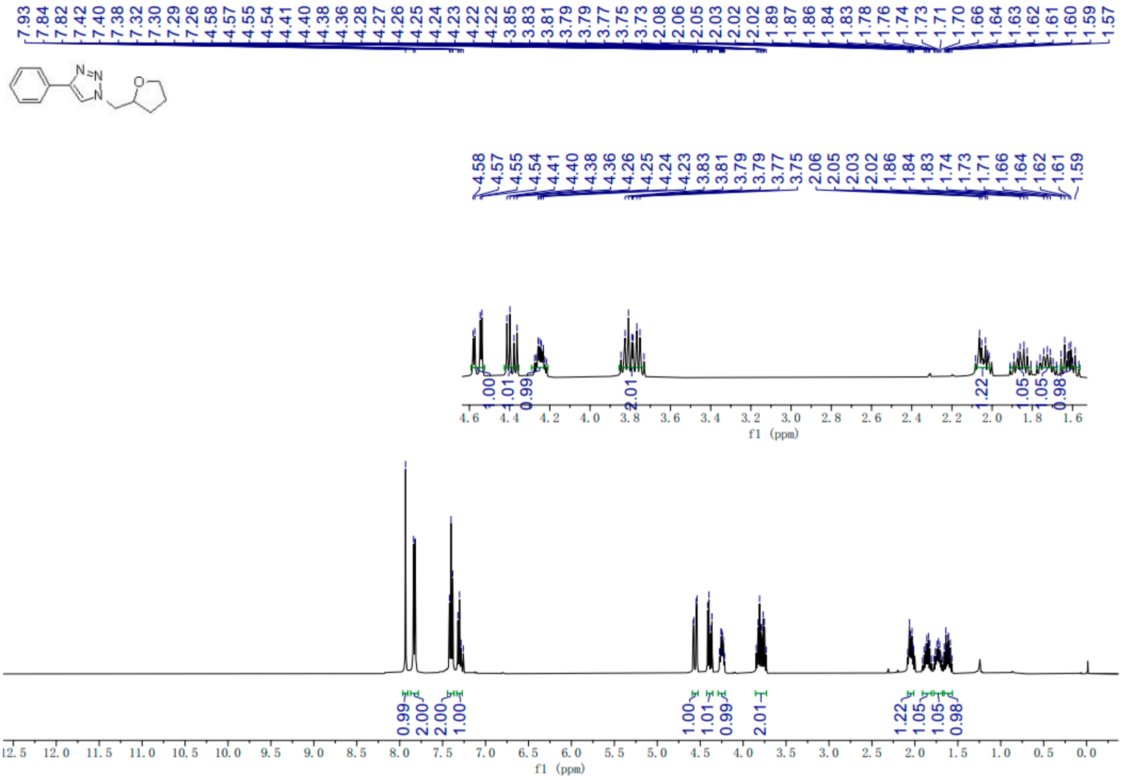

^{1}H NMR (400 MHz, CDCl$_3$) spectrum for 5ak

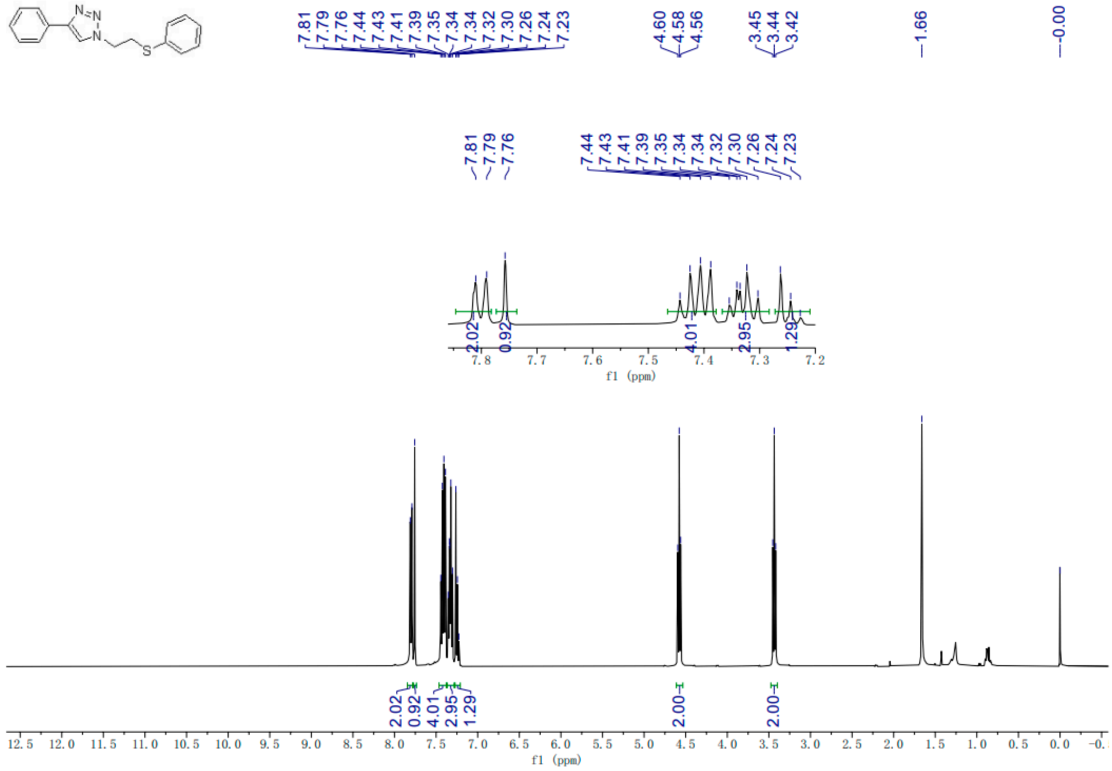

^{13}C NMR (101 MHz, CDCl$_3$) spectrum for 5ak

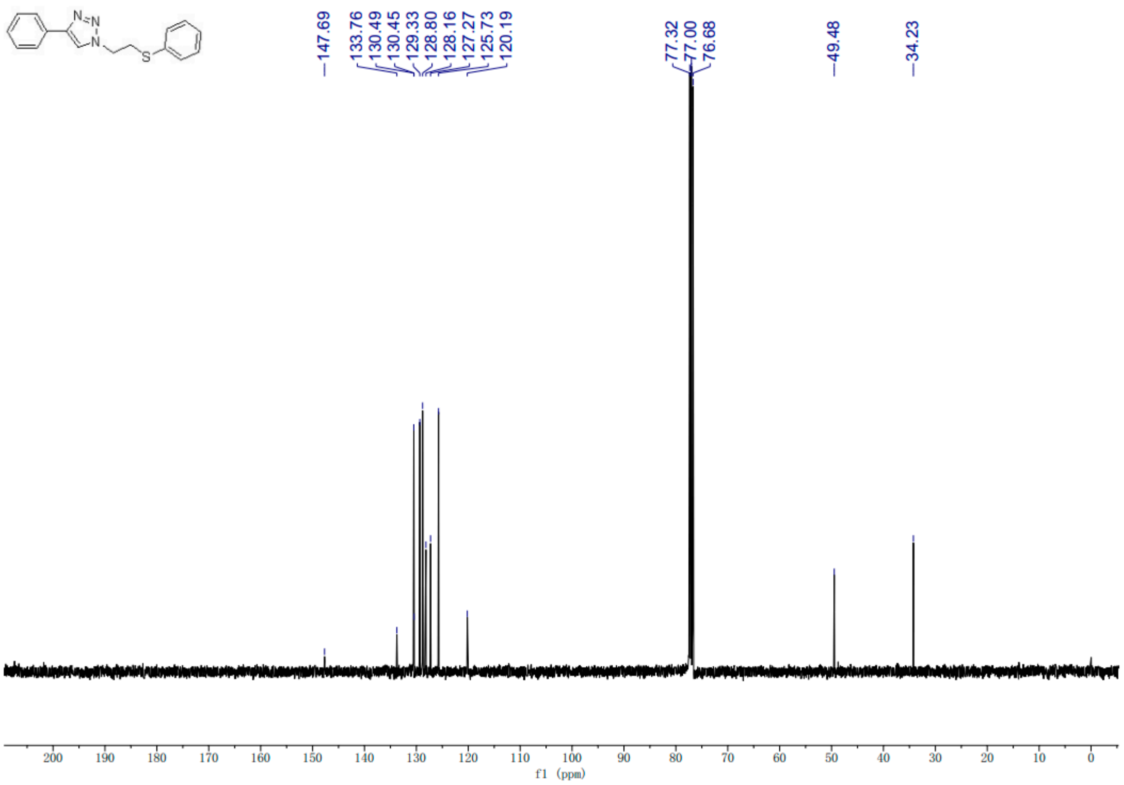

^{1}H NMR (400 MHz, CDCl$_3$) spectrum for 5al

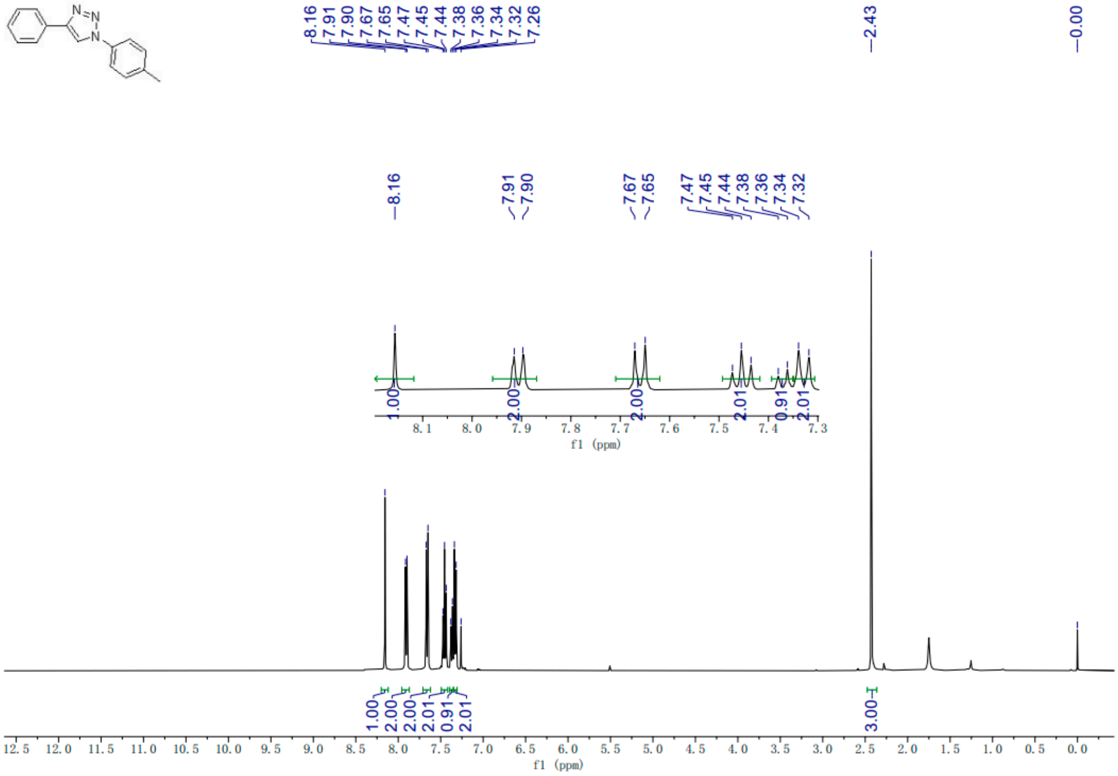

^{1}H NMR (400 MHz, CDCl$_3$) spectrum for 5am

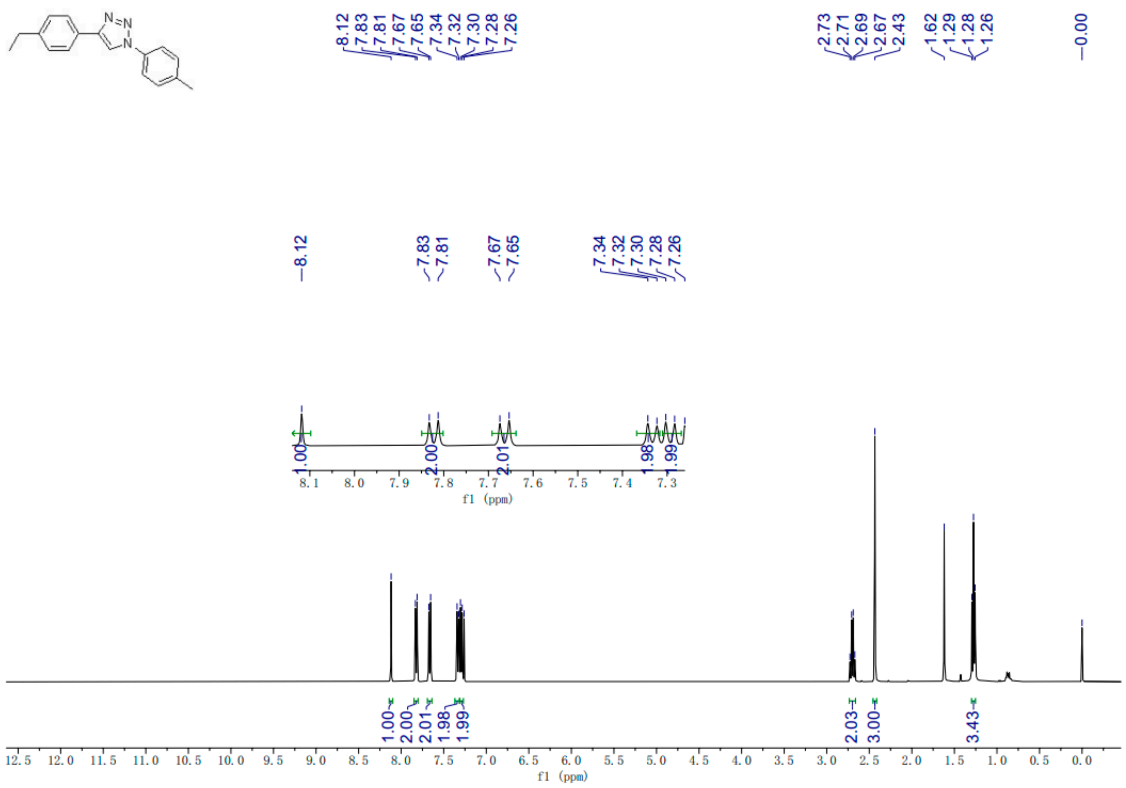

^{13}C NMR (101 MHz, CDCl$_3$) spectrum for 5am

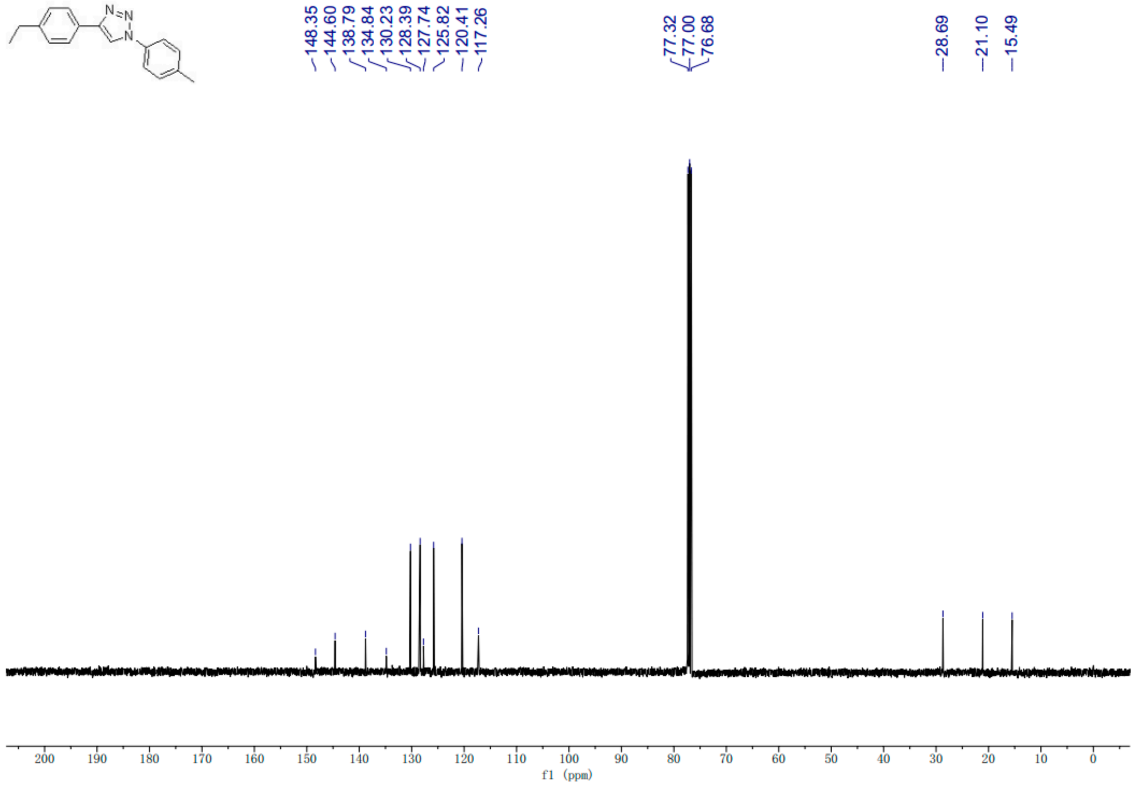

^{1}H NMR (400 MHz, CDCl$_3$) spectrum for 5an

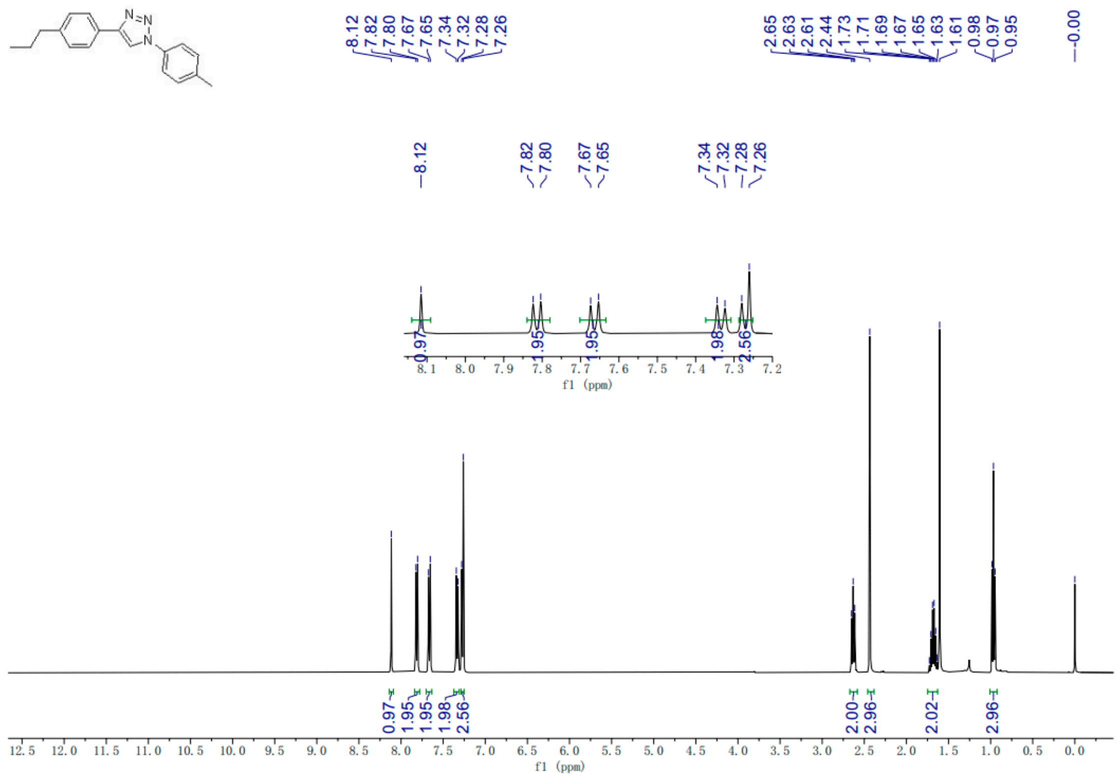

^{13}C NMR (101 MHz, CDCl$_3$) spectrum for 5an

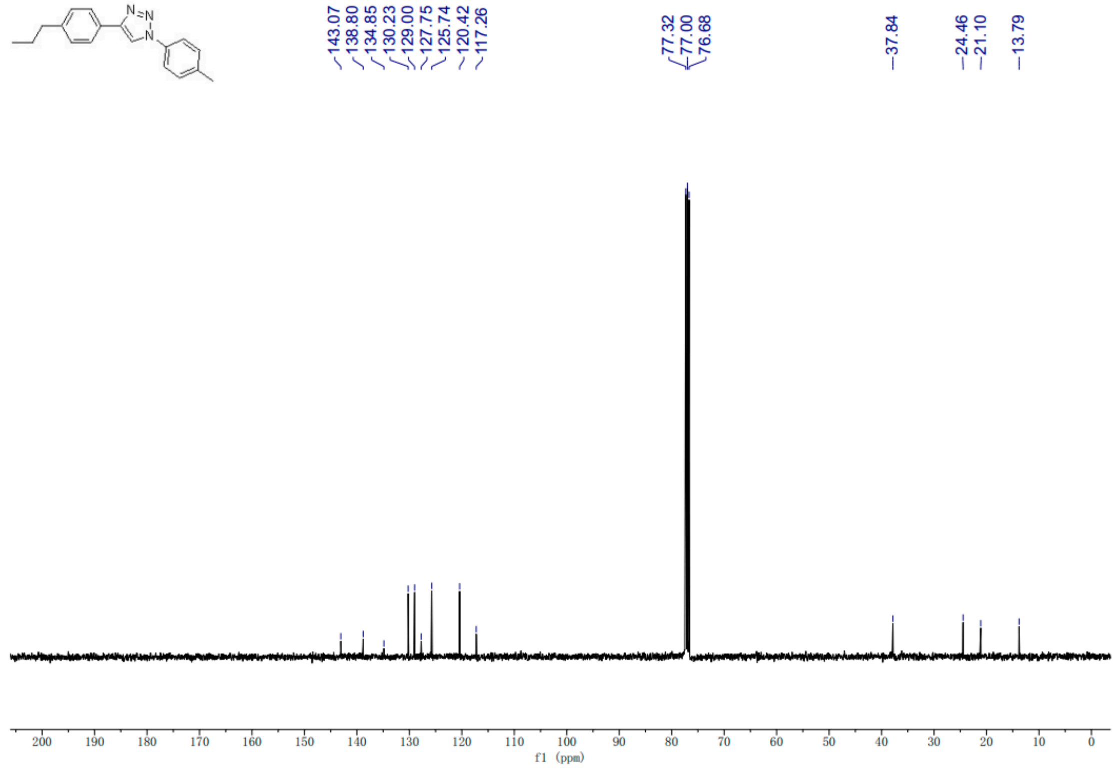

^{1}H NMR (400 MHz, CDCl$_3$) spectrum for 5ao

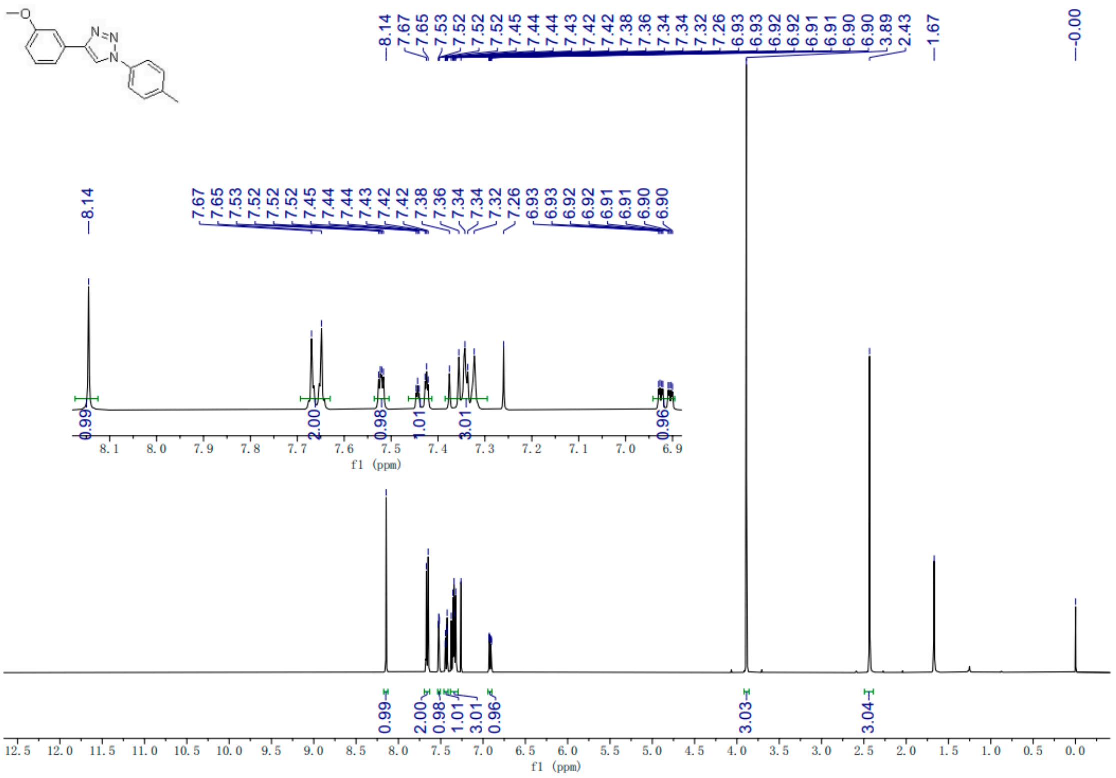

^{13}C NMR (101 MHz, $CDCl_3$) spectrum for 5ao

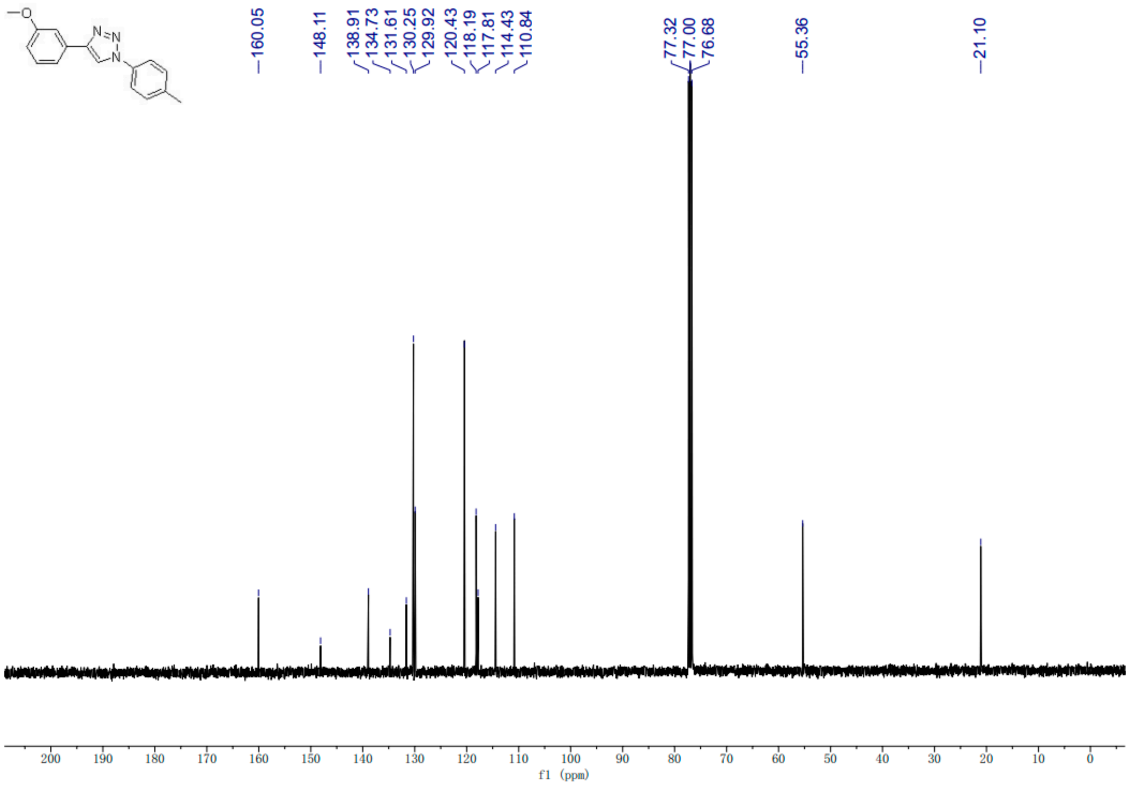

^{1}H NMR (400 MHz, $CDCl_3$) spectrum for 5ap

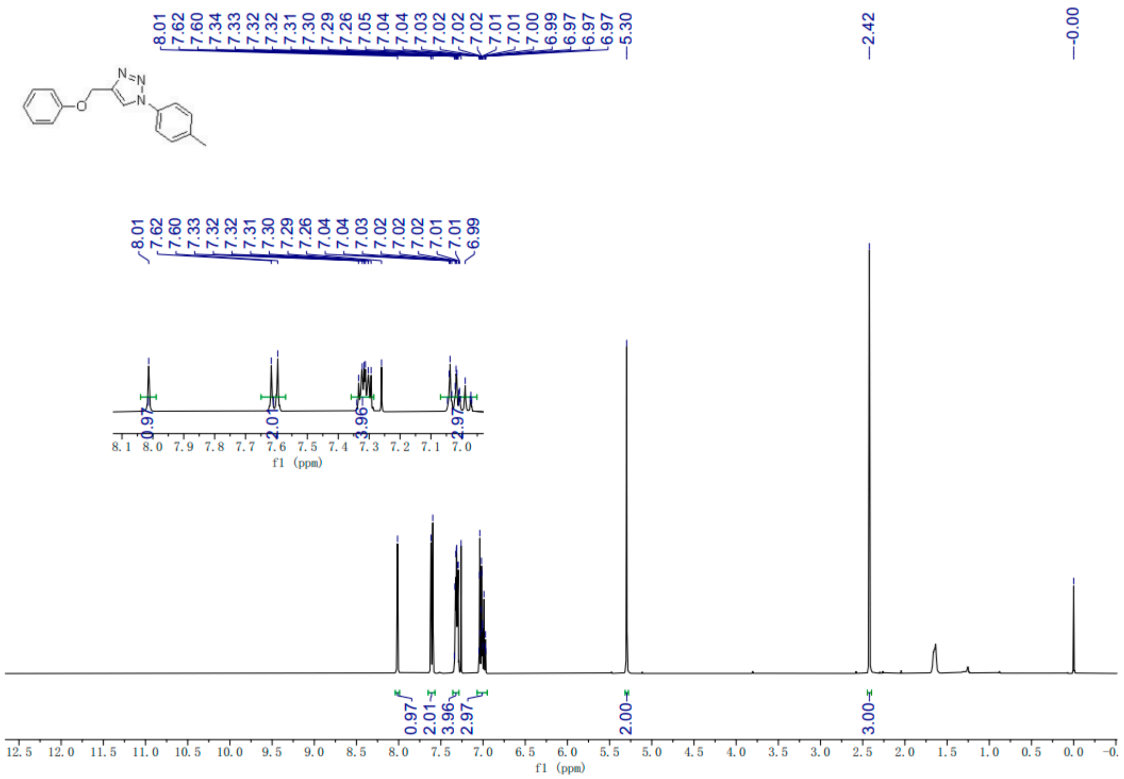

^{1}H NMR (400 MHz, $CDCl_3$) spectrum for 5aq

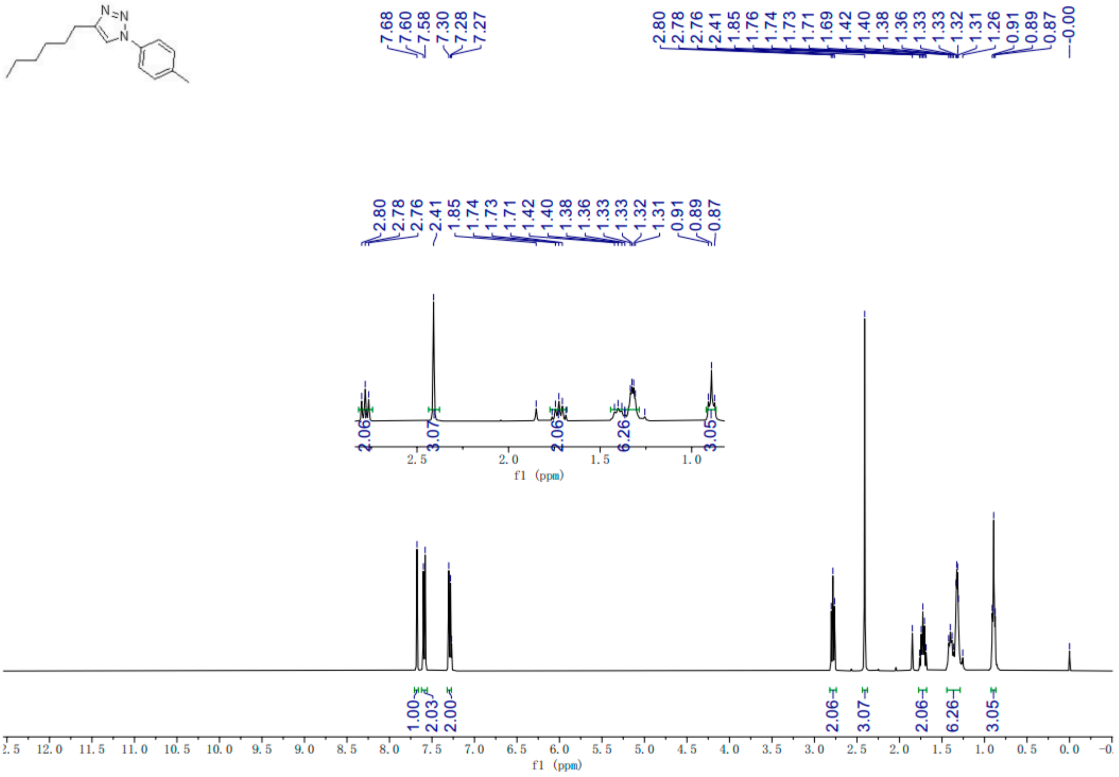

^{1}H NMR (400 MHz, $CDCl_3$) spectrum for 5ar

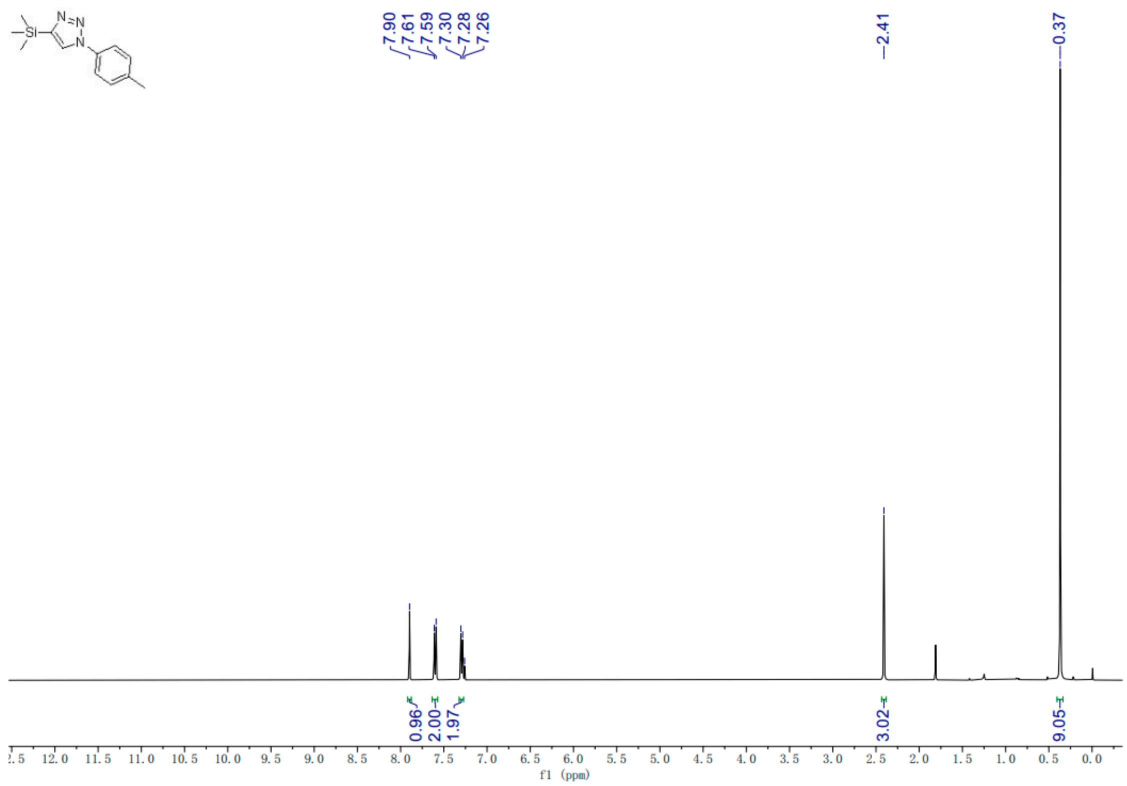

^{1}H NMR (400 MHz, CDCl$_3$) spectrum for 5as

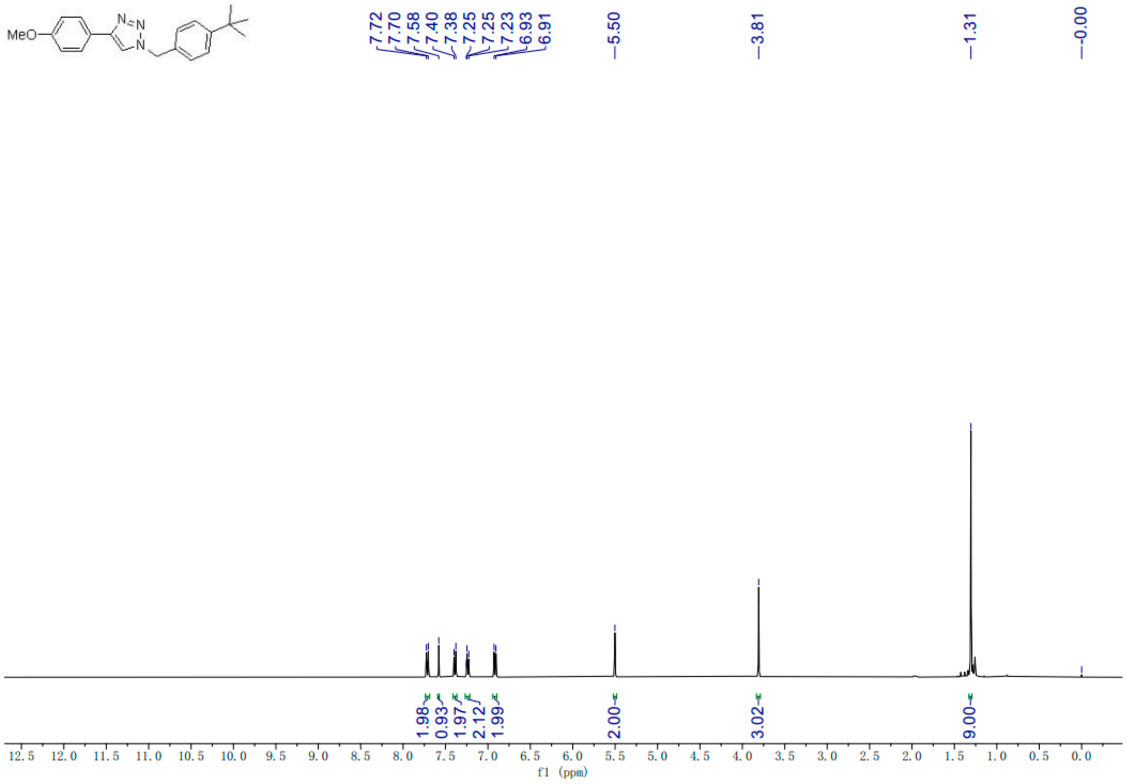

^{1}H NMR (400 MHz, CDCl$_3$) spectrum for 5at

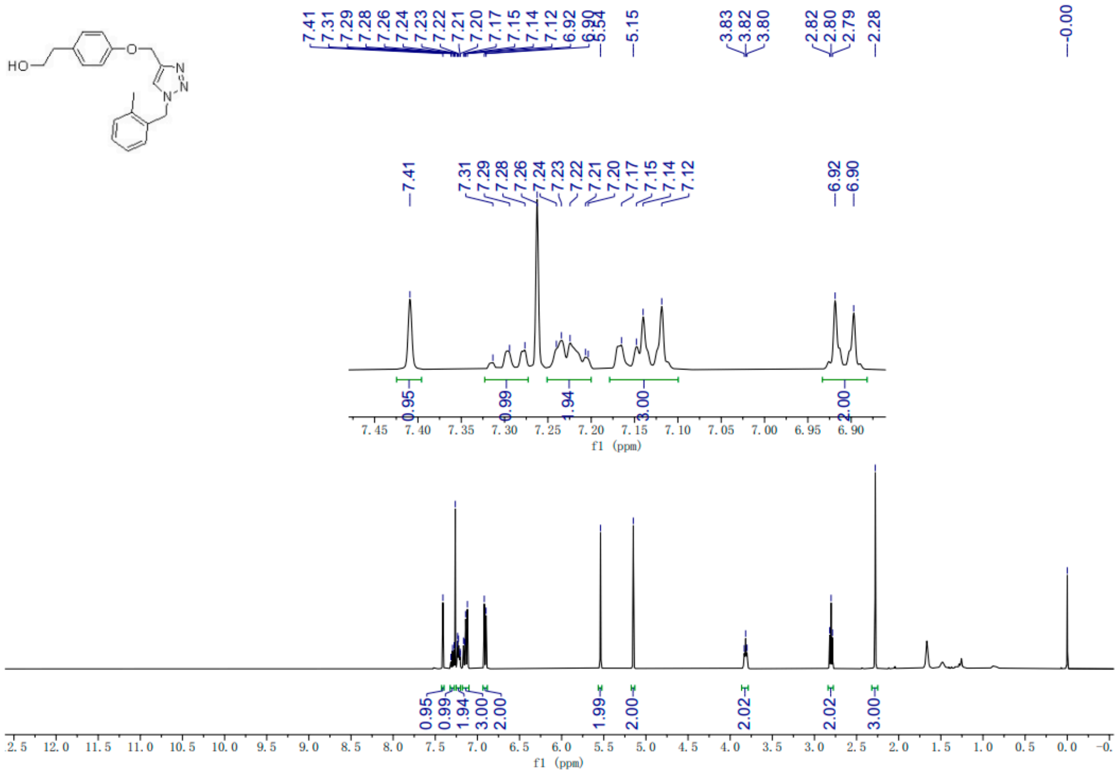

^{1}H NMR (400 MHz, CDCl₃) spectrum for 5au

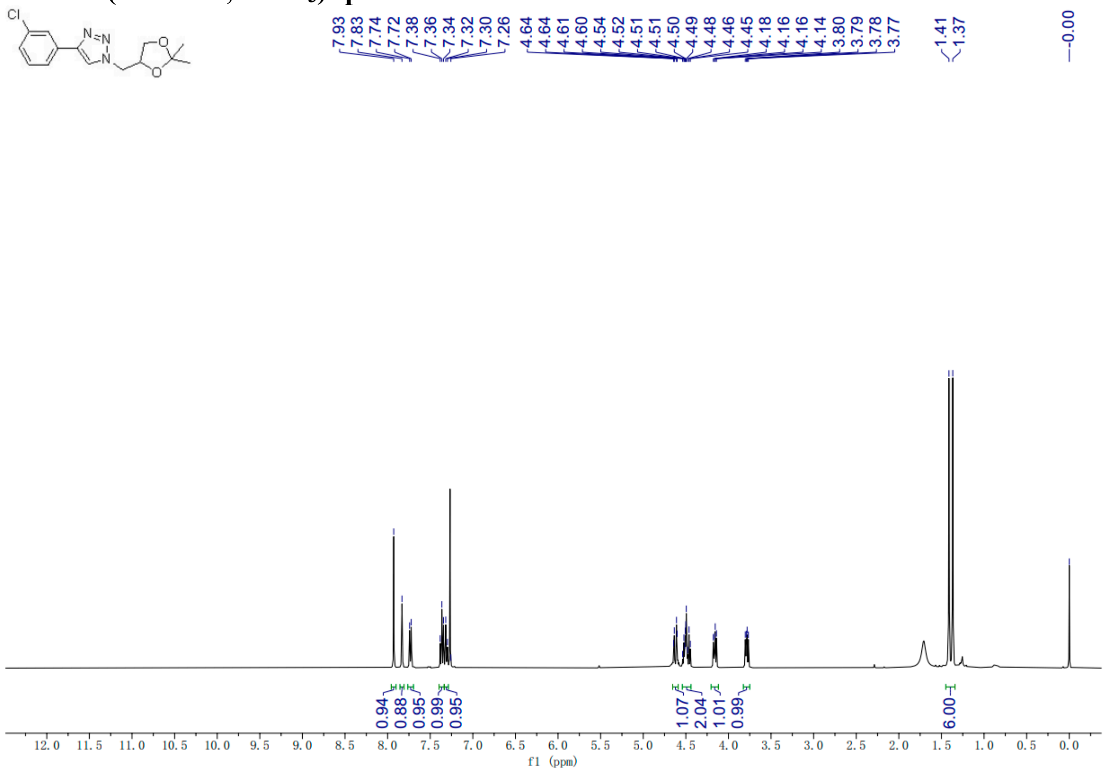

^{1}H NMR (400 MHz, CDCl₃) spectrum for 5av

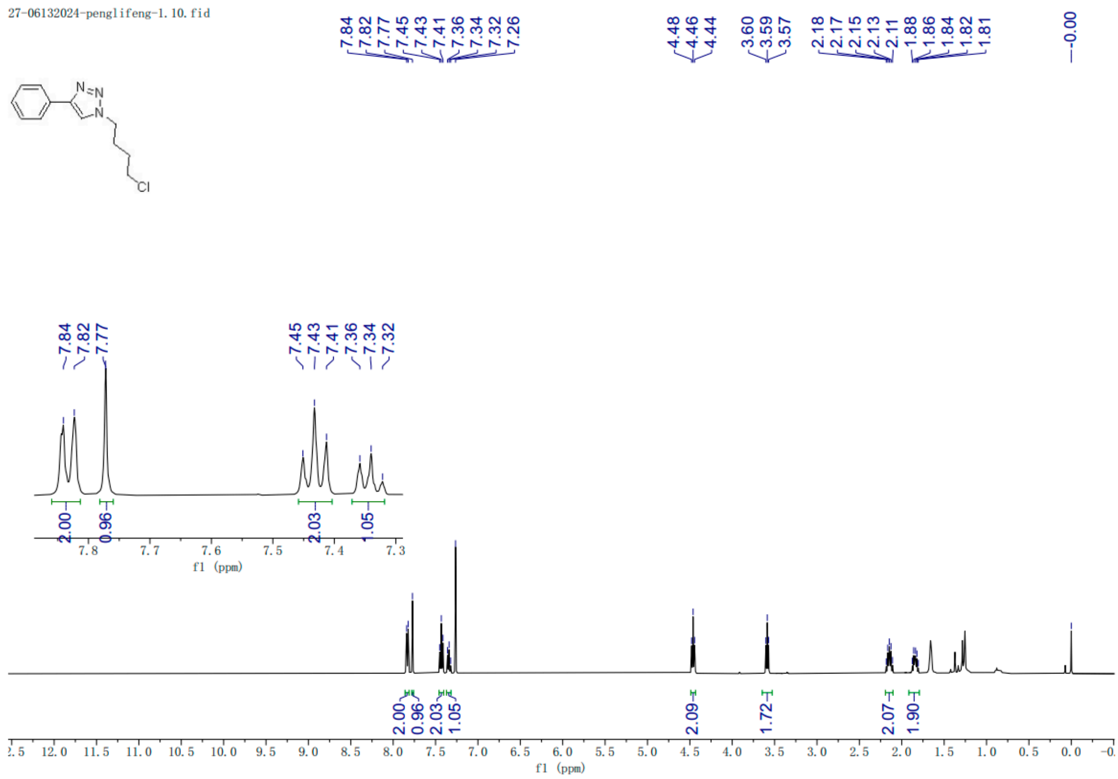

^{1}H NMR (400 MHz, CDCl$_3$) spectrum for 5aw

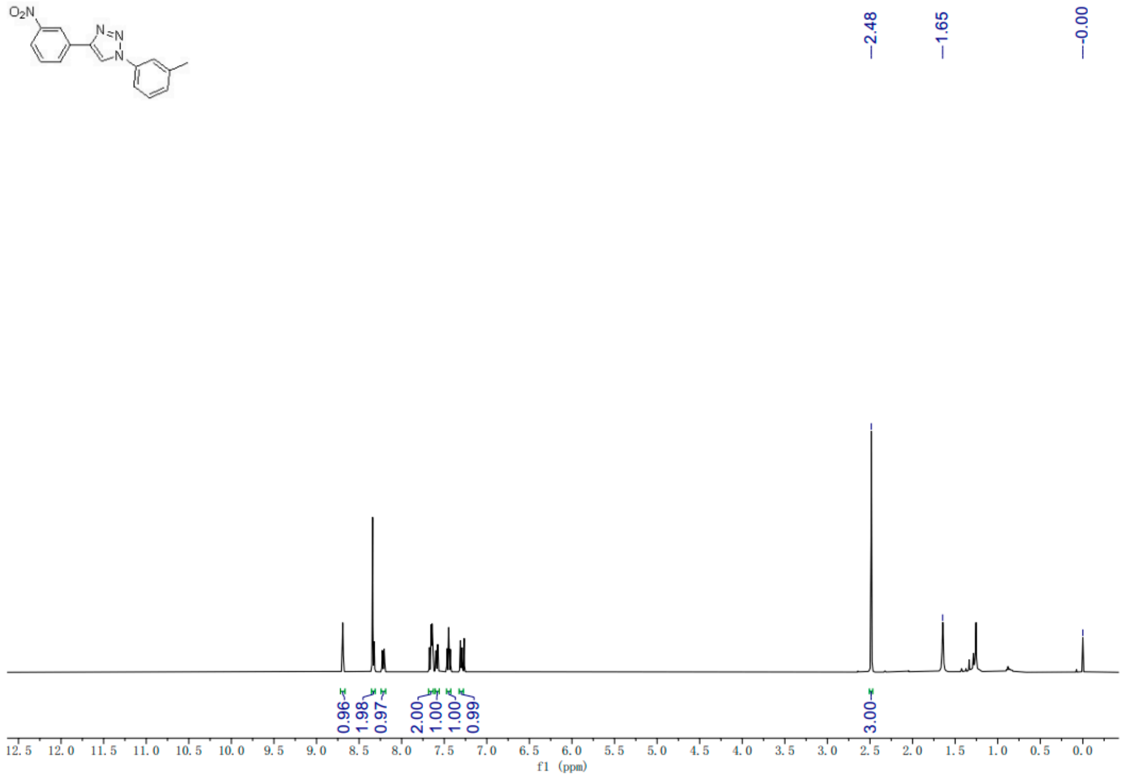

^{1}H NMR (400 MHz, CDCl$_3$) spectrum for 5ax

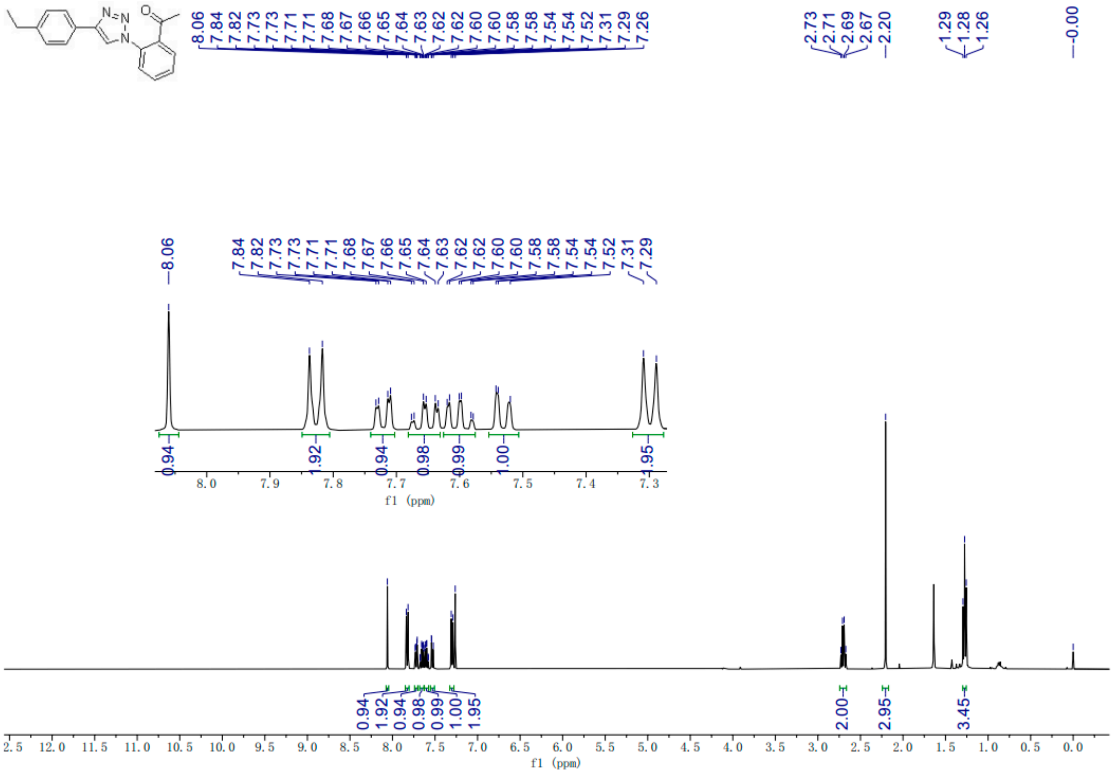

^{1}H NMR (400 MHz, $CDCl_3$) spectrum for 5ay

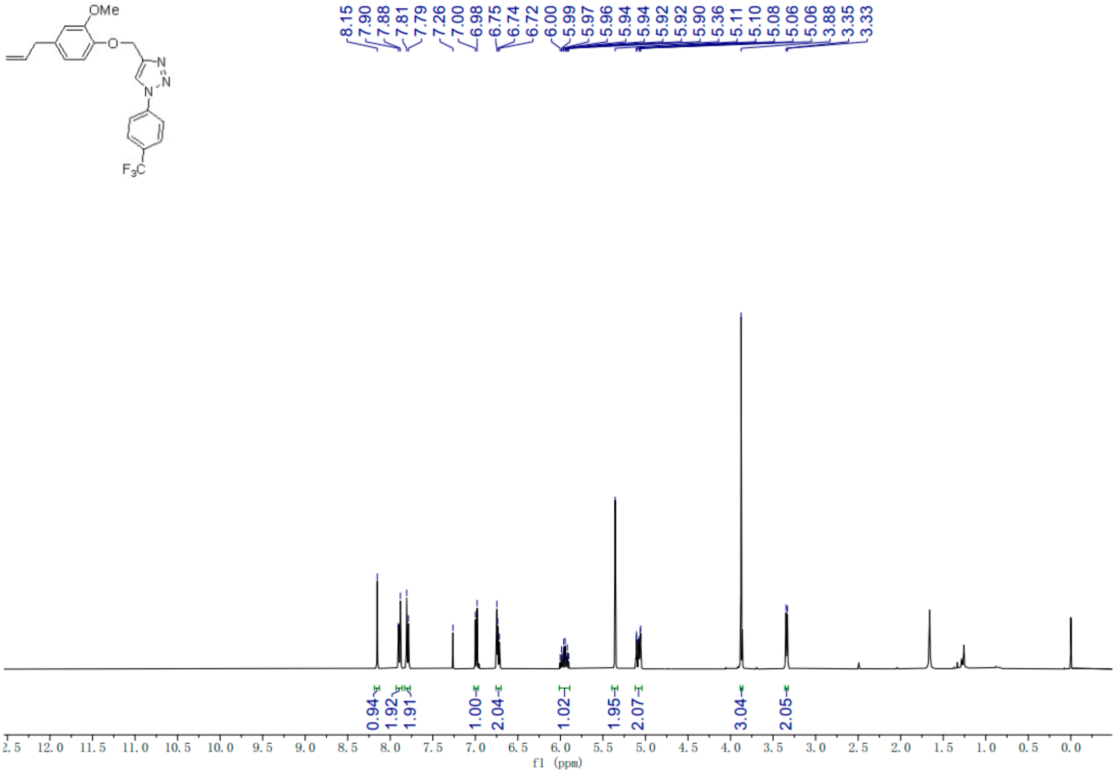

^{1}H NMR (400 MHz, $CDCl_3$) spectrum for 6a

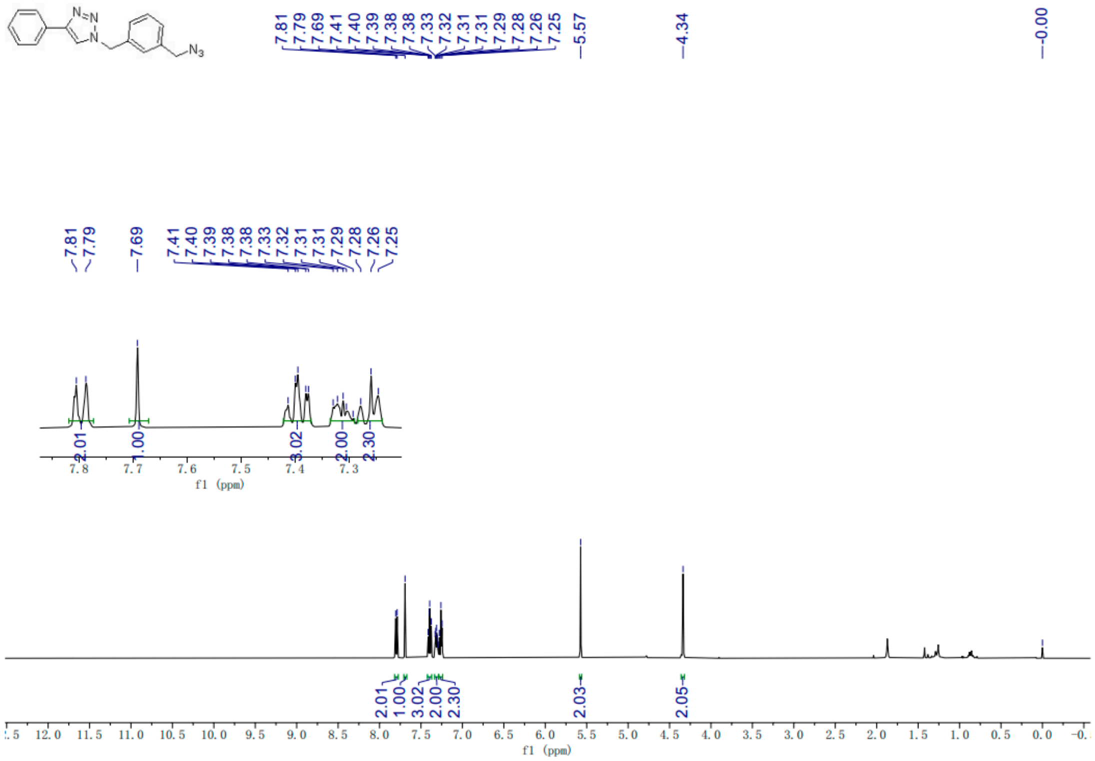

^{13}C NMR (101 MHz, CDCl$_3$) spectrum for 6a

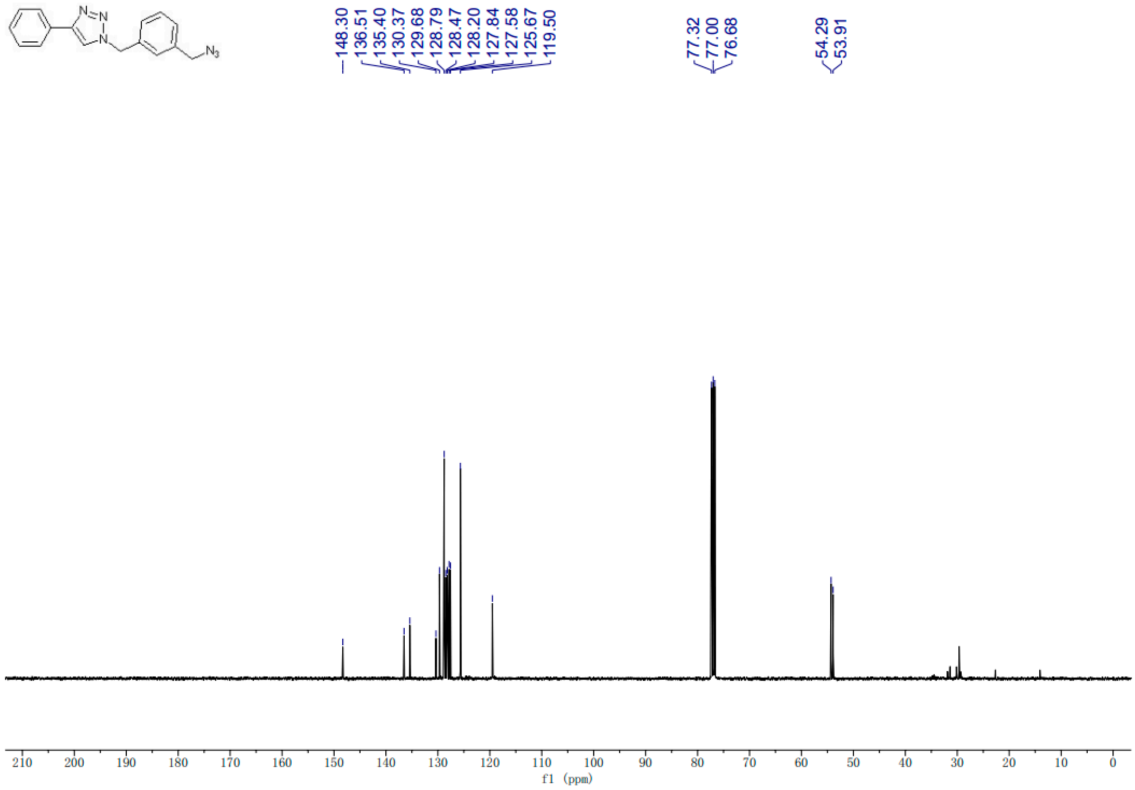

^{1}H NMR (400 MHz, CDCl$_3$) spectrum for 6b

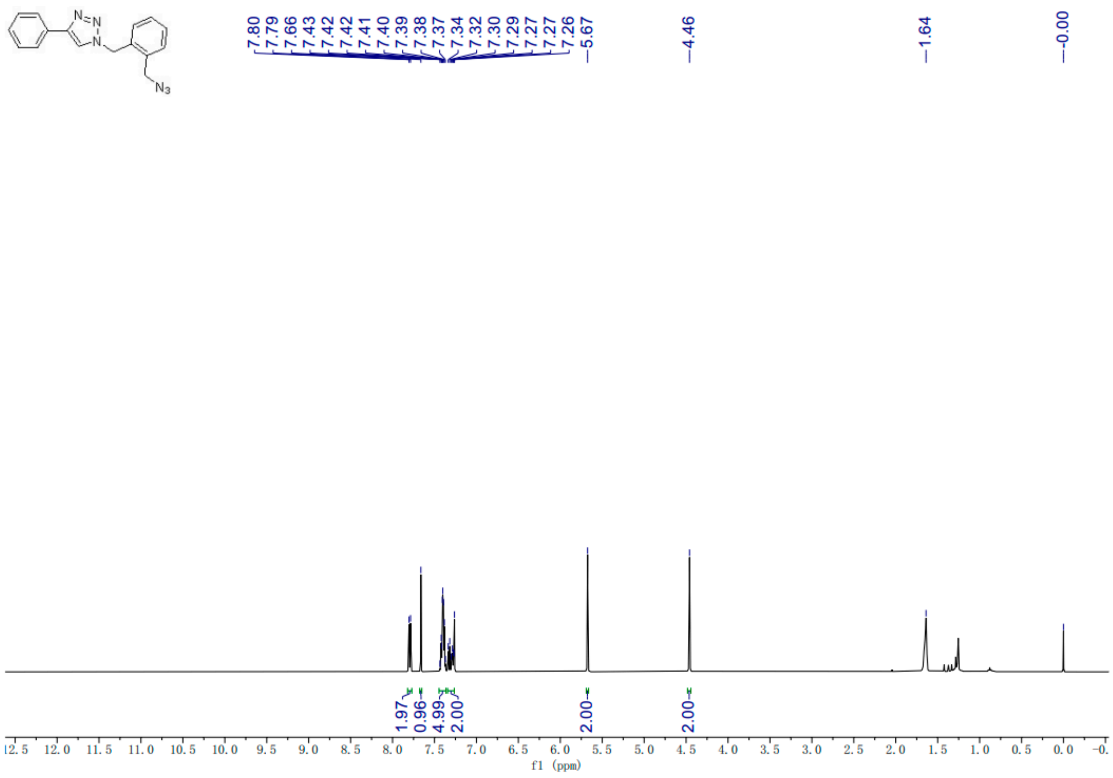

^{13}C NMR (101 MHz, $CDCl_3$) spectrum for 6b

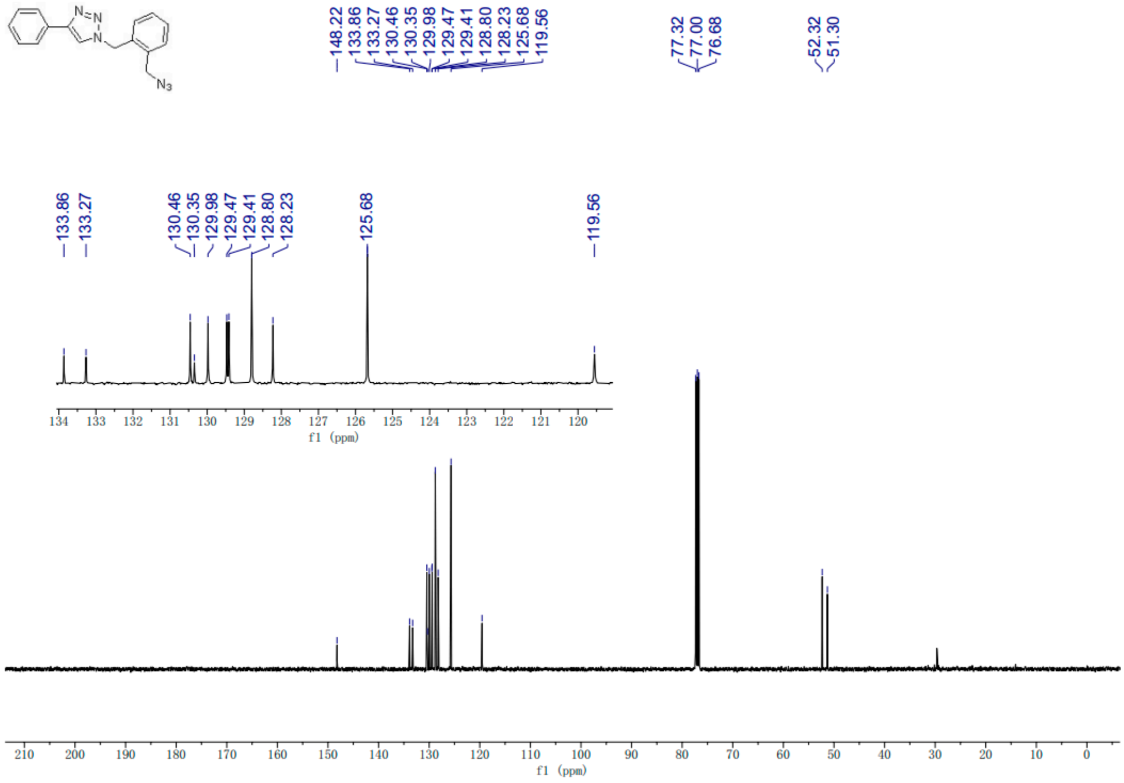

^{1}H NMR (400 MHz, $CDCl_3$) spectrum for 6c

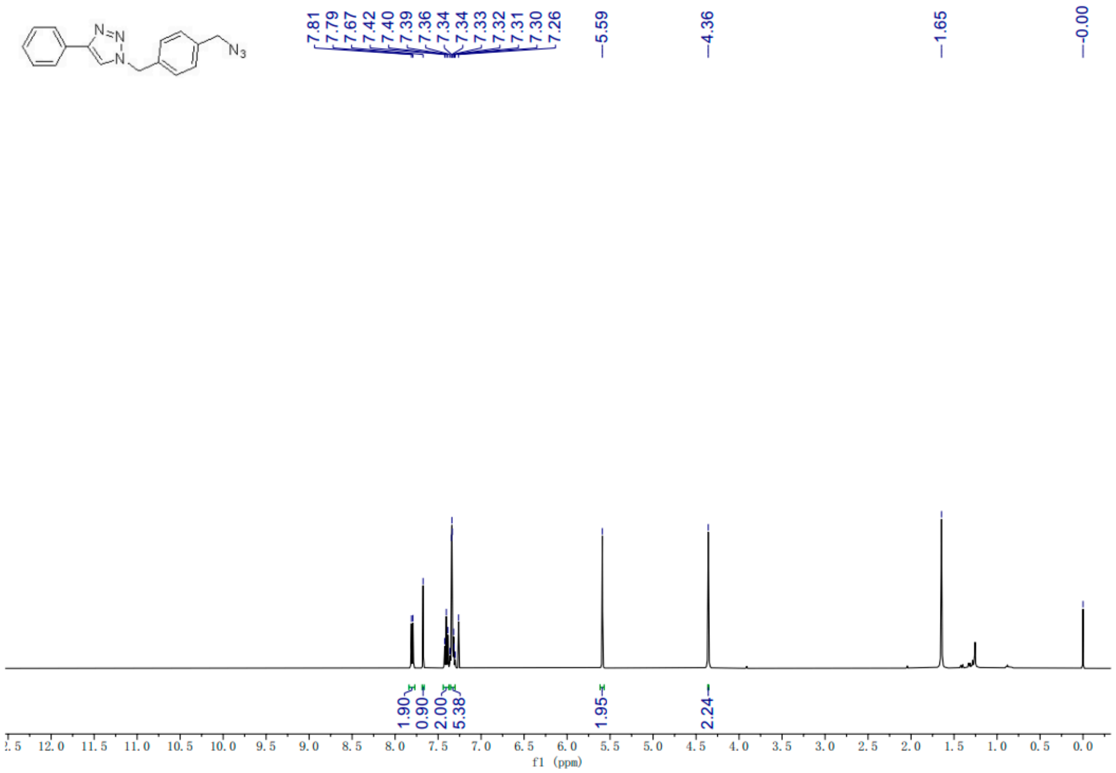

^{1}H NMR (400 MHz, CDCl$_3$) spectrum for 6d

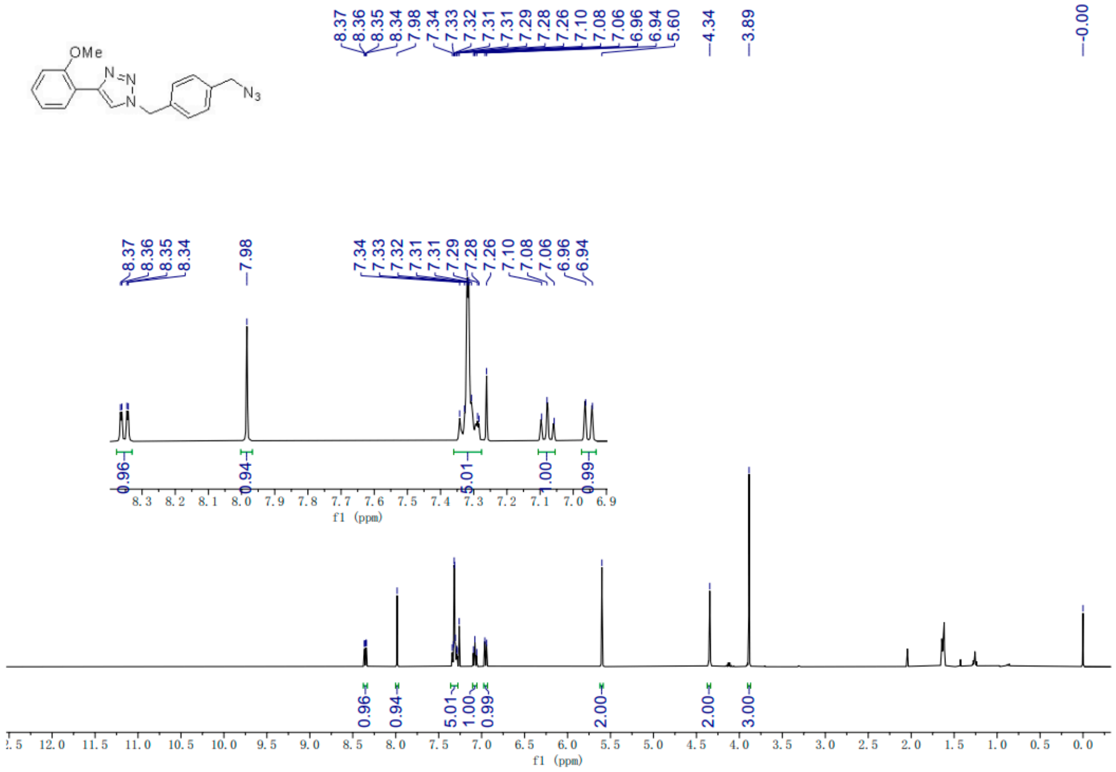

^{13}C NMR (101 MHz, CDCl$_3$) spectrum for 6d

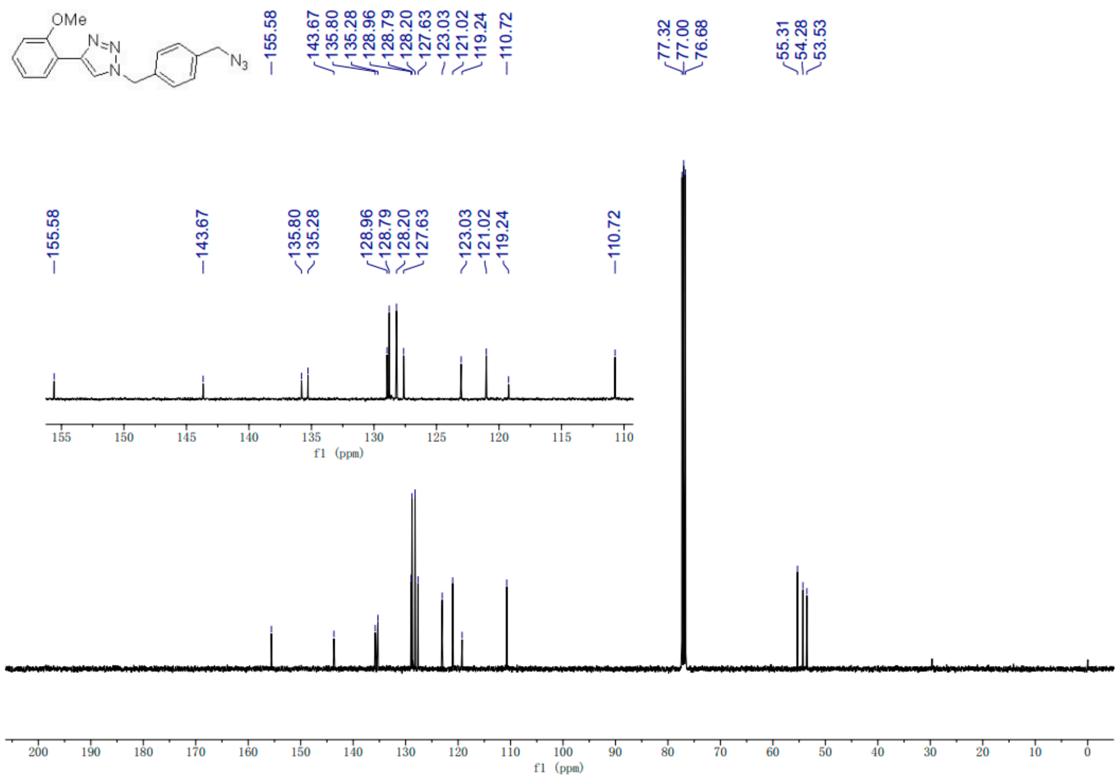

^{1}H NMR (400 MHz, $CDCl_3$) spectrum for 6e

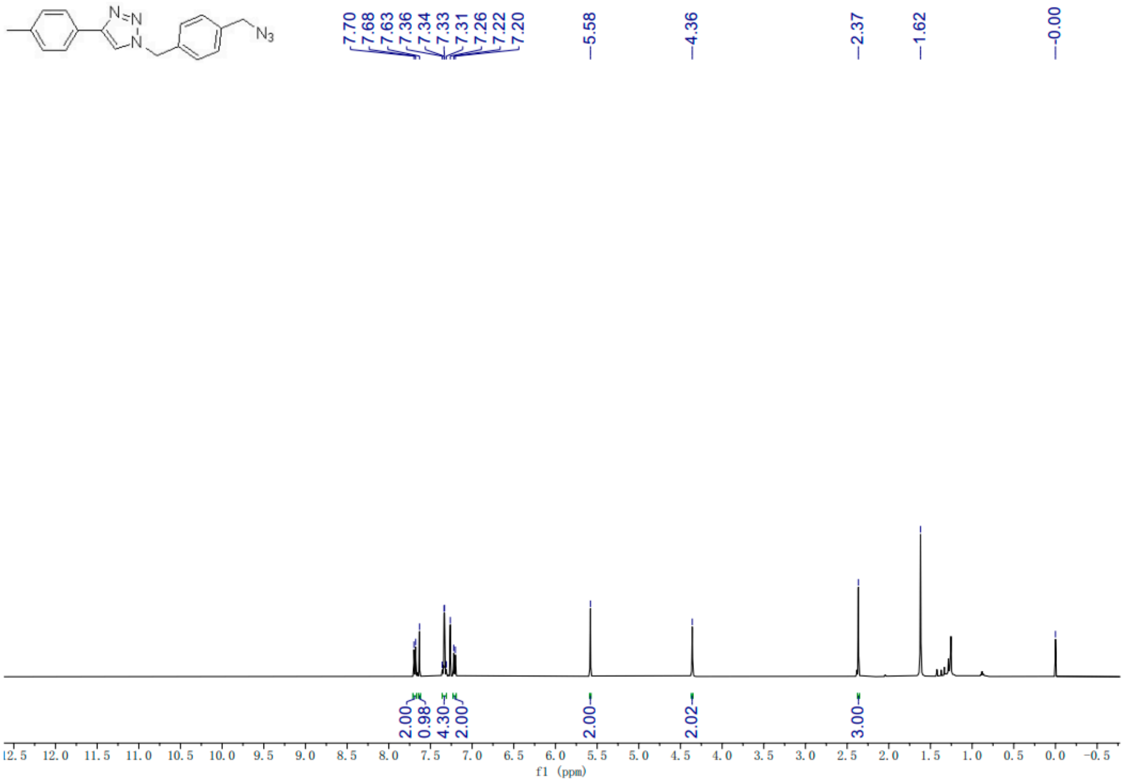

^{13}C NMR (101 MHz, $CDCl_3$) spectrum for 6e

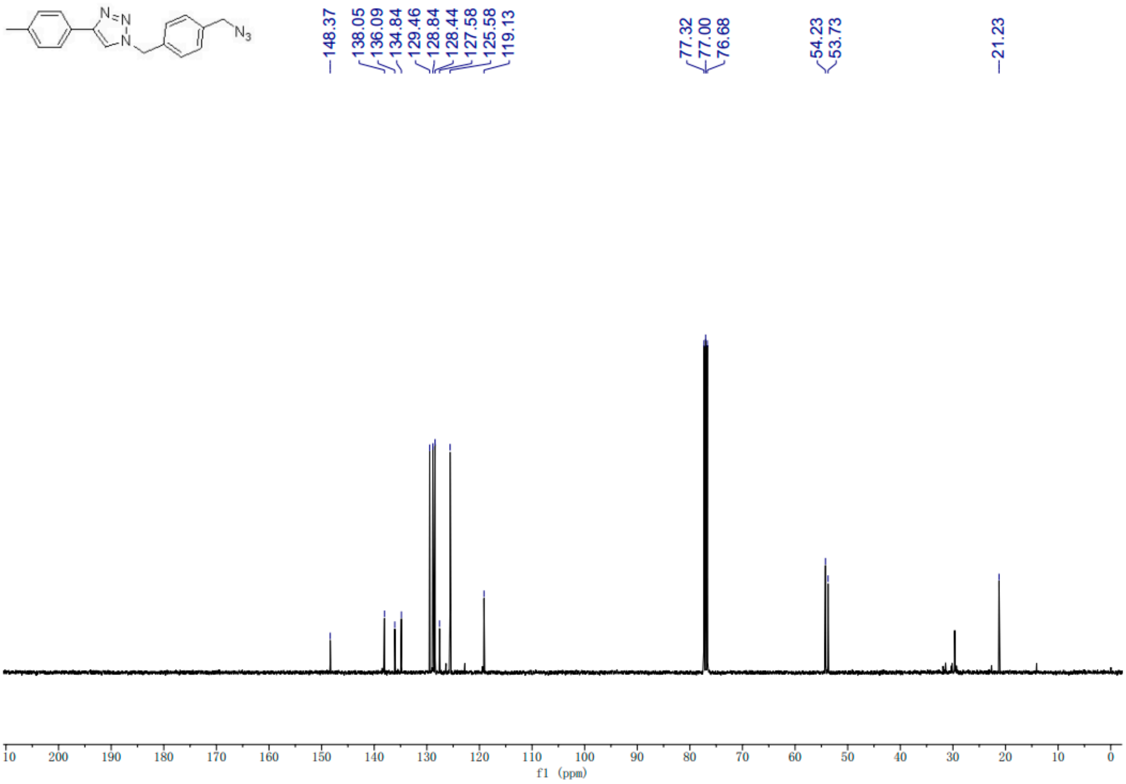

^{1}H NMR (400 MHz, CDCl$_3$) spectrum for 6f

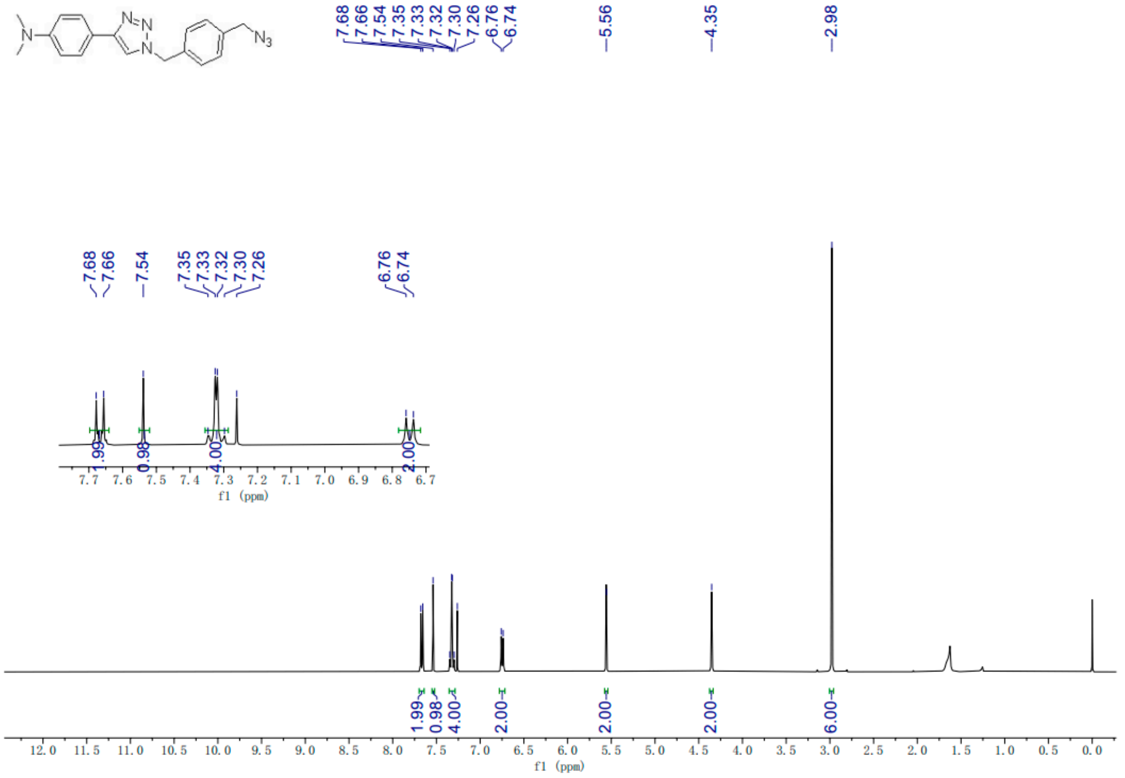

^{13}C NMR (101 MHz, CDCl$_3$) spectrum for 6f

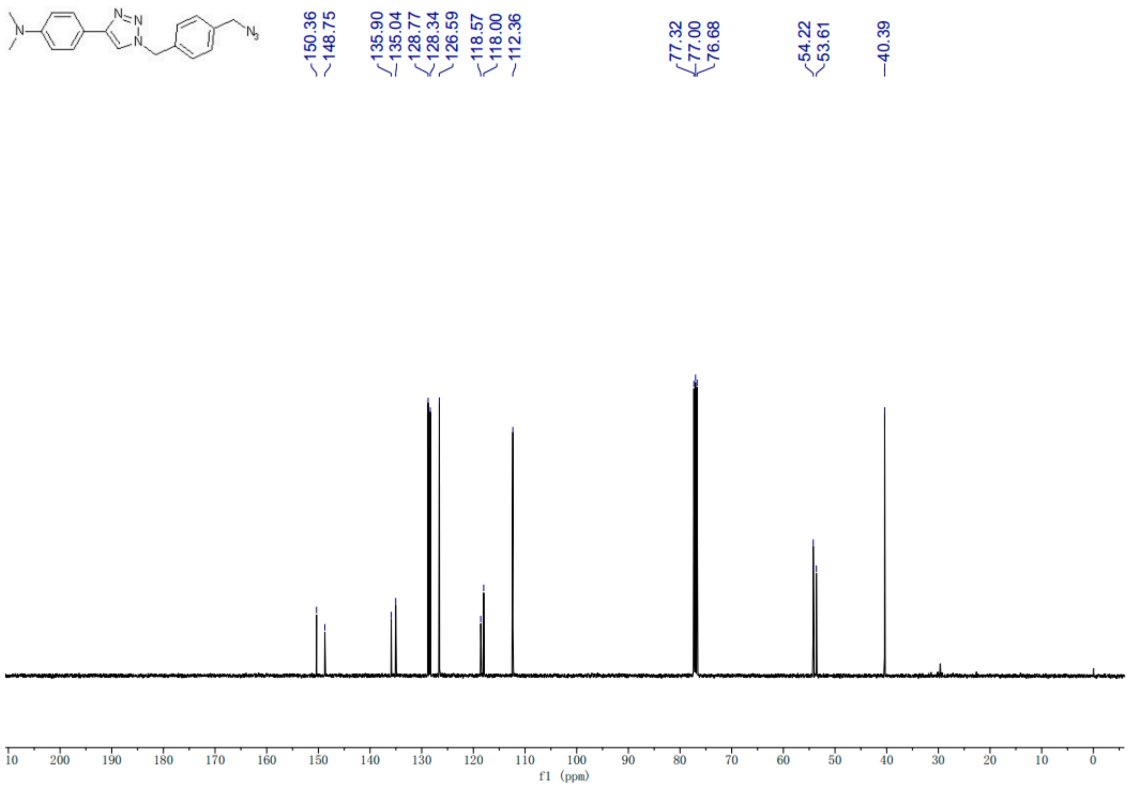

^{1}H NMR (400 MHz, $CDCl_3$) spectrum for 6g

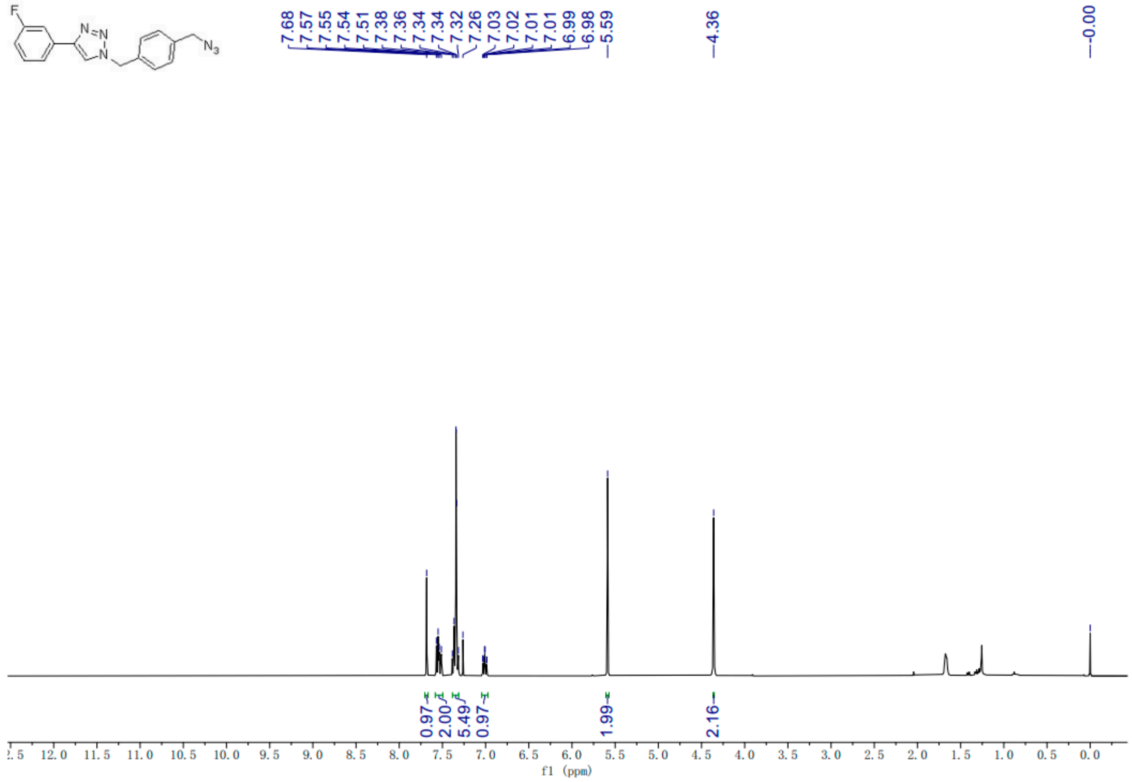

^{13}C NMR (101 MHz, $CDCl_3$) spectrum for 6g

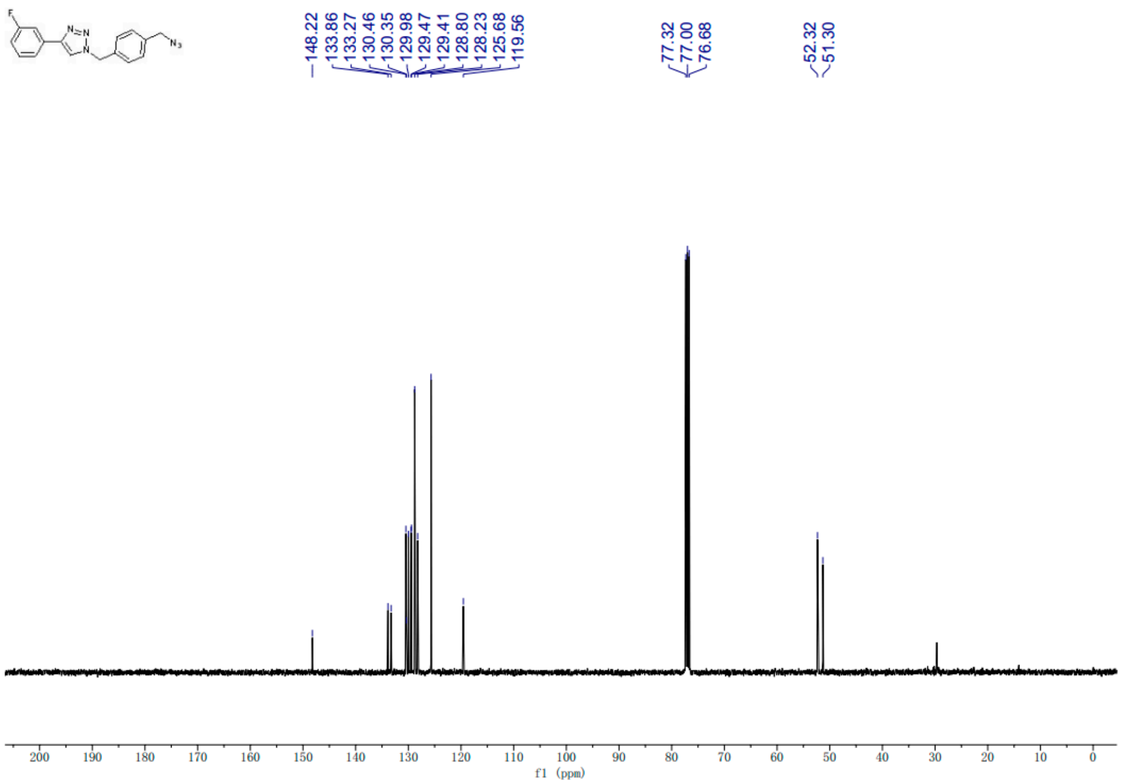

^{19}F NMR (376 MHz, CDCl$_3$) spectrum for 6g

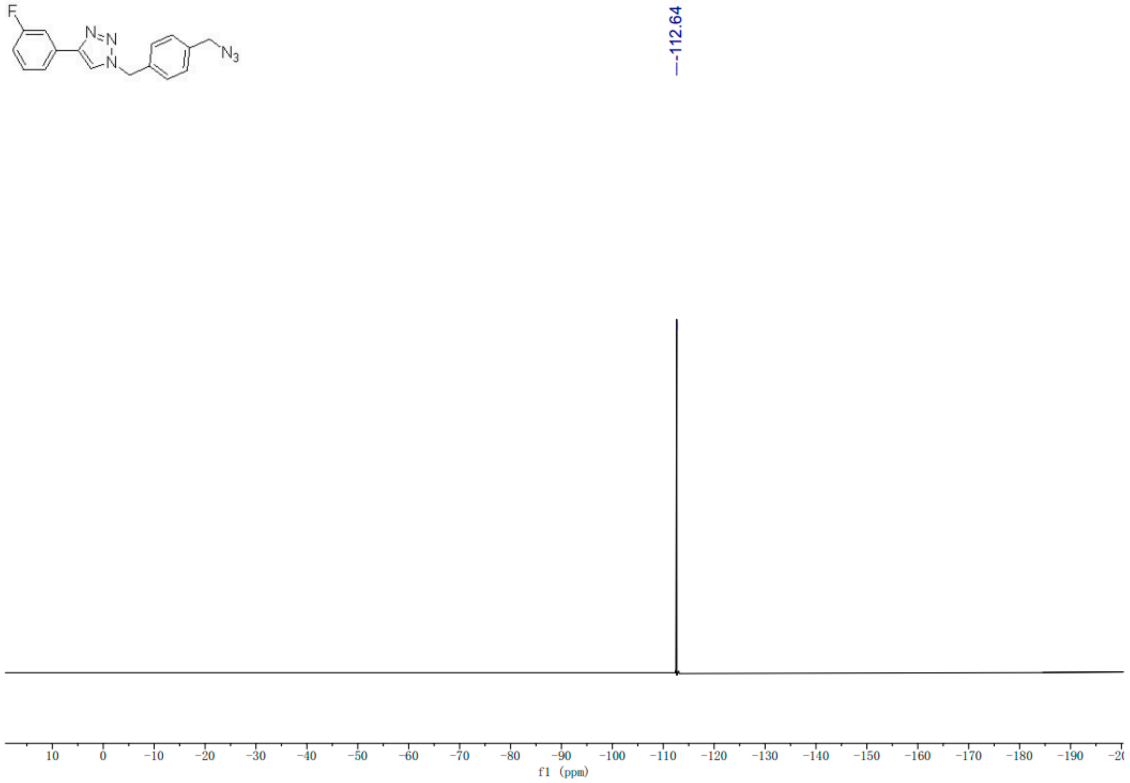

^{1}H NMR (400 MHz, CDCl$_3$) spectrum for 6h

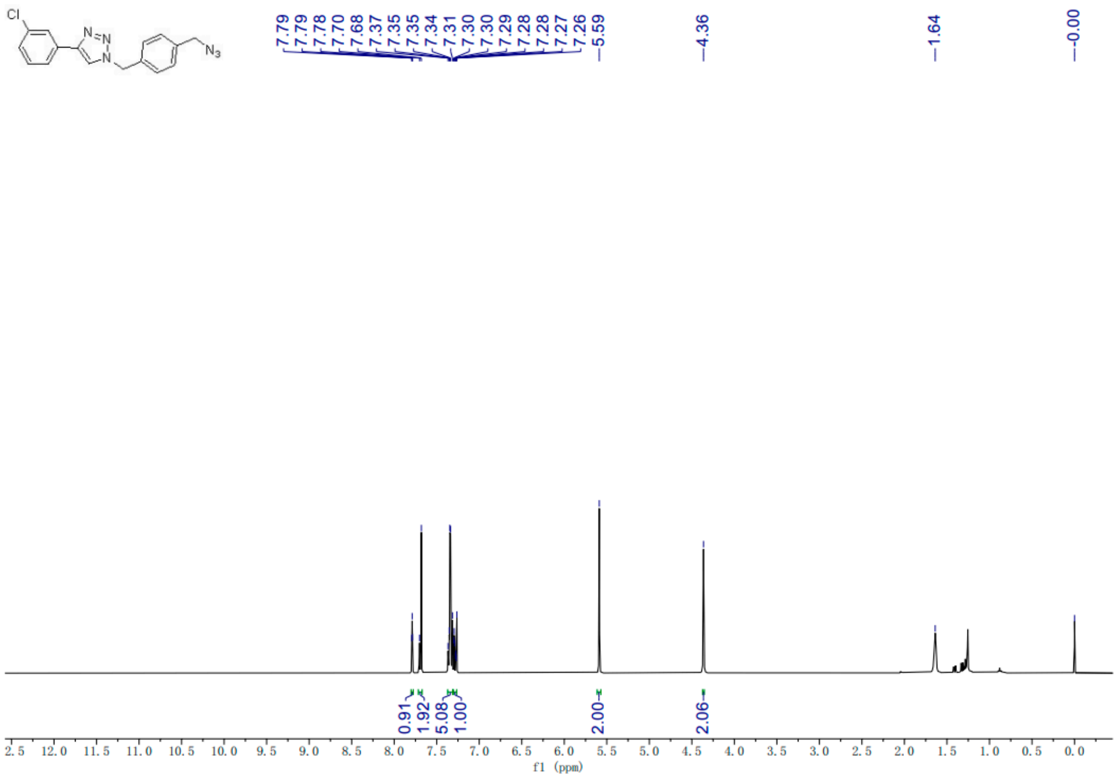

^{13}C NMR (101 MHz, $CDCl_3$) spectrum for 6h

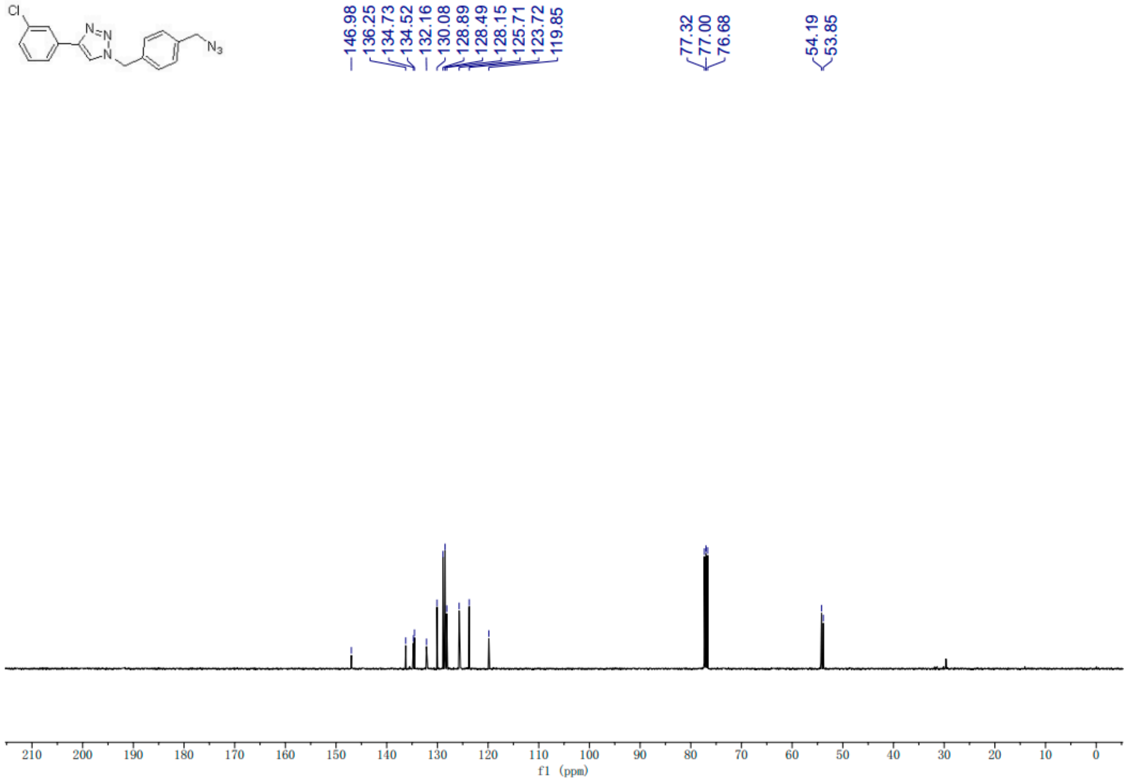

^{1}H NMR (400 MHz, $CDCl_3$) spectrum for 6i

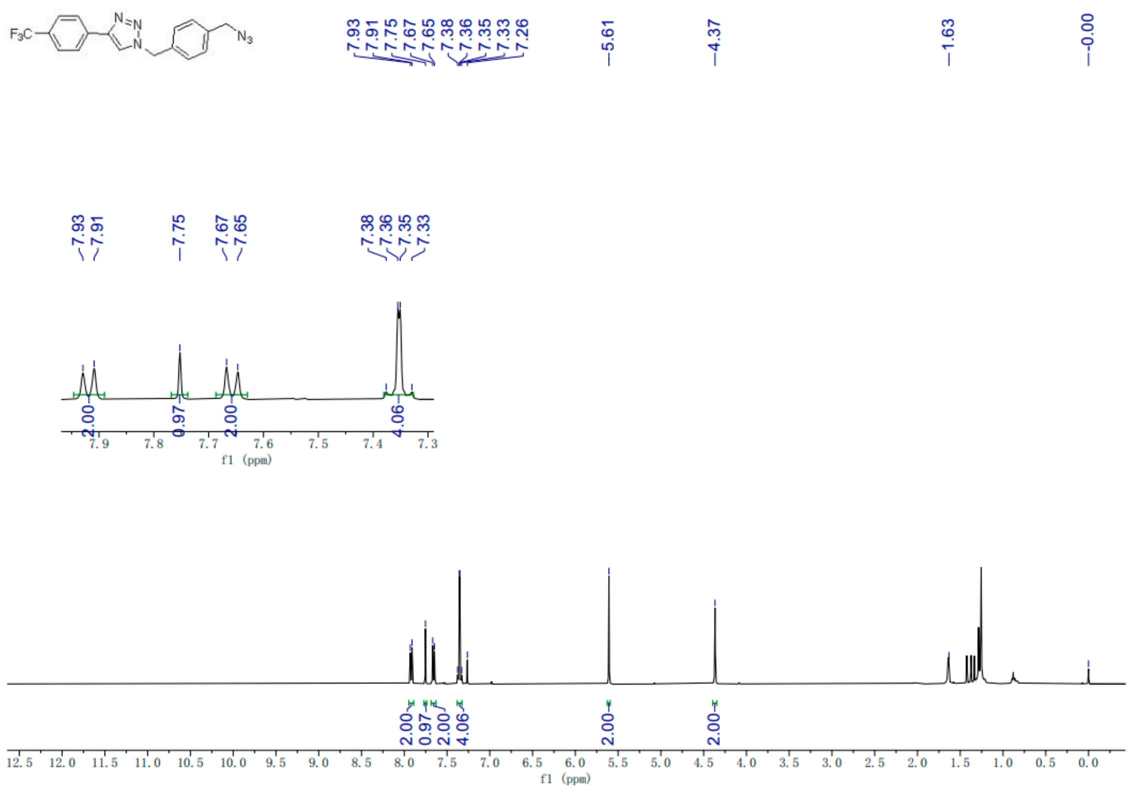

^{13}C NMR (101 MHz, CDCl₃) spectrum for 6i

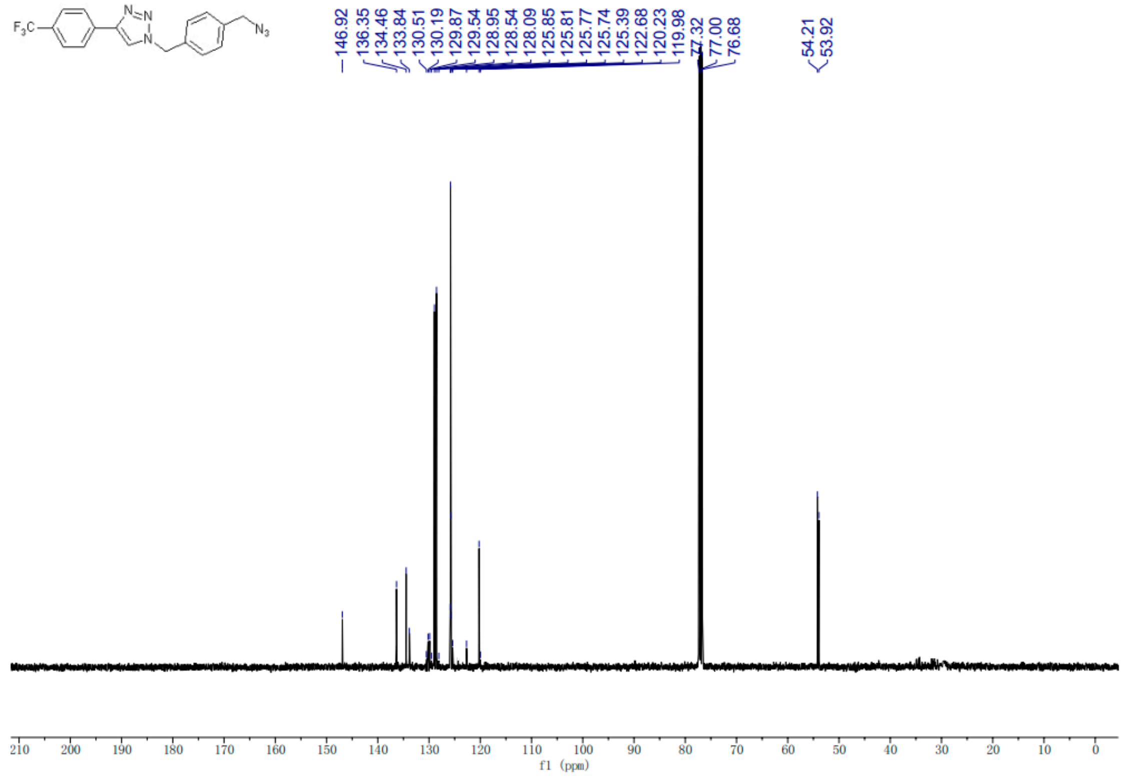

^{19}F NMR (376 MHz, CDCl₃) spectrum for 6i

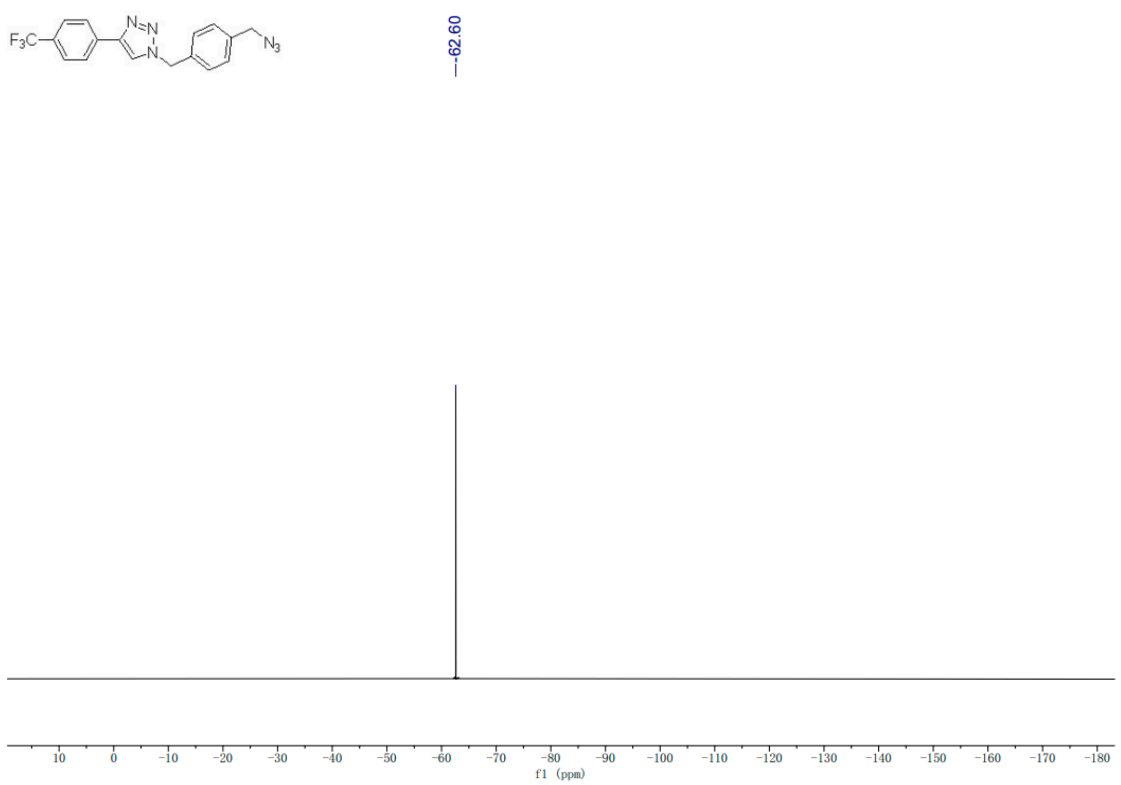

^{1}H NMR (400 MHz, $CDCl_3$) spectrum for 6j

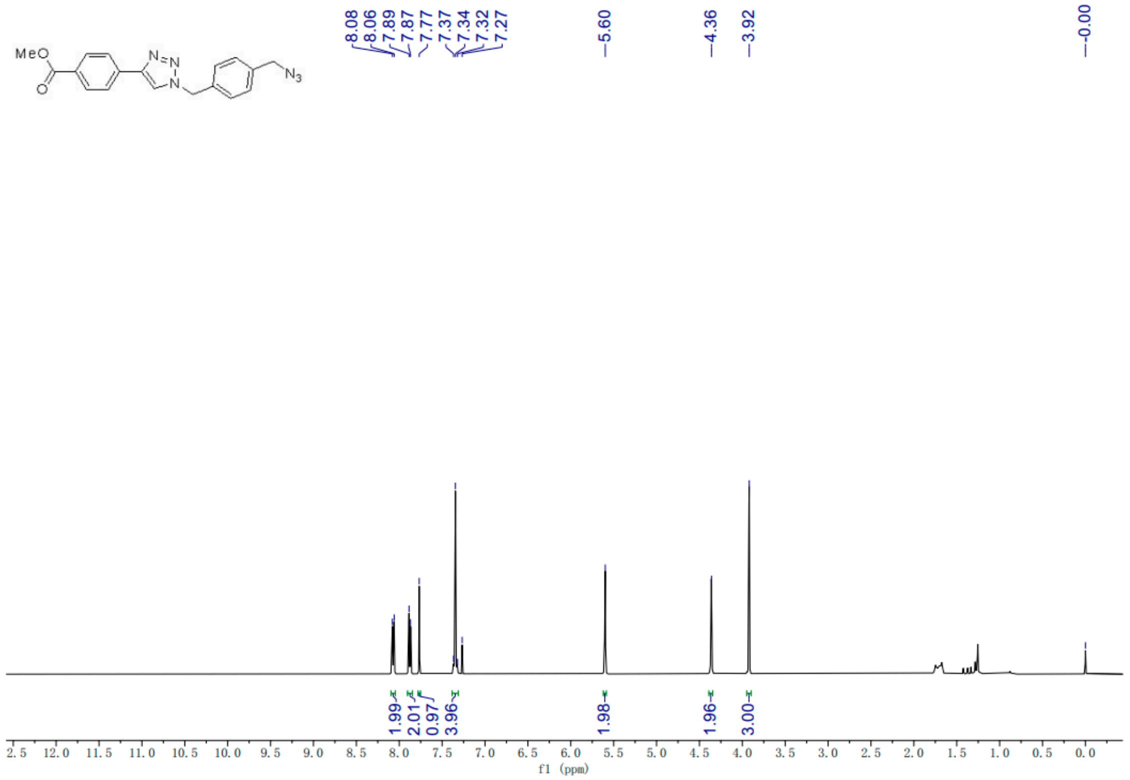

^{13}C NMR (101 MHz, $CDCl_3$) spectrum for 6j

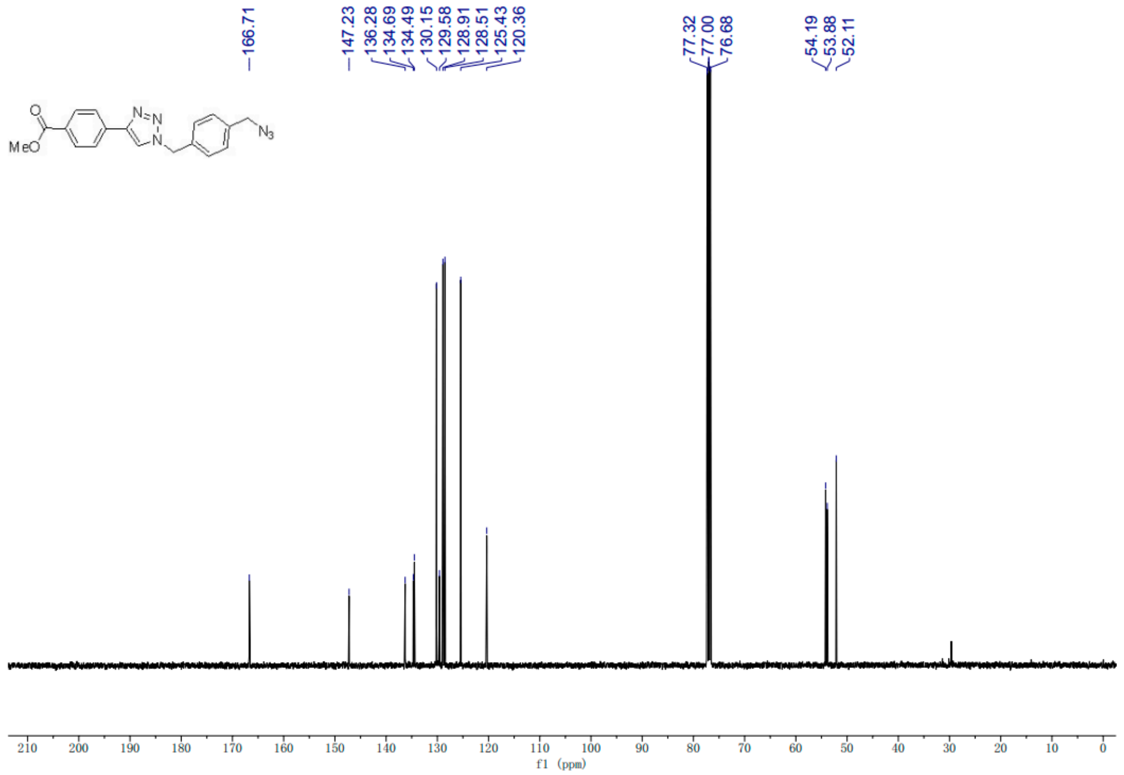

^{1}H NMR (400 MHz, $CDCl_3$) spectrum for 6k

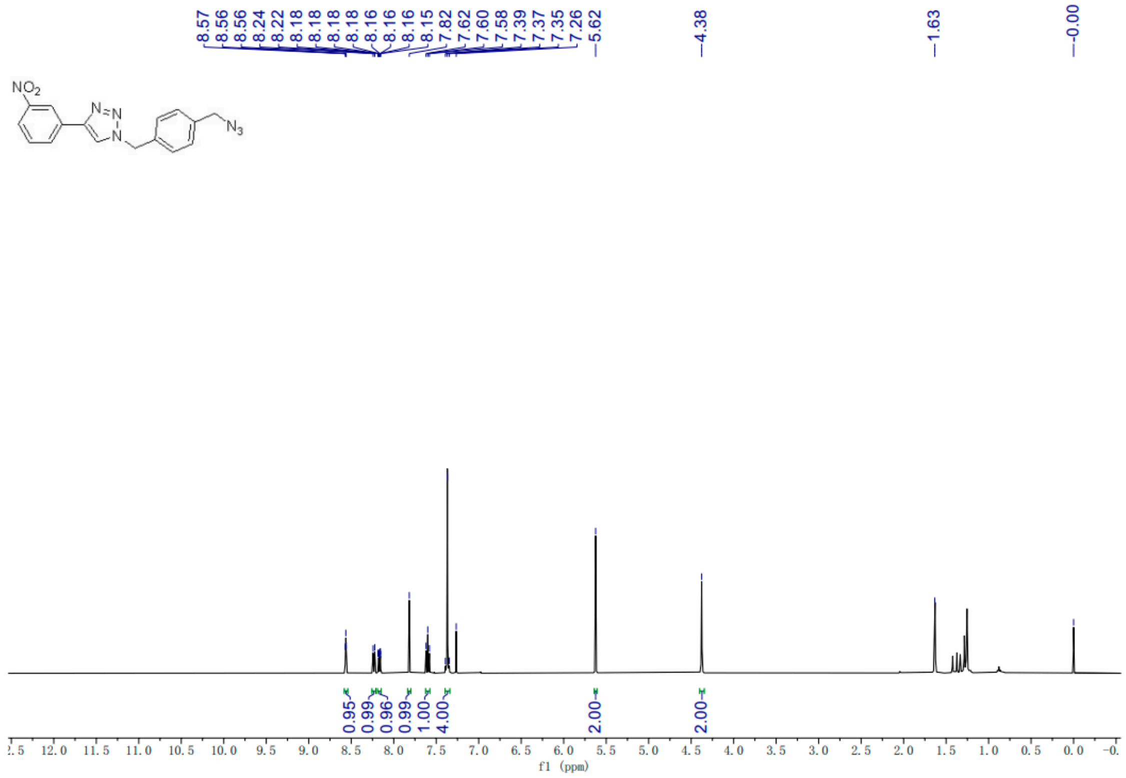

^{13}C NMR (101 MHz, $CDCl_3$) spectrum for 6k

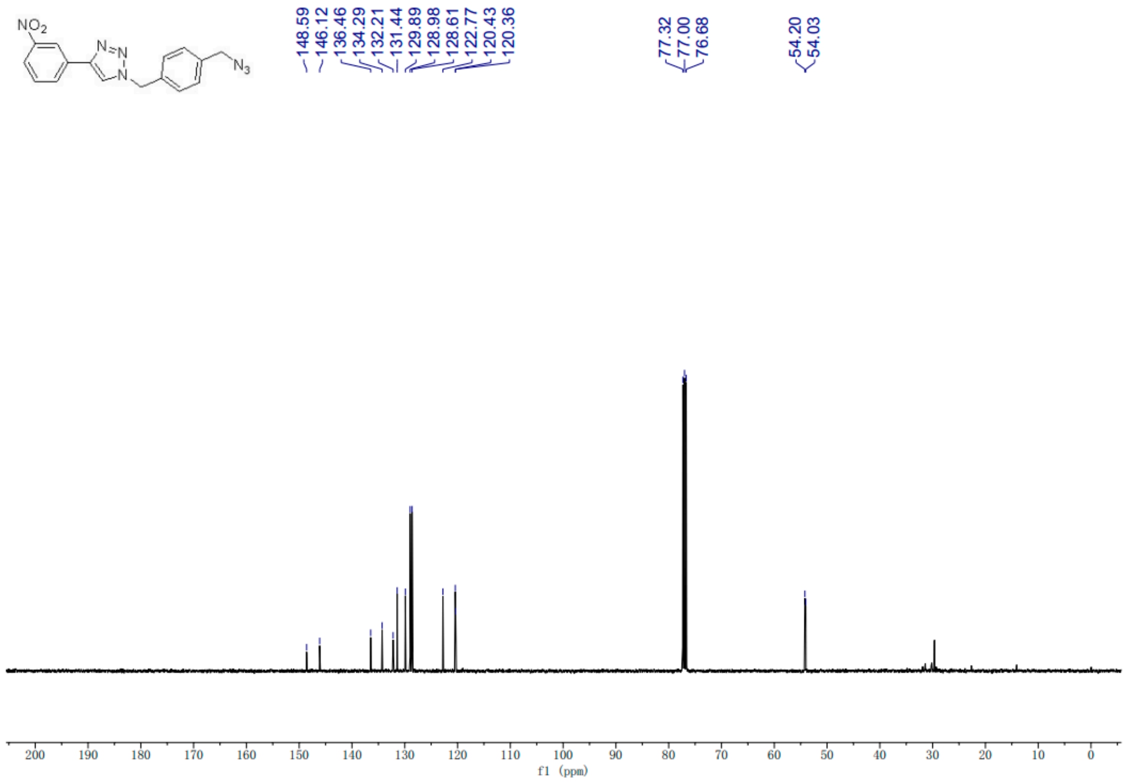

^{1}H NMR (400 MHz, $CDCl_3$) spectrum for 6l

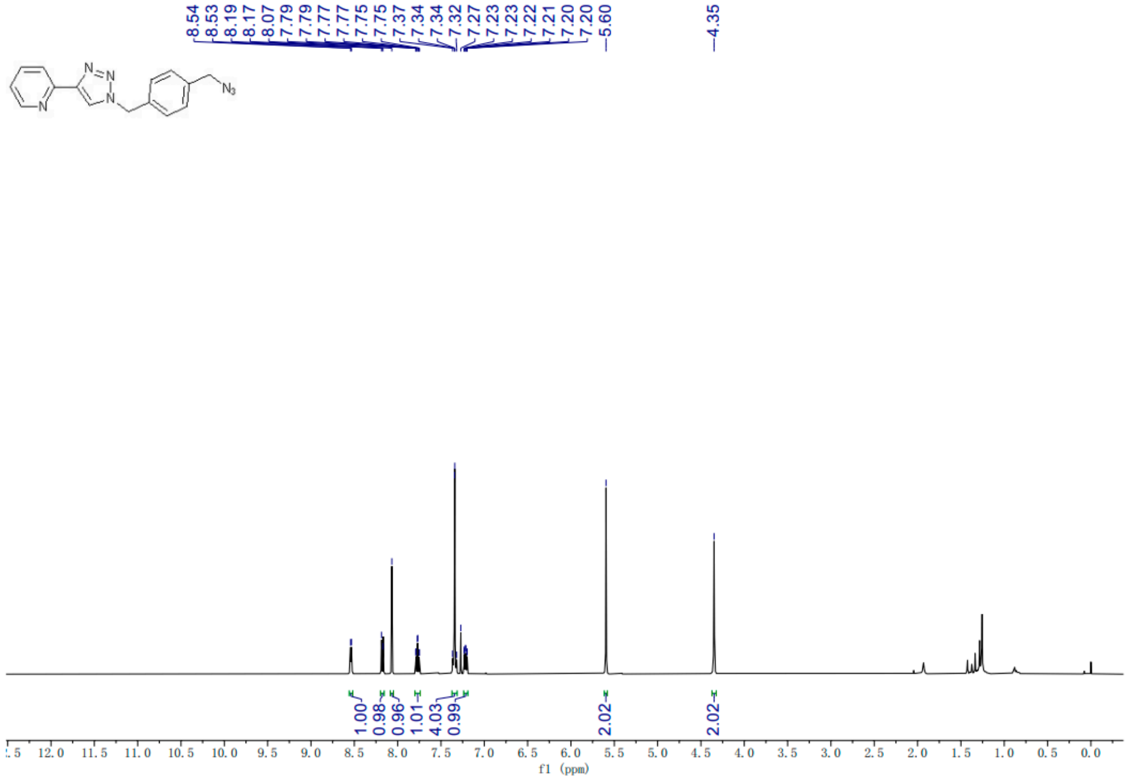

^{13}C NMR (101 MHz, $CDCl_3$) spectrum for 6l

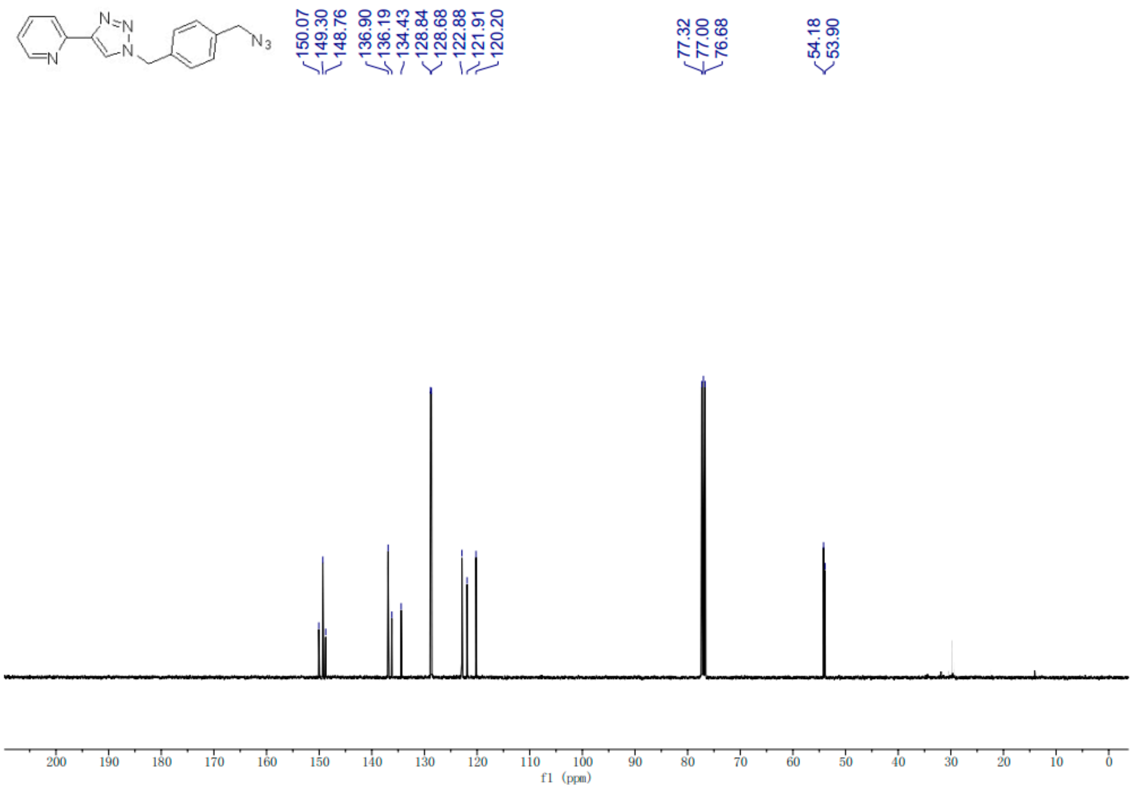

^{1}H NMR (400 MHz, CDCl$_3$) spectrum for 6m

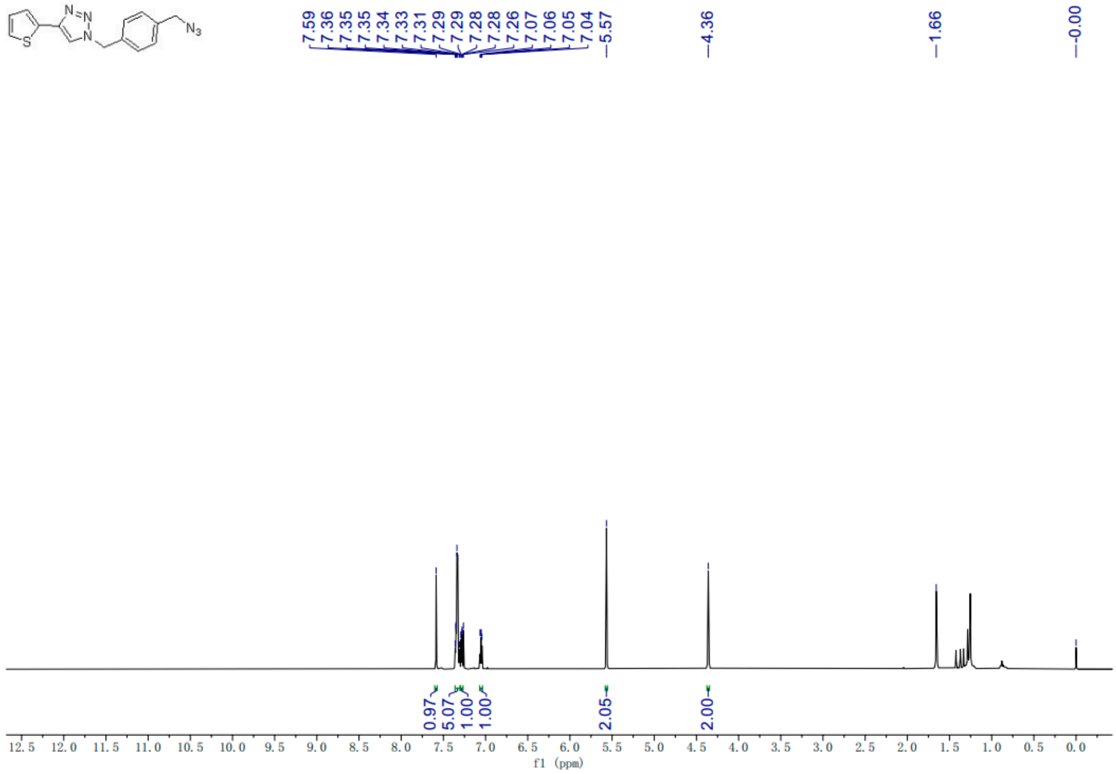

^{13}C NMR (101 MHz, CDCl$_3$) spectrum for 6m

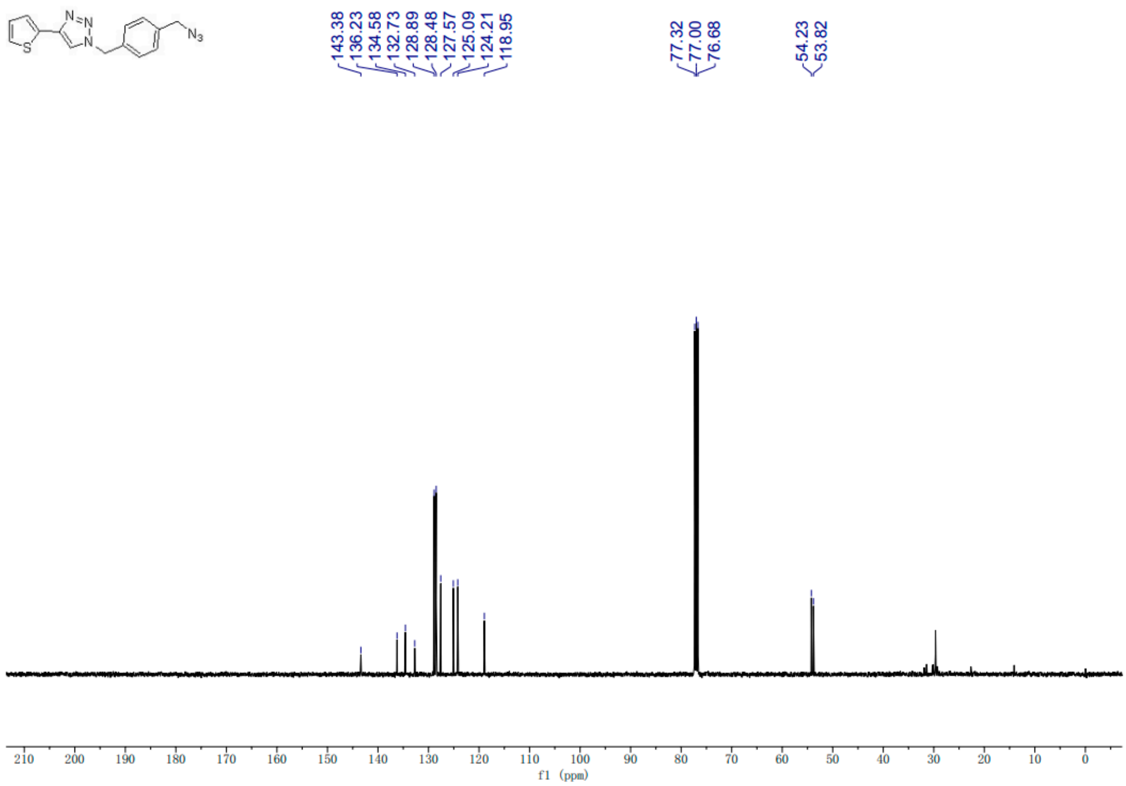

^{1}H NMR (400 MHz, $CDCl_3$) spectrum for 6n

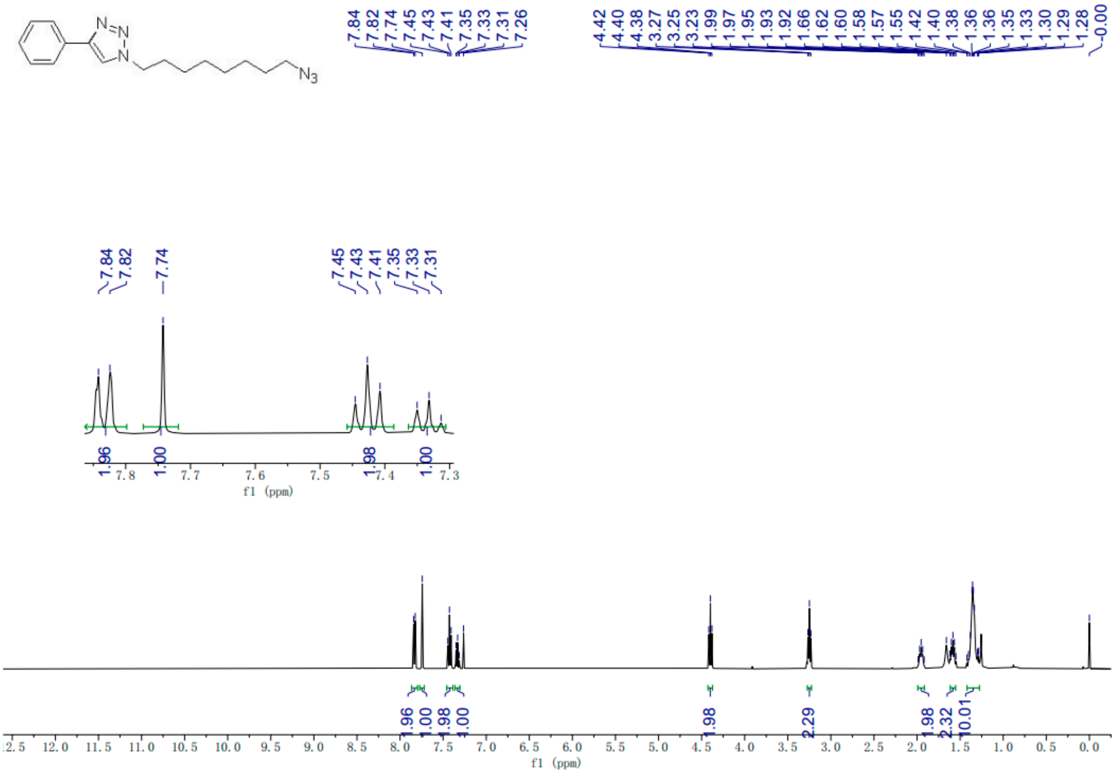

^{13}C NMR (101 MHz, $CDCl_3$) spectrum for 6n

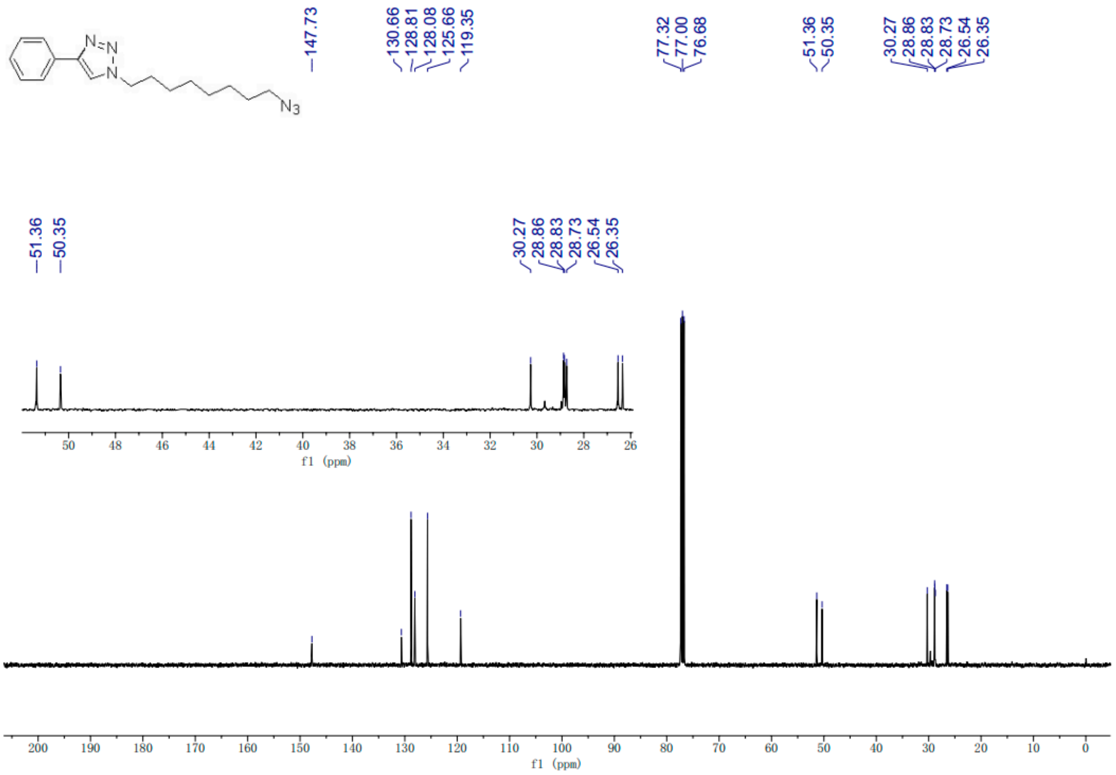

^{1}H NMR (400 MHz, $CDCl_3$) spectrum for 7a

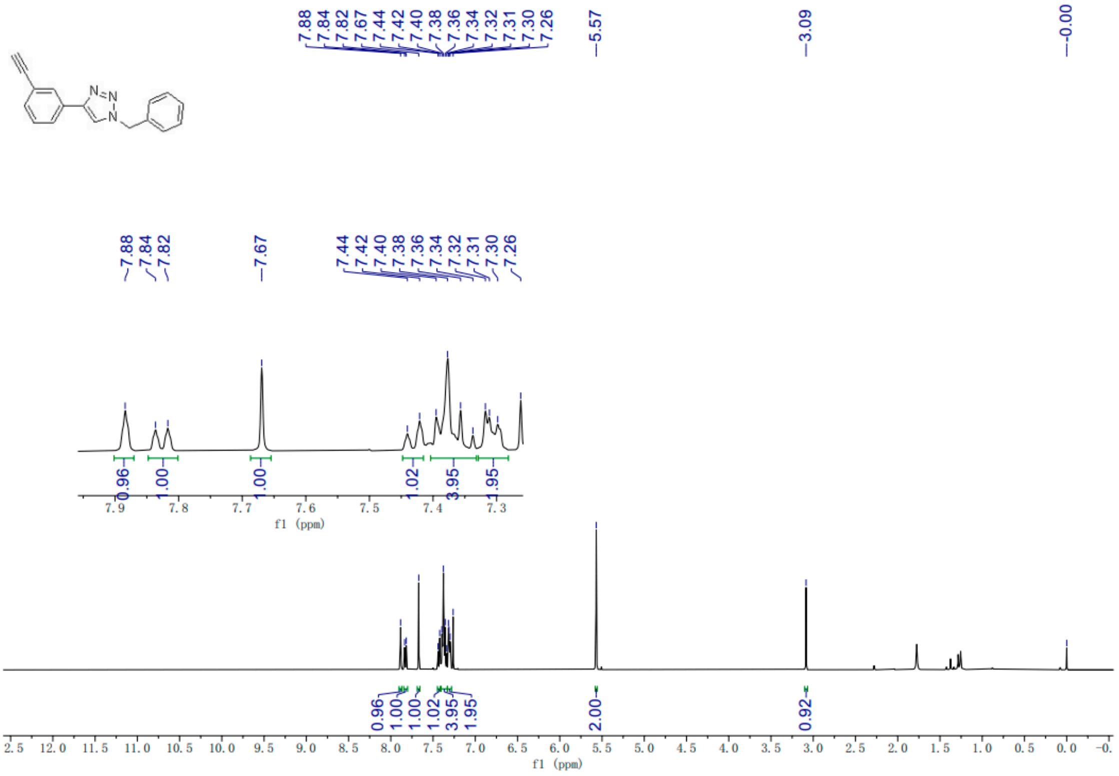

^{13}C NMR (101 MHz, $CDCl_3$) spectrum for 7a

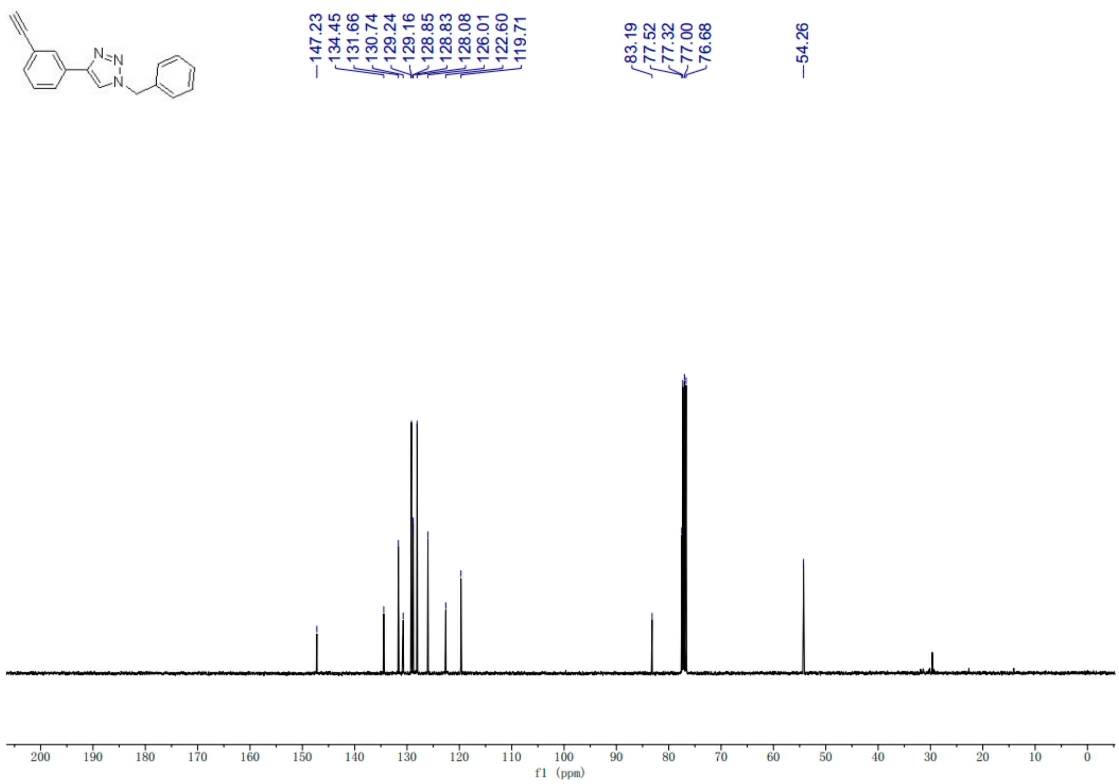

^{1}H NMR (400 MHz, CDCl$_3$) spectrum for 7b

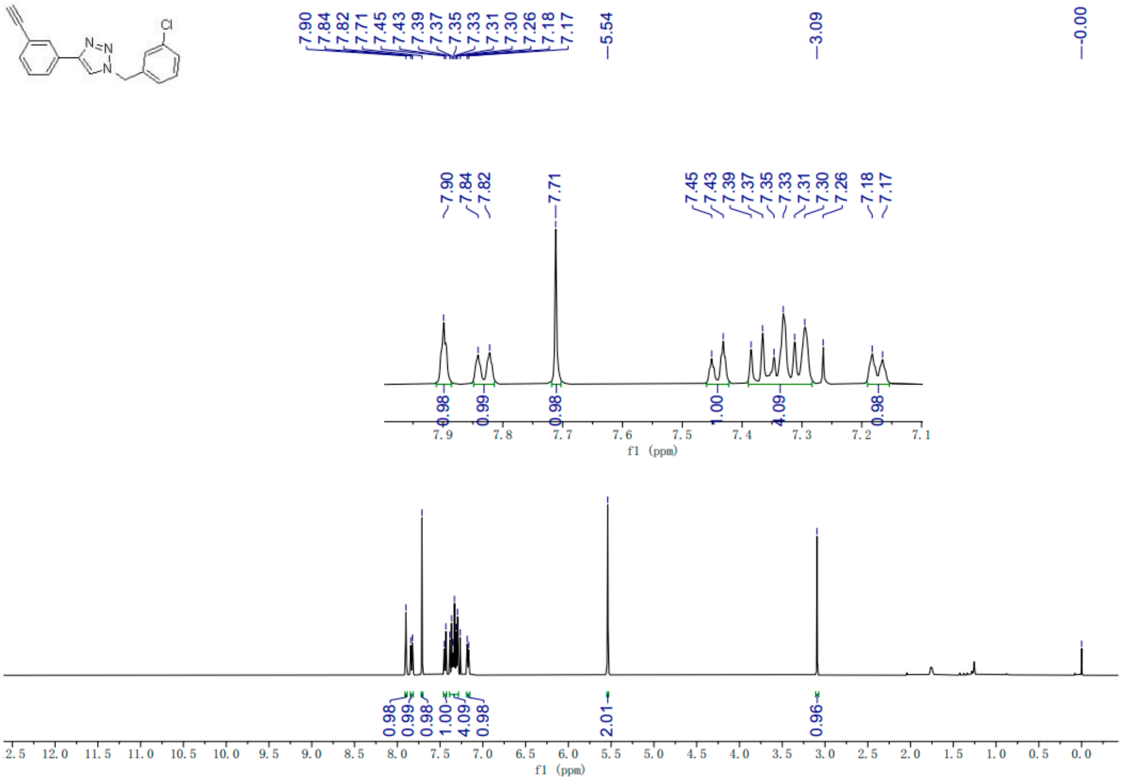

^{13}C NMR (101 MHz, CDCl$_3$) spectrum for 7b

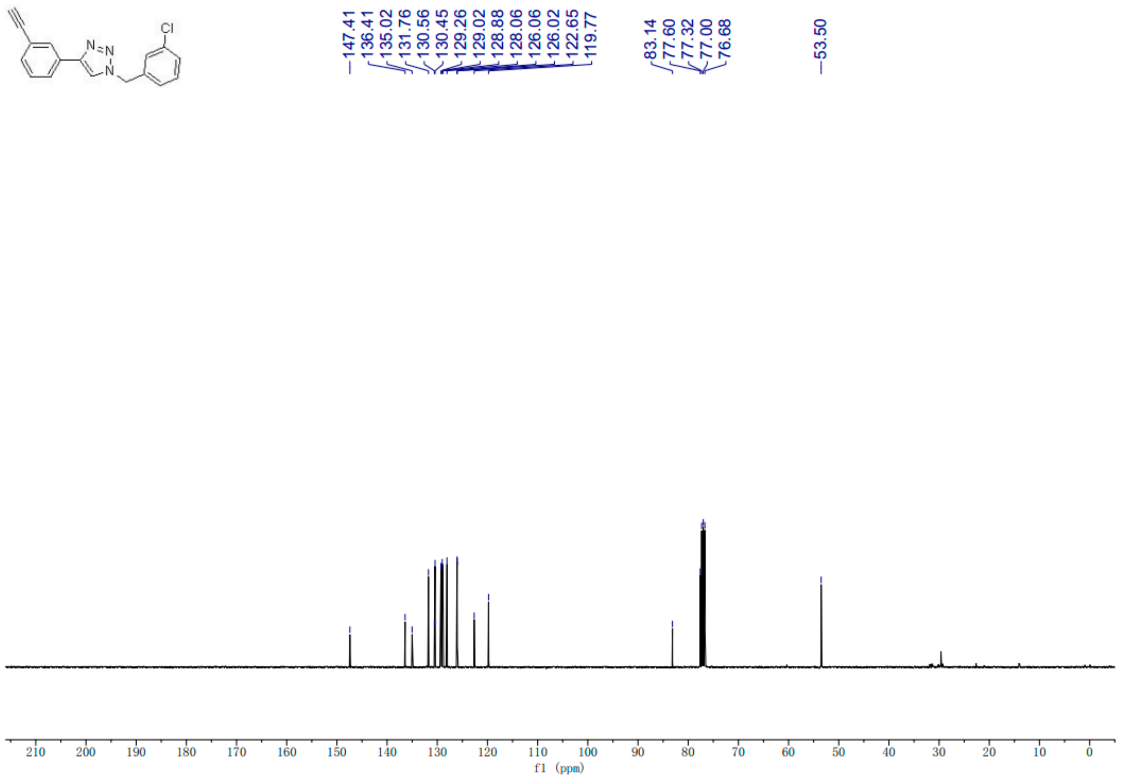

^{1}H NMR (400 MHz, CDCl$_3$) spectrum for 7c

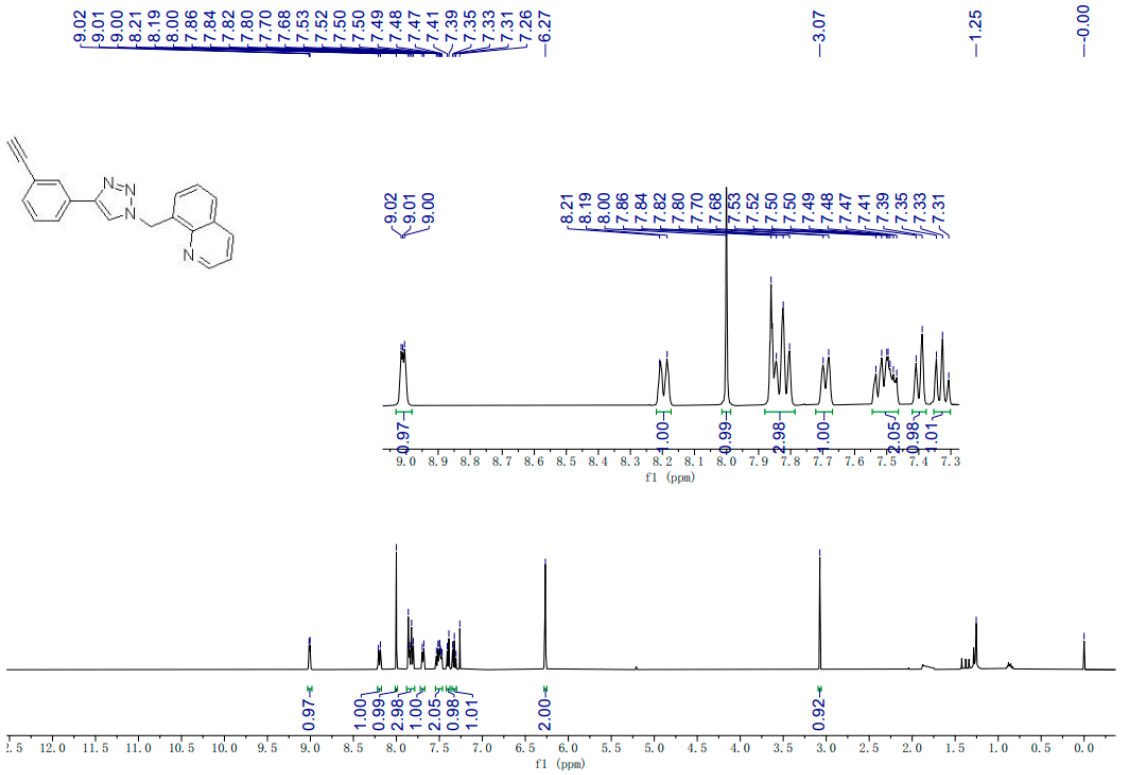

^{13}C NMR (101 MHz, CDCl$_3$) spectrum for 7c

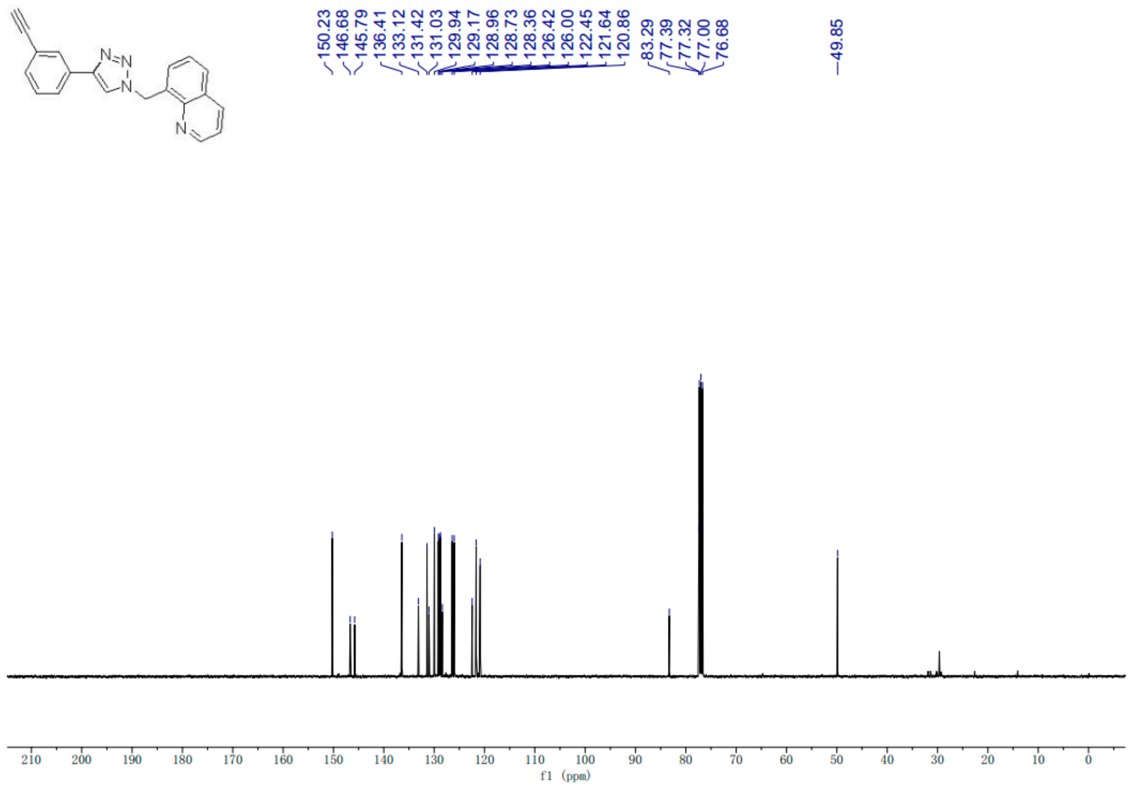

^{1}H NMR (400 MHz, CDCl3) spectrum for 7d

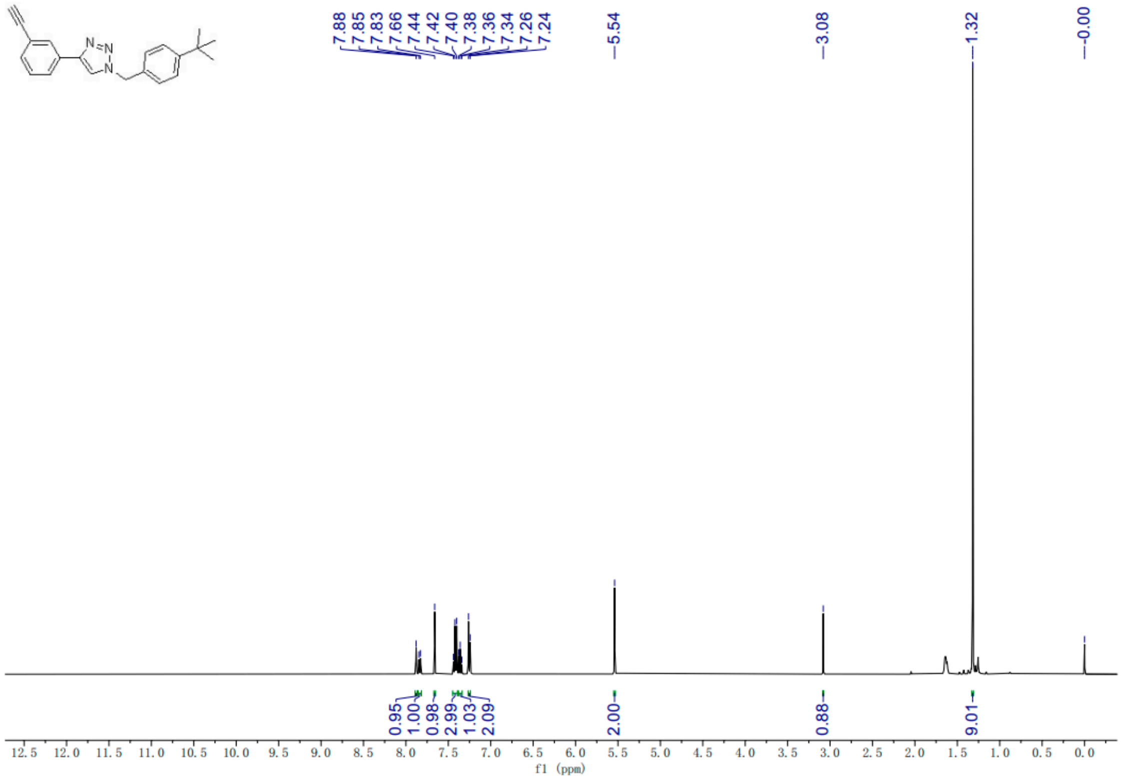

^{13}C NMR (101 MHz, CDCl3) spectrum for 7d

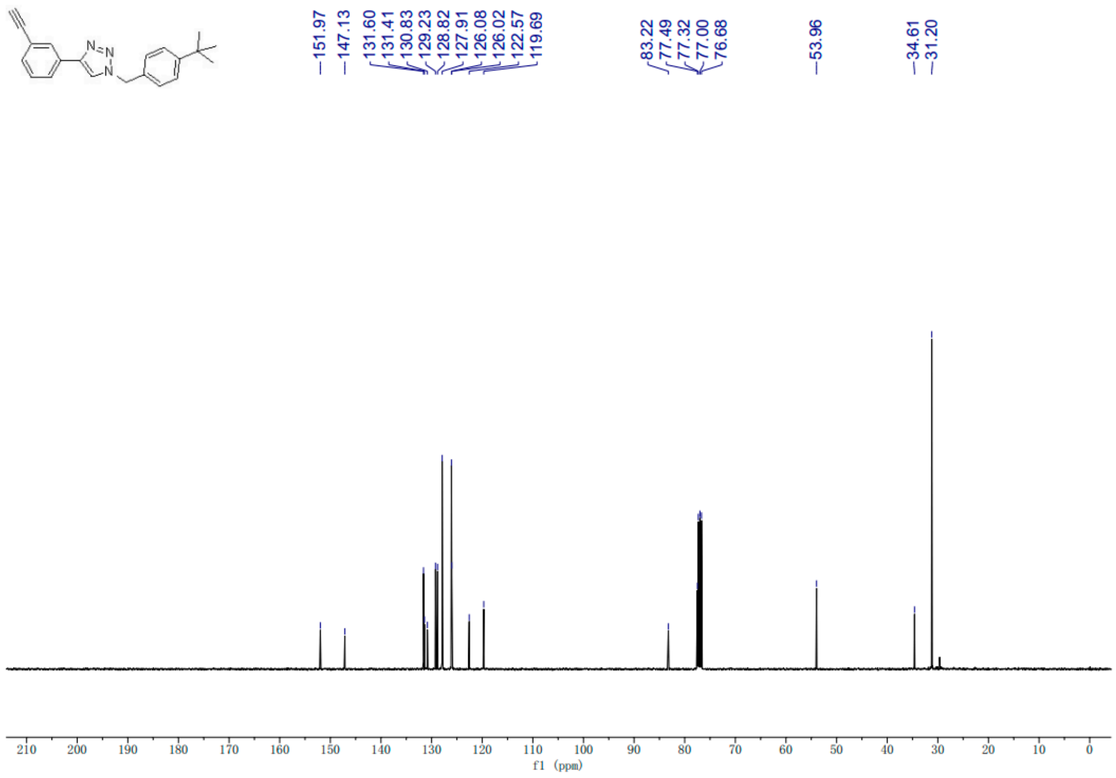

^{1}H NMR (400 MHz, $CDCl_3$) spectrum for 7e

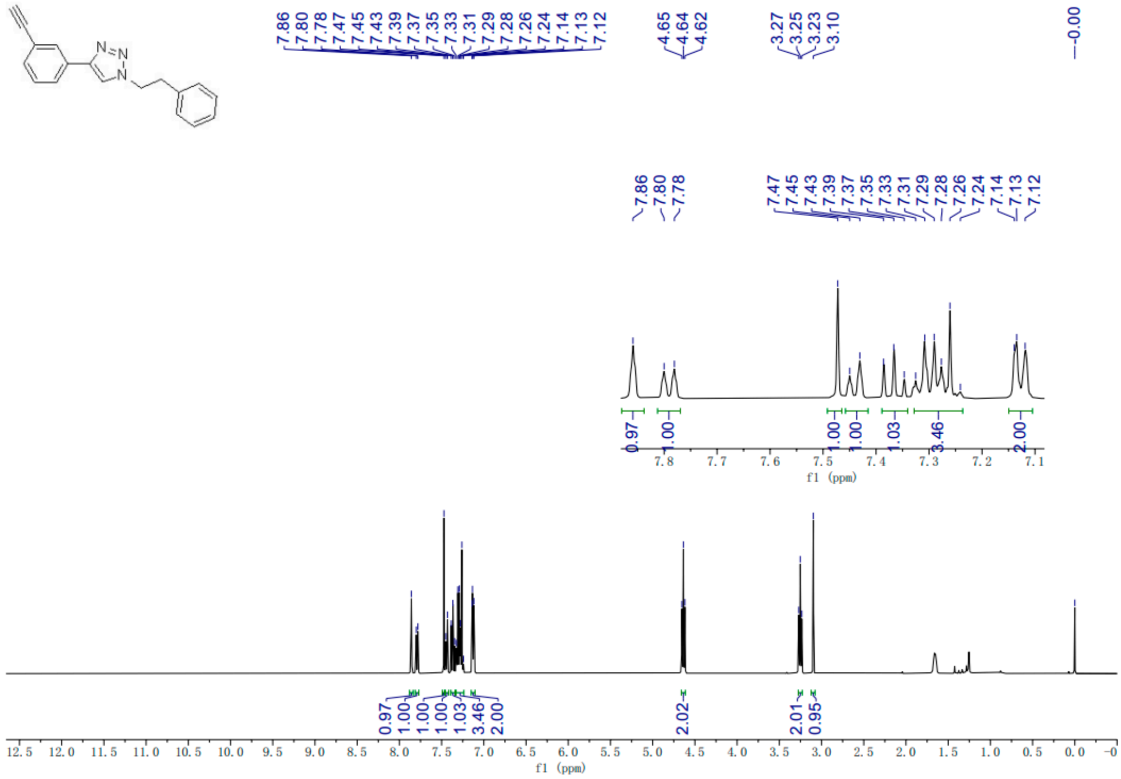

^{13}C NMR (101 MHz, $CDCl_3$) spectrum for 7e

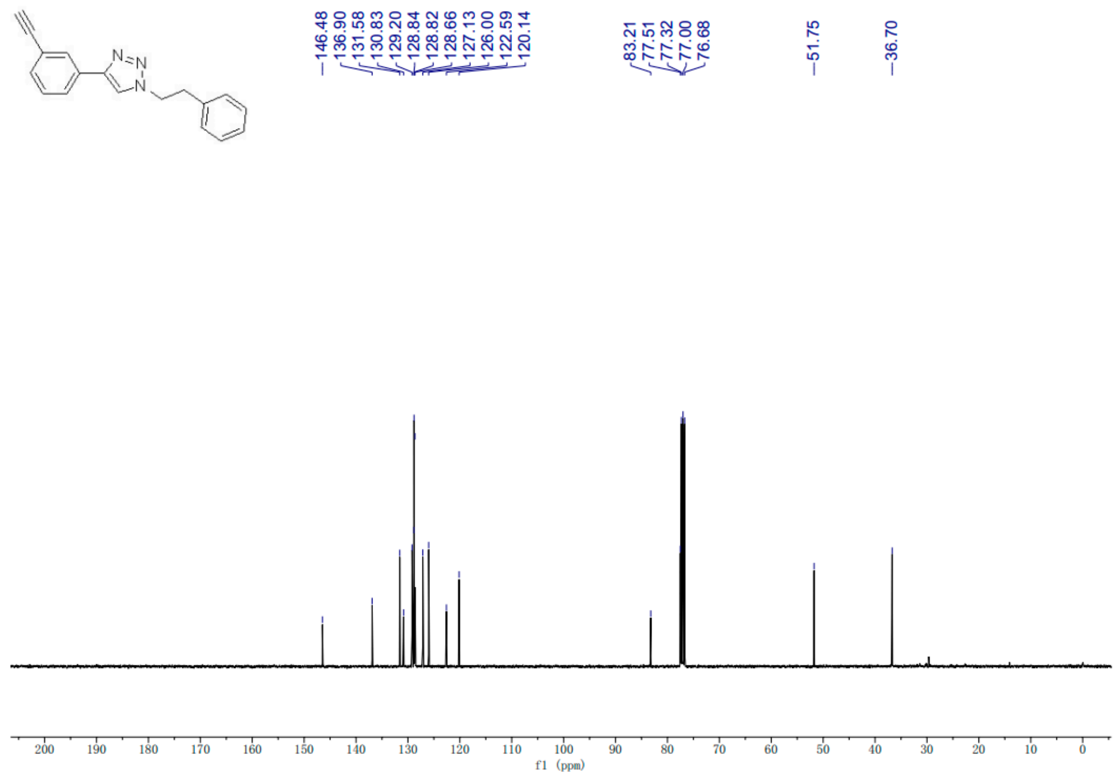

^{1}H NMR (400 MHz, CDCl$_3$) spectrum for 7f

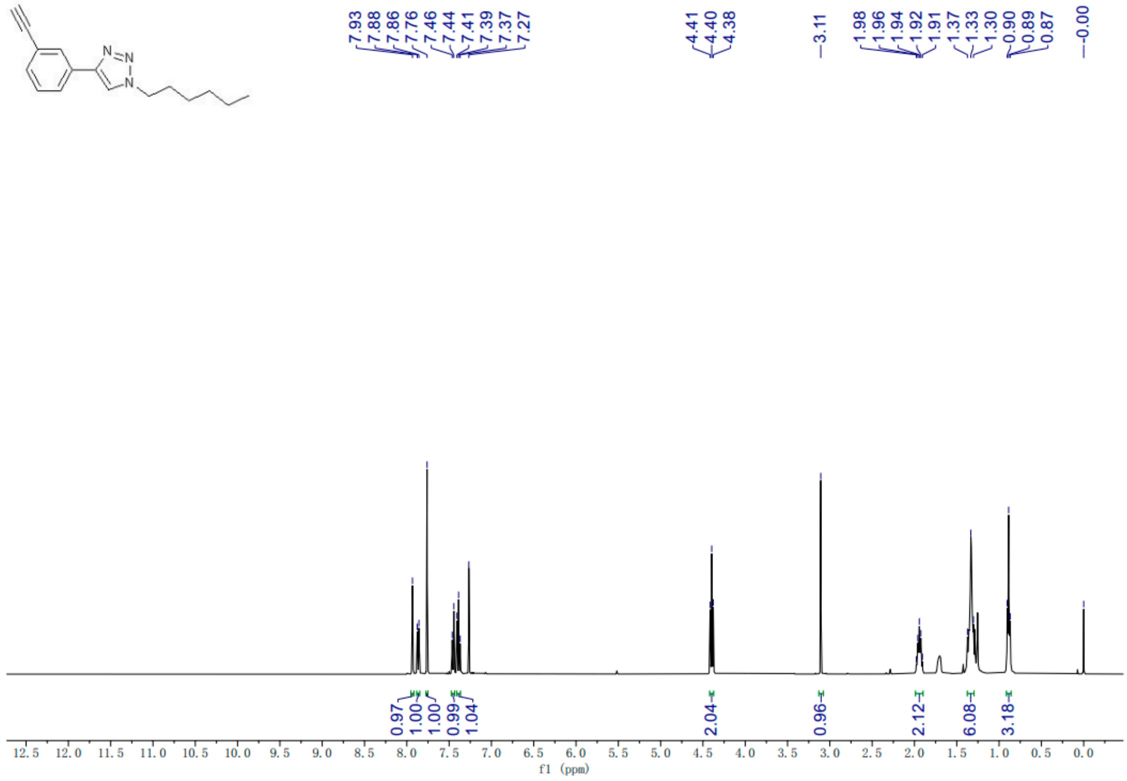

^{13}C NMR (101 MHz, CDCl$_3$) spectrum for 7f

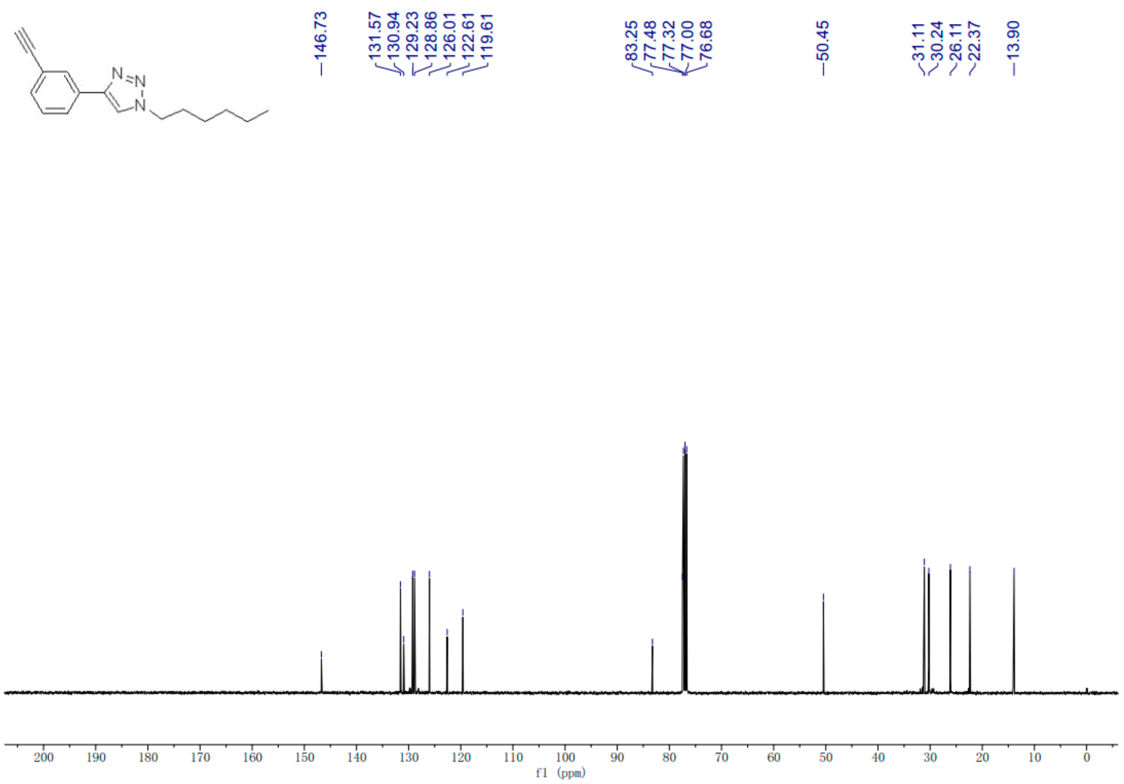

^{1}H NMR (400 MHz, $CDCl_3$) spectrum for 7g

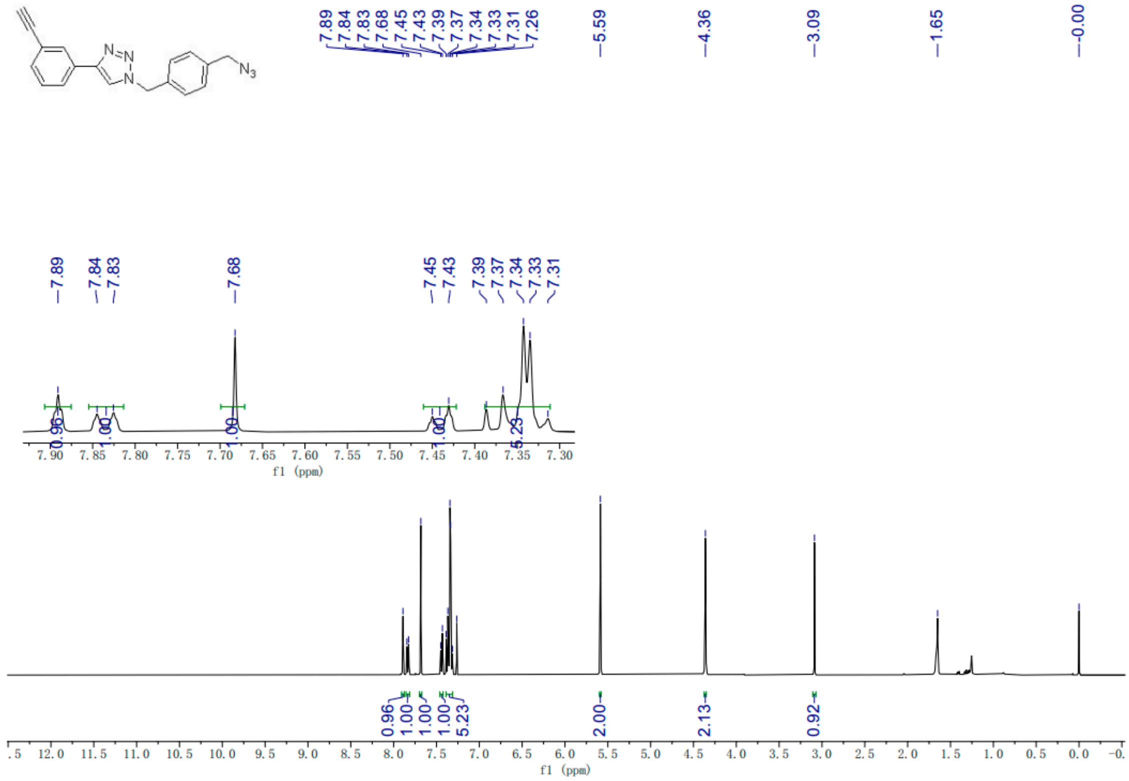

^{13}C NMR (101 MHz, $CDCl_3$) spectrum for 7g

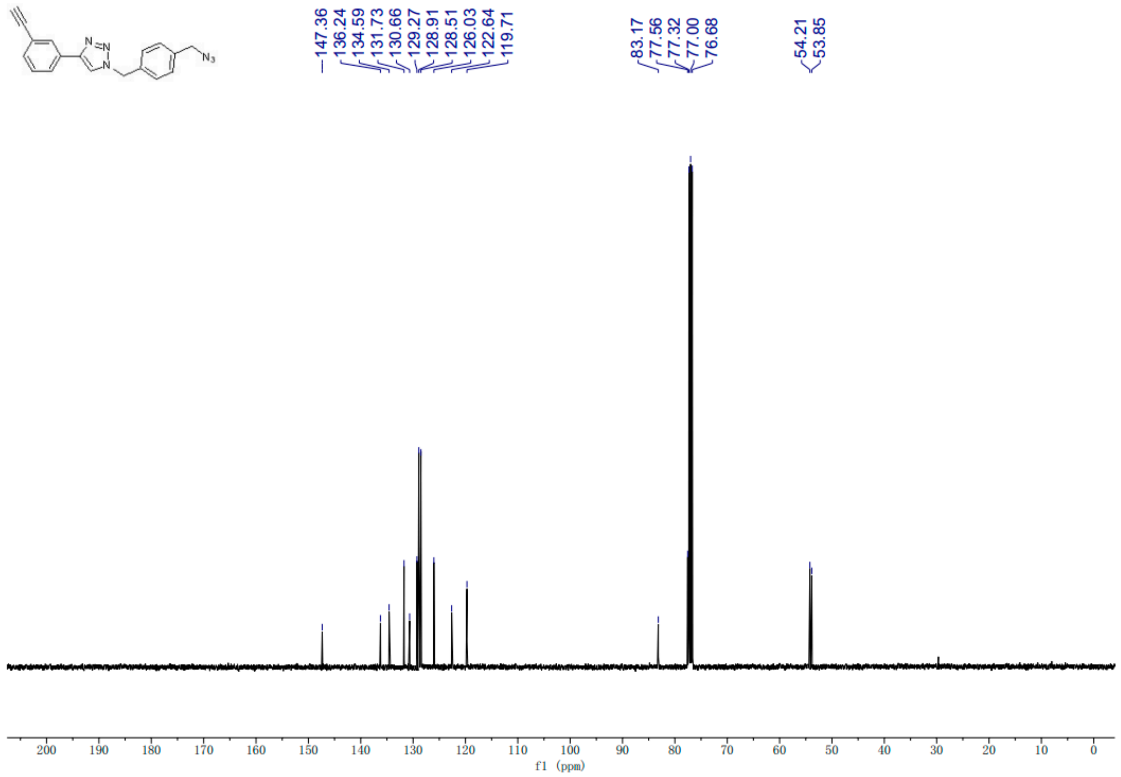

^{1}H NMR (400 MHz, CDCl$_3$) spectrum for 7h

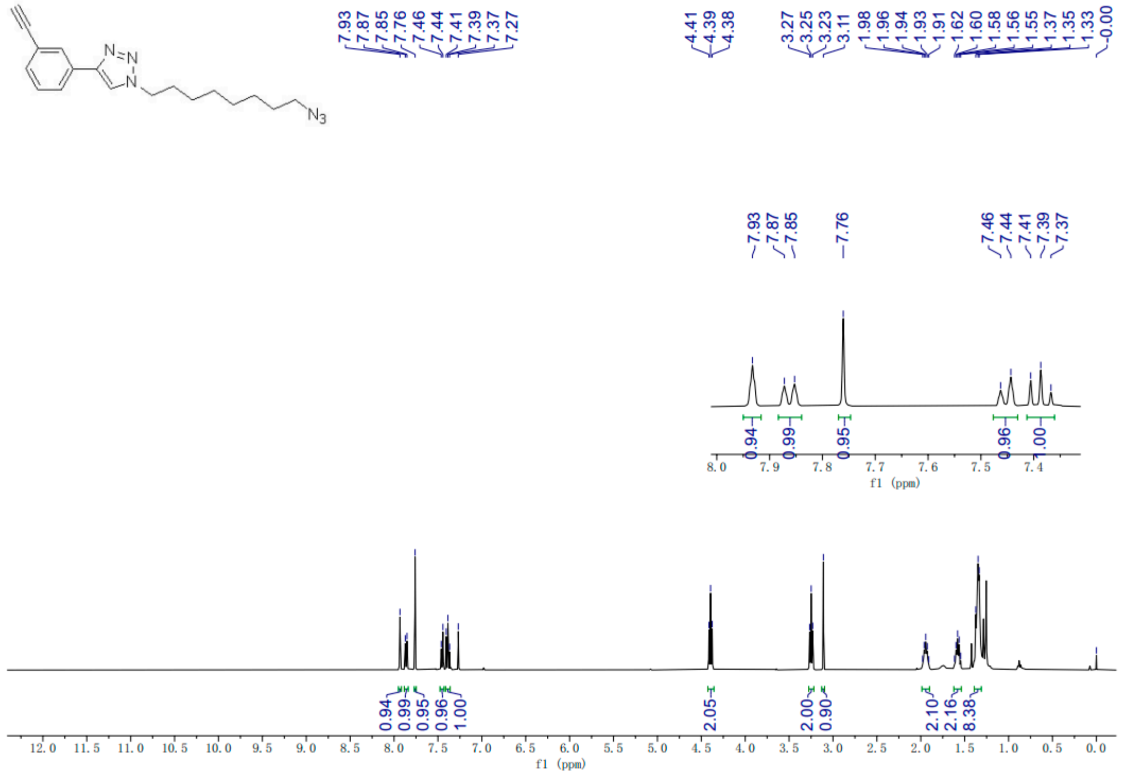

^{13}C NMR (101 MHz, CDCl$_3$) spectrum for 7h

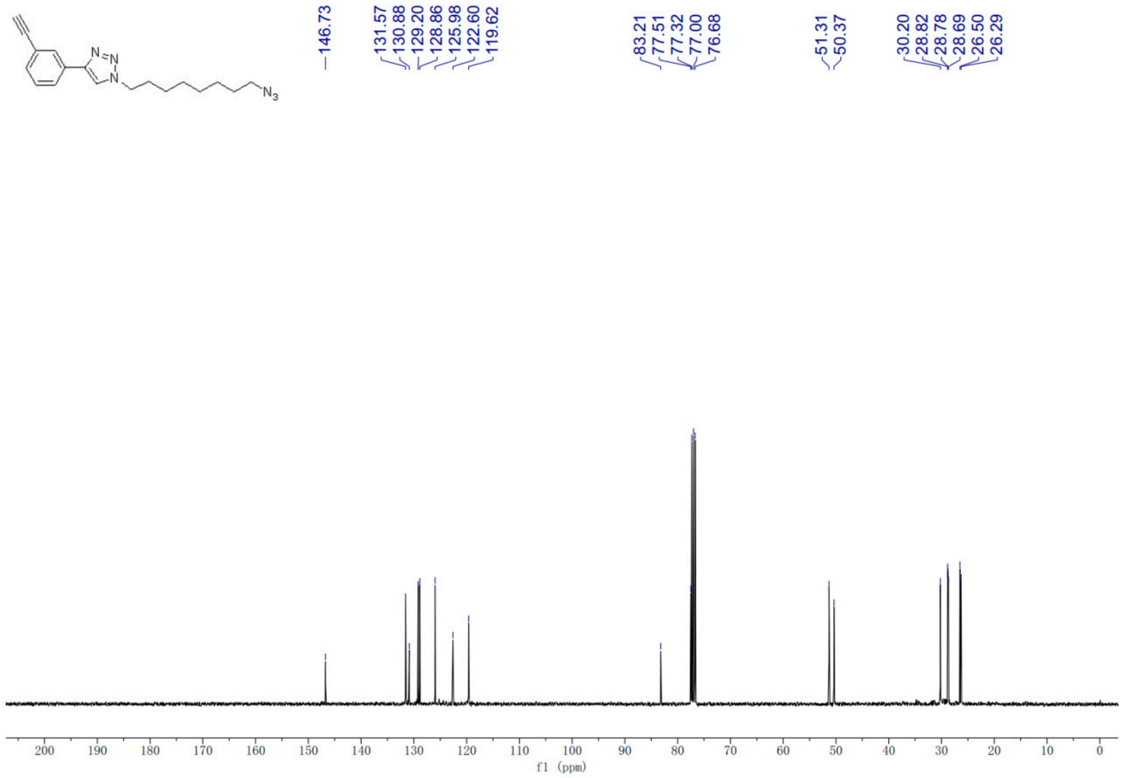

^{1}H NMR (400 MHz, $CDCl_3$) spectrum for 8a

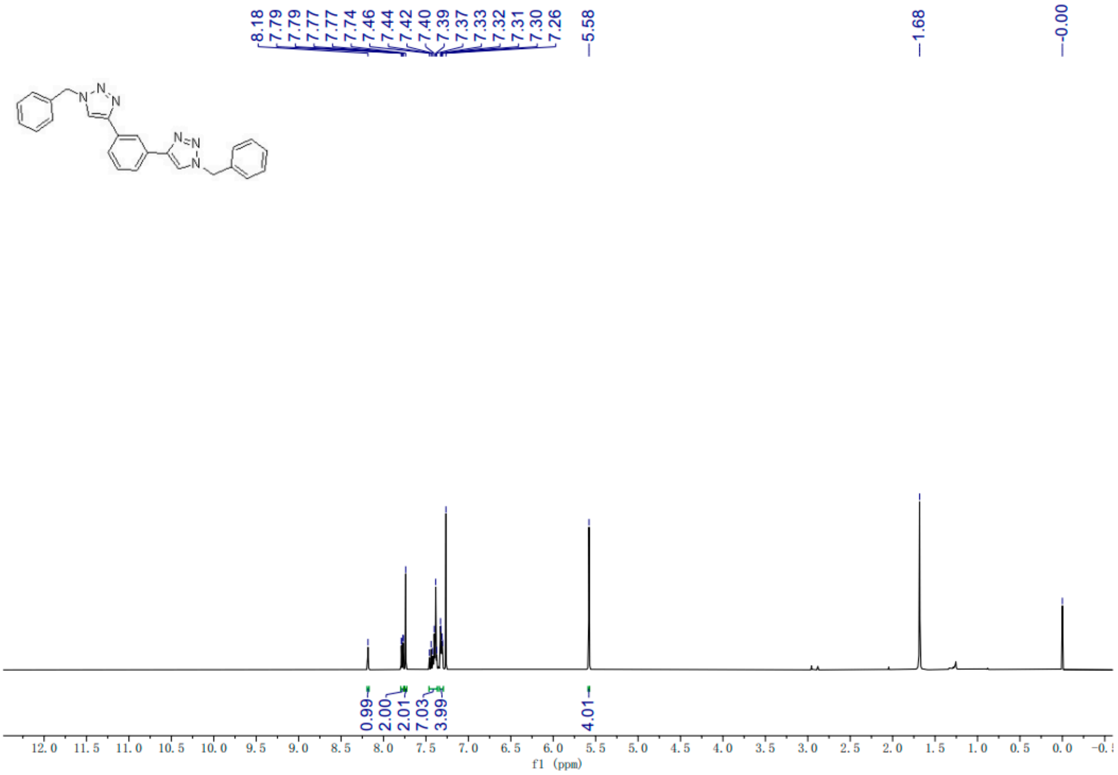

^{1}H NMR (400 MHz, $CDCl_3$) spectrum for 8b

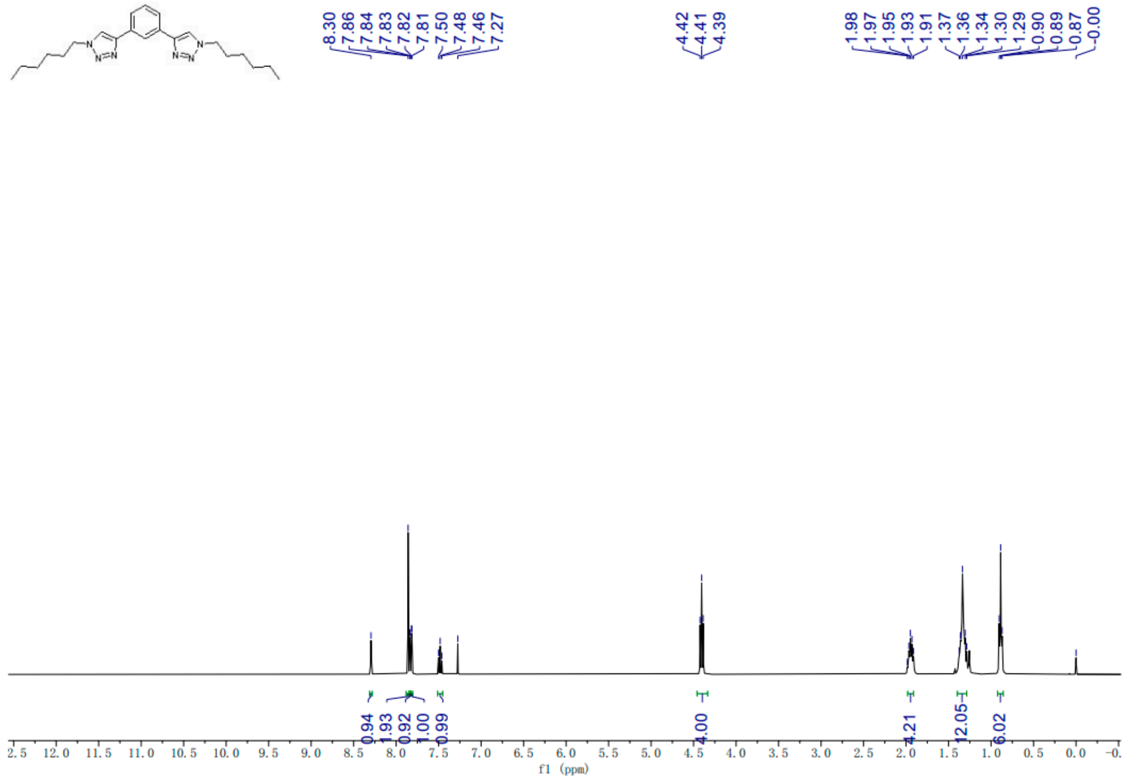

^{1}H NMR (400 MHz, $CDCl_3$) spectrum for 8c

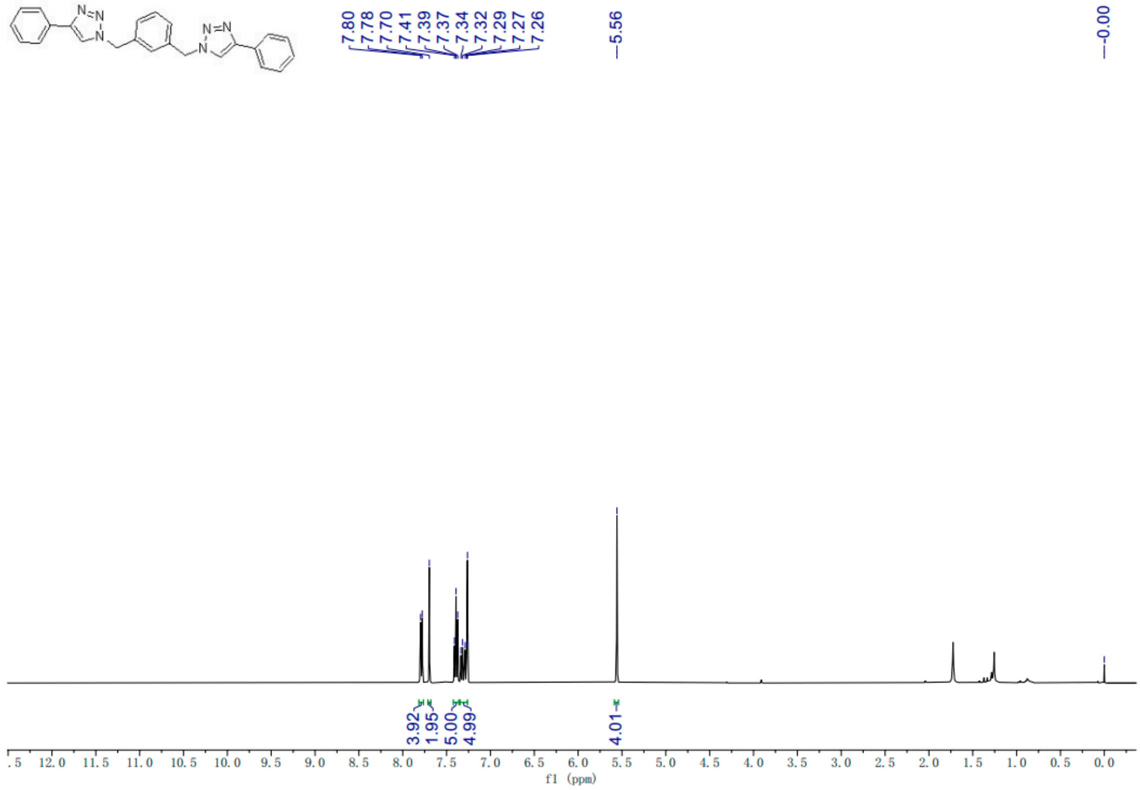

^{1}H NMR (400 MHz, $CDCl_3$) spectrum for 8d

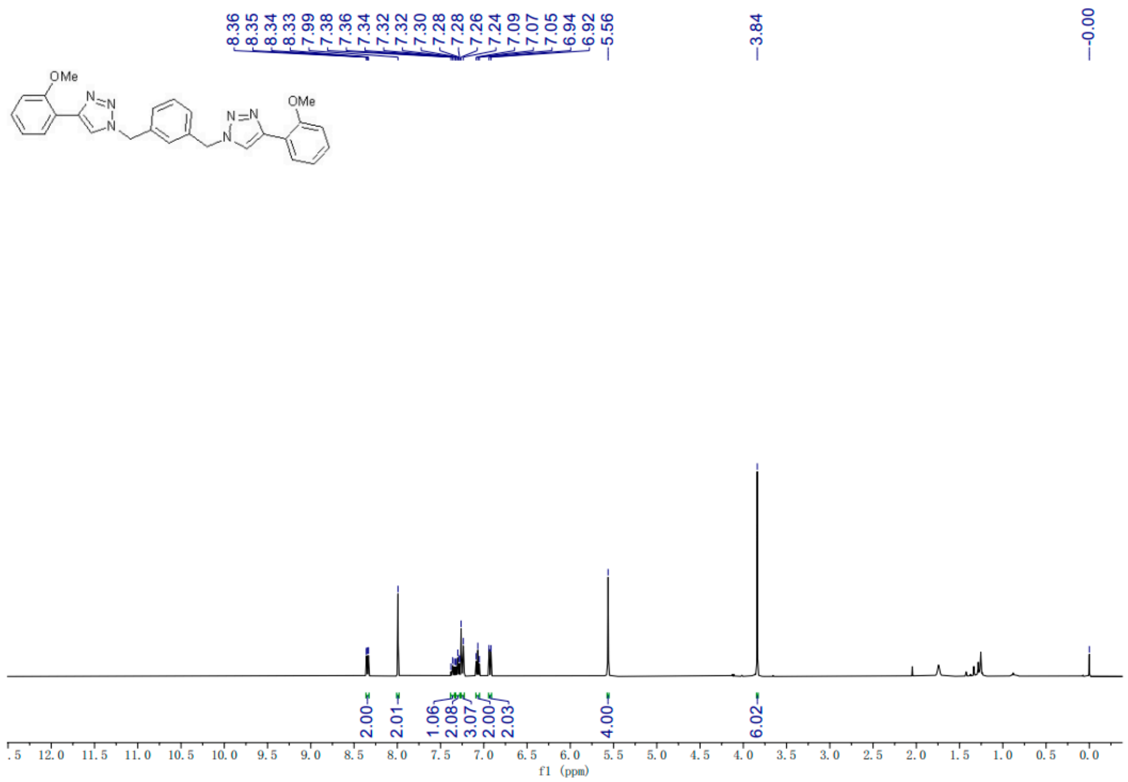

^{13}C NMR (101 MHz, $CDCl_3$) spectrum for 8d

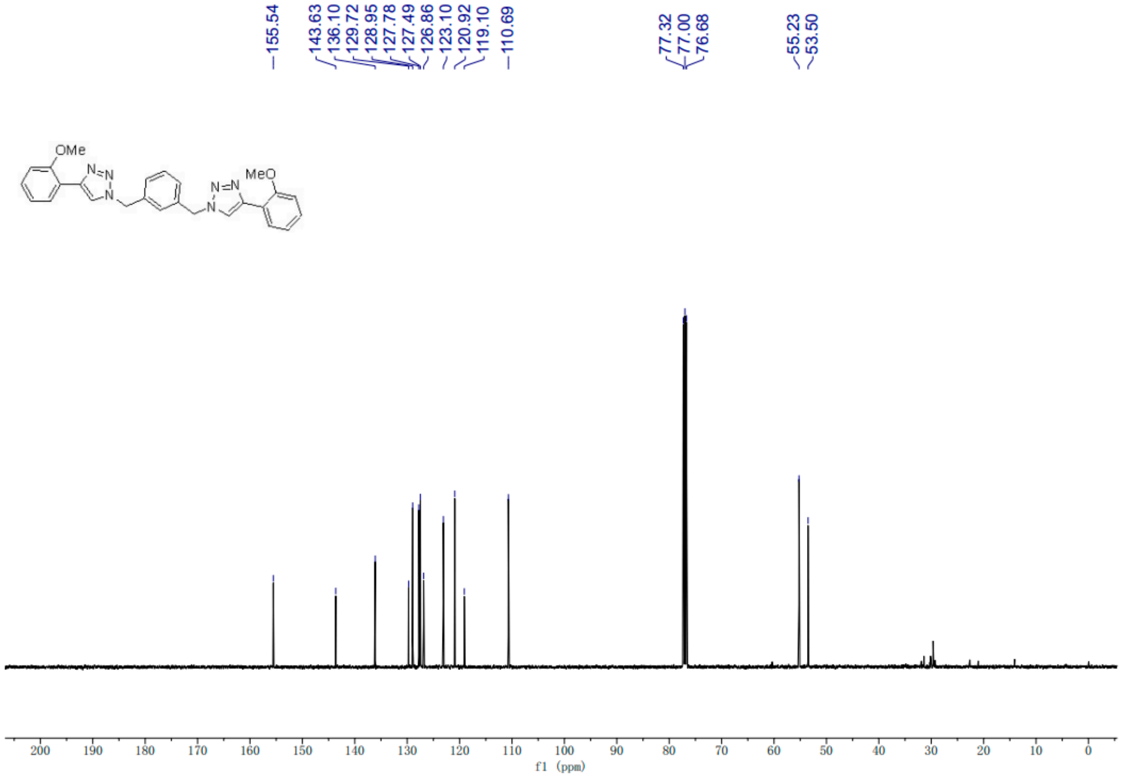

^{1}H NMR (400 MHz, $CDCl_3$) spectrum for 8e

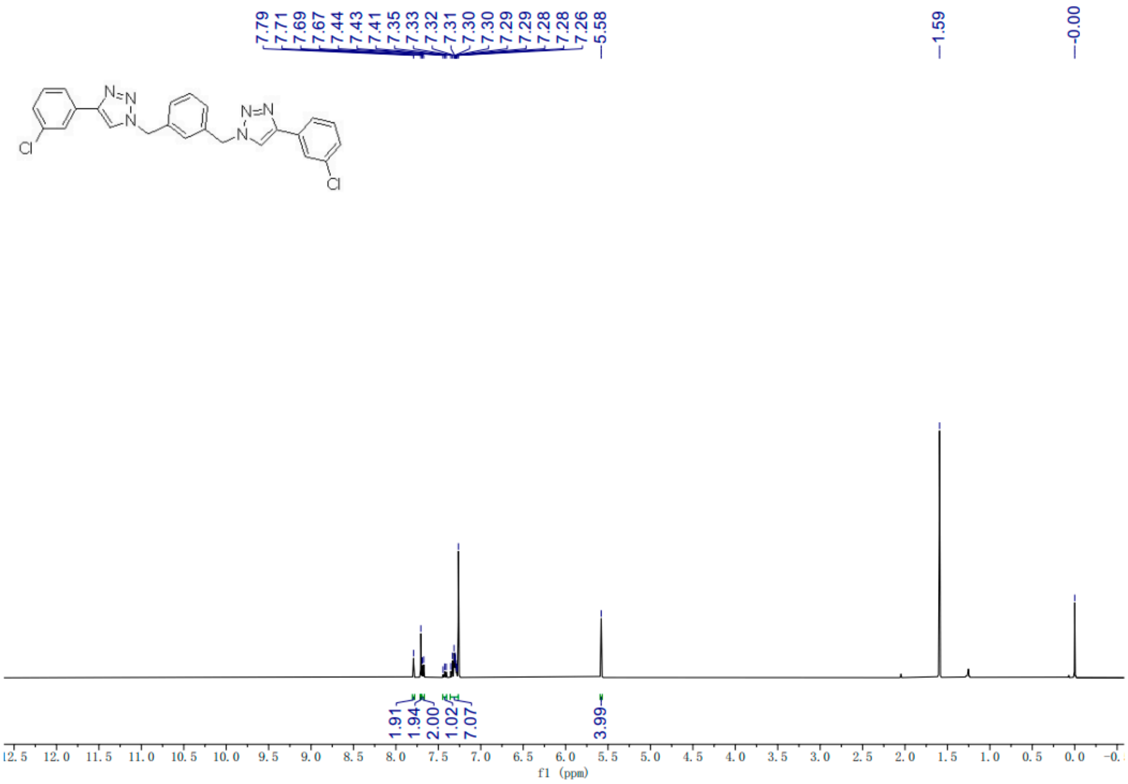

^{13}C NMR (101 MHz, DMSO-d_6) spectrum for 8e

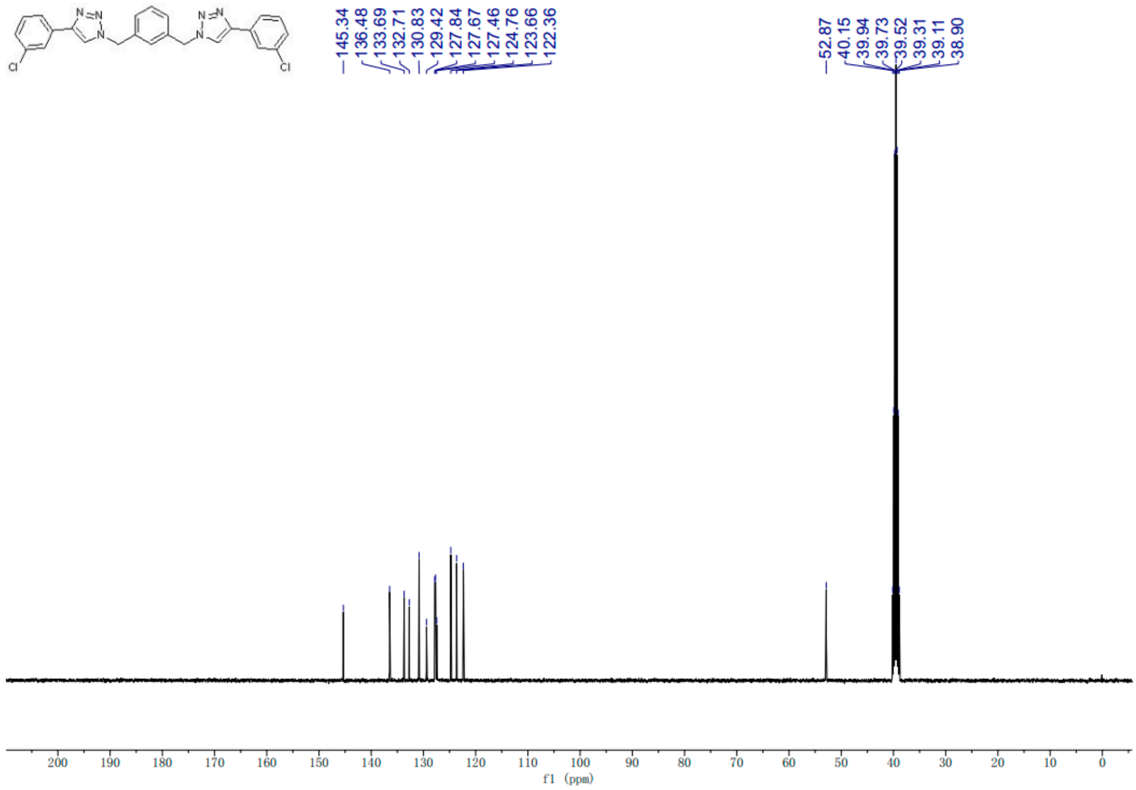

^{1}H NMR (400 MHz, $CDCl_3$) spectrum for 9a

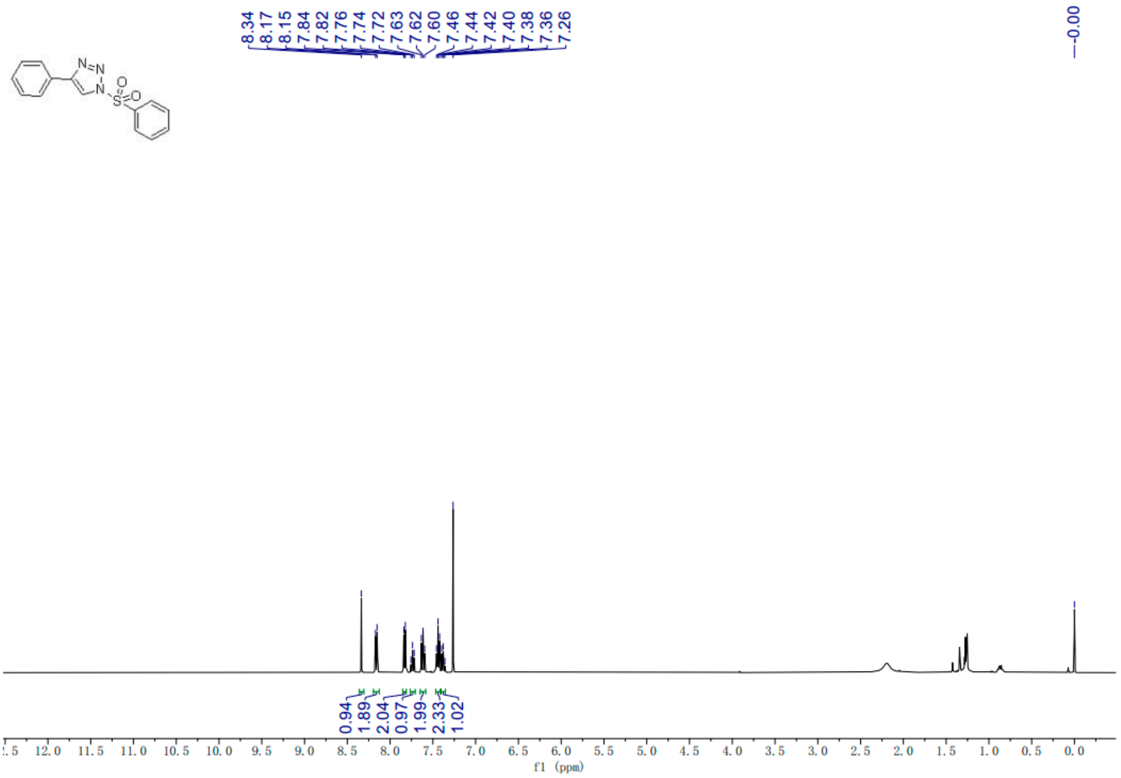

^{1}H NMR (400 MHz, $CDCl_3$) spectrum for 9b

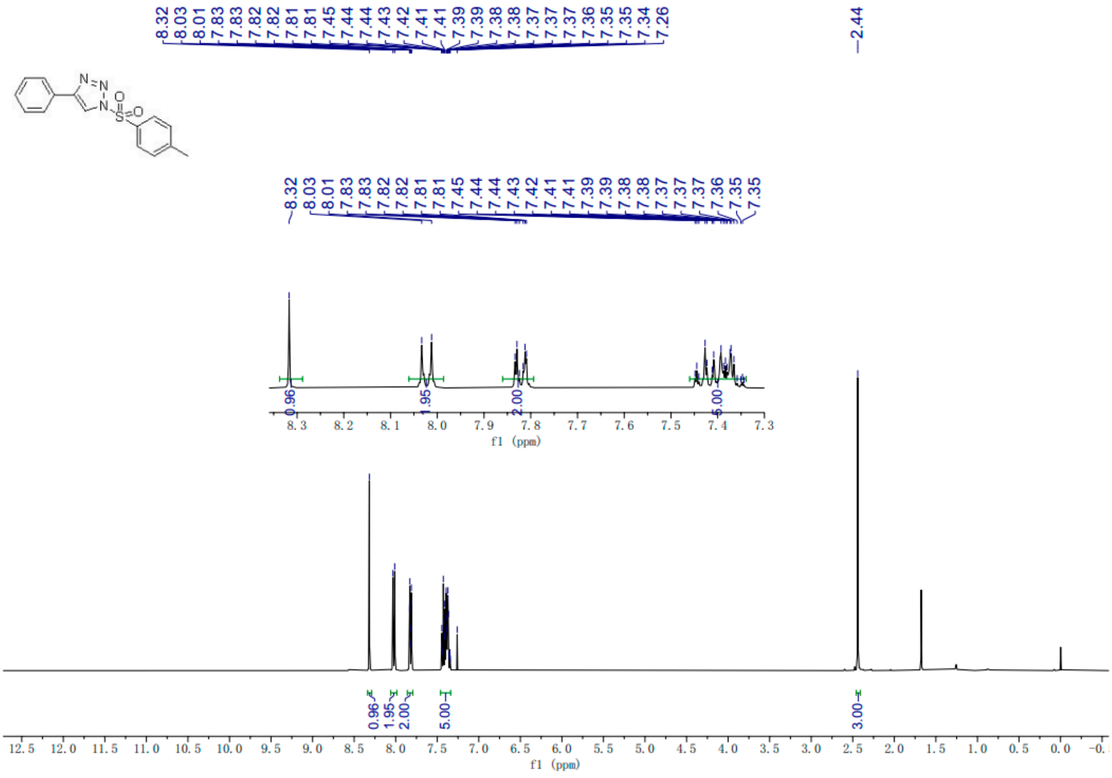

^{1}H NMR (400 MHz, $CDCl_3$) spectrum for 9c

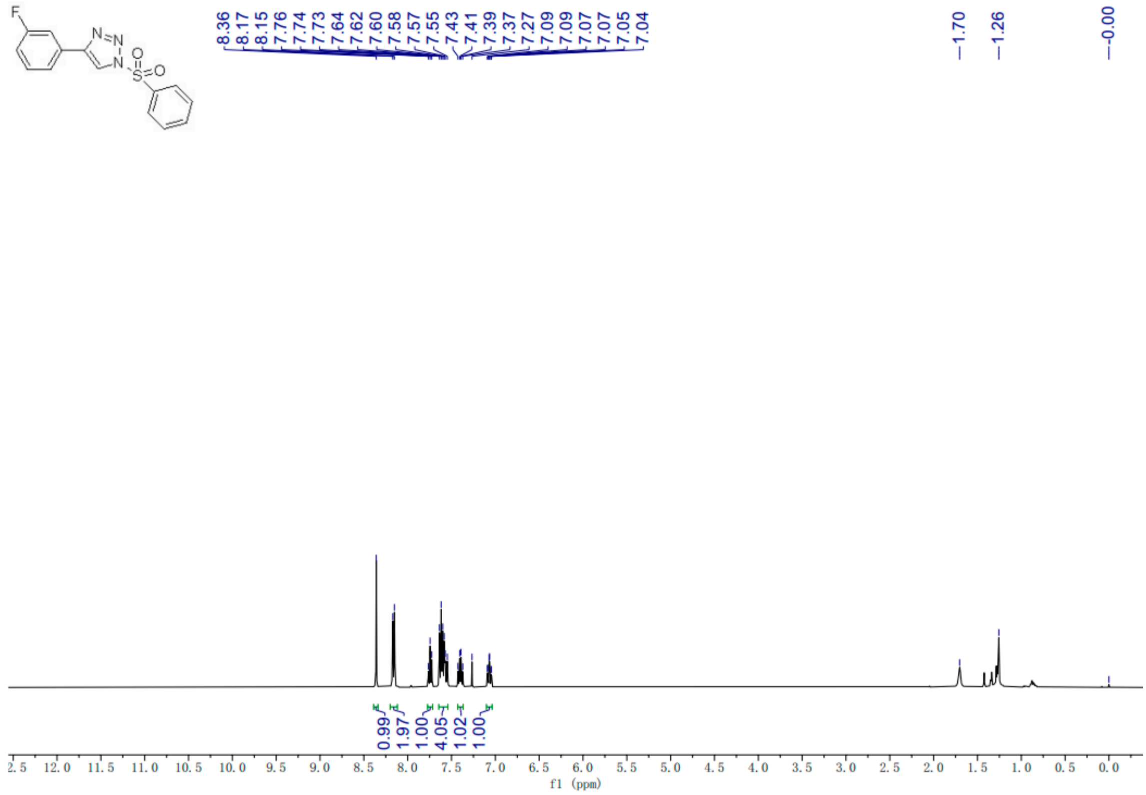

^{13}C NMR (101 MHz, $CDCl_3$) spectrum for 9c

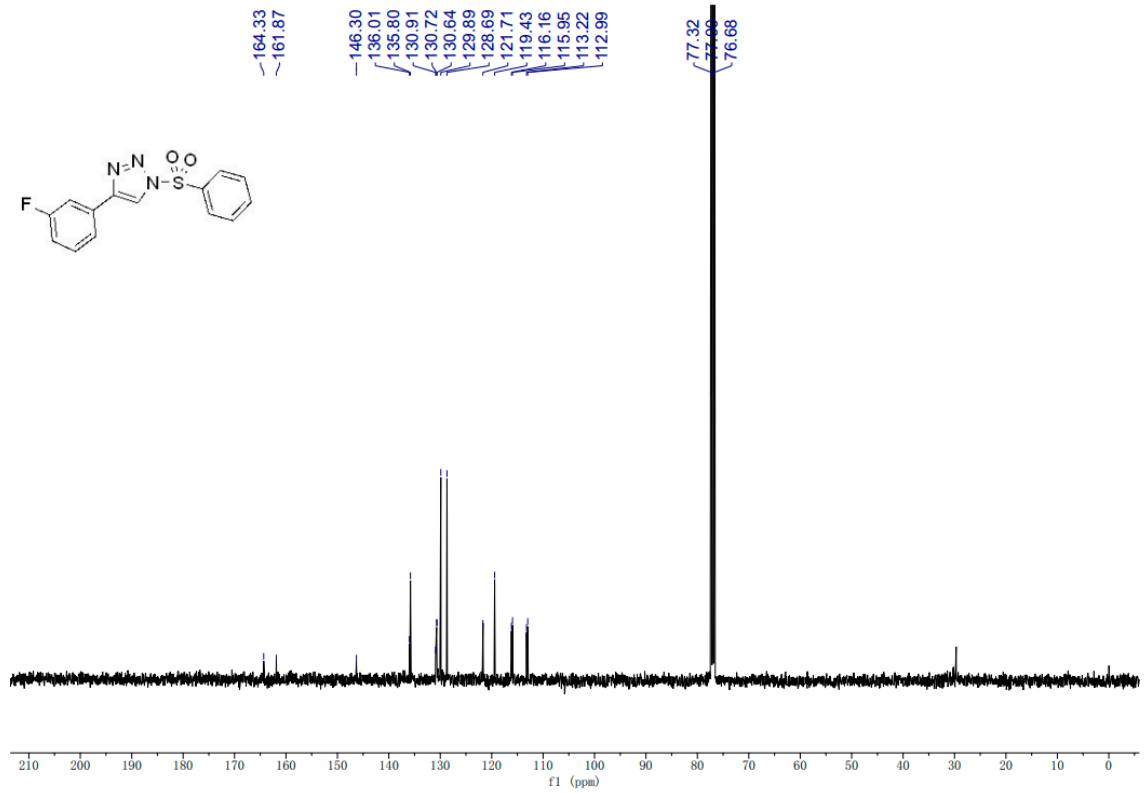

^{19}F NMR (376 MHz, $CDCl_3$) spectrum for 9c

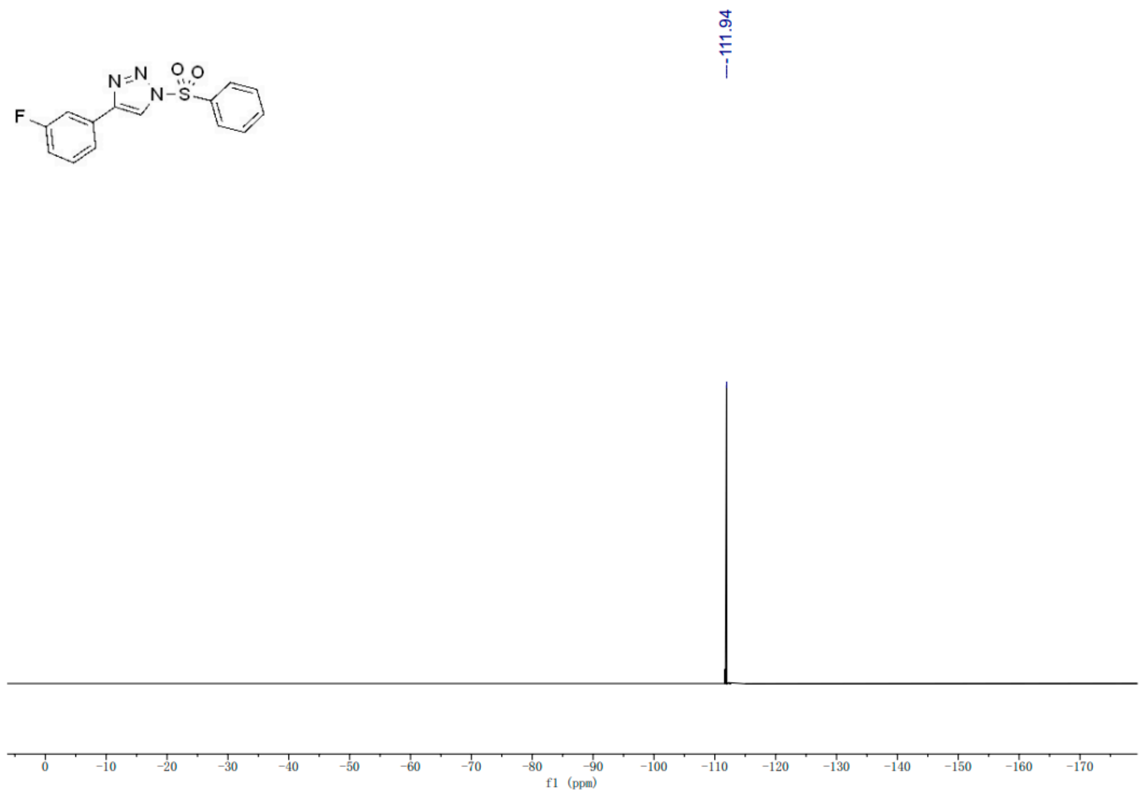

^{1}H NMR (400 MHz, $CDCl_3$) spectrum for 9d

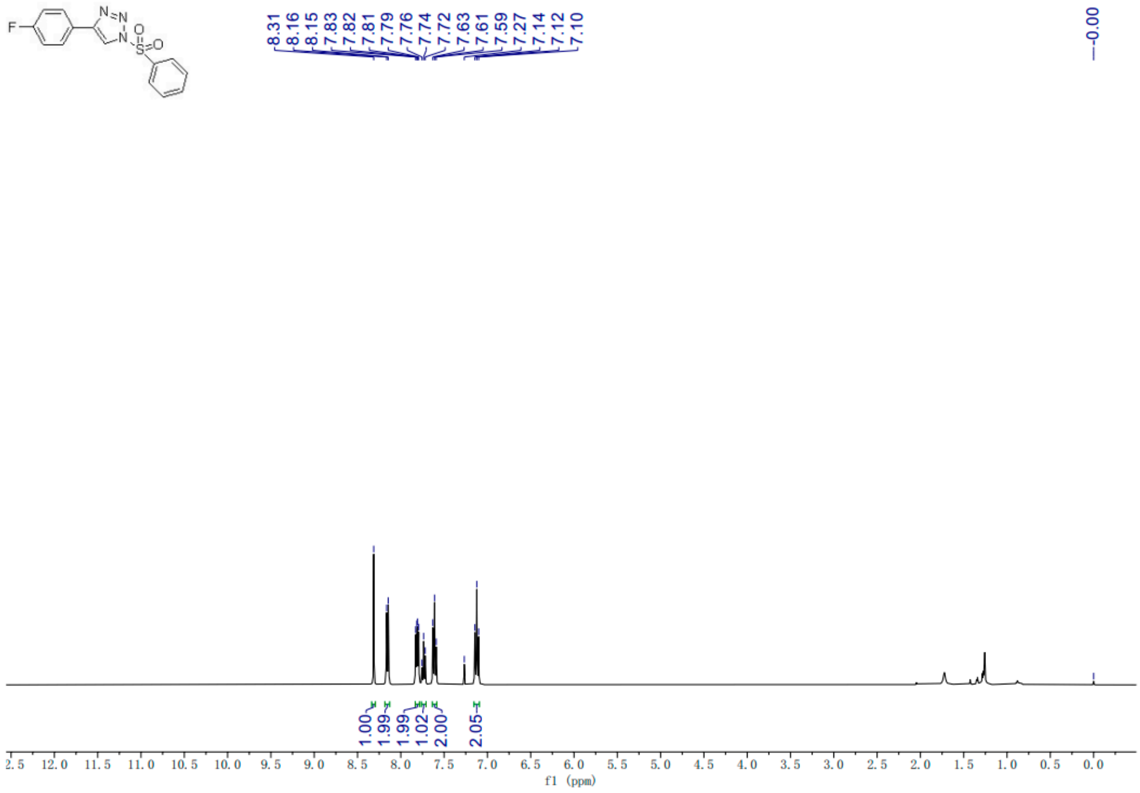

^{13}C NMR (101 MHz, $CDCl_3$) spectrum for 9d

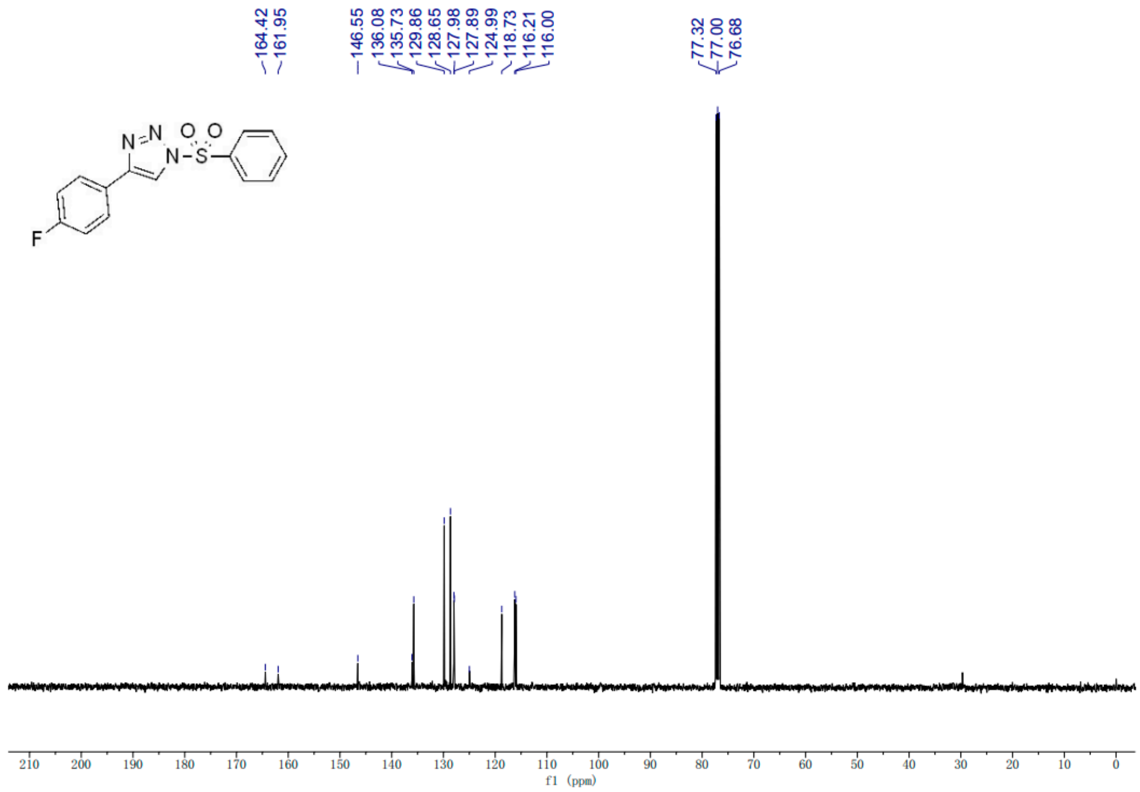

^{19}F NMR (376 MHz, $CDCl_3$) spectrum for 9d

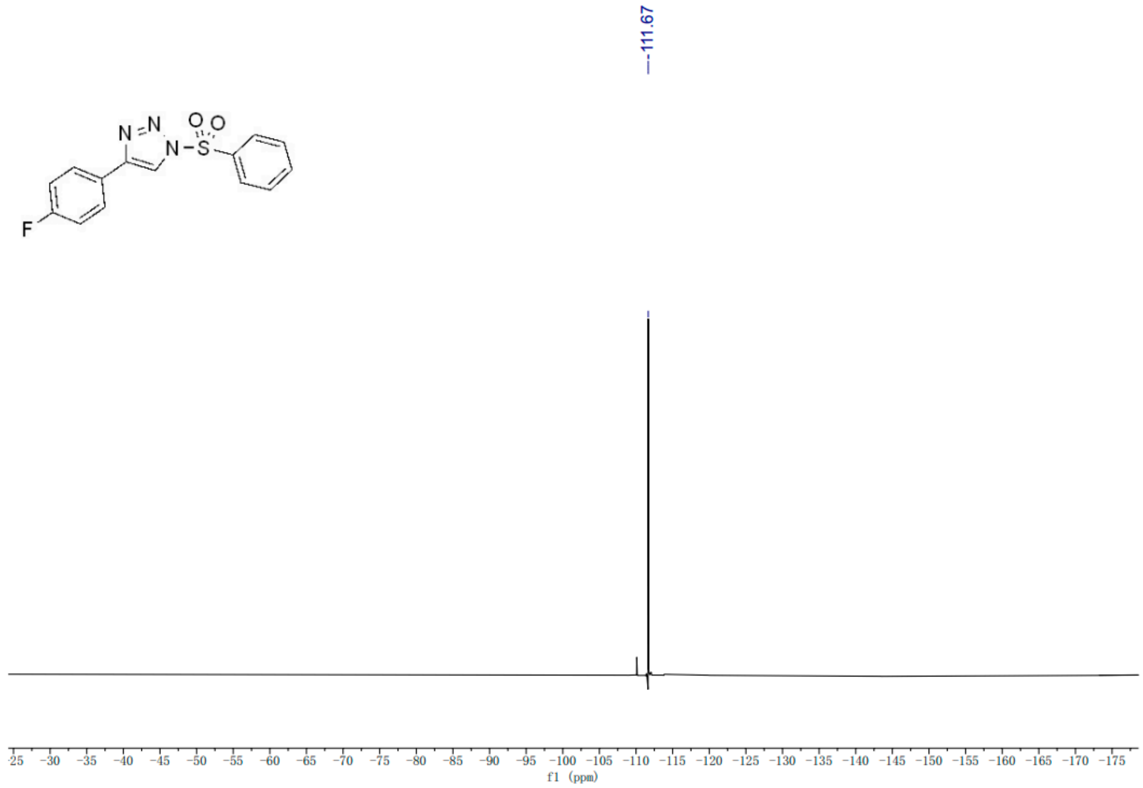

^{1}H NMR (400 MHz, $CDCl_3$) spectrum for 9e

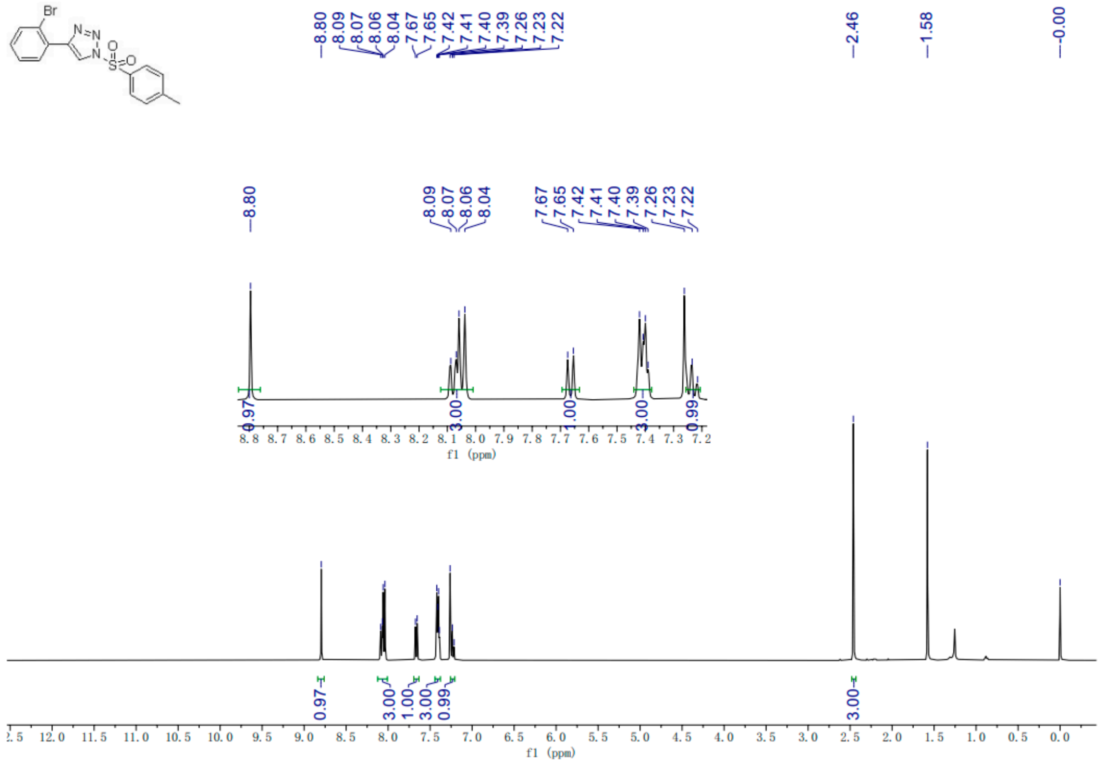

^{1}H NMR (400 MHz, CDCl$_3$) spectrum for 9f

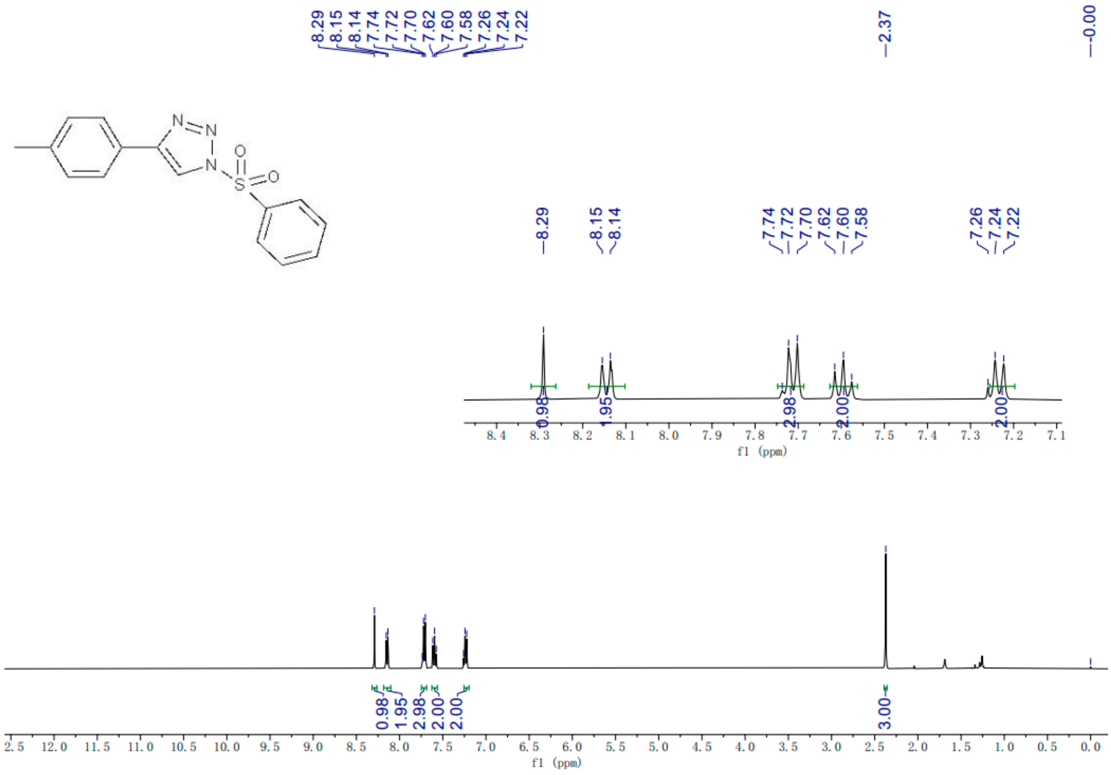

^{13}C NMR (101 MHz, CDCl$_3$) spectrum for 9f

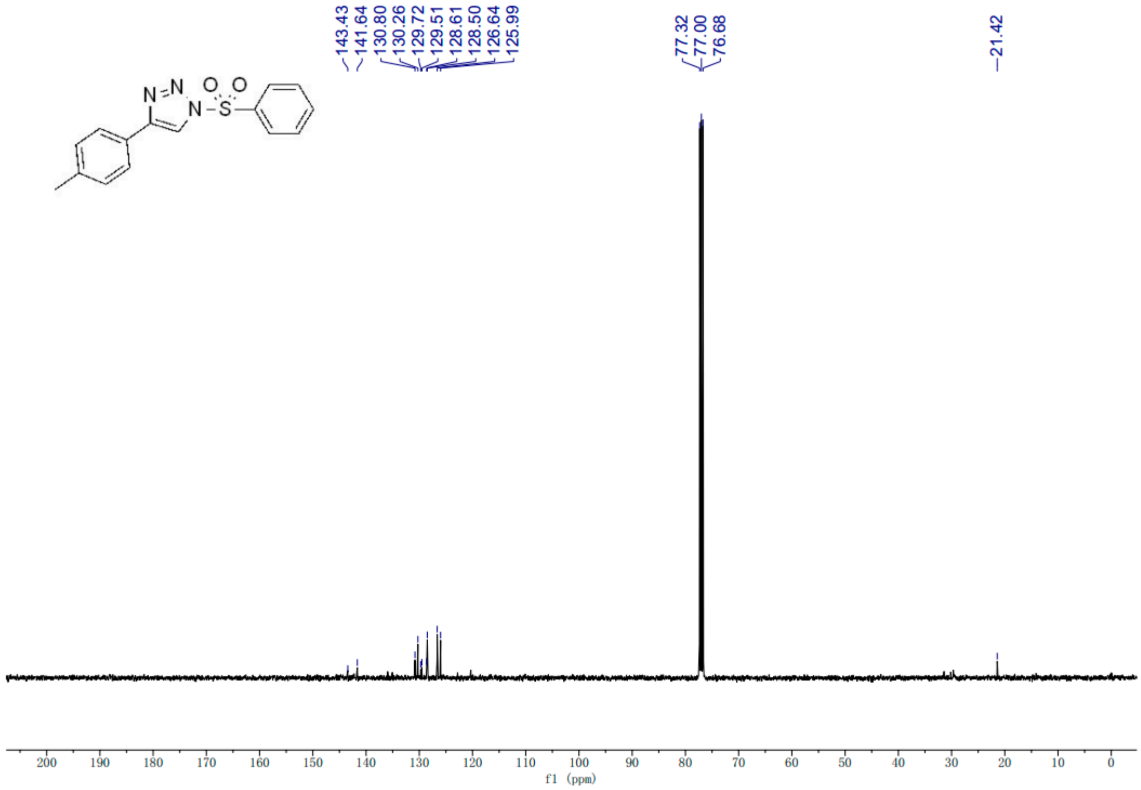

^{1}H NMR (400 MHz, $CDCl_3$) spectrum for 9g

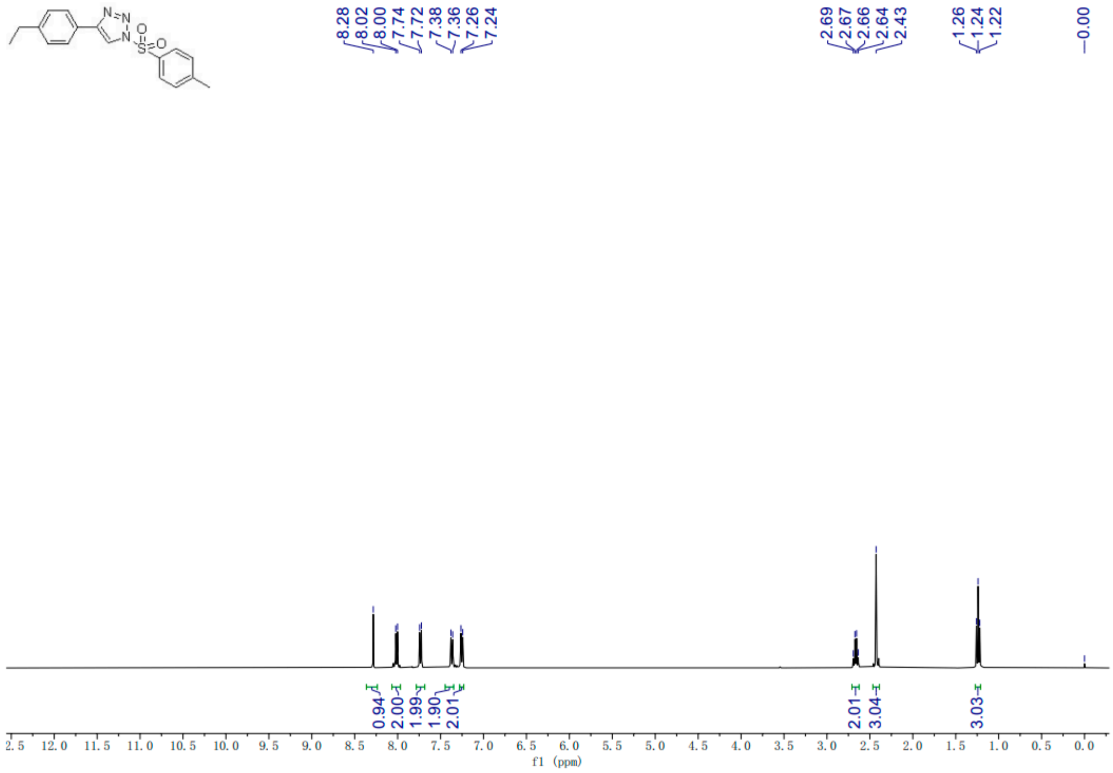

^{1}H NMR (400 MHz, $CDCl_3$) spectrum for 9h

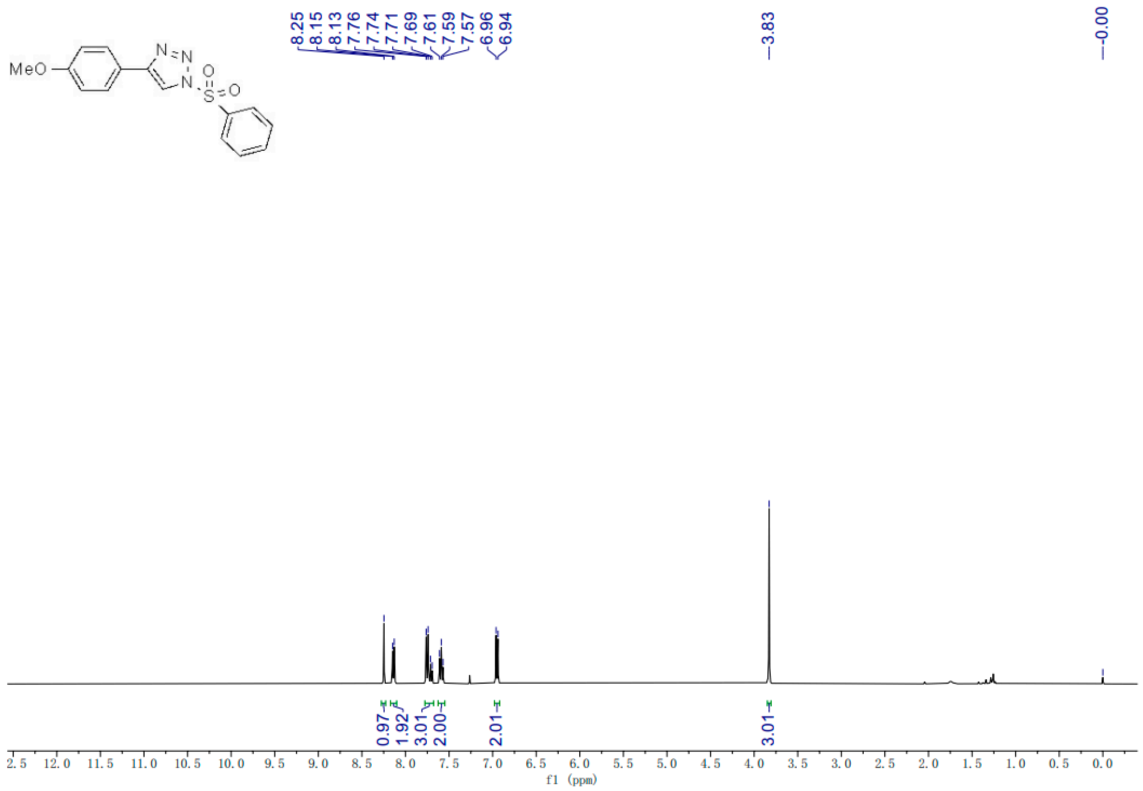

^{13}C NMR (101 MHz, CDCl$_3$) spectrum for 9h

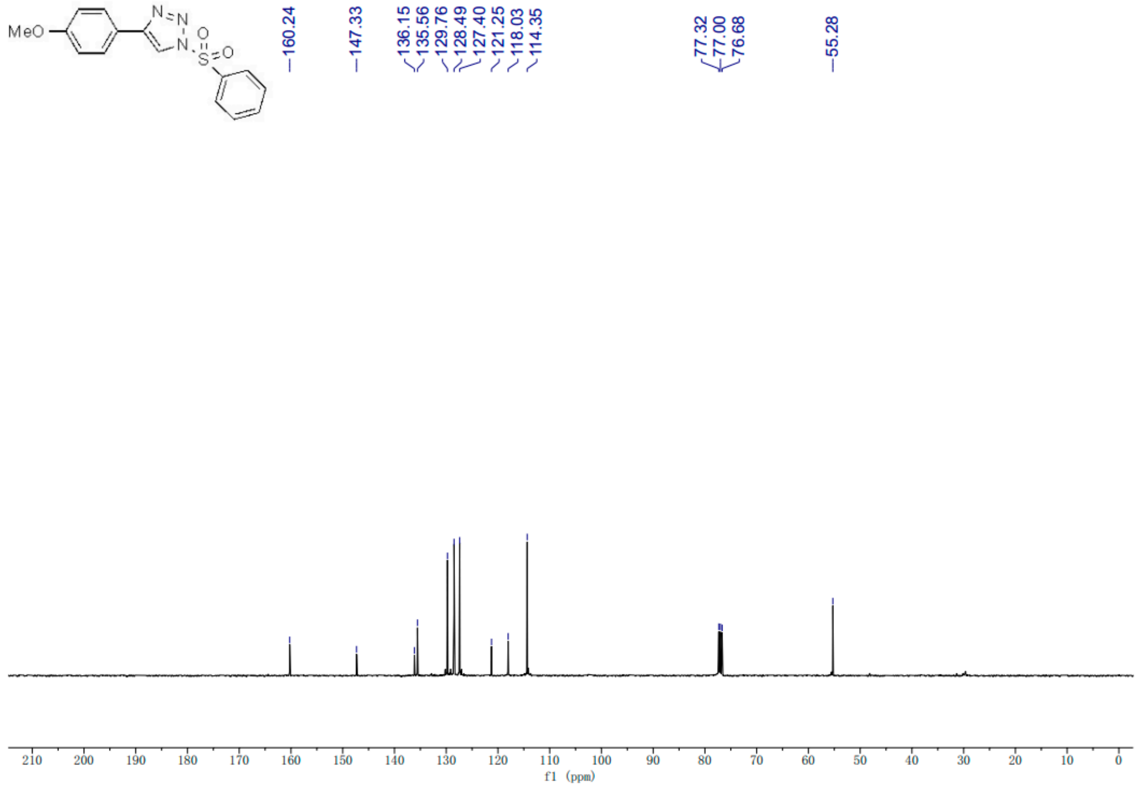

Supplement: Supplementary file 1 [file molecules-31-01723-s001.zip › molecules-4281934-supplementary.pdf]
